# Supplementary material for: Genomic variations in paired normal controls for lung adenocarcinomas
Source: Oncotarget. 2017 Oct 24;8(61):104113–22. doi: 10.18632/oncotarget.22020 (PMC5732791; doi:10.18632/oncotarget.22020)
Supplement: Supplementary file 1 [file oncotarget-08-104113-s001.pdf]

Genomic variations in paired normal controls for lung adenocarcinomas

SUPPLEMENTARY MATERIALS

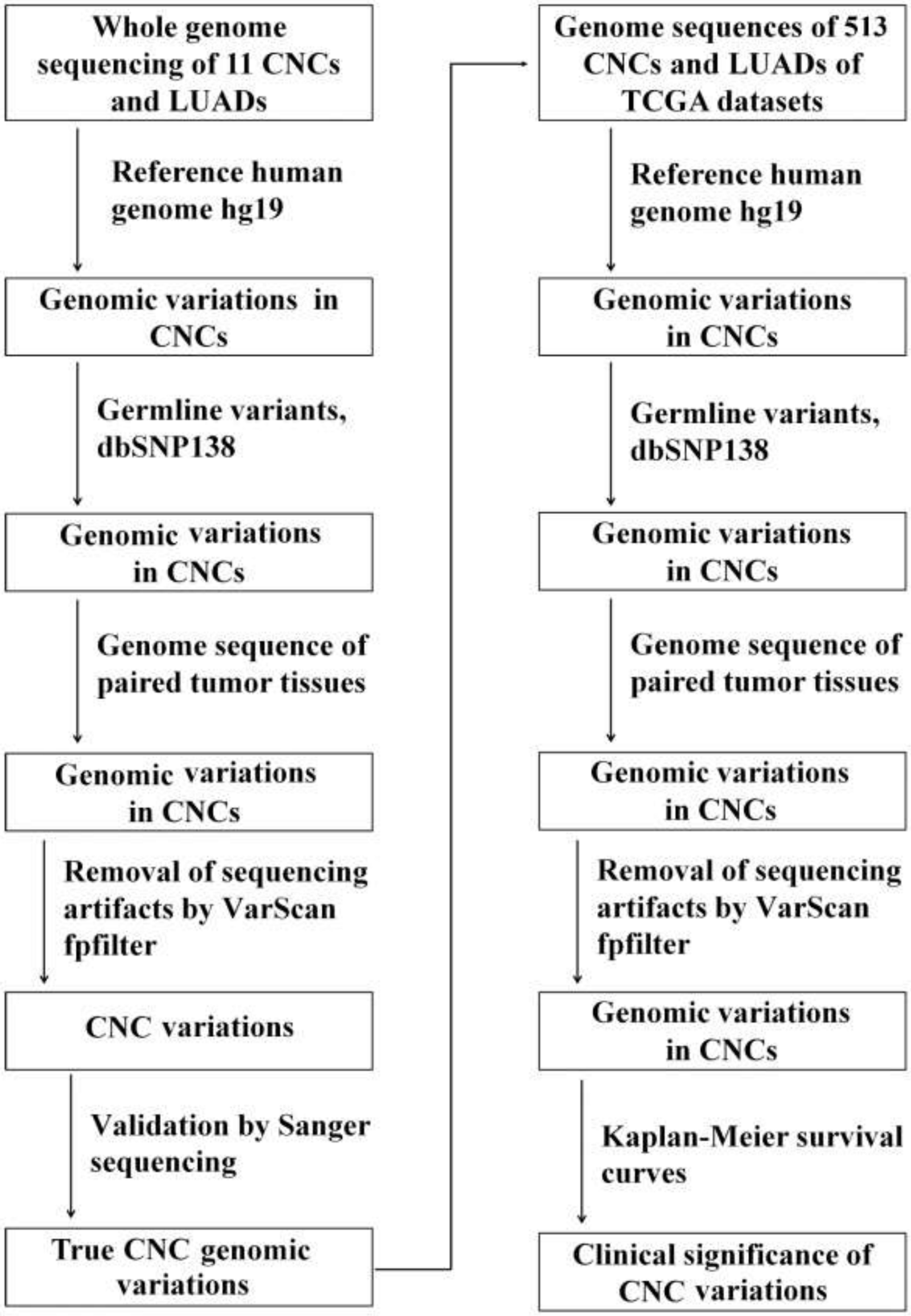

Supplementary Figure 1: Study design of this study.

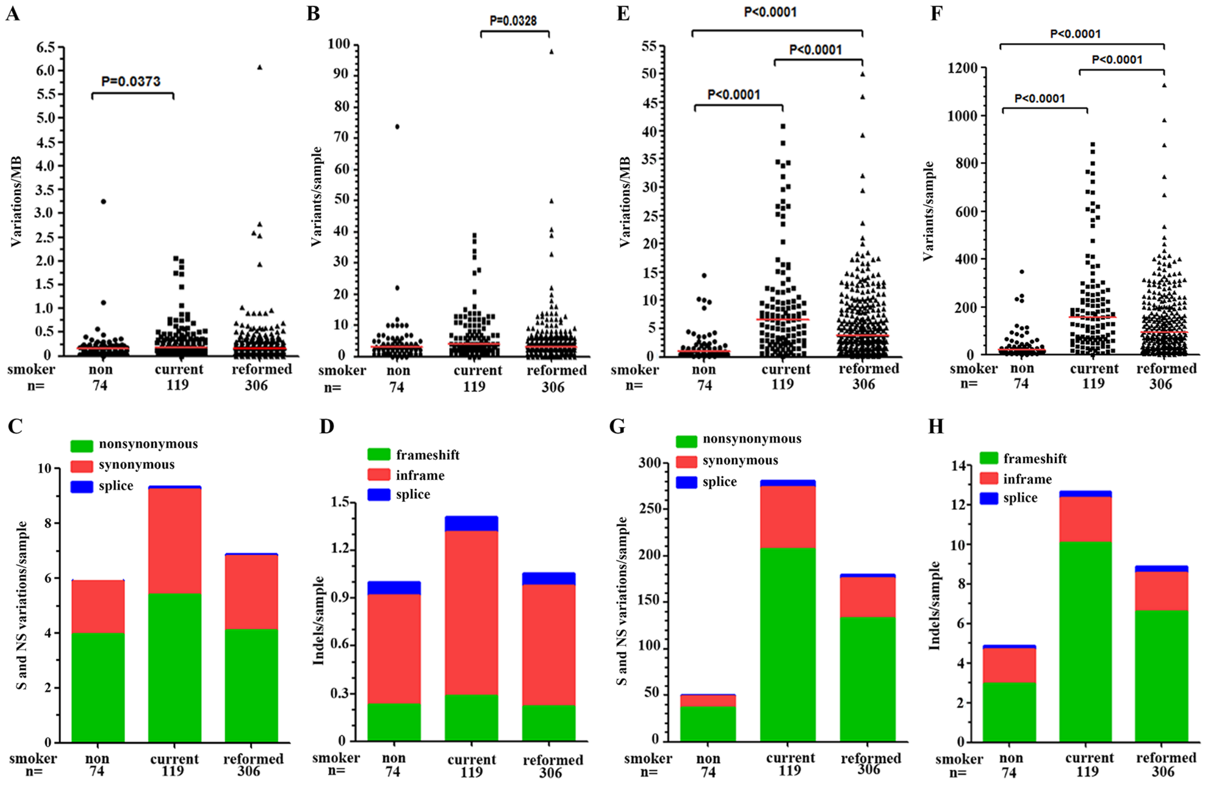

Supplementary Figure 2: Comparison of genomic variations in CNCs and tumors in smokers and nonsmokers. **(A)** Mutation frequency (variations/Mb) in genomes of CNCs of non-smokers, current smokers, and reformed smokers. **(B)** Genomic variations/sample in CNCs of non-smokers, current smokers, and reformed smokers. **(C)** Synonymous and nonsynonymous variations/sample in CNCs of non-smokers, current smokers, and reformed smokers. **(D)** Indels/sample in genomes of CNCs of non-smokers, current smokers, and reformed smokers. **(E)** Mutation frequency (variations/Mb) in genomes of tumor samples of non-smokers, current smokers, and reformed smokers. **(F)** Genomic variations/sample in tumors of non-smokers, current smokers, and reformed smokers. **(G)** Synonymous and nonsynonymous variations/sample in tumors of non-smokers, current smokers, and reformed smokers. **(H)** Indels/sample in genomes of tumors of non-smokers, current smokers, and reformed smokers.

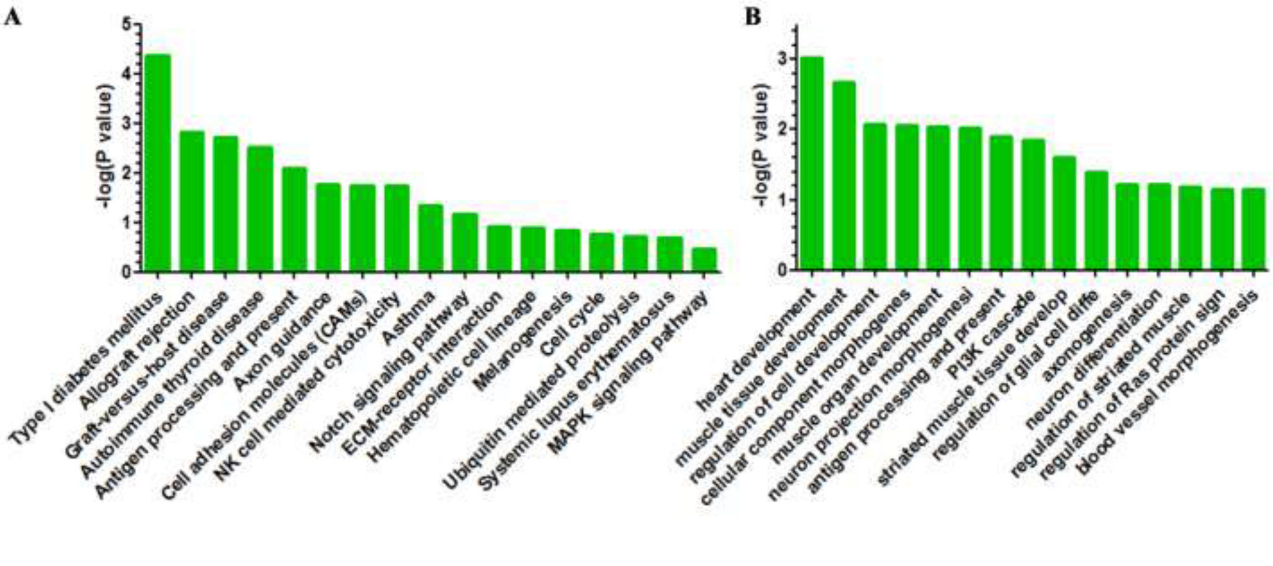

Supplementary Figure 3: The KEGG and GO analyses of the CNC mutations. **(A)** The GO analysis of pathways altered in CNCs. **(B)**. KEGG analysis.

Supplementary Table 1: Sequences of primers used to validate genomic variations in normal lung tissues

Gene name	Sequence
ZNF521	F1: GGATGGAGGACTGGAAGATG
	R1: GGTTATAGAGTGAGGGCAGGA
	F2: ATGGAGGACTGGAAGATGAA
	R2: AAGAGTGTTGAGGTCGTTGA
TMC6	F1: GGCATCTCCCTGGGTAGTGG
	R1: CAAGGCGCTTGTGAGGTAAG
	F2: GCCTGTGGTGAGCCCATCCT
	R2: CAGTCGCCAATCAGCCGTGT
CEP250	F1: AGTCAGTCCTCCCGGCATCA
	R1: GCCTTCTCCCTCCACTCAATCT
	F2: CAGGCAGTGCTCAAGGAACG
	R2: CCCTGGCTTCTGTCTGTCTCA
UNC93A	F1: GGGTTCATGCTATCATCTGG
	R1: CTCCTCGGACCTCACTGCTC
	F2: TTGCTTGGAGTTGTCTTGCCTTTC
	R2: TTGACCTGTCCTGGAGCGTGGG
C9orf66	F1: TGCTCAGGACGACAGAAGGC
	R1: AGAAGGTGGAAATGCGGAAGT
	F2: AAAGGAAGGAGCCGTTTATGAGA
	R2: CGGCTTAGAAGGTGGAAATGC
HIST1H1D	F1: CAATCATCACTCAGCGTCTC
	R1: GCTGGCTTCTTTACCTTCTTA
	F2: AGAGCCTGTGCTATTGTTCC
	R2: AGCTTGATACGGCTGTTGTT

Supplementary Table 2: The characteristics of the 513 patients whose samples were analyzed in this study.

See Supplementary File 1

Supplementary Table 3: Comparison of CNC mutations in normal lung tissues and peripheral blood

	Exonic mutations/MB	Mutated genes/ sample	Nonsynonymous mutations/sample	Synonymous mutations/sample	Rearrangements/sample	
					Frameshift	Inframe
Lung	0.2664	5.3185	4.5111	2.6222	0.2593	0.7185
Blood	0.2741	5.2778	4.3228	2.9180	0.2302	0.8466
P value	0.8605	0.9581	0.7909	0.6185	0.6074	0.2703

Supplementary Table 4: Comparison of CNC mutations in males and females

	Exonic mutations/MB	Mutated genes/ sample	Nonsynonymous mutations/sample	Synonymous mutations/sample	Rearrangements/sample	
					Frameshift	Inframe
Males	0.2661	5.1004	4.1883	2.7615	0.2803	0.8619
Female	0.2773	5.4526	4.5329	2.9088	0.2007	0.7701
P value	0.7704	0.6068	0.5826	0.7788	0.1110	0.3705

Supplementary Table 5: Genomic variations in CNCs of 513 patients with LUAD.

See Supplementary File 2
