# Supplementary material for: Genomic variations in paired normal controls for lung adenocarcinomas
Source: Oncotarget. 2017 Oct 24;8(61):104113–22. doi: 10.18632/oncotarget.22020 (PMC5732791; doi:10.18632/oncotarget.22020)
Supplement: Supplementary file 2 [file oncotarget-08-104113-s002.docx]

**Supplementary Table 2: The characteristics of the 513 patients whose samples were analyzed in this study**

| **Characteristics** | **Total (n=513)** | **Lung tissue (n=135)** | **Blood (n=378)** |
| --- | --- | --- | --- |
| Gender | | | |
| Male | 239 | 58 | 181 |
| Female | 274 | 77 | 197 |
| Age (years) | | | |
| <65 | 219 | 61 | 158 |
| ≥65 | 275 | 74 | 201 |
| n.d. | 19 | 0 | 19 |
| Median (range) | 66 (33 – 68) | 66 (39 – 86) | 66 (33 – 68) |
| Race | | | |
| White | 389 | 110 | 279 |
| Black | 50 | 21 | 29 |
| Asian | 8 | 2 | 6 |
| n.d. | 66 | 2 | 64 |
| Smoking | | | |
| Smoker | 425 | 114 | 311 |
| Non-smoker | 74 | 14 | 60 |
| n.d. | 14 | 7 | 7 |
| Histology | | | |
| Adenocarcinoma | 481 | 128 | 353 |
| Bronchioloalveolar carcinoma | 23 | 4 | 19 |
| Mucinous (colloid) carcinoma | 9 | 3 | 6 |
| Survival |  |  |  |
| Alive | 388 | 79 | 309 |
| Dead | 125 | 56 | 69 |
| Anatomic subdivision | | | |
| R-lower | 96 | 20 | 76 |
| R-middle | 23 | 7 | 16 |
| R-upper | 182 | 42 | 140 |
| L-lower | 77 | 20 | 57 |
| L-middle | 0 | 0 | 0 |
| L-upper | 122 | 39 | 83 |
| Bronchial | 1 | 0 | 1 |
| n.d. | 12 | 7 | 5 |
| TNM stage | | | |
| I | 5 | 2 | 3 |
| IA | 131 | 35 | 96 |
| IB | 139 | 35 | 104 |
| II | 1 | 1 | 0 |
| IIA | 53 | 12 | 41 |
| IIB | 73 | 20 | 53 |
| IIIA | 73 | 23 | 50 |
| IIIB | 11 | 1 | 10 |
| IV | 27 | 6 | 21 |

n.d., not determined
