# Supplementary material for: Genomic variations in paired normal controls for lung adenocarcinomas
Source: Oncotarget. 2017 Oct 24;8(61):104113–22. doi: 10.18632/oncotarget.22020 (PMC5732791; doi:10.18632/oncotarget.22020)
Supplement: Supplementary file 3 [file oncotarget-08-104113-s003.docx]

**Supplementary Table 5: Genomic variations in CNCs of 513 patients with LUAD**

| **Patient ID** | **CNC** | **Gene** | **Chr** | **Start** | **End** | **Reference** | **Alt** | **Function** | **Tanscriptor** | **cDNA position** | **AA position** |
| --- | --- | --- | --- | --- | --- | --- | --- | --- | --- | --- | --- |
| 100 | LUNG | KAT2B | 3 | 20082154 | 20082154 | G | A | nonsynonymous | NM_003884 | c.G185A | p.G62D |
| 100 | LUNG | SKA3 | 13 | 21729950 | 21729950 | G | A | nonsynonymous | NM_145061 | c.C1120T | p.L374F |
| 100 | LUNG | ZNF814 | 19 | 58385748 | 58385748 | G | A | nonsynonymous | NM_001144989 | c.C1010T | p.A337V |
| 101 | BLOOD | KRTAP5-1 | 11 | 1606146 | 1606146 | - | GCC | inframe | NM_001005922 | c.333_334insGGC | p.C112delinsGC |
| 101 | BLOOD | HSPG2 | 1 | 22186113 | 22186113 | T | G | nonsynonymous | NM_001291860 | c.A5242C | p.T1748P |
| 103 | LUNG | MAP3K4 | 6 | 161413044 | 161413044 | - | CCG | inframe | NM_005922 | c.81_82insCCG | p.P27delinsPP |
| 103 | LUNG | SGK223 | 8 | 8176387 | 8176387 | - | GGGGCG | inframe | NM_001080826 | c.3497_3498insCGCCCC | p.P1166delinsPAP |
| 103 | LUNG | MUC4 | 3 | 195505836 | 195505836 | G | C | nonsynonymous | NM_018406 | c.C12615G | p.H4205Q |
| 103 | LUNG | MUC4 | 3 | 195508249 | 195508249 | A | G | nonsynonymous | NM_018406 | c.T10202C | p.V3401A |
| 103 | LUNG | DND1 | 5 | 140052430 | 140052430 | G | T | nonsynonymous | NM_194249 | c.C204A | p.D68E |
| 103 | LUNG | FAT3 | 11 | 92600263 | 92600263 | C | A | nonsynonymous | NM_001008781 | c.C12015A | p.F4005L |
| 103 | LUNG | GAS6 | 13 | 114537583 | 114537583 | T | G | nonsynonymous | NM_000820 | c.A775C | p.T259P |
| 104 | LUNG | PITRM1 | 10 | 3208567 | 3208567 | - | GCACGCTAGGGAAGAGAGA | stopgain | NM_014889 | c.271_272insTCTCTCTTCCCTAGCGTGC | p.Q91_F92delinsLSLPX |
| 104 | LUNG | KRT4 | 12 | 53207583 | 53207583 | - | CACCAAAGCCACCAGTGCCGAAACCAGC | frameshift | NM_002272 | c.259_260insGCTGGTTTCGGCACTGGTGGCTTTGGTG | p.G87fs |
| 104 | LUNG | POU5F2 | 5 | 93076551 | 93076551 | A | G | nonsynonymous | NM_153216 | c.T719C | p.I240T |
| 104 | LUNG | BAG4 | 8 | 38034599 | 38034599 | G | A | nonsynonymous | NM_004874 | c.G212A | p.G71D |
| 104 | LUNG | ULK1 | 12 | 132395337 | 132395337 | T | C | nonsynonymous | NM_003565 | c.T940C | p.S314P |
| 104 | LUNG | PLEKHG3 | 14 | 65209993 | 65209993 | A | G | nonsynonymous | NM_015549 | c.A3064G | p.R1022G |
| 104 | LUNG | FOXO4 | X | 70320825 | 70320825 | G | A | nonsynonymous | NM_005938 | c.G745A | p.E249K |
| 105 | BLOOD | SSPO | 7 | 149503917 | 149503920 | GATA | - | frameshift | NM_198455 | c.8745_8745del | p.G2915fs |
| 105 | BLOOD | ZFHX3 | 16 | 72831357 | 72831357 | - | TTGTTG | inframe | NM_006885 | c.5223_5224insCAACAA | p.A1742delinsQQA |
| 105 | BLOOD | MUC4 | 3 | 195505836 | 195505836 | G | C | nonsynonymous | NM_018406 | c.C12615G | p.H4205Q |
| 105 | BLOOD | LEMD2 | 6 | 33756859 | 33756859 | T | C | nonsynonymous | NM_181336 | c.A35G | p.E12G |
| 105 | BLOOD | PLAC4 | 21 | 42551222 | 42551222 | T | G | nonsynonymous | NM_182832 | c.A334C | p.I112L |
| 105 | BLOOD | SLC16A2 | X | 73641569 | 73641569 | T | C | nonsynonymous | NM_006517 | c.T97C | p.S33P |
| 106 | BLOOD | KRTAP4-1 | 17 | 39340796 | 39340852 | CGGCAGCAGCTGGACATACCACAGCTGGGGTGGCAGGTGGTCTGACAGCAGAGTGGG | - | inframe | NM_033060 | c.246_254del | p.82_85del |
| 106 | BLOOD | MUC4 | 3 | 195512830 | 195512830 | A | G | nonsynonymous | NM_018406 | c.T5621C | p.L1874P |
| 107 | BLOOD | PPM1N | 19 | 46002238 | 46002238 | G | A | nonsynonymous | NM_001080401 | c.G508A | p.V170I |
| 107 | BLOOD | POTED | 21 | 15011856 | 15011856 | G | A | nonsynonymous | NM_174981 | c.G1430A | p.R477Q |
| 108 | BLOOD | RPTN | 1 | 152129066 | 152129101 | TGGTGGGAATCTCTGTCTTGTTTCTCAGACTGACCA | - | inframe | NM_001122965 | c.474_509del | p.158_170del |
| 108 | BLOOD | PRR21 | 2 | 240982213 | 240982213 | C | G | nonsynonymous | NM_001080835 | c.G187C | p.A63P |
| 108 | BLOOD | YIPF5 | 5 | 143540080 | 143540080 | A | C | nonsynonymous | NM_030799 | c.T655G | p.C219G |
| 108 | BLOOD | TNKS | 8 | 9413579 | 9413579 | A | C | nonsynonymous | NM_003747 | c.A130C | p.T44P |
| 108 | BLOOD | SRSF9 | 12 | 120899912 | 120899912 | G | T | stopgain | NM_003769 | c.C576A | p.Y192X |
| 108 | BLOOD | NAP1L3 | X | 92928135 | 92928135 | C | T | nonsynonymous | NM_004538 | c.G169A | p.G57S |
| 109 | BLOOD | POU4F2 | 4 | 147560457 | 147560457 | - | GGC | inframe | NM_004575 | c.165_166insGGC | p.G55delinsGG |
| 109 | BLOOD | MUC4 | 3 | 195511814 | 195511814 | T | C | nonsynonymous | NM_018406 | c.A6637G | p.S2213G |
| 10 | BLOOD | MAP3K1 | 5 | 56177849 | 56177851 | CAA | - | inframe | NM_005921 | c.2822_2824del | p.941_942del |
| 10 | BLOOD | ZNF667 | 19 | 56952770 | 56952771 | AT | - | frameshift | NM_022103 | c.1593_1594del | p.T531fs |
| 10 | BLOOD | MAP4 | 3 | 47957741 | 47957741 | T | C | nonsynonymous | NM_002375 | c.A1576G | p.T526A |
| 10 | BLOOD | LAMB2 | 3 | 49160649 | 49160649 | G | T | nonsynonymous | NM_002292 | c.C4140A | p.N1380K |
| 10 | BLOOD | DNAH1 | 3 | 52392595 | 52392595 | G | A | nonsynonymous | NM_015512 | c.G4108A | p.E1370K |
| 10 | BLOOD | TLR10 | 4 | 38774816 | 38774816 | C | T | nonsynonymous | NM_030956 | c.G2396A | p.R799Q |
| 10 | BLOOD | TLX1 | 10 | 102891390 | 102891390 | G | A | nonsynonymous | NM_005521 | c.G92A | p.G31D |
| 10 | BLOOD | ZNF814 | 19 | 58385546 | 58385546 | G | T | nonsynonymous | NM_001144989 | c.C1212A | p.D404E |
| 110 | BLOOD | PSPH | 7 | 56088902 | 56088902 | C | T | nonsynonymous | NM_004577 | c.G4A | p.V2I |
| 110 | BLOOD | FANK1 | 10 | 127585212 | 127585212 | A | T | nonsynonymous | NM_145235 | c.A1T | p.M1L |
| 111 | LUNG | NEGR1 | 1 | 72748110 | 72748110 | C | T | nonsynonymous | NM_173808 | c.G68A | p.S23N |
| 111 | LUNG | ATP1A2 | 1 | 160093040 | 160093040 | G | T | nonsynonymous | NM_000702 | c.G215T | p.R72L |
| 111 | LUNG | PARP14 | 3 | 122433259 | 122433259 | T | C | nonsynonymous | NM_017554 | c.T3983C | p.L1328P |
| 111 | LUNG | UBE2D3 | 4 | 103730849 | 103730849 | G | T | nonsynonymous | NM_181891 | c.C100A | p.Q34K |
| 111 | LUNG | COBL | 7 | 51287624 | 51287624 | C | G | nonsynonymous | NM_015198 | c.G59C | p.R20P |
| 111 | LUNG | BAG4 | 8 | 38034599 | 38034599 | G | A | nonsynonymous | NM_004874 | c.G212A | p.G71D |
| 111 | LUNG | ANKRD30A | 10 | 37421172 | 37421172 | G | A | nonsynonymous | NM_052997 | c.G347A | p.S116N |
| 111 | LUNG | KRT85 | 12 | 52758850 | 52758850 | G | T | stopgain | NM_002283 | c.C525A | p.Y175X |
| 111 | LUNG | MAPK6 | 15 | 52356350 | 52356350 | G | T | nonsynonymous | NM_002748 | c.G1319T | p.C440F |
| 111 | LUNG | MYO1C | 17 | 1386153 | 1386153 | A | C | splicing |  |  |  |
| 111 | LUNG | SUV39H1 | X | 48564946 | 48564946 | C | G | nonsynonymous | NM_003173 | c.C1033G | p.P345A |
| 112 | LUNG | KRT4 | 12 | 53207583 | 53207583 | - | CACCAAAGCCACCAGTGCCGAAACCAG | inframe | NM_002272 | c.259_260insCTGGTTTCGGCACTGGTGGCTTTGGTG | p.G87delinsAGFGTGGFGG |
| 112 | LUNG | DCAKD | 17 | 43112232 | 43112233 | CT | - | frameshift | NM_001288654 | c.21_22del | p.T7fs |
| 112 | LUNG | PLSCR4 | 3 | 145924511 | 145924511 | G | T | stopgain | NM_020353 | c.C156A | p.Y52X |
| 112 | LUNG | BEGAIN | 14 | 101004782 | 101004782 | C | T | nonsynonymous | NM_020836 | c.G1306A | p.A436T |
| 112 | LUNG | CELSR1 | 22 | 46790131 | 46790131 | A | C | nonsynonymous | NM_014246 | c.T5872G | p.W1958G |
| 113 | BLOOD | IRF5 | 7 | 128587352 | 128587381 | ACTCTGCAGCCGCCCACTCTGCGGCCGCCT | - | inframe | NM_001098630 | c.502_531del | p.168_177del |
| 113 | BLOOD | CHD5 | 1 | 6181605 | 6181605 | C | A | nonsynonymous | NM_015557 | c.G4728T | p.M1576I |
| 113 | BLOOD | DND1 | 5 | 140052407 | 140052407 | G | A | nonsynonymous | NM_194249 | c.C227T | p.P76L |
| 114 | LUNG | THAP11 | 16 | 67876824 | 67876826 | CAG | - | inframe | NM_020457 | c.367_369del | p.123_123del |
| 114 | LUNG | PARP14 | 3 | 122433259 | 122433259 | T | C | nonsynonymous | NM_017554 | c.T3983C | p.L1328P |
| 114 | LUNG | MYO10 | 5 | 16703173 | 16703173 | C | G | nonsynonymous | NM_012334 | c.G2371C | p.A791P |
| 115 | LUNG | BAIAP2L2 | 22 | 38483155 | 38483155 | - | TCATGGGTG | inframe | NM_025045 | c.1234_1235insCACCCATGA | p.N412delinsTPMN |
| 115 | LUNG | CNTN5 | 11 | 99690461 | 99690461 | A | G | nonsynonymous | NM_014361 | c.A242G | p.N81S |
| 115 | LUNG | UPF3A | 13 | 115047496 | 115047496 | G | C | nonsynonymous | NM_023011 | c.G208C | p.V70L,UPF3A |
| 115 | LUNG | MADCAM1 | 19 | 501762 | 501762 | A | C | nonsynonymous | NM_130760 | c.A761C | p.Q254P |
| 116 | LUNG | CREB3L2 | 7 | 137612914 | 137612916 | TGG | - | inframe | NM_194071 | c.299_301del | p.100_101del |
| 116 | LUNG | HLA-DPB1 | 6 | 33048691 | 33048691 | C | G | nonsynonymous | NM_002121 | c.C343G | p.P115A |
| 116 | LUNG | HLA-DPB1 | 6 | 33048694 | 33048694 | A | G | nonsynonymous | NM_002121 | c.A346G | p.M116V |
| 116 | LUNG | SHROOM2 | X | 9914947 | 9914947 | G | C | nonsynonymous | NM_001649 | c.G4821C | p.L1607F |
| 116 | LUNG | HCFC1 | X | 153220360 | 153220360 | A | G | nonsynonymous | NM_005334 | c.T3490C | p.S1164P |
| 117 | BLOOD | MAGEF1 | 3 | 184429133 | 184429133 | - | TCC | inframe | NM_022149 | c.476_477insGGA | p.D159delinsED |
| 117 | BLOOD | PYROXD2 | 10 | 100167734 | 100167735 | CA | - | frameshift | NM_032709 | c.167_168del | p.L56fs |
| 117 | BLOOD | NME4 | 16 | 450140 | 450140 | - | AG | frameshift | NM_001286435 | c.472_473insAG | p.R158fs |
| 117 | BLOOD | OR2T12 | 1 | 248458402 | 248458402 | G | A | nonsynonymous | NM_001004692 | c.C479T | p.A160V |
| 117 | BLOOD | CD96 | 3 | 111342600 | 111342600 | G | A | splicing |  |  |  |
| 117 | BLOOD | FAM86B1 | 8 | 12044029 | 12044029 | G | A | nonsynonymous | NM_001083537 | c.C472T | p.R158W |
| 117 | BLOOD | ITGA7 | 12 | 56101421 | 56101421 | A | C | nonsynonymous | NM_001144996 | c.T46G | p.C16G |
| 117 | BLOOD | MMP19 | 12 | 56236594 | 56236594 | C | T | stopgain | NM_002429 | c.G21A | p.W7X |
| 117 | BLOOD | TPSD1 | 16 | 1306971 | 1306971 | A | G | nonsynonymous | NM_012217 | c.A428G | p.H143R |
| 117 | BLOOD | VWA3A | 16 | 22149798 | 22149798 | C | T | nonsynonymous | NM_173615 | c.C2257T | p.P753S |
| 117 | BLOOD | KRT10 | 17 | 38975147 | 38975147 | G | T | nonsynonymous | NM_000421 | c.C1640A | p.S547Y |
| 117 | BLOOD | SALL3 | 18 | 76753753 | 76753753 | C | T | nonsynonymous | NM_171999 | c.C1762T | p.L588F |
| 118 | BLOOD | IGSF3 | 1 | 117122285 | 117122285 | - | TCC | inframe | NM_001007237 | c.3062_3063insGGA | p.D1021delinsED |
| 118 | BLOOD | DCP1B | 12 | 2062323 | 2062323 | - | TGC | inframe | NM_152640 | c.782_783insGCA | p.Q261delinsQQ |
| 118 | BLOOD | KRTAP4-5 | 17 | 39305775 | 39305775 | - | GGCAGCAGCTGGGGC | inframe | NM_033188 | c.244_245insGCCCCAGCTGCTGCC | p.Q82delinsRPSCCQ |
| 118 | BLOOD | HRC | 19 | 49657889 | 49657889 | - | TCC | inframe | NM_002152 | c.605_606insGGA | p.E202delinsEE |
| 118 | BLOOD | EPHA8 | 1 | 22919852 | 22919852 | G | T | nonsynonymous | NM_020526 | c.G1349T | p.R450L |
| 118 | BLOOD | KCNN3 | 1 | 154842244 | 154842244 | A | T | nonsynonymous | NM_002249 | c.T197A | p.L66H |
| 118 | BLOOD | IQSEC1 | 3 | 12977260 | 12977260 | T | C | nonsynonymous | NM_014869 | c.A1298G | p.E433G |
| 118 | BLOOD | ADAMTS19 | 5 | 128797373 | 128797373 | G | T | nonsynonymous | NM_133638 | c.G652T | p.A218S |
| 118 | BLOOD | ATXN1 | 6 | 16327770 | 16327770 | C | A | nonsynonymous | NM_000332 | c.G772T | p.G258C |
| 118 | BLOOD | HLA-A | 6 | 29910716 | 29910716 | C | G | nonsynonymous | NM_002116 | c.C256G | p.Q86E |
| 118 | BLOOD | CDSN | 6 | 31084964 | 31084964 | T | C | nonsynonymous | NM_001264 | c.A428G | p.N143S |
| 118 | BLOOD | PIM1 | 6 | 37139030 | 37139030 | G | C | nonsynonymous | NM_001243186 | c.G643C | p.E215Q |
| 118 | BLOOD | MYOM2 | 8 | 2050520 | 2050520 | G | A | nonsynonymous | NM_003970 | c.G2683A | p.V895M |
| 118 | BLOOD | NOXA1 | 9 | 140323373 | 140323373 | C | A | nonsynonymous | NM_001256067 | c.C409A | p.L137I |
| 118 | BLOOD | ABCC9 | 12 | 22028630 | 22028630 | C | T | nonsynonymous | NM_005691 | c.G2050A | p.G684S |
| 118 | BLOOD | TNFRSF19 | 13 | 24242181 | 24242181 | C | A | nonsynonymous | NM_018647 | c.C799A | p.L267I |
| 118 | BLOOD | OR1D5 | 17 | 2966273 | 2966273 | G | A | nonsynonymous | NM_014566 | c.C629T | p.P210L |
| 118 | BLOOD | FAM83G | 17 | 18881605 | 18881605 | C | A | nonsynonymous | NM_001039999 | c.G1374T | p.Q458H |
| 118 | BLOOD | LRRC30 | 18 | 7231740 | 7231740 | G | A | nonsynonymous | NM_001105581 | c.G604A | p.A202T |
| 118 | BLOOD | FCGBP | 19 | 40389741 | 40389741 | A | G | nonsynonymous | NM_003890 | c.T8441C | p.V2814A |
| 118 | BLOOD | FCGBP | 19 | 40392802 | 40392802 | C | T | nonsynonymous | NM_003890 | c.G7702A | p.A2568T |
| 118 | BLOOD | TPTE | 21 | 10969121 | 10969121 | T | G | nonsynonymous | NM_199261 | c.A127C | p.T43P |
| 118 | BLOOD | C21orf2 | 21 | 45755665 | 45755665 | G | A | nonsynonymous | NM_004928 | c.C119T | p.P40L |
| 118 | BLOOD | SLC2A11 | 22 | 24226887 | 24226887 | G | A | nonsynonymous | NM_001024938 | c.G1342A | p.G448R |
| 118 | BLOOD | SLC5A4 | 22 | 32643460 | 32643460 | C | A | stopgain | NM_014227 | c.G415T | p.E139X |
| 118 | BLOOD | SLC16A8 | 22 | 38474713 | 38474713 | T | G | splicing |  |  |  |
| 118 | BLOOD | DMD | X | 32380996 | 32380996 | C | T | nonsynonymous | NM_000109 | c.G5210A | p.R1737H |
| 118 | BLOOD | LANCL3 | X | 37527659 | 37527659 | T | C | nonsynonymous | NM_198511 | c.T1142C | p.L381P |
| 118 | BLOOD | HDX | X | 83723541 | 83723541 | A | G | nonsynonymous | NM_001177479 | c.T1190C | p.F397S |
| 118 | BLOOD | ARMCX5-GPRASP2,GPRASP2 | X | 101970316 | 101970316 | A | C | nonsynonymous | NM_001184875 | c.A519C | p.R173S |
| 118 | BLOOD | TCEAL5 | X | 102529190 | 102529190 | C | G | nonsynonymous | NM_001012979 | c.G302C | p.R101P |
| 118 | BLOOD | XIAP | X | 123034511 | 123034511 | A | C | nonsynonymous | NM_001167 | c.A1268C | p.Q423P |
| 118 | BLOOD | MAGEC1 | X | 140993852 | 140993852 | C | G | nonsynonymous | NM_005462 | c.C662G | p.T221S |
| 118 | BLOOD | MAGEC1 | X | 140993859 | 140993859 | T | A | nonsynonymous | NM_005462 | c.T669A | p.S223R |
| 118 | BLOOD | MAGEC1 | X | 140993864 | 140993864 | T | C | nonsynonymous | NM_005462 | c.T674C | p.F225S |
| 119 | LUNG | TBP | 6 | 170871013 | 170871013 | - | CAG | inframe | NM_003194 | c.189_190insCAG | p.Q63delinsQQ |
| 119 | LUNG | ODF1 | 8 | 103573011 | 103573037 | TGCAACCCCTGCAGCCCCTGCAACCCG | - | inframe | NM_024410 | c.652_678del | p.218_226del |
| 119 | LUNG | WDR66 | 12 | 122359397 | 122359397 | - | GAGGAGGAGGAGAAA | inframe | NM_144668 | c.186_187insGAGGAGGAGGAGAAA | p.G62delinsGEEEEK |
| 119 | LUNG | HDGFRP2 | 19 | 4499633 | 4499647 | AGCTGGCCGGGGAGG | - | frameshift | NM_001001520 | c.1721_1722del | p.K574fs |
| 119 | LUNG | NEFH | 22 | 29885859 | 29885876 | GCTAAGTCCCCAGAGAAG | - | inframe | NM_021076 | c.2230_2247del | p.744_749del |
| 119 | LUNG | LEXM | 1 | 55273304 | 55273304 | C | T | nonsynonymous | NM_152607 | c.C298T | p.R100W |
| 119 | LUNG | FMN2 | 1 | 240371462 | 240371462 | G | T | nonsynonymous | NM_001305424 | c.G3362T | p.G1121V |
| 119 | LUNG | WDR27 | 6 | 170059557 | 170059557 | G | A | nonsynonymous | NM_182552 | c.C1187T | p.A396V |
| 11 | BLOOD | KRTAP5-1 | 11 | 1606121 | 1606150 | CCACAGCCACCCTTGGATCCCCCACAAGAG | - | inframe | NM_001005922 | c.330_359del | p.110_120del |
| 11 | BLOOD | KRTAP5-5 | 11 | 1651191 | 1651199 | GGCTGTGGA | - | inframe | NM_001001480 | c.121_129del | p.41_43del |
| 11 | BLOOD | UTS2B | 3 | 190995923 | 190995923 | C | T | nonsynonymous | NM_198152 | c.G140A | p.R47H |
| 11 | BLOOD | MAGEC3 | X | 140967165 | 140967165 | T | C | nonsynonymous | NM_138702 | c.T463C | p.S155P |
| 120 | LUNG | PIGF | 2 | 46839443 | 46839443 | T | C | nonsynonymous | NM_002643 | c.A361G | p.T121A |
| 120 | LUNG | HLA-DQB2 | 6 | 32725559 | 32725559 | C | T | nonsynonymous | NM_001300790 | c.G748A | p.G250S |
| 120 | LUNG | CYP2A13 | 19 | 41595958 | 41595958 | C | T | nonsynonymous | NM_000766 | c.C350T | p.A117V |
| 121 | LUNG | DNAH1 | 3 | 52385992 | 52385992 | A | G | nonsynonymous | NM_015512 | c.A2744G | p.N915S |
| 122 | LUNG | MADCAM1 | 19 | 501786 | 501786 | C | A | nonsynonymous | NM_130760 | c.C785A | p.P262Q |
| 123 | LUNG | POLR1B | 2 | 113300002 | 113300026 | TCCGGCGTGTACCGAGAGACTGGCG | - | frameshift | NM_001282772 | c.45_69del | p.L15fs |
| 124 | LUNG | TPSAB1 | 16 | 1291972 | 1291972 | C | G | nonsynonymous | NM_003294 | c.C644G | p.T215S |
| 124 | LUNG | TPSAB1 | 16 | 1291975 | 1291975 | G | A | nonsynonymous | NM_003294 | c.G647A | p.R216Q |
| 124 | LUNG | ARMC5 | 16 | 31475864 | 31475864 | C | T | nonsynonymous | NM_001301820 | c.C1616T | p.P539L |
| 124 | LUNG | CES1 | 16 | 55862824 | 55862824 | C | T | nonsynonymous | NM_001025194 | c.G112A | p.V38I |
| 125 | LUNG | MUC3A | 7 | 100550432 | 100550432 | C | T | nonsynonymous | NM_005960 | c.C1013T | p.T338I |
| 125 | LUNG | ANKMY2 | 7 | 16676082 | 16676082 | - | A | splicing |  |  |  |
| 125 | LUNG | FOXE1 | 9 | 100616701 | 100616706 | GCCGCC | - | inframe | NM_004473 | c.505_510del | p.169_170del |
| 126 | LUNG | MUC4 | 3 | 195507062 | 195507062 | C | T | nonsynonymous | NM_018406 | c.G11389A | p.D3797N |
| 127 | LUNG | GRK7 | 3 | 141526640 | 141526640 | G | A | nonsynonymous | NM_139209 | c.G1204A | p.D402N |
| 127 | LUNG | STARD8 | X | 67935175 | 67935175 | A | T | nonsynonymous | NM_001142503 | c.A191T | p.N64I |
| 128 | LUNG | NCOR1 | 17 | 16097870 | 16097870 | C | A | nonsynonymous | NM_006311 | c.G14T | p.G5V |
| 129 | LUNG | MUC4 | 3 | 195515411 | 195515411 | G | T | nonsynonymous | NM_018406 | c.C3040A | p.P1014T |
| 129 | LUNG | ARSD | X | 2836238 | 2836238 | G | A | nonsynonymous | NM_001669 | c.C470T | p.S157F |
| 12 | BLOOD | CCDC180 | 9 | 100092968 | 100092968 | - | GAGGAG | inframe | NM_020893 | c.2325_2326insGAGGAG | p.E775delinsEEE |
| 12 | BLOOD | ZNF717 | 3 | 75790880 | 75790880 | A | G | nonsynonymous | NM_001290210 | c.T65C | p.V22A |
| 12 | BLOOD | FGF22 | 19 | 643568 | 643568 | C | T | nonsynonymous | NM_001300812 | c.C454T | p.P152S |
| 130 | BLOOD | SPHK2 | 19 | 49132867 | 49132867 | G | C | nonsynonymous | NM_020126 | c.G1802C | p.G601A |
| 131 | LUNG | CHD3 | 17 | 7796803 | 7796803 | T | C | nonsynonymous | NM_001005273 | c.T709C | p.S237P |
| 131 | LUNG | ZNF497 | 19 | 58867794 | 58867794 | T | C | nonsynonymous | NM_198458 | c.A1208G | p.H403R |
| 132 | LUNG | FAM90A1 | 12 | 8374781 | 8374781 | - | ACG | inframe | NM_018088 | c.1031_1032insCGT | p.T344delinsTV |
| 132 | LUNG | HDGFRP2 | 19 | 4499633 | 4499647 | AGCTGGCCGGGGAGG | - | frameshift | NM_001001520 | c.1721_1722del | p.K574fs |
| 132 | LUNG | TXLNA | 1 | 32658026 | 32658026 | G | T | nonsynonymous | NM_175852 | c.G1078T | p.D360Y |
| 132 | LUNG | IL17RC | 3 | 9962283 | 9962283 | C | T | nonsynonymous | NM_153461 | c.C787T | p.P263S |
| 132 | LUNG | PLEC | 8 | 144992767 | 144992767 | C | T | nonsynonymous | NM_201380 | c.G11633A | p.R3878Q |
| 132 | LUNG | ANXA11 | 10 | 81923309 | 81923309 | A | T | nonsynonymous | NM_145869 | c.T1010A | p.L337H |
| 132 | LUNG | TMEM202 | 15 | 72690680 | 72690680 | G | A | nonsynonymous | NM_001080462 | c.G13A | p.E5K |
| 132 | LUNG | PSKH1 | 16 | 67942803 | 67942803 | G | A | nonsynonymous | NM_006742 | c.G151A | p.G51R |
| 133 | LUNG | FBN2 | 5 | 127640728 | 127640728 | C | A | nonsynonymous | NM_001999 | c.G5721T | p.L1907F |
| 133 | LUNG | POLR3B | 12 | 106820975 | 106820975 | C | T | nonsynonymous | NM_001160708 | c.C928T | p.L310F,POLR3B |
| 133 | LUNG | DACT1 | 14 | 59113231 | 59113231 | G | T | nonsynonymous | NM_016651 | c.G1890T | p.K630N |
| 133 | LUNG | SIRPA | 20 | 1895963 | 1895963 | A | G | nonsynonymous | NM_001040023 | c.A298G | p.N100D |
| 133 | LUNG | PLAC4 | 21 | 42551222 | 42551222 | T | G | nonsynonymous | NM_182832 | c.A334C | p.I112L |
| 134 | LUNG | SMARCA2 | 9 | 2039777 | 2039779 | CAG | - | inframe | NM_001289396 | c.667_669del | p.223_223del |
| 134 | LUNG | PER1 | 17 | 8047048 | 8047048 | T | G | nonsynonymous | NM_002616 | c.A2608C | p.T870P |
| 134 | LUNG | PLAC4 | 21 | 42551245 | 42551245 | A | G | nonsynonymous | NM_182832 | c.T311C | p.L104P |
| 134 | LUNG | PLAC4 | 21 | 42551270 | 42551270 | A | G | nonsynonymous | NM_182832 | c.T286C | p.Y96H |
| 135 | LUNG | SNED1 | 2 | 241992569 | 241992569 | G | T | splicing |  |  |  |
| 135 | LUNG | MUC4 | 3 | 195501162 | 195501162 | G | A | nonsynonymous | NM_004532 | c.C250T | p.P84S |
| 135 | LUNG | MUC4 | 3 | 195505788 | 195505788 | G | C | nonsynonymous | NM_018406 | c.C12663G | p.H4221Q |
| 135 | LUNG | TCEB3C,TCEB3CL | 18 | 44555312 | 44555312 | G | C | nonsynonymous | NM_145653 | c.C902G | p.S301C |
| 136 | LUNG | ATXN1 | 6 | 16327907 | 16327909 | TGA | - | inframe | NM_000332 | c.633_635del | p.211_212del |
| 136 | LUNG | MOG | 6 | 29625033 | 29625035 | TCC | - | inframe | NM_206809 | c.47_49del | p.16_17del |
| 136 | LUNG | FAM120A | 9 | 96259883 | 96259883 | - | A | splicing |  |  |  |
| 136 | LUNG | MCCC1 | 3 | 182755108 | 182755108 | G | A | nonsynonymous | NM_020166 | c.C1492T | p.L498F |
| 136 | LUNG | TRIM24 | 7 | 138252385 | 138252385 | C | A | nonsynonymous | NM_015905 | c.C1690A | p.Q564K |
| 136 | LUNG | ZCCHC14 | 16 | 87448079 | 87448079 | C | G | nonsynonymous | NM_015144 | c.G1133C | p.R378P |
| 136 | LUNG | CCDC144NL | 17 | 20769896 | 20769896 | G | T | nonsynonymous | NM_001004306 | c.C536A | p.T179N |
| 136 | LUNG | CCDC144NL | 17 | 20769899 | 20769899 | G | T | stopgain | NM_001004306 | c.C533A | p.S178X |
| 136 | LUNG | TADA2A | 17 | 35831222 | 35831222 | T | G | nonsynonymous | NM_001166105 | c.T1053G | p.I351M |
| 136 | LUNG | MAPT | 17 | 44071316 | 44071316 | C | T | nonsynonymous | NM_001123066 | c.C1534T | p.P512S |
| 136 | LUNG | ARSD | X | 2835863 | 2835863 | G | T | nonsynonymous | NM_001669 | c.C845A | p.A282D |
| 137 | LUNG | CYP21A2 | 6 | 32006215 | 32006217 | CTG | - | inframe | NM_000500 | c.16_18del | p.6_6del |
| 137 | LUNG | MFSD6 | 2 | 191334519 | 191334519 | G | C | nonsynonymous | NM_017694 | c.G1568C | p.R523P |
| 137 | LUNG | FANCD2 | 3 | 10088394 | 10088394 | C | G | nonsynonymous | NM_001018115 | c.C1265G | p.S422C |
| 137 | LUNG | CYP21A2 | 6 | 32006886 | 32006886 | G | A | nonsynonymous | NM_000500 | c.G308A | p.R103K |
| 137 | LUNG | HLA-DRB5 | 6 | 32487309 | 32487309 | T | C | nonsynonymous | NM_002125 | c.A490G | p.S164G |
| 137 | LUNG | HLA-DQB1 | 6 | 32629935 | 32629935 | C | G | nonsynonymous | NM_001243961 | c.G470C | p.G157A |
| 137 | LUNG | PEX6 | 6 | 42946386 | 42946386 | G | A | nonsynonymous | NM_000287 | c.C503T | p.P168L |
| 137 | LUNG | GSTA5 | 6 | 52702355 | 52702355 | C | T | splicing |  |  |  |
| 137 | LUNG | NPC1L1 | 7 | 44556415 | 44556415 | G | A | nonsynonymous | NM_013389 | c.C3487T | p.R1163C |
| 137 | LUNG | NPC1 | 18 | 21124945 | 21124945 | C | G | nonsynonymous | NM_000271 | c.G1926C | p.M642I |
| 137 | LUNG | CYP4F2 | 19 | 15989730 | 15989730 | T | C | nonsynonymous | NM_001082 | c.A1414G | p.T472A |
| 138 | LUNG | RBMXL1 | 1 | 89449298 | 89449298 | C | G | nonsynonymous | NM_019610 | c.G212C | p.G71A |
| 138 | LUNG | MUC4 | 3 | 195505788 | 195505788 | G | C | nonsynonymous | NM_018406 | c.C12663G | p.H4221Q |
| 138 | LUNG | KRT1 | 12 | 53069236 | 53069256 | TAGCTGCTACCTCCGGAGCCA | - | inframe | NM_006121 | c.1656_1676del | p.552_559del |
| 138 | LUNG | SCARB1 | 12 | 125299542 | 125299542 | C | T | nonsynonymous | NM_005505 | c.G403A | p.V135I |
| 138 | LUNG | PRSS53 | 16 | 31096113 | 31096113 | G | A | nonsynonymous | NM_001039503 | c.C1267T | p.R423C |
| 138 | LUNG | TULP2 | 19 | 49391370 | 49391370 | C | T | nonsynonymous | NM_003323 | c.G785A | p.R262H |
| 138 | LUNG | PPP6R1 | 19 | 55743494 | 55743494 | G | A | nonsynonymous | NM_014931 | c.C2074T | p.R692C |
| 138 | LUNG | ZNF814 | 19 | 58385748 | 58385748 | G | A | nonsynonymous | NM_001144989 | c.C1010T | p.A337V |
| 139 | LUNG | ZNF814 | 19 | 58385546 | 58385546 | G | T | nonsynonymous | NM_001144989 | c.C1212A | p.D404E |
| 13 | BLOOD | ASXL1 | 20 | 31021642 | 31021642 | - | T | frameshift | NM_015338 | c.1642dupT | p.S547fs |
| 13 | BLOOD | UBAP2L | 1 | 154201124 | 154201124 | T | G | nonsynonymous | NM_014847 | c.T202G | p.C68G |
| 13 | BLOOD | CCSAP | 1 | 229478125 | 229478125 | G | C | nonsynonymous | NM_145257 | c.C88G | p.L30V |
| 13 | BLOOD | SCN9A | 2 | 167129356 | 167129356 | C | A | nonsynonymous | NM_002977 | c.G2871T | p.L957F |
| 13 | BLOOD | UNC5B | 10 | 73053604 | 73053604 | C | G | nonsynonymous | NM_170744 | c.C2089G | p.L697V |
| 13 | BLOOD | TRHDE | 12 | 72666739 | 72666739 | C | A | nonsynonymous | NM_013381 | c.C181A | p.L61I |
| 13 | BLOOD | ATXN3 | 14 | 92537379 | 92537379 | T | C | nonsynonymous | NM_001164782 | c.A43G | p.T15A,ATXN3 |
| 140 | BLOOD | POLG | 15 | 89876827 | 89876827 | - | TGC | inframe | NM_001126131 | c.158_159insGCA | p.Q53delinsQQ |
| 140 | BLOOD | MEGF6 | 1 | 3422063 | 3422063 | G | A | nonsynonymous | NM_001409 | c.C1976T | p.P659L |
| 140 | BLOOD | TYSND1 | 10 | 71905741 | 71905741 | C | A | nonsynonymous | NM_173555 | c.G602T | p.R201L |
| 140 | BLOOD | NAT10 | 11 | 34165053 | 34165053 | G | A | nonsynonymous | NM_024662 | c.G2947A | p.A983T |
| 140 | BLOOD | ZNF30 | 19 | 35422772 | 35422772 | G | A | nonsynonymous | NM_194325 | c.G35A | p.G12E |
| 140 | BLOOD | PNKP | 19 | 50370425 | 50370425 | C | G | nonsynonymous | NM_007254 | c.G37C | p.E13Q |
| 140 | BLOOD | MYBPC2 | 19 | 50949163 | 50949163 | G | A | nonsynonymous | NM_004533 | c.G1160A | p.R387Q |
| 140 | BLOOD | SHROOM2 | X | 9914947 | 9914947 | G | C | nonsynonymous | NM_001649 | c.G4821C | p.L1607F |
| 140 | BLOOD | ZCCHC5 | X | 77913569 | 77913569 | G | A | nonsynonymous | NM_152694 | c.C349T | p.P117S |
| 141 | BLOOD | ERICH6 | 3 | 150421591 | 150421593 | TCC | - | inframe | NM_152394 | c.93_95del | p.31_32del |
| 141 | BLOOD | ABCF1 | 6 | 30558477 | 30558477 | - | A | frameshift | NM_001025091 | c.2538dupA | p.X846delinsX |
| 141 | BLOOD | AEBP1 | 7 | 44148573 | 44148576 | TGAG | - | inframe | NM_001129 | c.1016_1018del | p.339_340del |
| 141 | BLOOD | SGK223 | 8 | 8176387 | 8176387 | - | GGGGCG | inframe | NM_001080826 | c.3497_3498insCGCCCC | p.P1166delinsPAP |
| 141 | BLOOD | BAGE2,BAGE3 | 21 | 11029597 | 11029597 | C | - | splicing |  |  |  |
| 141 | BLOOD | ARAP1 | 11 | 72425263 | 72425263 | G | C | nonsynonymous | NM_001040118 | c.C613G | p.P205A |
| 141 | BLOOD | AKAP8 | 19 | 15484742 | 15484742 | G | A | nonsynonymous | NM_005858 | c.C226T | p.H76Y |
| 141 | BLOOD | ARSD | X | 2835863 | 2835863 | G | T | nonsynonymous | NM_001669 | c.C845A | p.A282D |
| 142 | BLOOD | OR10A7 | 12 | 55615307 | 55615307 | C | - | frameshift | NM_001005280 | c.499delC | p.P167fs |
| 142 | BLOOD | SYNC | 1 | 33160741 | 33160741 | C | T | nonsynonymous | NM_030786 | c.G958A | p.E320K |
| 142 | BLOOD | CCDC73 | 11 | 32676392 | 32676392 | T | C | nonsynonymous | NM_001008391 | c.A772G | p.M258V |
| 142 | BLOOD | POLD4 | 11 | 67120515 | 67120515 | G | A | nonsynonymous | NM_021173 | c.C131T | p.A44V |
| 142 | BLOOD | OR10A7 | 12 | 55615305 | 55615305 | T | G | nonsynonymous | NM_001005280 | c.T497G | p.L166R |
| 142 | BLOOD | USP50 | 15 | 50793009 | 50793009 | C | G | nonsynonymous | NM_203494 | c.G962C | p.G321A |
| 143 | BLOOD | CDC27 | 17 | 45219669 | 45219669 | G | A | nonsynonymous | NM_001256 | c.C1304T | p.S435F |
| 144 | BLOOD | ITPR1 | 3 | 4725969 | 4725969 | G | A | nonsynonymous | NM_001168272 | c.G3458A | p.G1153E |
| 145 | BLOOD | FAM90A1 | 12 | 8374781 | 8374781 | - | ACG | inframe | NM_018088 | c.1031_1032insCGT | p.T344delinsTV |
| 145 | BLOOD | HLA-DRB5 | 6 | 32489708 | 32489708 | C | A | nonsynonymous | NM_002125 | c.G344T | p.G115V |
| 145 | BLOOD | NCOR1 | 17 | 16097870 | 16097870 | C | A | nonsynonymous | NM_006311 | c.G14T | p.G5V |
| 145 | BLOOD | RBMX | X | 135957700 | 135957700 | T | C | nonsynonymous | NM_002139 | c.A586G | p.R196G |
| 146 | BLOOD | CUX2 | 12 | 111758031 | 111758031 | G | A | nonsynonymous | NM_015267 | c.G2218A | p.G740R |
| 146 | BLOOD | MADCAM1 | 19 | 501786 | 501786 | C | A | nonsynonymous | NM_130760 | c.C785A | p.P262Q |
| 146 | BLOOD | CDH22 | 20 | 44839037 | 44839037 | C | T | nonsynonymous | NM_021248 | c.G1195A | p.G399S |
| 146 | BLOOD | ARSD | X | 2836184 | 2836184 | C | T | nonsynonymous | NM_001669 | c.G524A | p.G175D |
| 147 | BLOOD | SFRP1 | 8 | 41166638 | 41166640 | GCT | - | inframe | NM_003012 | c.39_41del | p.13_14del |
| 147 | BLOOD | BAIAP2L2 | 22 | 38483155 | 38483155 | - | TCATGGGTG | inframe | NM_025045 | c.1234_1235insCACCCATGA | p.N412delinsTPMN |
| 147 | BLOOD | MUC20 | 3 | 195447886 | 195447886 | G | C | nonsynonymous | NM_001282506 | c.G8C | p.C3S |
| 148 | BLOOD | RAD17 | 5 | 68692375 | 68692375 | - | A | splicing |  |  |  |
| 149 | BLOOD | TNK1 | 17 | 7287832 | 7287832 | C | A | nonsynonymous | NM_001251902 | c.C896A | p.A299D |
| 149 | BLOOD | EVPL | 17 | 74019710 | 74019710 | T | C | nonsynonymous | NM_001988 | c.A224G | p.Q75R |
| 14 | BLOOD | OBSCN | 1 | 228503785 | 228503785 | G | T | nonsynonymous | NM_001098623 | c.G13250T | p.G4417V |
| 14 | BLOOD | ZNF517 | 8 | 146033767 | 146033767 | G | C | nonsynonymous | NM_213605 | c.G1466C | p.C489S |
| 14 | BLOOD | TH | 11 | 2188164 | 2188164 | C | A | nonsynonymous | NM_199292 | c.G887T | p.R296L |
| 14 | BLOOD | IGSF9B | 11 | 133805532 | 133805532 | G | A | nonsynonymous | NM_001277285 | c.C947T | p.S316L |
| 14 | BLOOD | ZNF814 | 19 | 58385748 | 58385748 | G | A | nonsynonymous | NM_001144989 | c.C1010T | p.A337V |
| 150 | LUNG | IRF5 | 7 | 128587352 | 128587381 | ACTCTGCAGCCGCCCACTCTGCGGCCGCCT | - | inframe | NM_001098630 | c.502_531del | p.168_177del |
| 150 | LUNG | ASPH | 8 | 62550924 | 62550924 | - | A | splicing |  |  |  |
| 150 | LUNG | KRTAP5-5 | 11 | 1651199 | 1651228 | AGGCTGTGGGGGCTGTGGCTCCGGCTGTGC | - | inframe | NM_001001480 | c.129_158del | p.43_53del |
| 150 | LUNG | BAZ2A | 12 | 56992733 | 56992733 | G | T | nonsynonymous | NM_013449 | c.C5471A | p.P1824Q |
| 150 | LUNG | SULF2 | 20 | 46305296 | 46305296 | C | T | nonsynonymous | NM_018837 | c.G1322A | p.R441H |
| 151 | LUNG | TRMT1L | 1 | 185119609 | 185119609 | C | A | nonsynonymous | NM_030934 | c.G362T | p.C121F |
| 151 | LUNG | C2orf73 | 2 | 54587647 | 54587647 | C | T | nonsynonymous | NM_001100396 | c.C812T | p.A271V |
| 151 | LUNG | AKAP13 | 15 | 86287035 | 86287035 | A | C | nonsynonymous | NM_007200 | c.A8371C | p.T2791P |
| 151 | LUNG | ZNF814 | 19 | 58385748 | 58385748 | G | A | nonsynonymous | NM_001144989 | c.C1010T | p.A337V |
| 152 | LUNG | PTCD2 | 5 | 71648562 | 71648563 | AT | - | frameshift | NM_024754 | c.923_924del | p.H308fs |
| 152 | LUNG | TNRC6A | 16 | 24788423 | 24788434 | GCAGCCACAGCC | - | inframe | NM_014494 | c.333_344del | p.111_115del |
| 152 | LUNG | RELT | 11 | 73105746 | 73105746 | G | C | nonsynonymous | NM_152222 | c.G1013C | p.R338P |
| 152 | LUNG | SLC35G4 | 18 | 11610382 | 11610382 | C | A | nonsynonymous | NM_001282300 | c.C788A | p.T263N |
| 152 | LUNG | TPM4 | 19 | 16192805 | 16192805 | C | T | nonsynonymous | NM_003290 | c.C215T | p.T72M |
| 152 | LUNG | LRFN3 | 19 | 36435631 | 36435631 | C | T | nonsynonymous | NM_024509 | c.C1597T | p.P533S |
| 152 | LUNG | RIMBP3 | 22 | 20458648 | 20458648 | C | T | nonsynonymous | NM_015672 | c.G2654A | p.R885Q |
| 153 | LUNG | C2orf54 | 2 | 241827906 | 241827906 | C | A | nonsynonymous | NM_001085437 | c.G1054T | p.G352C |
| 154 | LUNG | ASPN | 9 | 95237025 | 95237027 | TCA | - | inframe | NM_017680 | c.153_155del | p.51_52del |
| 154 | LUNG | KRTAP4-1 | 17 | 39340796 | 39340852 | CGGCAGCAGCTGGACATACCACAGCTGGGGTGGCAGGTGGTCTGACAGCAGAGTGGG | - | inframe | NM_033060 | c.246_254del | p.82_85del |
| 154 | LUNG | LRPAP1 | 4 | 3517886 | 3517886 | G | A | nonsynonymous | NM_002337 | c.C791T | p.A264V |
| 154 | LUNG | PCSK7 | 11 | 117079612 | 117079612 | C | T | splicing |  |  |  |
| 155 | LUNG | TCERG1 | 5 | 145838636 | 145838653 | CAGGCCCAGGCCCAGGCC | - | inframe | NM_006706 | c.628_645del | p.210_215del |
| 156 | LUNG | KRTAP5-1 | 11 | 1606121 | 1606150 | CCACAGCCACCCTTGGATCCCCCACAAGAG | - | inframe | NM_001005922 | c.330_359del | p.110_120del |
| 156 | LUNG | CCDC93 | 2 | 118753931 | 118753931 | A | C | nonsynonymous | NM_019044 | c.T371G | p.V124G |
| 156 | LUNG | FBN2 | 5 | 127640728 | 127640728 | C | A | nonsynonymous | NM_001999 | c.G5721T | p.L1907F |
| 157 | LUNG | SMPD1 | 11 | 6411931 | 6411942 | CTGGTGCTGGCG | - | inframe | NM_000543 | c.103_114del | p.35_38del |
| 157 | LUNG | KDM6B | 17 | 7750178 | 7750186 | ACCACCACC | - | inframe | NM_001080424 | c.753_761del | p.251_254del |
| 157 | LUNG | ART5 | 11 | 3660256 | 3660256 | C | A | nonsynonymous | NM_053017 | c.G797T | p.R266M |
| 157 | LUNG | CACNA1G | 17 | 48650006 | 48650006 | C | T | nonsynonymous | NM_018896 | c.C838T | p.R280W |
| 158 | LUNG | RPTN | 1 | 152129066 | 152129101 | TGGTGGGAATCTCTGTCTTGTTTCTCAGACTGACCA | - | inframe | NM_001122965 | c.474_509del | p.158_170del |
| 158 | LUNG | MUC20 | 3 | 195447886 | 195447886 | G | C | nonsynonymous | NM_001282506 | c.G8C | p.C3S |
| 158 | LUNG | KRTAP4-8 | 17 | 39253953 | 39253953 | G | T | nonsynonymous | NM_031960 | c.C384A | p.S128R |
| 158 | LUNG | COL5A3 | 19 | 10077022 | 10077022 | T | G | nonsynonymous | NM_015719 | c.A4750C | p.T1584P |
| 159 | LUNG | ATG3 | 3 | 112253058 | 112253058 | - | A | frameshift | NM_001278712 | c.920dupT | p.L307fs |
| 159 | LUNG | GOLGA2 | 9 | 131020796 | 131020798 | CCT | - | inframe | NM_004486 | c.2144_2146del | p.715_716del |
| 159 | LUNG | ZC3H13 | 13 | 46549659 | 46549659 | - | TTCCCTCTCTCT | inframe | NM_015070 | c.2226_2227insAGAGAGAGGGAA | p.R743delinsRERER |
| 159 | LUNG | RECQL5 | 17 | 73626918 | 73626918 | - | TG | splicing |  |  |  |
| 159 | LUNG | ABI3BP | 3 | 100568897 | 100568897 | G | A | nonsynonymous | NM_015429 | c.C1367T | p.T456M |
| 159 | LUNG | MUC4 | 3 | 195474138 | 195474138 | A | G | nonsynonymous | NM_004532 | c.T3440C | p.L1147P |
| 159 | LUNG | OR51B2 | 11 | 5345524 | 5345524 | A | C | nonsynonymous | NM_033180 | c.T4G | p.W2G |
| 159 | LUNG | DCHS1 | 11 | 6644149 | 6644149 | C | T | nonsynonymous | NM_003737 | c.G8758A | p.V2920M |
| 159 | LUNG | EIF3F | 11 | 8009126 | 8009126 | C | G | nonsynonymous | NM_003754 | c.C227G | p.A76G |
| 159 | LUNG | MTL5 | 11 | 68509835 | 68509835 | A | G | nonsynonymous | NM_004923 | c.T779C | p.V260A |
| 159 | LUNG | TGDS | 13 | 95248348 | 95248348 | C | T | nonsynonymous | NM_014305 | c.G43A | p.G15S |
| 159 | LUNG | MCEMP1 | 19 | 7743830 | 7743830 | A | G | nonsynonymous | NM_174918 | c.A508G | p.K170E |
| 159 | LUNG | RIMBP3 | 22 | 20457855 | 20457855 | T | A | nonsynonymous | NM_015672 | c.A3447T | p.E1149D |
| 159 | LUNG | SEC14L3 | 22 | 30856091 | 30856091 | C | T | nonsynonymous | NM_174975 | c.G1120A | p.A374T |
| 159 | LUNG | P2RY8 | X | 1585079 | 1585079 | C | T | nonsynonymous | NM_178129 | c.G373A | p.V125I |
| 159 | LUNG | HYPM | X | 37850385 | 37850385 | C | T | nonsynonymous | NM_012274 | c.C293T | p.A98V |
| 15 | BLOOD | MUC20 | 3 | 195447901 | 195447901 | C | G | nonsynonymous | NM_001282506 | c.C23G | p.A8G |
| 15 | BLOOD | NELFCD | 20 | 57556400 | 57556400 | G | T | nonsynonymous | NM_198976 | c.G90T | p.W30C |
| 160 | LUNG | TTLL10 | 1 | 1118415 | 1118415 | G | T | nonsynonymous | NM_001130045 | c.G1076T | p.R359L |
| 160 | LUNG | KRTAP4-7 | 17 | 39240795 | 39240795 | A | T | nonsynonymous | NM_033061 | c.A337T | p.S113C |
| 160 | LUNG | ARHGAP27 | 17 | 43507549 | 43507549 | C | A | stopgain | NM_001282290 | c.G97T | p.E33X |
| 161 | LUNG | HCFC1 | X | 153220360 | 153220360 | A | G | nonsynonymous | NM_005334 | c.T3490C | p.S1164P |
| 162 | LUNG | FAM90A1 | 12 | 8374781 | 8374781 | - | ACG | inframe | NM_018088 | c.1031_1032insCGT | p.T344delinsTV |
| 162 | LUNG | PHF21A | 11 | 45986887 | 45986887 | G | T | nonsynonymous | NM_001101802 | c.C972A | p.S324R |
| 162 | LUNG | MADCAM1 | 19 | 501762 | 501762 | A | C | nonsynonymous | NM_130760 | c.A761C | p.Q254P |
| 162 | LUNG | DMKN | 19 | 36002386 | 36002386 | C | T | nonsynonymous | NM_033317 | c.G845A | p.S282N |
| 162 | LUNG | PNKP | 19 | 50370425 | 50370425 | C | G | nonsynonymous | NM_007254 | c.G37C | p.E13Q |
| 162 | LUNG | GGT5 | 22 | 24628911 | 24628911 | C | A | nonsynonymous | NM_004121 | c.G476T | p.W159L |
| 162 | LUNG | RBMX | X | 135958704 | 135958704 | G | C | nonsynonymous | NM_002139 | c.C499G | p.P167A |
| 162 | LUNG | RBMX | X | 135958730 | 135958730 | C | A | nonsynonymous | NM_002139 | c.G473T | p.G158V |
| 162 | LUNG | RBMX | X | 135960119 | 135960119 | C | T | nonsynonymous | NM_002139 | c.G343A | p.G115R |
| 164 | LUNG | COL9A3 | 20 | 61460315 | 61460315 | C | A | nonsynonymous | NM_001853 | c.C995A | p.P332H |
| 165 | LUNG | FAM90A1 | 12 | 8374781 | 8374781 | - | ACG | inframe | NM_018088 | c.1031_1032insCGT | p.T344delinsTV |
| 165 | LUNG | BAIAP2L2 | 22 | 38482353 | 38482394 | TGCGGGAGCGGGACTGGCCATCCCAGTACTCCGAGGGTGCTA | - | inframe | NM_025045 | c.1322_1363del | p.441_455del |
| 165 | LUNG | IGLON5 | 19 | 51831008 | 51831008 | G | T | nonsynonymous | NM_001101372 | c.G790T | p.G264C |
| 166 | LUNG | ADAM17 | 2 | 9695676 | 9695676 | G | C | nonsynonymous | NM_003183 | c.C59G | p.P20R |
| 166 | LUNG | ADAMTS7 | 15 | 79058880 | 79058880 | G | T | nonsynonymous | NM_014272 | c.C3373A | p.L1125M |
| 166 | LUNG | GOLGA6L10 | 15 | 82635194 | 82635194 | T | C | nonsynonymous | NM_001164465 | c.A1505G | p.E502G |
| 166 | LUNG | CDYL2 | 16 | 80642037 | 80642037 | G | T | nonsynonymous | NM_152342 | c.C1314A | p.S438R |
| 166 | LUNG | NCOR1 | 17 | 16097870 | 16097870 | C | A | nonsynonymous | NM_006311 | c.G14T | p.G5V |
| 167 | LUNG | BSN | 3 | 49694290 | 49694290 | G | A | nonsynonymous | NM_003458 | c.G7301A | p.R2434H |
| 167 | LUNG | ITIH3 | 3 | 52841772 | 52841772 | G | A | nonsynonymous | NM_002217 | c.G2271A | p.M757I |
| 167 | LUNG | HERC2 | 15 | 28517492 | 28517492 | C | A | nonsynonymous | NM_004667 | c.G952T | p.D318Y |
| 168 | BLOOD | PDCD6 | 5 | 271858 | 271869 | CCGGCCCTGGGG | - | inframe | NM_013232 | c.23_34del | p.8_12del |
| 168 | BLOOD | PER1 | 17 | 8047060 | 8047060 | T | G | nonsynonymous | NM_002616 | c.A2596C | p.T866P |
| 168 | BLOOD | TNFRSF6B | 20 | 62328263 | 62328263 | T | G | nonsynonymous | NM_003823 | c.T143G | p.V48G |
| 168 | BLOOD | ZXDB | X | 57618870 | 57618870 | G | A | nonsynonymous | NM_007157 | c.G389A | p.G130D |
| 169 | BLOOD | WDR66 | 12 | 122359397 | 122359397 | - | GAGGAGGAGGAGAAA | inframe | NM_144668 | c.186_187insGAGGAGGAGGAGAAA | p.G62delinsGEEEEK |
| 169 | BLOOD | VCX2 | X | 8138165 | 8138168 | CCTC | - | frameshift | NM_016378 | c.325_328del | p.E109fs |
| 169 | BLOOD | GOLGA6L2 | 15 | 23690560 | 23690560 | A | C | nonsynonymous | NM_001304388 | c.T110G | p.I37S |
| 169 | BLOOD | GOLGA6L2 | 15 | 23690572 | 23690572 | C | T | nonsynonymous | NM_001304388 | c.G98A | p.R33H |
| 169 | BLOOD | KRTAP4-3 | 17 | 39324124 | 39324124 | G | T | nonsynonymous | NM_033187 | c.C301A | p.R101S |
| 16 | BLOOD | KRT1 | 12 | 53069223 | 53069243 | ACCTCCGGAGCCGTAGCTGCT | - | inframe | NM_006121 | c.1669_1689del | p.557_563del |
| 16 | BLOOD | SELPLG | 12 | 109017651 | 109017680 | GAGTGGTCTGTGCCTCCGTGGGCACTGGTT | - | inframe | NM_003006 | c.404_433del | p.135_145del |
| 16 | BLOOD | HLA-B | 6 | 31324003 | 31324003 | T | A | nonsynonymous | NM_005514 | c.A560T | p.E187V |
| 16 | BLOOD | MUC5B | 11 | 1258240 | 1258240 | C | T | nonsynonymous | NM_002458 | c.C3143T | p.A1048V |
| 16 | BLOOD | ATN1 | 12 | 7045178 | 7045178 | G | A | nonsynonymous | NM_001007026 | c.G748A | p.G250S |
| 16 | BLOOD | MADCAM1 | 19 | 501786 | 501786 | C | A | nonsynonymous | NM_130760 | c.C785A | p.P262Q |
| 170 | BLOOD | TRIM60 | 4 | 165962248 | 165962248 | T | A | nonsynonymous | NM_152620 | c.T1024A | p.S342T |
| 170 | BLOOD | TNXB | 6 | 32024560 | 32024560 | G | A | nonsynonymous | NM_019105 | c.C7946T | p.T2649M |
| 170 | BLOOD | RP1L1 | 8 | 10467605 | 10467605 | C | T | nonsynonymous | NM_178857 | c.G4003A | p.G1335R |
| 170 | BLOOD | MTMR7 | 8 | 17206500 | 17206500 | G | A | stopgain | NM_004686 | c.C559T | p.R187X |
| 170 | BLOOD | SQRDL | 15 | 45968443 | 45968443 | C | G | nonsynonymous | NM_021199 | c.C799G | p.R267G |
| 170 | BLOOD | TP53 | 17 | 7577120 | 7577120 | C | A | nonsynonymous | NM_000546 | c.G818T | p.R273L |
| 170 | BLOOD | ZNF814 | 19 | 58385748 | 58385748 | G | A | nonsynonymous | NM_001144989 | c.C1010T | p.A337V |
| 170 | BLOOD | SUN2 | 22 | 39136283 | 39136283 | C | G | nonsynonymous | NM_001199580 | c.G1345C | p.V449L |
| 171 | BLOOD | RBM5 | 3 | 50155888 | 50155889 | GA | - | frameshift | NM_005778 | c.2447_2448del | p.*816fs |
| 171 | BLOOD | DND1 | 5 | 140052430 | 140052430 | G | T | nonsynonymous | NM_194249 | c.C204A | p.D68E |
| 171 | BLOOD | EDNRB | 13 | 78470614 | 78470614 | G | T | nonsynonymous | NM_003991 | c.C1274A | p.P425Q |
| 171 | BLOOD | GPC6 | 13 | 95050865 | 95050865 | A | G | nonsynonymous | NM_005708 | c.A1435G | p.N479D |
| 171 | BLOOD | MYO16 | 13 | 109793101 | 109793101 | T | C | nonsynonymous | NM_001198950 | c.T4541C | p.L1514P |
| 171 | BLOOD | CASKIN1 | 16 | 2231127 | 2231127 | C | A | nonsynonymous | NM_020764 | c.G2242T | p.G748C |
| 172 | BLOOD | FOXE1 | 9 | 100616701 | 100616706 | GCCGCC | - | inframe | NM_004473 | c.505_510del | p.169_170del |
| 172 | BLOOD | WDR66 | 12 | 122359397 | 122359397 | - | GAGGAGGAGGAGAAA | inframe | NM_144668 | c.186_187insGAGGAGGAGGAGAAA | p.G62delinsGEEEEK |
| 172 | BLOOD | LIMD1 | 3 | 45637765 | 45637765 | A | G | nonsynonymous | NM_014240 | c.A1394G | p.K465R |
| 172 | BLOOD | MUC20 | 3 | 195447886 | 195447886 | G | C | nonsynonymous | NM_001282506 | c.G8C | p.C3S |
| 172 | BLOOD | CES1 | 16 | 55862824 | 55862824 | C | T | nonsynonymous | NM_001025194 | c.G112A | p.V38I |
| 173 | BLOOD | PIK3R4 | 3 | 130447530 | 130447530 | - | A | splicing |  |  |  |
| 173 | BLOOD | TBP | 6 | 170871013 | 170871013 | - | CAG | inframe | NM_003194 | c.189_190insCAG | p.Q63delinsQQ |
| 173 | BLOOD | SMOC1 | 14 | 70420250 | 70420250 | G | A | splicing |  |  |  |
| 174 | BLOOD | HSPBP1 | 19 | 55790886 | 55790886 | - | GCCGCCGCC | inframe | NM_001130106 | c.90_91insGGCGGCGGC | p.S31delinsGGGS |
| 174 | BLOOD | ADM2 | 22 | 50921149 | 50921166 | ACACTCGGGCCCCCGAAG | - | inframe | NM_001253845 | c.264_281del | p.88_94del |
| 174 | BLOOD | SPRR2G | 1 | 153122459 | 153122459 | G | A | nonsynonymous | NM_001014291 | c.C128T | p.P43L |
| 174 | BLOOD | ASPM | 1 | 197073475 | 197073475 | C | G | nonsynonymous | NM_018136 | c.G4906C | p.V1636L |
| 175 | BLOOD | KRTAP4-7 | 17 | 39240810 | 39240810 | T | A | nonsynonymous | NM_033061 | c.T352A | p.C118S |
| 175 | BLOOD | MADCAM1 | 19 | 501767 | 501767 | C | T | nonsynonymous | NM_130760 | c.C766T | p.P256S |
| 175 | BLOOD | GDAP1L1 | 20 | 42887221 | 42887221 | C | A | nonsynonymous | NM_024034 | c.C521A | p.P174H |
| 175 | BLOOD | PLAC4 | 21 | 42551425 | 42551425 | C | G | nonsynonymous | NM_182832 | c.G131C | p.R44P |
| 176 | LUNG | DDX11 | 12 | 31237978 | 31237978 | C | T | nonsynonymous | NM_001257144 | c.C556T | p.R186W |
| 176 | LUNG | RAVER1 | 19 | 10431396 | 10431396 | G | A | nonsynonymous | NM_133452 | c.C1756T | p.L586F |
| 177 | LUNG | TCF20 | 22 | 42611145 | 42611150 | CCACTG | - | inframe | NM_005650 | c.162_167del | p.54_56del |
| 177 | LUNG | DNMT3A | 2 | 25462012 | 25462012 | G | A | nonsynonymous | NM_175629 | c.C2395T | p.P799S |
| 177 | LUNG | CCDC66 | 3 | 56655621 | 56655621 | A | G | nonsynonymous | NM_001141947 | c.A2822G | p.E941G |
| 177 | LUNG | SLC7A2 | 8 | 17417877 | 17417877 | C | A | nonsynonymous | NM_001008539 | c.C1339A | p.P447T |
| 177 | LUNG | HMBOX1 | 8 | 28908626 | 28908626 | G | A | nonsynonymous | NM_024567 | c.G1217A | p.R406Q |
| 177 | LUNG | CBWD6 | 9 | 69238234 | 69238234 | G | T | nonsynonymous | NM_001085457 | c.C658A | p.L220I |
| 177 | LUNG | FANCC | 9 | 98011497 | 98011497 | G | A | nonsynonymous | NM_000136 | c.C77T | p.S26F |
| 177 | LUNG | ZNF79 | 9 | 130207072 | 130207072 | G | A | nonsynonymous | NM_007135 | c.G1093A | p.A365T |
| 177 | LUNG | ASB6 | 9 | 132400503 | 132400503 | G | A | nonsynonymous | NM_017873 | c.C832T | p.R278C |
| 177 | LUNG | C10orf71 | 10 | 50531716 | 50531716 | C | A | nonsynonymous | NM_001135196 | c.C1126A | p.P376T |
| 177 | LUNG | DLG5 | 10 | 79566664 | 79566664 | C | T | nonsynonymous | NM_004747 | c.G4819A | p.D1607N |
| 177 | LUNG | LRP5 | 11 | 68131324 | 68131324 | C | T | nonsynonymous | NM_002335 | c.C796T | p.R266C |
| 177 | LUNG | SLC35F4 | 14 | 58047996 | 58047996 | C | A | nonsynonymous | NM_001206920 | c.G740T | p.C247F |
| 177 | LUNG | PCSK4 | 19 | 1482438 | 1482438 | G | A | nonsynonymous | NM_017573 | c.C1733T | p.T578M |
| 177 | LUNG | HMG20B | 19 | 3573767 | 3573767 | G | A | nonsynonymous | NM_006339 | c.G116A | p.R39H |
| 177 | LUNG | PLIN4 | 19 | 4511283 | 4511283 | C | T | nonsynonymous | NM_001080400 | c.G2647A | p.A883T |
| 178 | LUNG | TDG | 12 | 104373728 | 104373728 | - | A | frameshift | NM_003211 | c.287dupA | p.E96fs |
| 178 | LUNG | TRIOBP | 22 | 38119755 | 38119757 | CAA | - | inframe | NM_001039141 | c.1192_1194del | p.398_398del |
| 178 | LUNG | ANGPTL7 | 1 | 11249724 | 11249724 | T | A | nonsynonymous | NM_021146 | c.T88A | p.S30T |
| 178 | LUNG | IL17RC | 3 | 9970102 | 9970102 | C | T | nonsynonymous | NM_153461 | c.C1204T | p.R402W |
| 178 | LUNG | TNK2 | 3 | 195595512 | 195595512 | G | A | nonsynonymous | NM_005781 | c.C1612T | p.R538W |
| 178 | LUNG | DBH | 9 | 136501729 | 136501729 | G | A | nonsynonymous | NM_000787 | c.G236A | p.R79Q |
| 178 | LUNG | FDXACB1 | 11 | 111747587 | 111747587 | C | T | nonsynonymous | NM_138378 | c.G478A | p.D160N |
| 178 | LUNG | C11orf1 | 11 | 111753246 | 111753246 | G | A | nonsynonymous | NM_022761 | c.G200A | p.R67H |
| 178 | LUNG | DLAT | 11 | 111896251 | 111896251 | G | C | nonsynonymous | NM_001931 | c.G55C | p.E19Q |
| 178 | LUNG | APOA4 | 11 | 116693871 | 116693871 | C | T | nonsynonymous | NM_000482 | c.G37A | p.V13M |
| 178 | LUNG | MPZL2 | 11 | 118133311 | 118133311 | T | A | nonsynonymous | NM_005797 | c.A278T | p.D93V |
| 178 | LUNG | HCAR3 | 12 | 123200334 | 123200334 | T | C | nonsynonymous | NM_006018 | c.A951G | p.I317M |
| 178 | LUNG | ADGRD1 | 12 | 131620650 | 131620650 | C | T | nonsynonymous | NM_198827 | c.C2336T | p.A779V |
| 178 | LUNG | NOC4L | 12 | 132632512 | 132632512 | G | A | nonsynonymous | NM_024078 | c.G691A | p.V231M |
| 178 | LUNG | PDPR | 16 | 70182390 | 70182390 | T | G | nonsynonymous | NM_017990 | c.T1986G | p.N662K |
| 178 | LUNG | CLEC17A | 19 | 14710890 | 14710890 | A | G | nonsynonymous | NM_001204118 | c.A790G | p.K264E |
| 178 | LUNG | FPR1 | 19 | 52249959 | 52249959 | G | T | nonsynonymous | NM_001193306 | c.C289A | p.L97M |
| 178 | LUNG | IL9R | X | 155239824 | 155239824 | A | G | nonsynonymous | NM_002186 | c.A1316G | p.N439S |
| 178 | LUNG | IL9R | X | 155239827 | 155239827 | A | G | nonsynonymous | NM_002186 | c.A1319G | p.N440S |
| 179 | LUNG | UBA1 | X | 47073983 | 47073983 | G | T | nonsynonymous | NM_003334 | c.G2988T | p.M996I |
| 17 | BLOOD | DDHD1 | 14 | 53619480 | 53619480 | - | GCCGCC | inframe | NM_001160147 | c.336_337insGGCGGC | p.S113delinsGGS |
| 17 | BLOOD | WDR73 | 15 | 85186877 | 85186894 | CTTGGCTCCGTGTTCCAT | - | inframe | NM_032856 | c.944_961del | p.315_321del |
| 180 | LUNG | C15orf40 | 15 | 83677270 | 83677270 | - | A | frameshift | NM_001160113 | c.395dupT | p.L132fs |
| 181 | LUNG | PRB3 | 12 | 11420460 | 11420460 | - | G | frameshift | NM_006249 | c.722dupC | p.P241fs |
| 181 | LUNG | BCL6B | 17 | 6928019 | 6928019 | - | CAG | inframe | NM_181844 | c.701_702insCAG | p.S234delinsSS |
| 181 | LUNG | ANAPC1 | 2 | 112620113 | 112620113 | G | T | stopgain | NM_022662 | c.C1115A | p.S372X |
| 181 | LUNG | TMEM8A | 16 | 426329 | 426329 | A | C | nonsynonymous | NM_021259 | c.T1031G | p.V344G |
| 181 | LUNG | LAMA5 | 20 | 60888452 | 60888452 | T | A | nonsynonymous | NM_005560 | c.A8723T | p.E2908V |
| 182 | LUNG | FAM90A1 | 12 | 8374781 | 8374781 | - | ACG | inframe | NM_018088 | c.1031_1032insCGT | p.T344delinsTV |
| 182 | LUNG | WDR66 | 12 | 122359397 | 122359397 | - | GAGGAGGAGGAGAAA | inframe | NM_144668 | c.186_187insGAGGAGGAGGAGAAA | p.G62delinsGEEEEK |
| 182 | LUNG | DBNL | 7 | 44097827 | 44097827 | G | A | nonsynonymous | NM_001014436 | c.G652A | p.A218T |
| 182 | LUNG | MADCAM1 | 19 | 501762 | 501762 | A | C | nonsynonymous | NM_130760 | c.A761C | p.Q254P |
| 182 | LUNG | MADCAM1 | 19 | 501767 | 501767 | C | T | nonsynonymous | NM_130760 | c.C766T | p.P256S |
| 183 | BLOOD | MUC4 | 3 | 195511273 | 195511273 | G | A | nonsynonymous | NM_018406 | c.C7178T | p.A2393V |
| 184 | BLOOD | MUC3A | 7 | 100550396 | 100550396 | G | C | nonsynonymous | NM_005960 | c.G977C | p.S326T |
| 184 | BLOOD | BMS1 | 10 | 43287985 | 43287985 | A | G | nonsynonymous | NM_014753 | c.A824G | p.K275R |
| 185 | BLOOD | RETSAT | 2 | 85570849 | 85570849 | C | T | nonsynonymous | NM_017750 | c.G1606A | p.G536R |
| 185 | BLOOD | WASF3 | 13 | 27254303 | 27254303 | G | C | nonsynonymous | NM_001291965 | c.G672C | p.Q224H |
| 185 | BLOOD | TPSD1 | 16 | 1306971 | 1306971 | A | G | nonsynonymous | NM_012217 | c.A428G | p.H143R |
| 186 | BLOOD | FAM86B2 | 8 | 12287957 | 12287957 | C | T | nonsynonymous | NM_001137610 | c.G244A | p.E82K |
| 186 | BLOOD | POLB | 8 | 42226805 | 42226805 | C | G | nonsynonymous | NM_002690 | c.C725G | p.P242R |
| 186 | BLOOD | KNDC1 | 10 | 134980918 | 134980918 | C | T | nonsynonymous | NM_152643 | c.C136T | p.R46W |
| 186 | BLOOD | TSC2 | 16 | 2133701 | 2133701 | G | A | nonsynonymous | NM_000548 | c.G3889A | p.A1297T |
| 186 | BLOOD | FASN | 17 | 80043506 | 80043506 | G | A | nonsynonymous | NM_004104 | c.C3974T | p.P1325L |
| 186 | BLOOD | ARSD | X | 2836238 | 2836238 | G | A | nonsynonymous | NM_001669 | c.C470T | p.S157F |
| 187 | LUNG | MINK1 | 17 | 4797910 | 4797910 | G | A | nonsynonymous | NM_015716 | c.G2788A | p.D930N |
| 187 | LUNG | DZANK1 | 20 | 18433273 | 18433273 | T | G | nonsynonymous | NM_001099407 | c.A529C | p.T177P |
| 188 | LUNG | CDCP2 | 1 | 54605318 | 54605318 | - | GG | frameshift | NM_201546 | c.1224_1225insCC | p.M409fs |
| 188 | LUNG | LTF | 3 | 46501284 | 46501284 | - | CTT | inframe | NM_002343 | c.68_69insAAG | p.R23delinsRR |
| 188 | LUNG | DHFRL1 | 3 | 93779907 | 93779907 | - | A | frameshift | NM_001195643 | c.448dupT | p.S150fs |
| 188 | LUNG | OR5H15 | 3 | 97887844 | 97887844 | T | - | frameshift | NM_001005515 | c.301delT | p.F101fs |
| 188 | LUNG | KIAA2018 | 3 | 113376111 | 113376113 | TGC | - | inframe | NM_001009899 | c.4416_4418del | p.1472_1473del |
| 188 | LUNG | TBP | 6 | 170871013 | 170871013 | - | CAG | inframe | NM_003194 | c.189_190insCAG | p.Q63delinsQQ |
| 188 | LUNG | OR13C2 | 9 | 107367393 | 107367396 | GTTA | - | frameshift | NM_001004481 | c.513_516del | p.N171fs |
| 188 | LUNG | OR13C2 | 9 | 107367665 | 107367666 | GC | - | frameshift | NM_001004481 | c.243_244del | p.T81fs |
| 188 | LUNG | HCN2 | 19 | 615947 | 615955 | CCGCCGCCG | - | inframe | NM_001194 | c.2143_2151del | p.715_717del |
| 188 | LUNG | OR7G3 | 19 | 9236698 | 9236698 | - | ATGGT | frameshift | NM_001001958 | c.928_929insACCAT | p.S310fs |
| 188 | LUNG | ZNF772 | 19 | 57988666 | 57988666 | - | GCC | inframe | NM_001024596 | c.11_12insGGC | p.A4delinsAA |
| 188 | LUNG | OR4F5 | 1 | 69761 | 69761 | A | T | nonsynonymous | NM_001005484 | c.A671T | p.D224V |
| 188 | LUNG | UBXN11 | 1 | 26608843 | 26608843 | C | A | nonsynonymous | NM_183008 | c.G1510T | p.G504C |
| 188 | LUNG | TMEM61 | 1 | 55451915 | 55451915 | C | T | nonsynonymous | NM_182532 | c.C161T | p.T54M |
| 188 | LUNG | ITGB3BP | 1 | 63920630 | 63920630 | C | T | nonsynonymous | NM_014288 | c.G264A | p.M88I |
| 188 | LUNG | LRIG2 | 1 | 113657466 | 113657466 | G | A | nonsynonymous | NM_014813 | c.G2498A | p.R833K |
| 188 | LUNG | PDE4DIP | 1 | 144864277 | 144864277 | G | A | stopgain | NM_014644 | c.C5818T | p.R1940X |
| 188 | LUNG | PYCR2 | 1 | 226109338 | 226109338 | T | C | nonsynonymous | NM_013328 | c.A547G | p.M183V |
| 188 | LUNG | IRAK2 | 3 | 10280481 | 10280481 | C | T | nonsynonymous | NM_001570 | c.C1523T | p.T508M |
| 188 | LUNG | TBC1D5 | 3 | 17226601 | 17226601 | C | G | nonsynonymous | NM_014744 | c.G1852C | p.V618L |
| 188 | LUNG | SUSD5 | 3 | 33194757 | 33194757 | T | C | nonsynonymous | NM_015551 | c.A1367G | p.E456G |
| 188 | LUNG | LRRFIP2 | 3 | 37096000 | 37096000 | G | A | nonsynonymous | NM_006309 | c.C1957T | p.R653W |
| 188 | LUNG | MYRIP | 3 | 40251349 | 40251349 | C | T | nonsynonymous | NM_015460 | c.C1670T | p.S557L |
| 188 | LUNG | ZBTB47 | 3 | 42703029 | 42703029 | A | G | nonsynonymous | NM_145166 | c.A1526G | p.N509S |
| 188 | LUNG | DNAH1 | 3 | 52360767 | 52360767 | C | A | nonsynonymous | NM_015512 | c.C598A | p.L200M |
| 188 | LUNG | ITIH1 | 3 | 52825833 | 52825833 | A | G | nonsynonymous | NM_001166434 | c.A2216G | p.H739R |
| 188 | LUNG | LNP1 | 3 | 100174634 | 100174634 | G | A | nonsynonymous | NM_001085451 | c.G401A | p.R134K |
| 188 | LUNG | SLC9C1 | 3 | 111997692 | 111997692 | C | T | nonsynonymous | NM_183061 | c.G202A | p.A68T |
| 188 | LUNG | GPR156 | 3 | 119886517 | 119886517 | G | A | nonsynonymous | NM_153002 | c.C1807T | p.R603W |
| 188 | LUNG | DNAJC13 | 3 | 132175595 | 132175595 | C | T | nonsynonymous | NM_015268 | c.C1268T | p.A423V |
| 188 | LUNG | TRIM42 | 3 | 140407211 | 140407211 | A | T | nonsynonymous | NM_152616 | c.A1687T | p.T563S |
| 188 | LUNG | ATR | 3 | 142232408 | 142232408 | T | C | nonsynonymous | NM_001184 | c.A4576G | p.I1526V |
| 188 | LUNG | YEATS2 | 3 | 183503988 | 183503988 | C | G | nonsynonymous | NM_018023 | c.C2812G | p.L938V |
| 188 | LUNG | SSPO | 7 | 149488591 | 149488591 | G | T | nonsynonymous | NM_198455 | c.G5042T | p.R1681M |
| 188 | LUNG | ZNF705G | 8 | 7218632 | 7218632 | C | T | nonsynonymous | NM_001164457 | c.G139A | p.G47R |
| 188 | LUNG | ANK1 | 8 | 41542098 | 41542098 | C | T | nonsynonymous | NM_020475 | c.G4501A | p.D1501N |
| 188 | LUNG | ADAMTSL1 | 9 | 18680350 | 18680350 | G | C | nonsynonymous | NM_001040272 | c.G1177C | p.G393R |
| 188 | LUNG | CNTNAP3B | 9 | 43822686 | 43822686 | C | A | nonsynonymous | NM_001201380 | c.C1240A | p.R414S |
| 188 | LUNG | TBC1D2 | 9 | 101006405 | 101006405 | T | C | nonsynonymous | NM_001267571 | c.A518G | p.E173G |
| 188 | LUNG | WDR31 | 9 | 116093356 | 116093356 | C | T | nonsynonymous | NM_001012361 | c.G157A | p.A53T |
| 188 | LUNG | MEGF9 | 9 | 123476257 | 123476257 | G | A | nonsynonymous | NM_001080497 | c.C380T | p.P127L |
| 188 | LUNG | PARD3 | 10 | 34400467 | 34400467 | G | A | nonsynonymous | NM_019619 | c.C3701T | p.S1234L |
| 188 | LUNG | PLAU | 10 | 75671356 | 75671356 | G | T | nonsynonymous | NM_002658 | c.G43T | p.V15L |
| 188 | LUNG | LRIT2 | 10 | 85982343 | 85982343 | G | C | nonsynonymous | NM_001017924 | c.C986G | p.A329G |
| 188 | LUNG | POLL | 10 | 103339446 | 103339446 | C | T | nonsynonymous | NM_001174084 | c.G1492A | p.E498K |
| 188 | LUNG | SEC23IP | 10 | 121685696 | 121685696 | C | G | nonsynonymous | NM_007190 | c.C2270G | p.S757C |
| 188 | LUNG | LRRC43 | 12 | 122685163 | 122685163 | A | G | nonsynonymous | NM_001098519 | c.A1576G | p.K526E |
| 188 | LUNG | LRRC43 | 12 | 122685164 | 122685164 | A | G | nonsynonymous | NM_001098519 | c.A1577G | p.K526R |
| 188 | LUNG | DIS3 | 13 | 73346905 | 73346905 | C | T | nonsynonymous | NM_014953 | c.G1312A | p.D438N |
| 188 | LUNG | PIBF1 | 13 | 73401237 | 73401237 | G | A | nonsynonymous | NM_006346 | c.G896A | p.R299Q |
| 188 | LUNG | CLN5 | 13 | 77574606 | 77574606 | C | A | nonsynonymous | NM_006493 | c.C726A | p.N242K |
| 188 | LUNG | ING1 | 13 | 111371966 | 111371966 | C | T | nonsynonymous | NM_005537 | c.C956T | p.P319L |
| 188 | LUNG | TUBGCP3 | 13 | 113201864 | 113201864 | C | T | nonsynonymous | NM_001286279 | c.G1238A | p.R413H |
| 188 | LUNG | RTN4RL1 | 17 | 1840677 | 1840677 | C | T | nonsynonymous | NM_178568 | c.G439A | p.G147S |
| 188 | LUNG | SLC2A4 | 17 | 7187929 | 7187929 | C | T | nonsynonymous | NM_001042 | c.C853T | p.R285W |
| 188 | LUNG | TP53 | 17 | 7577138 | 7577138 | C | T | nonsynonymous | NM_000546 | c.G800A | p.R267Q |
| 188 | LUNG | MYO15A | 17 | 18065973 | 18065973 | A | G | nonsynonymous | NM_016239 | c.A9592G | p.I3198V |
| 188 | LUNG | SMCR8 | 17 | 18220784 | 18220784 | A | G | nonsynonymous | NM_144775 | c.A1681G | p.I561V |
| 188 | LUNG | MAPK7 | 17 | 19284156 | 19284156 | G | A | nonsynonymous | NM_139033 | c.G634A | p.A212T |
| 188 | LUNG | ATAD5 | 17 | 29161517 | 29161517 | G | A | nonsynonymous | NM_024857 | c.G418A | p.D140N |
| 188 | LUNG | HEATR6 | 17 | 58143637 | 58143637 | G | A | nonsynonymous | NM_022070 | c.C1349T | p.T450M |
| 188 | LUNG | SLC9A3R1 | 17 | 72758167 | 72758167 | G | A | nonsynonymous | NM_004252 | c.G458A | p.R153Q |
| 188 | LUNG | METRNL | 17 | 81052130 | 81052130 | A | G | nonsynonymous | NM_001004431 | c.A746G | p.H249R |
| 188 | LUNG | MOB3A | 19 | 2078292 | 2078292 | C | A | nonsynonymous | NM_130807 | c.G268T | p.V90F |
| 188 | LUNG | ZNF57 | 19 | 2917542 | 2917542 | A | G | nonsynonymous | NM_173480 | c.A923G | p.Y308C |
| 188 | LUNG | HDGFRP2 | 19 | 4501243 | 4501243 | G | C | nonsynonymous | NM_001001520 | c.G1830C | p.K610N |
| 188 | LUNG | PLIN4 | 19 | 4511746 | 4511746 | A | T | nonsynonymous | NM_001080400 | c.T2184A | p.N728K |
| 188 | LUNG | ACER1 | 19 | 6307246 | 6307246 | C | T | nonsynonymous | NM_133492 | c.G544A | p.V182I |
| 188 | LUNG | DHPS | 19 | 12792390 | 12792390 | T | C | nonsynonymous | NM_001930 | c.A191G | p.Q64R |
| 188 | LUNG | OR10H4 | 19 | 16060577 | 16060577 | T | C | nonsynonymous | NM_001004465 | c.T760C | p.Y254H |
| 188 | LUNG | TMEM59L | 19 | 18727850 | 18727850 | G | A | nonsynonymous | NM_012109 | c.G602A | p.R201H |
| 188 | LUNG | ZFP30 | 19 | 38126475 | 38126475 | C | T | nonsynonymous | NM_014898 | c.G967A | p.E323K |
| 188 | LUNG | AXL | 19 | 41754430 | 41754430 | G | A | nonsynonymous | NM_021913 | c.G1549A | p.G517S |
| 188 | LUNG | NTN5 | 19 | 49167531 | 49167531 | A | G | nonsynonymous | NM_145807 | c.T839C | p.I280T |
| 188 | LUNG | BIRC8 | 19 | 53793083 | 53793083 | C | T | nonsynonymous | NM_033341 | c.G545A | p.R182H |
| 188 | LUNG | KIR2DL4 | 19 | 55317456 | 55317456 | G | A | nonsynonymous | NM_001080772 | c.G412A | p.A138T |
| 189 | LUNG | WDR73 | 15 | 85186877 | 85186894 | CTTGGCTCCGTGTTCCAT | - | inframe | NM_032856 | c.944_961del | p.315_321del |
| 189 | LUNG | ANKRD36 | 2 | 97877440 | 97877440 | T | C | nonsynonymous | NM_001164315 | c.T3431C | p.M1144T |
| 189 | LUNG | ZNF705B | 8 | 7808211 | 7808211 | A | C | nonsynonymous | NM_001193630 | c.A260C | p.K87T |
| 18 | BLOOD | SLC5A10 | 17 | 18918510 | 18918510 | - | CGGTACGGGGGTGGGGGC | inframe | NM_001042450 | c.1239_1240insCGGTACGGGGGTGGGGGC | p.G413delinsGRYGGGG |
| 18 | BLOOD | OR7E24 | 19 | 9361741 | 9361741 | T | - | frameshift | NM_001079935 | c.22delT | p.F8fs |
| 18 | BLOOD | MADCAM1 | 19 | 501786 | 501786 | C | A | nonsynonymous | NM_130760 | c.C785A | p.P262Q |
| 190 | LUNG | MIR205HG | 1 | 209605637 | 209605648 | AGCAGCAGCAGC | - | inframe | NM_001104548 | c.252_263del | p.84_88del |
| 190 | LUNG | DMKN | 19 | 36002410 | 36002412 | CTG | - | inframe | NM_033317 | c.819_821del | p.273_274del |
| 190 | LUNG | HLA-DRB1 | 6 | 32551970 | 32551970 | T | G | nonsynonymous | NM_002124 | c.A286C | p.I96L |
| 190 | LUNG | MUC5B | 11 | 1253976 | 1253976 | A | G | nonsynonymous | NM_002458 | c.A2041G | p.S681G |
| 190 | LUNG | FCGBP | 19 | 40392347 | 40392347 | C | G | nonsynonymous | NM_003890 | c.G8157C | p.Q2719H |
| 191 | LUNG | SSPO | 7 | 149519007 | 149519007 | G | - | frameshift | NM_198455 | c.12811delG | p.G4271fs |
| 191 | LUNG | SGK223 | 8 | 8234868 | 8234868 | - | GCCGCT | inframe | NM_001080826 | c.1050_1051insAGCGGC | p.A351delinsSGA |
| 191 | LUNG | OR51B5 | 11 | 5364542 | 5364558 | CAGCCCCAGGTCTGTGG | - | frameshift | NM_001005567 | c.197_213del | p.A66fs |
| 191 | LUNG | CPSF3L | 1 | 1247736 | 1247736 | C | T | nonsynonymous | NM_017871 | c.G1477A | p.V493M |
| 191 | LUNG | AQP12B | 2 | 241622080 | 241622080 | C | T | nonsynonymous | NM_001102467 | c.G175A | p.E59K |
| 191 | LUNG | SNED1 | 2 | 242021197 | 242021197 | G | A | nonsynonymous | NM_001080437 | c.G4022A | p.R1341H |
| 191 | LUNG | ZKSCAN7 | 3 | 44598757 | 44598757 | G | T | nonsynonymous | NM_001288590 | c.G218T | p.S73I |
| 191 | LUNG | CASP8AP2 | 6 | 90578741 | 90578741 | C | T | nonsynonymous | NM_001137667 | c.C5732T | p.T1911I,CASP8AP2 |
| 191 | LUNG | KCNU1 | 8 | 36698038 | 36698038 | C | A | nonsynonymous | NM_001031836 | c.C1576A | p.L526M |
| 191 | LUNG | HRCT1 | 9 | 35906583 | 35906583 | T | A | nonsynonymous | NM_001039792 | c.T299A | p.L100H |
| 191 | LUNG | TJP2 | 9 | 71836104 | 71836104 | G | A | nonsynonymous | NM_004817 | c.G644A | p.R215H |
| 191 | LUNG | SVIL | 10 | 29777637 | 29777637 | C | G | nonsynonymous | NM_021738 | c.G4241C | p.S1414T |
| 191 | LUNG | DNAH9 | 17 | 11648261 | 11648261 | A | G | nonsynonymous | NM_001372 | c.A6259G | p.M2087V |
| 191 | LUNG | ZNF440 | 19 | 11943382 | 11943382 | G | A | nonsynonymous | NM_152357 | c.G1391A | p.G464E |
| 191 | LUNG | ZNF792 | 19 | 35449651 | 35449651 | C | T | nonsynonymous | NM_175872 | c.G1108A | p.D370N |
| 191 | LUNG | ADORA2A | 22 | 24837424 | 24837424 | C | A | nonsynonymous | NM_000675 | c.C1206A | p.D402E |
| 192 | LUNG | MN1 | 22 | 28194934 | 28194936 | TGC | - | inframe | NM_002430 | c.1596_1598del | p.532_533del |
| 193 | BLOOD | WDR66 | 12 | 122359397 | 122359397 | - | GAGGAGGAGGAGAAA | inframe | NM_144668 | c.186_187insGAGGAGGAGGAGAAA | p.G62delinsGEEEEK |
| 193 | BLOOD | MUC4 | 3 | 195507756 | 195507756 | C | G | nonsynonymous | NM_018406 | c.G10695C | p.Q3565H |
| 193 | BLOOD | CGB1 | 19 | 49539560 | 49539560 | T | C | nonsynonymous | NM_033377 | c.A10G | p.R4G |
| 193 | BLOOD | ZNF814 | 19 | 58385546 | 58385546 | G | T | nonsynonymous | NM_001144989 | c.C1212A | p.D404E |
| 193 | BLOOD | ZNF814 | 19 | 58385748 | 58385748 | G | A | nonsynonymous | NM_001144989 | c.C1010T | p.A337V |
| 193 | BLOOD | SHANK3 | 22 | 51160159 | 51160159 | G | T | stopgain | NM_033517 | c.G3856T | p.E1286X |
| 193 | BLOOD | ZXDB | X | 57618870 | 57618870 | G | A | nonsynonymous | NM_007157 | c.G389A | p.G130D |
| 194 | LUNG | RBM47 | 4 | 40440858 | 40440858 | G | A | nonsynonymous | NM_019027 | c.C53T | p.S18F |
| 194 | LUNG | CRHR2 | 7 | 30721794 | 30721794 | C | A | nonsynonymous | NM_001202482 | c.G103T | p.G35C |
| 194 | LUNG | SSPO | 7 | 149484634 | 149484634 | A | C | nonsynonymous | NM_198455 | c.A3557C | p.D1186A |
| 194 | LUNG | KRTAP4-7 | 17 | 39240795 | 39240795 | A | T | nonsynonymous | NM_033061 | c.A337T | p.S113C |
| 195 | LUNG | CNTNAP3B | 9 | 43915893 | 43915893 | G | C | nonsynonymous | NM_001201380 | c.G3741C | p.M1247I |
| 197 | BLOOD | TMEM175 | 4 | 952220 | 952220 | A | C | nonsynonymous | NM_032326 | c.A1451C | p.H484P |
| 197 | BLOOD | CHD3 | 17 | 7796803 | 7796803 | T | C | nonsynonymous | NM_001005273 | c.T709C | p.S237P |
| 197 | BLOOD | FCGBP | 19 | 40440482 | 40440482 | G | T | nonsynonymous | NM_003890 | c.C44A | p.T15N |
| 197 | BLOOD | RANGAP1 | 22 | 41652800 | 41652800 | A | C | nonsynonymous | NM_001278651 | c.T803G | p.V268G |
| 198 | BLOOD | FAM90A1 | 12 | 8374781 | 8374781 | - | ACG | inframe | NM_018088 | c.1031_1032insCGT | p.T344delinsTV |
| 198 | BLOOD | MUC5B | 11 | 1269856 | 1269856 | C | G | nonsynonymous | NM_002458 | c.C11746G | p.P3916A |
| 198 | BLOOD | MUC5B | 11 | 1269860 | 1269860 | C | G | nonsynonymous | NM_002458 | c.C11750G | p.T3917R |
| 198 | BLOOD | HOXC5 | 12 | 54427115 | 54427115 | A | C | nonsynonymous | NM_018953 | c.A209C | p.H70P |
| 199 | BLOOD | SAAL1 | 11 | 18127558 | 18127558 | - | CGG | inframe | NM_138421 | c.30_31insCCG | p.G11delinsPG |
| 199 | BLOOD | ZC3H6 | 2 | 113067501 | 113067501 | G | T | nonsynonymous | NM_198581 | c.G376T | p.G126C |
| 199 | BLOOD | SMPD4 | 2 | 130939152 | 130939152 | G | T | nonsynonymous | NM_017951 | c.C23A | p.A8E |
| 199 | BLOOD | OBSL1 | 2 | 220417300 | 220417300 | G | T | nonsynonymous | NM_015311 | c.C5266A | p.R1756S |
| 199 | BLOOD | EPHB1 | 3 | 134644720 | 134644720 | G | T | nonsynonymous | NM_004441 | c.G121T | p.G41W |
| 199 | BLOOD | PHC3 | 3 | 169866933 | 169866933 | C | A | nonsynonymous | NM_024947 | c.G514T | p.G172W |
| 199 | BLOOD | EVC2 | 4 | 5570334 | 5570334 | C | A | nonsynonymous | NM_147127 | c.G3394T | p.A1132S |
| 199 | BLOOD | RNF39 | 6 | 30039034 | 30039034 | G | C | nonsynonymous | NM_025236 | c.C1117G | p.R373G |
| 199 | BLOOD | MUC21 | 6 | 30955160 | 30955160 | G | A | nonsynonymous | NM_001010909 | c.G1208A | p.S403N |
| 199 | BLOOD | CLPSL1 | 6 | 35754823 | 35754823 | T | C | nonsynonymous | NM_001010886 | c.T148C | p.C50R |
| 199 | BLOOD | ABCA13 | 7 | 48619913 | 48619913 | G | T | nonsynonymous | NM_152701 | c.G14448T | p.W4816C |
| 199 | BLOOD | SMC2 | 9 | 106860779 | 106860779 | A | C | nonsynonymous | NM_001042551 | c.A371C | p.N124T |
| 199 | BLOOD | PSD | 10 | 104175811 | 104175811 | C | A | nonsynonymous | NM_002779 | c.G720T | p.W240C |
| 199 | BLOOD | RCOR2 | 11 | 63683880 | 63683880 | G | T | nonsynonymous | NM_173587 | c.C49A | p.R17S |
| 199 | BLOOD | CNTN5 | 11 | 100061878 | 100061878 | G | T | nonsynonymous | NM_014361 | c.G1601T | p.G534V |
| 199 | BLOOD | TMPRSS5 | 11 | 113561598 | 113561598 | C | A | nonsynonymous | NM_030770 | c.G1028T | p.W343L |
| 199 | BLOOD | GXYLT1 | 12 | 42512844 | 42512844 | A | C | nonsynonymous | NM_173601 | c.T444G | p.H148Q |
| 199 | BLOOD | DHH | 12 | 49483679 | 49483679 | C | T | nonsynonymous | NM_021044 | c.G1154A | p.R385Q |
| 199 | BLOOD | TBX5 | 12 | 114836454 | 114836454 | C | A | nonsynonymous | NM_000192 | c.G434T | p.G145V |
| 199 | BLOOD | NUDT15 | 13 | 48611907 | 48611907 | G | T | nonsynonymous | NM_018283 | c.G25T | p.G9W |
| 199 | BLOOD | INF2 | 14 | 105173339 | 105173339 | G | T | nonsynonymous | NM_022489 | c.G936T | p.W312C |
| 199 | BLOOD | AKT1 | 14 | 105239898 | 105239898 | C | A | nonsynonymous | NM_005163 | c.G722T | p.R241L |
| 199 | BLOOD | APOBR | 16 | 28508910 | 28508910 | G | T | stopgain | NM_018690 | c.G2548T | p.E850X |
| 199 | BLOOD | ARMC5 | 16 | 31478095 | 31478095 | G | T | nonsynonymous | NM_001301820 | c.G2789T | p.R930L |
| 199 | BLOOD | FBXW10 | 17 | 18670143 | 18670143 | G | A | nonsynonymous | NM_001267585 | c.G1672A | p.V558M |
| 199 | BLOOD | IFI35 | 17 | 41165880 | 41165880 | G | T | nonsynonymous | NM_005533 | c.G656T | p.G219V |
| 199 | BLOOD | MAPT | 17 | 44051810 | 44051810 | C | A | nonsynonymous | NM_001123066 | c.C280A | p.H94N |
| 199 | BLOOD | TMC8 | 17 | 76136981 | 76136981 | G | T | nonsynonymous | NM_152468 | c.G1969T | p.D657Y |
| 199 | BLOOD | MAPK4 | 18 | 48248452 | 48248452 | C | A | nonsynonymous | NM_002747 | c.C836A | p.P279H |
| 199 | BLOOD | ABCA7 | 19 | 1055203 | 1055203 | G | A | nonsynonymous | NM_019112 | c.G4058A | p.R1353Q |
| 199 | BLOOD | SLC25A41 | 19 | 6433605 | 6433605 | G | A | stopgain | NM_173637 | c.C100T | p.Q34X |
| 199 | BLOOD | FDX1L | 19 | 10426651 | 10426651 | C | T | nonsynonymous | NM_001031734 | c.G31A | p.G11R |
| 199 | BLOOD | MAN2B1 | 19 | 12767785 | 12767785 | G | T | nonsynonymous | NM_000528 | c.C1505A | p.P502Q |
| 199 | BLOOD | SIGLEC11 | 19 | 50462682 | 50462682 | C | T | nonsynonymous | NM_052884 | c.G992A | p.R331H |
| 199 | BLOOD | EMILIN3 | 20 | 39989972 | 39989972 | C | A | nonsynonymous | NM_052846 | c.G2237T | p.R746L |
| 199 | BLOOD | SHROOM2 | X | 9914947 | 9914947 | G | C | nonsynonymous | NM_001649 | c.G4821C | p.L1607F |
| 199 | BLOOD | ARHGAP6 | X | 11157535 | 11157535 | G | C | nonsynonymous | NM_013427 | c.C2373G | p.D791E |
| 199 | BLOOD | DMD | X | 31893307 | 31893307 | T | G | splicing | NM_013427 | c.C2373G | p.D791E |
| 199 | BLOOD | NRK | X | 105142585 | 105142585 | G | T | stopgain | NM_198465 | c.G589T | p.G197X |
| 19 | BLOOD | AXDND1 | 1 | 179504035 | 179504040 | AAGAAC | - | inframe | NM_144696 | c.2969_2974del | p.990_992del |
| 19 | BLOOD | WDR73 | 15 | 85186877 | 85186894 | CTTGGCTCCGTGTTCCAT | - | inframe | NM_032856 | c.944_961del | p.315_321del |
| 19 | BLOOD | OR7G3 | 19 | 9236698 | 9236698 | - | ATGGT | frameshift | NM_001001958 | c.928_929insACCAT | p.S310fs |
| 19 | BLOOD | NKX3-2 | 4 | 13545849 | 13545849 | C | G | nonsynonymous | NM_001189 | c.G190C | p.G64R |
| 19 | BLOOD | ANKRD18B | 9 | 33572353 | 33572353 | C | T | nonsynonymous | NM_001244752 | c.C3028T | p.L1010F |
| 19 | BLOOD | KRTAP4-6 | 17 | 39296135 | 39296135 | G | A | nonsynonymous | NM_030976 | c.C605T | p.S202F |
| 19 | BLOOD | ARSD | X | 2836238 | 2836238 | G | A | nonsynonymous | NM_001669 | c.C470T | p.S157F |
| 1 | BLOOD | XAB2 | 19 | 7692308 | 7692308 | C | T | nonsynonymous | NM_020196 | c.G343A | p.D115N |
| 1 | BLOOD | PAF1 | 19 | 39881473 | 39881473 | C | A | nonsynonymous | NM_019088 | c.G32T | p.R11L |
| 1 | BLOOD | CGB1 | 19 | 49539560 | 49539560 | T | C | nonsynonymous | NM_033377 | c.A10G | p.R4G |
| 1 | BLOOD | ARSD | X | 2833643 | 2833643 | C | A | nonsynonymous | NM_001669 | c.G954T | p.Q318H |
| 1 | BLOOD | ARSD | X | 2836211 | 2836211 | A | T | nonsynonymous | NM_001669 | c.T497A | p.L166Q |
| 1 | BLOOD | ARSD | X | 2836238 | 2836238 | G | A | nonsynonymous | NM_001669 | c.C470T | p.S157F |
| 200 | BLOOD | MXD4 | 4 | 2263652 | 2263652 | C | A | nonsynonymous | NM_006454 | c.G56T | p.R19M |
| 200 | BLOOD | POLR3B | 12 | 106820975 | 106820975 | C | T | nonsynonymous | NM_001160708 | c.C928T | p.L310F,POLR3B |
| 202 | BLOOD | ITPKB | 1 | 226924876 | 226924884 | CTGCCGCTG | - | inframe | NM_002221 | c.276_284del | p.92_95del |
| 202 | BLOOD | PDE4DIP | 1 | 145039594 | 145039594 | T | C | nonsynonymous | NM_001198832 | c.A16G | p.T6A |
| 202 | BLOOD | UCN2 | 3 | 48600230 | 48600230 | C | T | nonsynonymous | NM_033199 | c.G328A | p.G110S |
| 202 | BLOOD | MUC4 | 3 | 195505859 | 195505859 | T | C | nonsynonymous | NM_018406 | c.A12592G | p.T4198A |
| 202 | BLOOD | GOLGA8M | 15 | 28947425 | 28947425 | G | A | nonsynonymous | NM_001282468 | c.C1658T | p.S553F |
| 202 | BLOOD | GOLGA8M | 15 | 28947426 | 28947426 | A | G | nonsynonymous | NM_001282468 | c.T1657C | p.S553P |
| 203 | LUNG | KRTAP5-5 | 11 | 1651199 | 1651228 | AGGCTGTGGGGGCTGTGGCTCCGGCTGTGC | - | inframe | NM_001001480 | c.129_158del | p.43_53del |
| 203 | LUNG | MED25 | 19 | 50338843 | 50338843 | C | G | nonsynonymous | NM_030973 | c.C1727G | p.A576G |
| 203 | LUNG | KRTAP10-7 | 21 | 46021600 | 46021600 | C | G | nonsynonymous | NM_198689 | c.C1064G | p.S355C |
| 204 | LUNG | KRTAP5-5 | 11 | 1651199 | 1651228 | AGGCTGTGGGGGCTGTGGCTCCGGCTGTGC | - | inframe | NM_001001480 | c.129_158del | p.43_53del |
| 204 | LUNG | MUC4 | 3 | 195511714 | 195511714 | A | G | nonsynonymous | NM_018406 | c.T6737C | p.L2246P |
| 204 | LUNG | RP1L1 | 8 | 10467637 | 10467637 | T | C | nonsynonymous | NM_178857 | c.A3971G | p.E1324G |
| 204 | LUNG | TOR4A | 9 | 140173168 | 140173168 | G | C | nonsynonymous | NM_017723 | c.G27C | p.E9D |
| 204 | LUNG | MGAT2 | 14 | 50089064 | 50089064 | A | G | nonsynonymous | NM_002408 | c.A1078G | p.K360E |
| 204 | LUNG | MADCAM1 | 19 | 501801 | 501801 | A | C | nonsynonymous | NM_130760 | c.A800C | p.K267T |
| 204 | LUNG | MADCAM1 | 19 | 501802 | 501802 | G | C | nonsynonymous | NM_130760 | c.G801C | p.K267N |
| 204 | LUNG | LAMA5 | 20 | 60884427 | 60884427 | C | T | nonsynonymous | NM_005560 | c.G11053A | p.G3685R |
| 204 | LUNG | KCNQ2 | 20 | 62038442 | 62038442 | C | T | nonsynonymous | NM_172107 | c.G2174A | p.R725H |
| 204 | LUNG | ARSD | X | 2836181 | 2836181 | A | T | nonsynonymous | NM_001669 | c.T527A | p.M176K |
| 204 | LUNG | ARSD | X | 2836211 | 2836211 | A | T | nonsynonymous | NM_001669 | c.T497A | p.L166Q |
| 205 | LUNG | OR56A1 | 11 | 6048726 | 6048726 | A | G | nonsynonymous | NM_001001917 | c.T209C | p.L70P |
| 205 | LUNG | OR56A1 | 11 | 6048744 | 6048744 | T | A | nonsynonymous | NM_001001917 | c.A191T | p.Q64L |
| 205 | LUNG | SLC5A10 | 17 | 18923739 | 18923739 | G | C | nonsynonymous | NM_001042450 | c.G1786C | p.A596P |
| 205 | LUNG | ZSCAN30 | 18 | 32844197 | 32844197 | G | C | nonsynonymous | NM_001166012 | c.C120G | p.N40K |
| 206 | LUNG | KRTAP5-5 | 11 | 1651191 | 1651199 | GGCTGTGGA | - | inframe | NM_001001480 | c.121_129del | p.41_43del |
| 206 | LUNG | KRTAP4-5 | 17 | 39305775 | 39305775 | - | GGCAGCAGCTGGGGC | inframe | NM_033188 | c.244_245insGCCCCAGCTGCTGCC | p.Q82delinsRPSCCQ |
| 206 | LUNG | RECQL5 | 17 | 73626918 | 73626918 | - | TG | splicing |  |  |  |
| 206 | LUNG | HDGFRP2 | 19 | 4499633 | 4499647 | AGCTGGCCGGGGAGG | - | frameshift | NM_001001520 | c.1721_1722del | p.K574fs |
| 206 | LUNG | RBMX | X | 135960146 | 135960146 | - | AA | frameshift | NM_002139 | c.315_316insTT | p.P106fs |
| 206 | LUNG | TMPRSS5 | 11 | 113567620 | 113567620 | G | A | nonsynonymous | NM_030770 | c.C538T | p.P180S |
| 206 | LUNG | ROBO4 | 11 | 124761607 | 124761607 | G | A | nonsynonymous | NM_019055 | c.C1639T | p.R547W |
| 206 | LUNG | APLP2 | 11 | 129992369 | 129992369 | G | A | nonsynonymous | NM_001642 | c.G883A | p.G295S |
| 206 | LUNG | SNX19 | 11 | 130750661 | 130750661 | G | A | nonsynonymous | NM_014758 | c.C2614T | p.R872C |
| 206 | LUNG | KRTAP4-9 | 17 | 39261759 | 39261759 | G | A | nonsynonymous | NM_001146041 | c.G119A | p.C40Y |
| 206 | LUNG | KRTAP4-6 | 17 | 39296361 | 39296361 | A | G | nonsynonymous | NM_030976 | c.T379C | p.S127P |
| 206 | LUNG | ZNF225 | 19 | 44619988 | 44619988 | C | T | nonsynonymous | NM_013362 | c.C8T | p.T3M |
| 206 | LUNG | RBMX | X | 135960119 | 135960119 | C | T | nonsynonymous | NM_002139 | c.G343A | p.G115R |
| 207 | BLOOD | ODF1 | 8 | 103573011 | 103573037 | TGCAACCCCTGCAGCCCCTGCAACCCG | - | inframe | NM_024410 | c.652_678del | p.218_226del |
| 207 | BLOOD | ABCA1 | 9 | 107556793 | 107556793 | T | A | splicing |  |  |  |
| 207 | BLOOD | ABCA1 | 9 | 107556793 | 107556793 | - | A | splicing |  |  |  |
| 207 | BLOOD | VCX2 | X | 8138165 | 8138168 | CCTC | - | frameshift | NM_016378 | c.325_328del | p.E109fs |
| 207 | BLOOD | MUC4 | 3 | 195515510 | 195515510 | A | C | nonsynonymous | NM_018406 | c.T2941G | p.Y981D |
| 207 | BLOOD | HLA-B | 6 | 31324003 | 31324003 | T | G | nonsynonymous | NM_005514 | c.A560C | p.E187A |
| 207 | BLOOD | HLA-DRB5 | 6 | 32497970 | 32497970 | T | C | nonsynonymous | NM_002125 | c.A32G | p.Y11C |
| 207 | BLOOD | FAM86B2 | 8 | 12287957 | 12287957 | C | T | nonsynonymous | NM_001137610 | c.G244A | p.E82K |
| 207 | BLOOD | MPDZ | 9 | 13136783 | 13136783 | T | C | nonsynonymous | NM_003829 | c.A4220G | p.Y1407C |
| 207 | BLOOD | VWF | 12 | 6135214 | 6135214 | T | C | splicing |  |  |  |
| 207 | BLOOD | POLG | 15 | 89866657 | 89866657 | C | G | nonsynonymous | NM_001126131 | c.G2243C | p.W748S |
| 207 | BLOOD | HAGH | 16 | 1869987 | 1869987 | C | T | nonsynonymous | NM_005326 | c.G343A | p.V115I |
| 207 | BLOOD | NCOR1 | 17 | 16097870 | 16097870 | C | A | nonsynonymous | NM_006311 | c.G14T | p.G5V |
| 207 | BLOOD | MPRIP | 17 | 17062002 | 17062002 | C | T | nonsynonymous | NM_201274 | c.C1732T | p.P578S |
| 207 | BLOOD | RBMX | X | 135958730 | 135958730 | C | A | nonsynonymous | NM_002139 | c.G473T | p.G158V |
| 207 | BLOOD | RBMX | X | 135960230 | 135960230 | C | T | nonsynonymous | NM_002139 | c.G232A | p.A78T |
| 208 | BLOOD | CNDP1 | 18 | 72223591 | 72223591 | - | TGC | inframe | NM_032649 | c.43_44insTGC | p.V15delinsVL |
| 208 | BLOOD | EPPK1 | 8 | 144940551 | 144940551 | C | T | nonsynonymous | NM_031308 | c.G6871A | p.V2291M |
| 208 | BLOOD | LYZL2 | 10 | 30918597 | 30918597 | T | C | nonsynonymous | NM_183058 | c.A38G | p.K13R |
| 208 | BLOOD | QRICH2 | 17 | 74288410 | 74288410 | C | T | nonsynonymous | NM_032134 | c.G1900A | p.G634S |
| 209 | LUNG | MICA;MICA | 6 | 31380161 | 31380161 | - | CTGCTGCTGCT | frameshift | NM_001177519 | c.952_953insCTGCTGCTGCT | p.G318fs |
| 209 | LUNG | MUC20 | 3 | 195447886 | 195447886 | G | C | nonsynonymous | NM_001282506 | c.G8C | p.C3S |
| 20 | BLOOD | ANKRD36 | 2 | 97877478 | 97877478 | G | A | nonsynonymous | NM_001164315 | c.G3469A | p.V1157M |
| 20 | BLOOD | PABPC1 | 8 | 101719201 | 101719201 | A | G | nonsynonymous | NM_002568 | c.T1361C | p.I454T |
| 20 | BLOOD | FREM2 | 13 | 39262175 | 39262175 | G | A | nonsynonymous | NM_207361 | c.G694A | p.E232K |
| 210 | BLOOD | CCDC144NL | 17 | 20769899 | 20769899 | G | T | stopgain | NM_001004306 | c.C533A | p.S178X |
| 210 | BLOOD | CDC27 | 17 | 45214528 | 45214528 | A | T | nonsynonymous | NM_001256 | c.T1903A | p.Y635N |
| 211 | LUNG | MXD3 | 5 | 176737427 | 176737427 | C | A | nonsynonymous | NM_001142935 | c.G321T | p.Q107H,MXD3 |
| 211 | LUNG | ABCA2 | 9 | 139904436 | 139904436 | C | A | nonsynonymous | NM_001606 | c.G6494T | p.R2165L |
| 211 | LUNG | SLC25A21 | 14 | 37198738 | 37198738 | C | A | stopgain | NM_030631 | c.G304T | p.G102X |
| 211 | LUNG | TBX21 | 17 | 45820022 | 45820022 | A | C | nonsynonymous | NM_013351 | c.A538C | p.T180P |
| 211 | LUNG | CHCHD10 | 22 | 24108345 | 24108345 | C | A | stopgain | NM_213720 | c.G379T | p.E127X |
| 211 | LUNG | ARSD | X | 2836041 | 2836041 | A | T | nonsynonymous | NM_001669 | c.T667A | p.F223I |
| 211 | LUNG | DIAPH2 | X | 96173558 | 96173558 | G | T | nonsynonymous | NM_006729 | c.G920T | p.R307M |
| 212 | LUNG | MSH3 | 5 | 79950710 | 79950736 | CAGCGGCCGCAGCGGCCGCAGCGCCCC | - | inframe | NM_002439 | c.164_190del | p.55_64del |
| 212 | LUNG | FAM90A1 | 12 | 8374781 | 8374781 | - | ACG | inframe | NM_018088 | c.1031_1032insCGT | p.T344delinsTV |
| 212 | LUNG | EPPK1 | 8 | 144946188 | 144946188 | G | A | nonsynonymous | NM_031308 | c.C1234T | p.R412W |
| 212 | LUNG | PHLDB3 | 19 | 43991217 | 43991217 | C | T | nonsynonymous | NM_198850 | c.G1208A | p.S403N |
| 213 | LUNG | ITPKB | 1 | 226924876 | 226924884 | CTGCCGCTG | - | inframe | NM_002221 | c.276_284del | p.92_95del |
| 213 | LUNG | NUTM2F | 9 | 97080945 | 97080947 | AGA | - | inframe | NM_017561 | c.2071_2073del | p.691_691del |
| 213 | LUNG | TEX40 | 11 | 64071272 | 64071272 | G | C | nonsynonymous | NM_001039496 | c.G460C | p.E154Q |
| 213 | LUNG | IL18BP | 11 | 71712875 | 71712875 | T | C | nonsynonymous | NM_001039660 | c.T553C | p.S185P |
| 213 | LUNG | CACNA1H | 16 | 1257797 | 1257797 | G | A | nonsynonymous | NM_021098 | c.G3091A | p.E1031K |
| 213 | LUNG | MADCAM1 | 19 | 501762 | 501762 | A | C | nonsynonymous | NM_130760 | c.A761C | p.Q254P |
| 213 | LUNG | MADCAM1 | 19 | 501786 | 501786 | C | A | nonsynonymous | NM_130760 | c.C785A | p.P262Q |
| 214 | LUNG | CDC27 | 17 | 45249335 | 45249335 | T | G | nonsynonymous | NM_001256 | c.A199C | p.T67P |
| 214 | LUNG | CBX8 | 17 | 77769127 | 77769127 | C | G | nonsynonymous | NM_020649 | c.G477C | p.E159D |
| 214 | LUNG | HCFC1 | X | 153220360 | 153220360 | A | G | nonsynonymous | NM_005334 | c.T3490C | p.S1164P |
| 215 | LUNG | KISS1 | 1 | 204159612 | 204159612 | T | - | stoploss | NM_002256 | c.417delA | p.X139W |
| 215 | LUNG | MAGEF1 | 3 | 184429133 | 184429133 | - | TCC | inframe | NM_022149 | c.476_477insGGA | p.D159delinsED |
| 215 | LUNG | TTC37 | 5 | 94839536 | 94839536 | A | G | nonsynonymous | NM_014639 | c.T3199C | p.Y1067H |
| 215 | LUNG | HERC2 | 15 | 28517492 | 28517492 | C | A | nonsynonymous | NM_004667 | c.G952T | p.D318Y |
| 215 | LUNG | IZUMO4 | 19 | 2097948 | 2097948 | C | T | nonsynonymous | NM_001039846 | c.C391T | p.R131C |
| 215 | LUNG | CGB1 | 19 | 49539560 | 49539560 | T | C | nonsynonymous | NM_033377 | c.A10G | p.R4G |
| 215 | LUNG | TRPM4 | 19 | 49686408 | 49686408 | A | C | nonsynonymous | NM_017636 | c.A1682C | p.D561A |
| 215 | LUNG | SLC6A16 | 19 | 49812952 | 49812952 | G | C | nonsynonymous | NM_014037 | c.C832G | p.L278V |
| 215 | LUNG | ADAMTS5 | 21 | 28338307 | 28338307 | G | T | nonsynonymous | NM_007038 | c.C404A | p.T135K |
| 215 | LUNG | KRTAP13-2 | 21 | 31744440 | 31744440 | G | A | nonsynonymous | NM_181621 | c.C92T | p.P31L |
| 215 | LUNG | KRTAP10-8 | 21 | 46032769 | 46032769 | G | A | nonsynonymous | NM_198695 | c.G752A | p.R251H |
| 215 | LUNG | SFI1 | 22 | 31924818 | 31924818 | C | T | stopgain | NM_001007467 | c.C235T | p.R79X |
| 215 | LUNG | CRLF2 | X | 1314931 | 1314931 | C | T | nonsynonymous | NM_001012288 | c.G394A | p.V132M |
| 216 | LUNG | VAT1 | 17 | 41174211 | 41174219 | GGCGGCGGC | - | inframe | NM_006373 | c.121_129del | p.41_43del |
| 216 | LUNG | MUC4 | 3 | 195505836 | 195505836 | G | C | nonsynonymous | NM_018406 | c.C12615G | p.H4205Q |
| 216 | LUNG | KCNJ12,KCNJ18 | 17 | 21319079 | 21319079 | C | A | nonsynonymous | NM_021012 | c.C425A | p.T142N |
| 217 | LUNG | NAALADL1 | 11 | 64814948 | 64814948 | C | A | nonsynonymous | NM_005468 | c.G1598T | p.R533L |
| 217 | LUNG | TARSL2 | 15 | 102264514 | 102264514 | C | A | nonsynonymous | NM_152334 | c.G77T | p.W26L |
| 218 | LUNG | SLC41A3 | 3 | 125745316 | 125745316 | C | A | nonsynonymous | NM_001008485 | c.G460T | p.A154S |
| 218 | LUNG | MUC4 | 3 | 195511884 | 195511884 | G | C | nonsynonymous | NM_018406 | c.C6567G | p.H2189Q |
| 218 | LUNG | HLA-DRB5 | 6 | 32497986 | 32497986 | G | A | nonsynonymous | NM_002125 | c.C16T | p.L6F |
| 218 | LUNG | FNDC1 | 6 | 159655140 | 159655140 | C | A | nonsynonymous | NM_032532 | c.C3596A | p.S1199Y |
| 218 | LUNG | LZTS2 | 10 | 102766486 | 102766486 | G | T | nonsynonymous | NM_032429 | c.G1571T | p.R524L |
| 218 | LUNG | ADAMTS7 | 15 | 79092728 | 79092728 | C | A | nonsynonymous | NM_014272 | c.G262T | p.G88W |
| 218 | LUNG | SLC38A5 | X | 48321350 | 48321350 | C | A | nonsynonymous | NM_033518 | c.G507T | p.K169N |
| 219 | LUNG | EIF2A | 3 | 150276210 | 150276210 | G | T | nonsynonymous | NM_032025 | c.G134T | p.S45I |
| 219 | LUNG | PLIN1 | 15 | 90216584 | 90216584 | C | A | nonsynonymous | NM_002666 | c.G107T | p.C36F |
| 21 | BLOOD | ARSD | X | 2836238 | 2836238 | G | A | nonsynonymous | NM_001669 | c.C470T | p.S157F |
| 220 | LUNG | POLR3C | 1 | 145610565 | 145610565 | C | T | splicing |  |  |  |
| 220 | LUNG | NEK6 | 9 | 127064291 | 127064291 | C | A | nonsynonymous | NM_014397 | c.C48A | p.N16K |
| 220 | LUNG | POLR3B | 12 | 106820975 | 106820975 | C | T | nonsynonymous | NM_001160708 | c.C928T | p.L310F,POLR3B |
| 221 | LUNG | AQP7 | 9 | 33385852 | 33385852 | C | T | nonsynonymous | NM_001170 | c.G538A | p.G180R |
| 221 | LUNG | MUC5B | 11 | 1253976 | 1253976 | A | G | nonsynonymous | NM_002458 | c.A2041G | p.S681G |
| 222 | LUNG | HS6ST1 | 2 | 129075877 | 129075877 | G | T | nonsynonymous | NM_004807 | c.C261A | p.D87E |
| 222 | LUNG | MUC20 | 3 | 195447886 | 195447886 | G | C | nonsynonymous | NM_001282506 | c.G8C | p.C3S |
| 222 | LUNG | MUC4 | 3 | 195489774 | 195489774 | G | T | nonsynonymous | NM_004532 | c.C1588A | p.L530M |
| 222 | LUNG | FBN3 | 19 | 8212267 | 8212267 | C | A | nonsynonymous | NM_032447 | c.G98T | p.W33L |
| 223 | LUNG | CNTNAP3B | 9 | 43849812 | 43849812 | T | G | nonsynonymous | NM_001201380 | c.T1717G | p.C573G |
| 223 | LUNG | HCFC1 | X | 153220360 | 153220360 | A | G | nonsynonymous | NM_005334 | c.T3490C | p.S1164P |
| 224 | LUNG | BIN1 | 2 | 127819725 | 127819725 | C | A | nonsynonymous | NM_139343 | c.G823T | p.G275W |
| 224 | LUNG | YTHDC2 | 5 | 112849618 | 112849618 | C | A | nonsynonymous | NM_022828 | c.C26A | p.P9Q |
| 224 | LUNG | ZNF219 | 14 | 21561335 | 21561335 | C | A | nonsynonymous | NM_016423 | c.G121T | p.G41W |
| 224 | LUNG | HMG20B | 19 | 3574552 | 3574552 | G | T | stopgain | NM_006339 | c.G319T | p.E107X |
| 224 | LUNG | CDC42EP1 | 22 | 37964562 | 37964562 | C | A | nonsynonymous | NM_152243 | c.C911A | p.P304Q |
| 225 | LUNG | CCNO | 5 | 54529169 | 54529169 | G | C | nonsynonymous | NM_021147 | c.C183G | p.F61L |
| 225 | LUNG | SSPO | 7 | 149488591 | 149488591 | G | T | nonsynonymous | NM_198455 | c.G5042T | p.R1681M |
| 225 | LUNG | MED24 | 17 | 38182916 | 38182916 | G | T | nonsynonymous | NM_014815 | c.C1793A | p.A598D |
| 226 | LUNG | NCOR2 | 12 | 124824721 | 124824721 | - | GCCGCTGCT | inframe | NM_006312 | c.5517_5518insAGCAGCGGC | p.G1840delinsSSGG |
| 227 | BLOOD | ZNF445 | 3 | 44496866 | 44496866 | C | A | nonsynonymous | NM_181489 | c.G176T | p.R59I |
| 227 | BLOOD | GCNT2 | 6 | 10529883 | 10529883 | A | C | nonsynonymous | NM_145649 | c.A739C | p.T247P |
| 227 | BLOOD | NCOR1 | 17 | 16097870 | 16097870 | C | A | nonsynonymous | NM_006311 | c.G14T | p.G5V |
| 227 | BLOOD | PNKP | 19 | 50370425 | 50370425 | C | G | nonsynonymous | NM_007254 | c.G37C | p.E13Q |
| 228 | LUNG | TLN1 | 9 | 35713007 | 35713007 | C | T | nonsynonymous | NM_006289 | c.G3386A | p.R1129Q |
| 228 | LUNG | SLC35G4 | 18 | 11610382 | 11610382 | C | A | nonsynonymous | NM_001282300 | c.C788A | p.T263N |
| 229 | BLOOD | UBXN11 | 1 | 26608855 | 26608855 | C | T | nonsynonymous | NM_183008 | c.G1498A | p.G500S |
| 229 | BLOOD | SSPO | 7 | 149488614 | 149488614 | C | A | nonsynonymous | NM_198455 | c.C5065A | p.P1689T |
| 229 | BLOOD | TBX21 | 17 | 45820022 | 45820022 | A | C | nonsynonymous | NM_013351 | c.A538C | p.T180P |
| 22 | BLOOD | PRG4 | 1 | 186276268 | 186276291 | GAGCCTGCACCCACCACTCCCAAA | - | inframe | NM_005807 | c.1417_1440del | p.473_480del |
| 22 | BLOOD | KRTAP5-1 | 11 | 1606121 | 1606150 | CCACAGCCACCCTTGGATCCCCCACAAGAG | - | inframe | NM_001005922 | c.330_359del | p.110_120del |
| 22 | BLOOD | KRTAP5-5 | 11 | 1651199 | 1651228 | AGGCTGTGGGGGCTGTGGCTCCGGCTGTGC | - | inframe | NM_001001480 | c.129_158del | p.43_53del |
| 230 | BLOOD | MYO7A | 11 | 76895771 | 76895792 | GGAGGCGGGGACACCAGGGCCT | - | frameshift | NM_001127179 | c.3514_3535del | p.G1172fs |
| 230 | BLOOD | OR5H14 | 3 | 97869007 | 97869007 | A | G | nonsynonymous | NM_001005514 | c.A778G | p.M260V |
| 230 | BLOOD | ENOSF1 | 18 | 675397 | 675397 | C | T | nonsynonymous | NM_017512 | c.G1154A | p.C385Y |
| 231 | BLOOD | COL18A1 | 21 | 46923943 | 46923945 | TTC | - | inframe | NM_130444 | c.3947_3949del | p.1316_1317del |
| 231 | BLOOD | IFRD2 | 3 | 50328048 | 50328048 | G | A | nonsynonymous | NM_006764 | c.C313T | p.R105C |
| 231 | BLOOD | MUC4 | 3 | 195505788 | 195505788 | G | C | nonsynonymous | NM_018406 | c.C12663G | p.H4221Q |
| 231 | BLOOD | COL20A1 | 20 | 61959758 | 61959758 | C | T | nonsynonymous | NM_020882 | c.C3689T | p.P1230L |
| 231 | BLOOD | KRTAP10-7 | 21 | 46020721 | 46020721 | G | A | nonsynonymous | NM_198689 | c.G185A | p.R62H |
| 231 | BLOOD | PCNT | 21 | 47811214 | 47811214 | C | T | nonsynonymous | NM_006031 | c.C4139T | p.A1380V |
| 233 | LUNG | CCNA1 | 13 | 37012866 | 37012866 | T | G | nonsynonymous | NM_003914 | c.T755G | p.V252G |
| 233 | LUNG | PLEKHH1 | 14 | 68048877 | 68048877 | T | G | nonsynonymous | NM_020715 | c.T3376G | p.F1126V |
| 234 | LUNG | ODF1 | 8 | 103572989 | 103573015 | CCCCTGCAGCCCCTGCAGCCCCTGCAA | - | inframe | NM_024410 | c.630_656del | p.210_219del |
| 234 | LUNG | TRMT1L | 1 | 185119609 | 185119609 | C | A | nonsynonymous | NM_030934 | c.G362T | p.C121F |
| 234 | LUNG | SNRK | 3 | 43389767 | 43389767 | G | T | nonsynonymous | NM_017719 | c.G2016T | p.L672F |
| 234 | LUNG | HDGFL1 | 6 | 22570103 | 22570103 | G | A | nonsynonymous | NM_138574 | c.G299A | p.G100D |
| 234 | LUNG | PPP1R35 | 7 | 100033270 | 100033270 | C | G | nonsynonymous | NM_145030 | c.G572C | p.R191P |
| 234 | LUNG | TRIM24 | 7 | 138252385 | 138252385 | C | A | nonsynonymous | NM_015905 | c.C1690A | p.Q564K |
| 234 | LUNG | CLIP1 | 12 | 122812697 | 122812697 | C | T | nonsynonymous | NM_002956 | c.G3013A | p.E1005K |
| 234 | LUNG | CASC5 | 15 | 40914465 | 40914465 | C | A | nonsynonymous | NM_170589 | c.C2081A | p.T694K |
| 234 | LUNG | ZCCHC14 | 16 | 87448079 | 87448079 | C | G | nonsynonymous | NM_015144 | c.G1133C | p.R378P |
| 234 | LUNG | VTN | 17 | 26694784 | 26694784 | C | A | nonsynonymous | NM_000638 | c.G1276T | p.V426L |
| 234 | LUNG | OR10H4 | 19 | 16060110 | 16060110 | G | T | nonsynonymous | NM_001004465 | c.G293T | p.C98F |
| 234 | LUNG | ATF4 | 22 | 39918522 | 39918522 | C | T | nonsynonymous | NM_001675 | c.C971T | p.A324V |
| 234 | LUNG | TTC38 | 22 | 46664412 | 46664412 | C | T | nonsynonymous | NM_017931 | c.C35T | p.A12V |
| 235 | BLOOD | AIM1L | 1 | 26671690 | 26671690 | A | G | nonsynonymous | NM_001039775 | c.T1459C | p.S487P |
| 235 | BLOOD | PLAC4 | 21 | 42551222 | 42551222 | T | G | nonsynonymous | NM_182832 | c.A334C | p.I112L |
| 235 | BLOOD | PLAC4 | 21 | 42551245 | 42551245 | A | G | nonsynonymous | NM_182832 | c.T311C | p.L104P |
| 235 | BLOOD | PLAC4 | 21 | 42551270 | 42551270 | A | G | nonsynonymous | NM_182832 | c.T286C | p.Y96H |
| 236 | BLOOD | FAM86C1 | 11 | 71498623 | 71498624 | AG | - | frameshift | NM_018172 | c.41_42del | p.Q14fs |
| 236 | BLOOD | SKA3 | 13 | 21729952 | 21729952 | - | A | splicing |  |  |  |
| 236 | BLOOD | WDR73 | 15 | 85186877 | 85186894 | CTTGGCTCCGTGTTCCAT | - | inframe | NM_032856 | c.944_961del | p.315_321del |
| 236 | BLOOD | ABTB1 | 3 | 127399121 | 127399121 | C | T | nonsynonymous | NM_172027 | c.C1240T | p.R414W |
| 236 | BLOOD | MUC4 | 3 | 195515387 | 195515387 | T | C | nonsynonymous | NM_018406 | c.A3064G | p.T1022A |
| 236 | BLOOD | UPF3A | 13 | 115047496 | 115047496 | G | C | nonsynonymous | NM_023011 | c.G208C | p.V70L,UPF3A |
| 236 | BLOOD | TPSAB1 | 16 | 1291622 | 1291622 | A | G | nonsynonymous | NM_003294 | c.A421G | p.T141A |
| 236 | BLOOD | B4GALT6 | 18 | 29264380 | 29264380 | G | A | nonsynonymous | NM_004775 | c.C10T | p.L4F |
| 236 | BLOOD | BFSP1 | 20 | 17511929 | 17511929 | C | G | nonsynonymous | NM_001195 | c.G46C | p.E16Q |
| 237 | BLOOD | MUC2 | 11 | 1093412 | 1093412 | C | T | nonsynonymous | NM_002457 | c.C5231T | p.T1744M |
| 237 | BLOOD | TRIM49C | 11 | 89774252 | 89774252 | G | A | nonsynonymous | NM_001195234 | c.G893A | p.S298N |
| 238 | BLOOD | NADK | 1 | 1684347 | 1684347 | - | CCT | inframe | NM_023018 | c.1336_1337insAGG | p.G446delinsEG |
| 238 | BLOOD | COL23A1 | 5 | 177689216 | 177689216 | A | G | splicing |  |  |  |
| 238 | BLOOD | CEL | 9 | 135947032 | 135947032 | C | A | nonsynonymous | NM_001807 | c.C2152A | p.P718T |
| 238 | BLOOD | PARP4 | 13 | 25021323 | 25021323 | A | G | nonsynonymous | NM_006437 | c.T3116C | p.I1039T |
| 239 | BLOOD | GLCCI1 | 7 | 8008992 | 8008994 | CCT | - | inframe | NM_138426 | c.11_13del | p.4_5del |
| 239 | BLOOD | GRIK3 | 1 | 37285454 | 37285454 | A | C | nonsynonymous | NM_000831 | c.T1756G | p.F586V |
| 239 | BLOOD | HS6ST1 | 2 | 129076016 | 129076016 | C | G | nonsynonymous | NM_004807 | c.G122C | p.S41T |
| 239 | BLOOD | SMPD1 | 11 | 6411941 | 6411941 | C | T | nonsynonymous | NM_000543 | c.C113T | p.A38V |
| 23 | BLOOD | KRTAP5-1 | 11 | 1606121 | 1606150 | CCACAGCCACCCTTGGATCCCCCACAAGAG | - | inframe | NM_001005922 | c.330_359del | p.110_120del |
| 23 | BLOOD | SHROOM2 | X | 9864187 | 9864187 | C | A | nonsynonymous | NM_001649 | c.C2239A | p.R747S |
| 241 | BLOOD | POLG | 15 | 89876828 | 89876839 | TGCTGCTGCTGC | - | inframe | NM_001126131 | c.147_158del | p.49_53del |
| 241 | BLOOD | NCOA3 | 20 | 46279815 | 46279823 | GCAGCAGCA | - | inframe | NM_001174087 | c.3738_3746del | p.1246_1249del |
| 241 | BLOOD | MED15 | 22 | 20920814 | 20920816 | CAG | - | inframe | NM_001003891 | c.751_753del | p.251_251del |
| 241 | BLOOD | CFHR2 | 1 | 196918614 | 196918614 | C | A | nonsynonymous | NM_005666 | c.C88A | p.H30N |
| 241 | BLOOD | ANKRD2 | 10 | 99338292 | 99338292 | C | T | nonsynonymous | NM_001291218 | c.C725T | p.A242V |
| 241 | BLOOD | MUC2 | 11 | 1093430 | 1093430 | C | A | nonsynonymous | NM_002457 | c.C5249A | p.T1750N |
| 241 | BLOOD | NCOR1 | 17 | 16097870 | 16097870 | C | A | nonsynonymous | NM_006311 | c.G14T | p.G5V |
| 242 | BLOOD | NUTM2F | 9 | 97080945 | 97080947 | AGA | - | inframe | NM_017561 | c.2071_2073del | p.691_691del |
| 242 | BLOOD | CDR2L | 17 | 72999277 | 72999277 | G | T | splicing |  |  |  |
| 243 | BLOOD | LTN1 | 21 | 30339206 | 30339206 | T | - | frameshift | NM_015565 | c.1745delA | p.N582fs |
| 244 | LUNG | OR12D2 | 6 | 29365197 | 29365197 | G | - | frameshift | NM_013936 | c.721delG | p.A241fs |
| 244 | LUNG | UBXN11 | 1 | 26608866 | 26608866 | C | G | nonsynonymous | NM_183008 | c.G1487C | p.G496A |
| 244 | LUNG | PTPN18 | 2 | 131113748 | 131113748 | G | C | nonsynonymous | NM_014369 | c.G68C | p.G23A |
| 244 | LUNG | KLHL6 | 3 | 183226281 | 183226281 | C | T | nonsynonymous | NM_130446 | c.G475A | p.D159N |
| 244 | LUNG | RPS3A | 4 | 152024138 | 152024138 | A | C | nonsynonymous | NM_001006 | c.A470C | p.Q157P |
| 244 | LUNG | VEGFC | 4 | 177713326 | 177713326 | T | A | nonsynonymous | NM_005429 | c.A140T | p.E47V |
| 244 | LUNG | BTNL3 | 5 | 180432813 | 180432813 | G | A | nonsynonymous | NM_197975 | c.G1342A | p.A448T |
| 244 | LUNG | C9orf173 | 9 | 140146901 | 140146901 | G | A | nonsynonymous | NM_001004353 | c.G416A | p.R139H |
| 244 | LUNG | DGKZ | 11 | 46397000 | 46397000 | G | A | nonsynonymous | NM_001105540 | c.G2293A | p.G765R |
| 244 | LUNG | SLC2A3 | 12 | 8074055 | 8074055 | T | C | nonsynonymous | NM_006931 | c.A1445G | p.E482G |
| 244 | LUNG | KMT2D | 12 | 49425443 | 49425443 | G | C | nonsynonymous | NM_003482 | c.C13045G | p.P4349A |
| 244 | LUNG | CLEC14A | 14 | 38724051 | 38724051 | C | T | nonsynonymous | NM_175060 | c.G1177A | p.D393N |
| 244 | LUNG | MYBBP1A | 17 | 4443680 | 4443680 | C | T | nonsynonymous | NM_014520 | c.G3397A | p.D1133N |
| 244 | LUNG | MYBBP1A | 17 | 4448380 | 4448380 | C | T | nonsynonymous | NM_014520 | c.G2251A | p.D751N |
| 244 | LUNG | MFSD6L | 17 | 8701319 | 8701319 | T | G | nonsynonymous | NM_152599 | c.A1120C | p.T374P |
| 244 | LUNG | RCVRN | 17 | 9804399 | 9804399 | T | C | nonsynonymous | NM_002903 | c.A400G | p.T134A |
| 244 | LUNG | GPR179 | 17 | 36499165 | 36499165 | C | G | nonsynonymous | NM_001004334 | c.G508C | p.G170R |
| 244 | LUNG | WNT9B | 17 | 44950086 | 44950086 | G | A | nonsynonymous | NM_003396 | c.G281A | p.R94Q |
| 244 | LUNG | ZACN | 17 | 74076071 | 74076071 | G | A | nonsynonymous | NM_180990 | c.G370A | p.E124K |
| 244 | LUNG | DNAH17 | 17 | 76558052 | 76558052 | A | G | nonsynonymous | NM_173628 | c.T1580C | p.M527T |
| 244 | LUNG | ATP13A1 | 19 | 19763424 | 19763424 | T | C | nonsynonymous | NM_020410 | c.A2206G | p.I736V |
| 244 | LUNG | ZNF737 | 19 | 20728156 | 20728156 | A | G | nonsynonymous | NM_001159293 | c.T853C | p.Y285H |
| 244 | LUNG | BID | 22 | 18218353 | 18218353 | A | G | nonsynonymous | NM_197966 | c.T719C | p.M240T |
| 245 | BLOOD | LOC554223 | 6 | 29760353 | 29760373 | GCGGGCGCCGTGGATGGAGCA | - | inframe | NM_001207043 | c.438_458del | p.146_153del |
| 245 | BLOOD | GBX1 | 7 | 150864425 | 150864425 | G | A | nonsynonymous | NM_001098834 | c.C211T | p.P71S |
| 246 | BLOOD | SRPX | X | 38079976 | 38079978 | GCA | - | inframe | NM_006307 | c.68_70del | p.23_24del |
| 246 | BLOOD | AIM1L | 1 | 26671690 | 26671690 | A | G | nonsynonymous | NM_001039775 | c.T1459C | p.S487P |
| 246 | BLOOD | SH3TC1 | 4 | 8228979 | 8228979 | G | A | nonsynonymous | NM_018986 | c.G1558A | p.D520N |
| 246 | BLOOD | TNRC18 | 7 | 5413826 | 5413826 | T | G | nonsynonymous | NM_001080495 | c.A3089C | p.H1030P |
| 246 | BLOOD | MRVI1 | 11 | 10631309 | 10631309 | G | T | nonsynonymous | NM_130385 | c.C1537A | p.R513S |
| 247 | BLOOD | CTAGE5 | 14 | 39784005 | 39784006 | TA | - | splicing |  |  |  |
| 247 | BLOOD | CDH23 | 10 | 73574856 | 73574856 | G | T | nonsynonymous | NM_022124 | c.G9886T | p.D3296Y |
| 247 | BLOOD | MXRA5 | X | 3228382 | 3228382 | G | A | nonsynonymous | NM_015419 | c.C7862T | p.A2621V |
| 248 | BLOOD | NPIPB5 | 16 | 22545744 | 22545755 | TCCACCCTCAGC | - | inframe | NM_001135865 | c.1440_1451del | p.480_484del |
| 248 | BLOOD | SLC5A10 | 17 | 18918510 | 18918510 | - | CGGTACGGGGGTGGGGGC | inframe | NM_001042450 | c.1239_1240insCGGTACGGGGGTGGGGGC | p.G413delinsGRYGGGG |
| 248 | BLOOD | KCNJ9 | 1 | 160057528 | 160057528 | A | G | nonsynonymous | NM_004983 | c.A1103G | p.E368G |
| 248 | BLOOD | PVRL4 | 1 | 161047311 | 161047311 | A | G | nonsynonymous | NM_030916 | c.T662C | p.L221P |
| 248 | BLOOD | CMYA5 | 5 | 79027572 | 79027572 | C | T | nonsynonymous | NM_153610 | c.C2984T | p.A995V |
| 248 | BLOOD | HK3 | 5 | 176318455 | 176318455 | G | C | nonsynonymous | NM_002115 | c.C193G | p.Q65E |
| 248 | BLOOD | USP17L7 | 8 | 11991249 | 11991249 | A | T | stopgain | NM_001256869 | c.T270A | p.Y90X |
| 248 | BLOOD | SIX4 | 14 | 61190384 | 61190384 | G | A | nonsynonymous | NM_017420 | c.C409T | p.P137S |
| 248 | BLOOD | KRT26 | 17 | 38922898 | 38922898 | C | T | nonsynonymous | NM_181539 | c.G1276A | p.V426I |
| 248 | BLOOD | C21orf2 | 21 | 45750089 | 45750089 | C | T | nonsynonymous | NM_004928 | c.G763A | p.A255T |
| 248 | BLOOD | ARSD | X | 2836181 | 2836181 | A | T | nonsynonymous | NM_001669 | c.T527A | p.M176K |
| 248 | BLOOD | ARSD | X | 2836184 | 2836184 | C | T | nonsynonymous | NM_001669 | c.G524A | p.G175D |
| 248 | BLOOD | RBMX | X | 135960230 | 135960230 | C | T | nonsynonymous | NM_002139 | c.G232A | p.A78T |
| 249 | BLOOD | MCC | 5 | 112824033 | 112824033 | - | GCTGCC | inframe | NM_001085377 | c.78_79insGGCAGC | p.S27delinsGSS |
| 249 | BLOOD | FAM86B2 | 8 | 12287957 | 12287957 | C | T | nonsynonymous | NM_001137610 | c.G244A | p.E82K |
| 249 | BLOOD | AQP7 | 9 | 33386465 | 33386465 | A | G | nonsynonymous | NM_001170 | c.T343C | p.Y115H |
| 249 | BLOOD | ATXN3 | 14 | 92537379 | 92537379 | T | C | nonsynonymous | NM_001164782 | c.A43G | p.T15A,ATXN3 |
| 249 | BLOOD | CHD3 | 17 | 7793971 | 7793971 | C | T | nonsynonymous | NM_001005273 | c.C296T | p.P99L |
| 249 | BLOOD | ZNF446 | 19 | 58991723 | 58991723 | C | T | nonsynonymous | NM_017908 | c.C983T | p.P328L |
| 24 | BLOOD | HLA-A | 6 | 29912108 | 29912108 | G | C | nonsynonymous | NM_002116 | c.G829C | p.E277Q |
| 24 | BLOOD | HOXC9 | 12 | 54394291 | 54394291 | C | A | nonsynonymous | NM_006897 | c.C319A | p.P107T |
| 24 | BLOOD | GLG1 | 16 | 74640671 | 74640671 | C | A | nonsynonymous | NM_012201 | c.G322T | p.G108W |
| 24 | BLOOD | CDC27 | 17 | 45234707 | 45234707 | T | A | nonsynonymous | NM_001256 | c.A519T | p.L173F |
| 24 | BLOOD | WDR18 | 19 | 992086 | 992086 | G | A | nonsynonymous | NM_024100 | c.G1063A | p.G355R |
| 24 | BLOOD | RBFOX2 | 22 | 36424359 | 36424359 | C | A | stopgain | NM_001082578 | c.G115T | p.G39X |
| 250 | BLOOD |  | 7 | 55588822 | 55588822 | C | T | nonsynonymous | NM_001284282 | c.G5A | p.C2Y,VOPP1 |
| 250 | BLOOD | PLEKHG2 | 19 | 39914191 | 39914191 | C | T | nonsynonymous | NM_022835 | c.C2497T | p.R833W |
| 251 | BLOOD | LOC554223 | 6 | 29760353 | 29760373 | GCGGGCGCCGTGGATGGAGCA | - | inframe | NM_001207043 | c.438_458del | p.146_153del |
| 251 | BLOOD | NPC1 | 18 | 21124945 | 21124945 | C | G | nonsynonymous | NM_000271 | c.G1926C | p.M642I |
| 251 | BLOOD | WFDC8 | 20 | 44190751 | 44190751 | T | G | nonsynonymous | NM_181510 | c.A134C | p.K45T |
| 252 | BLOOD | GAK | 4 | 875698 | 875698 | T | C | nonsynonymous | NM_005255 | c.A1658G | p.K553R |
| 252 | BLOOD | DRD4 | 11 | 640109 | 640109 | A | C | nonsynonymous | NM_000797 | c.A860C | p.Q287P |
| 253 | BLOOD | HRCT1 | 9 | 35906348 | 35906350 | CTG | - | inframe | NM_001039792 | c.64_66del | p.22_22del |
| 253 | BLOOD | CLUH | 17 | 2604907 | 2604907 | C | A | nonsynonymous | NM_015229 | c.G630T | p.K210N |
| 253 | BLOOD | CCDC144NL | 17 | 20769954 | 20769954 | T | G | nonsynonymous | NM_001004306 | c.A478C | p.T160P |
| 253 | BLOOD | NFATC1 | 18 | 77208983 | 77208983 | G | A | nonsynonymous | NM_001278673 | c.G172A | p.V58I,NFATC1 |
| 253 | BLOOD | PLIN5 | 19 | 4525051 | 4525051 | G | A | nonsynonymous | NM_001013706 | c.C758T | p.P253L |
| 253 | BLOOD | ARSD | X | 2836238 | 2836238 | G | A | nonsynonymous | NM_001669 | c.C470T | p.S157F |
| 254 | BLOOD | MUC2 | 11 | 1093066 | 1093066 | C | A | nonsynonymous | NM_002457 | c.C4885A | p.P1629T |
| 254 | BLOOD | ALPK3 | 15 | 85411508 | 85411508 | A | T | nonsynonymous | NM_020778 | c.A5545T | p.R1849W |
| 255 | BLOOD | FAM90A1 | 12 | 8374781 | 8374781 | - | ACG | inframe | NM_018088 | c.1031_1032insCGT | p.T344delinsTV |
| 255 | BLOOD | PLA2G4D | 15 | 42360938 | 42360938 | G | A | nonsynonymous | NM_178034 | c.C2432T | p.A811V |
| 255 | BLOOD | TPSAB1 | 16 | 1291175 | 1291175 | G | A | nonsynonymous | NM_003294 | c.G83A | p.R28Q |
| 255 | BLOOD | TPSAB1 | 16 | 1291178 | 1291178 | T | C | nonsynonymous | NM_003294 | c.T86C | p.V29A |
| 256 | BLOOD | PCDH12 | 5 | 141324955 | 141324955 | - | CTGCTGCTG | inframe | NM_016580 | c.3545_3546insCAGCAGCAG | p.R1182delinsSSSR |
| 256 | BLOOD | SPAG1 | 8 | 101206459 | 101206459 | - | GAC | inframe | NM_003114 | c.1059_1060insGAC | p.K353delinsKD |
| 256 | BLOOD | TPM2 | 9 | 35683240 | 35683240 | - | G | splicing |  |  |  |
| 256 | BLOOD | IL17RC | 3 | 9974543 | 9974543 | G | C | splicing |  |  |  |
| 256 | BLOOD | ARAP3 | 5 | 141059855 | 141059855 | C | T | nonsynonymous | NM_022481 | c.G199A | p.E67K |
| 256 | BLOOD | RANBP17 | 5 | 170725801 | 170725801 | T | G | nonsynonymous | NM_022897 | c.T3206G | p.V1069G |
| 256 | BLOOD | THEMIS | 6 | 128134833 | 128134833 | A | G | nonsynonymous | NM_001010923 | c.T953C | p.I318T |
| 256 | BLOOD | MAP3K4 | 6 | 161491684 | 161491684 | G | T | nonsynonymous | NM_005922 | c.G1752T | p.Q584H |
| 256 | BLOOD | CHMP4A | 14 | 24680739 | 24680739 | C | T | nonsynonymous | NM_014169 | c.G370A | p.G124R |
| 256 | BLOOD | ERCC4 | 16 | 14015897 | 14015897 | A | G | nonsynonymous | NM_005236 | c.A217G | p.I73V |
| 256 | BLOOD | MYH4 | 17 | 10358009 | 10358009 | C | G | nonsynonymous | NM_017533 | c.G2554C | p.E852Q |
| 256 | BLOOD | PLIN4 | 19 | 4511283 | 4511283 | C | T | nonsynonymous | NM_001080400 | c.G2647A | p.A883T |
| 258 | BLOOD | KRTAP17-1 | 17 | 39471753 | 39471767 | GCCCCCGCAGCCAGA | - | inframe | NM_031964 | c.136_150del | p.46_50del |
| 258 | BLOOD | HOXB9 | 17 | 46703361 | 46703361 | C | A | stopgain | NM_024017 | c.G271T | p.E91X |
| 258 | BLOOD | ZNF814 | 19 | 58385546 | 58385546 | G | T | nonsynonymous | NM_001144989 | c.C1212A | p.D404E |
| 258 | BLOOD | ZXDB | X | 57618849 | 57618849 | A | C | nonsynonymous | NM_007157 | c.A368C | p.E123A |
| 259 | BLOOD | FAM90A1 | 12 | 8374781 | 8374781 | - | ACG | inframe | NM_018088 | c.1031_1032insCGT | p.T344delinsTV |
| 259 | BLOOD | LRRC8D | 1 | 90398977 | 90398977 | G | A | nonsynonymous | NM_001134479 | c.G350A | p.R117H |
| 259 | BLOOD | GRM2 | 3 | 51743379 | 51743379 | C | T | nonsynonymous | NM_000839 | c.C380T | p.A127V |
| 259 | BLOOD | TP63 | 3 | 189597889 | 189597889 | G | T | nonsynonymous | NM_001114982 | c.G1104T | p.E368D,TP63 |
| 259 | BLOOD | ANO3 | 11 | 26664762 | 26664762 | C | T | nonsynonymous | NM_031418 | c.C2309T | p.A770V |
| 259 | BLOOD | ACSM4 | 12 | 7457081 | 7457081 | T | A | nonsynonymous | NM_001080454 | c.T154A | p.F52I |
| 259 | BLOOD | LRP3 | 19 | 33698369 | 33698369 | G | A | nonsynonymous | NM_002333 | c.G2201A | p.S734N |
| 259 | BLOOD | PHLDB3 | 19 | 43983666 | 43983666 | G | A | nonsynonymous | NM_198850 | c.C1565T | p.P522L |
| 259 | BLOOD | GP6 | 19 | 55526354 | 55526354 | G | A | nonsynonymous | NM_016363 | c.C955T | p.R319W |
| 259 | BLOOD | SNX5 | 20 | 17923047 | 17923047 | T | G | nonsynonymous | NM_001282454 | c.A854C | p.N285T |
| 25 | BLOOD | CACNA1B | 9 | 140773612 | 140773612 | - | A | splicing |  |  |  |
| 25 | BLOOD | SEMA3F | 3 | 50224126 | 50224126 | C | A | nonsynonymous | NM_004186 | c.C1894A | p.Q632K |
| 25 | BLOOD | CADPS2 | 7 | 122303575 | 122303575 | A | C | nonsynonymous | NM_017954 | c.T502G | p.C168G |
| 260 | BLOOD | KCNN2 | 5 | 113698631 | 113698631 | - | GCC | inframe | NM_021614 | c.159_160insGCC | p.A53delinsAA |
| 260 | BLOOD | TTN | 2 | 179506963 | 179506963 | C | T | splicing |  |  |  |
| 260 | BLOOD | JPH2 | 20 | 42744717 | 42744717 | C | T | nonsynonymous | NM_020433 | c.G1598A | p.R533H |
| 261 | BLOOD | CTAGE5 | 14 | 39784005 | 39784006 | TA | - | splicing |  |  |  |
| 263 | BLOOD | NUTM2F | 9 | 97080945 | 97080947 | AGA | - | inframe | NM_017561 | c.2071_2073del | p.691_691del |
| 263 | BLOOD | UBTFL1 | 11 | 89819885 | 89819885 | A | T | nonsynonymous | NM_001143975 | c.A768T | p.R256S |
| 263 | BLOOD | C17orf97 | 17 | 263442 | 263442 | G | A | nonsynonymous | NM_001013672 | c.G808A | p.E270K |
| 263 | BLOOD | ARSD | X | 2836181 | 2836181 | A | T | nonsynonymous | NM_001669 | c.T527A | p.M176K |
| 263 | BLOOD | ARSD | X | 2836184 | 2836184 | C | T | nonsynonymous | NM_001669 | c.G524A | p.G175D |
| 263 | BLOOD | ARSD | X | 2836211 | 2836211 | A | T | nonsynonymous | NM_001669 | c.T497A | p.L166Q |
| 263 | BLOOD | ARSD | X | 2836238 | 2836238 | G | A | nonsynonymous | NM_001669 | c.C470T | p.S157F |
| 263 | BLOOD | RBMX | X | 135958704 | 135958704 | G | C | nonsynonymous | NM_002139 | c.C499G | p.P167A |
| 264 | BLOOD | ZNF598 | 16 | 2049882 | 2049882 | - | TCC | inframe | NM_178167 | c.1667_1668insGGA | p.D556delinsED |
| 264 | BLOOD | DRD4 | 11 | 640109 | 640109 | A | C | nonsynonymous | NM_000797 | c.A860C | p.Q287P |
| 265 | LUNG | FAM90A1 | 12 | 8374781 | 8374781 | - | ACG | inframe | NM_018088 | c.1031_1032insCGT | p.T344delinsTV |
| 265 | LUNG | SEZ6 | 17 | 27283241 | 27283241 | C | G | nonsynonymous | NM_178860 | c.G2888C | p.R963P |
| 267 | BLOOD | ZNF814 | 19 | 58385546 | 58385546 | G | T | nonsynonymous | NM_001144989 | c.C1212A | p.D404E |
| 268 | BLOOD | SALL2 | 14 | 21991555 | 21991578 | CTCCTCTTCCTCCTCCTCAGACAA | - | inframe | NM_005407 | c.2284_2307del | p.762_769del |
| 268 | BLOOD | DAZAP1 | 19 | 1434835 | 1434835 | G | C | nonsynonymous | NM_018959 | c.G1148C | p.G383A |
| 268 | BLOOD | PNKP | 19 | 50370425 | 50370425 | C | G | nonsynonymous | NM_007254 | c.G37C | p.E13Q |
| 269 | BLOOD | FAM90A1 | 12 | 8374781 | 8374781 | - | ACG | inframe | NM_018088 | c.1031_1032insCGT | p.T344delinsTV |
| 269 | BLOOD | BAIAP2L2 | 22 | 38483155 | 38483155 | - | TCATGGGTG | inframe | NM_025045 | c.1234_1235insCACCCATGA | p.N412delinsTPMN |
| 269 | BLOOD | EXO1 | 1 | 242020734 | 242020734 | C | A | nonsynonymous | NM_130398 | c.C493A | p.Q165K |
| 269 | BLOOD | ACVR2A | 2 | 148676144 | 148676144 | A | C | nonsynonymous | NM_001278579 | c.A945C | p.K315N |
| 269 | BLOOD | ZNF706 | 8 | 102213962 | 102213962 | C | G | nonsynonymous | NM_016096 | c.G8C | p.R3P |
| 269 | BLOOD | CLIP1 | 12 | 122812697 | 122812697 | C | T | nonsynonymous | NM_002956 | c.G3013A | p.E1005K |
| 269 | BLOOD | PGF | 14 | 75416199 | 75416199 | A | C | nonsynonymous | NM_002632 | c.T176G | p.V59G |
| 269 | BLOOD | MYLK3 | 16 | 46744689 | 46744689 | C | A | nonsynonymous | NM_182493 | c.G2127T | p.L709F |
| 269 | BLOOD | PDPR | 16 | 70182390 | 70182390 | T | G | nonsynonymous | NM_017990 | c.T1986G | p.N662K |
| 269 | BLOOD | ZNF814 | 19 | 58385546 | 58385546 | G | T | nonsynonymous | NM_001144989 | c.C1212A | p.D404E |
| 269 | BLOOD | NCF4 | 22 | 37260160 | 37260160 | A | C | nonsynonymous | NM_000631 | c.A106C | p.T36P |
| 26 | BLOOD | C1QTNF2 | 5 | 159781895 | 159781895 | G | T | nonsynonymous | NM_031908 | c.C259A | p.Q87K |
| 26 | BLOOD | ARSD | X | 2833605 | 2833605 | C | T | stopgain | NM_001669 | c.G992A | p.W331X |
| 270 | BLOOD | AKAP2,PALM2-AKAP2 | 9 | 112900341 | 112900341 | - | GAAGCT | inframe | NM_001136562 | c.1824_1825insGAAGCT | p.E608delinsEEA |
| 270 | BLOOD | NOP2 | 12 | 6675769 | 6675771 | CTC | - | inframe | NM_001258308 | c.162_164del | p.54_55del |
| 270 | BLOOD | HRCT1 | 9 | 35906607 | 35906607 | G | A | nonsynonymous | NM_001039792 | c.G323A | p.R108H |
| 270 | BLOOD | DENND1A | 9 | 126144399 | 126144399 | G | T | nonsynonymous | NM_020946 | c.C2342A | p.A781D |
| 270 | BLOOD | GSTO2 | 10 | 106037734 | 106037734 | G | T | stopgain | NM_183239 | c.G226T | p.E76X |
| 271 | BLOOD | GGTLC2 | 22 | 22988927 | 22988927 | G | A | nonsynonymous | NM_199127 | c.G112A | p.A38T |
| 271 | BLOOD | GYG2 | X | 2778089 | 2778089 | G | A | nonsynonymous | NM_003918 | c.G913A | p.A305T |
| 272 | BLOOD | KRTAP4-3 | 17 | 39324124 | 39324124 | G | T | nonsynonymous | NM_033187 | c.C301A | p.R101S |
| 273 | BLOOD | FAM86B2 | 8 | 12287957 | 12287957 | C | T | nonsynonymous | NM_001137610 | c.G244A | p.E82K |
| 273 | BLOOD | PRMT8 | 12 | 3649787 | 3649787 | T | C | nonsynonymous | NM_019854 | c.T91C | p.S31P |
| 274 | BLOOD | TCEB3C,TCEB3CL | 18 | 44555312 | 44555312 | G | C | nonsynonymous | NM_145653 | c.C902G | p.S301C |
| 275 | BLOOD | TCEB3C,TCEB3CL | 18 | 44555312 | 44555312 | G | C | nonsynonymous | NM_145653 | c.C902G | p.S301C |
| 276 | BLOOD | PRRC2B | 9 | 134351730 | 134351730 | T | G | nonsynonymous | NM_013318 | c.T4214G | p.L1405R |
| 276 | BLOOD | CHD3 | 17 | 7796794 | 7796794 | A | C | nonsynonymous | NM_001005273 | c.A700C | p.I234L |
| 277 | BLOOD | PNPLA7 | 9 | 140356449 | 140356449 | - | C | frameshift | NM_152286 | c.3614dupG | p.G1205fs |
| 277 | BLOOD | ADM2 | 22 | 50921149 | 50921166 | ACACTCGGGCCCCCGAAG | - | inframe | NM_001253845 | c.264_281del | p.88_94del |
| 277 | BLOOD | MUC4 | 3 | 195505813 | 195505813 | T | C | nonsynonymous | NM_018406 | c.A12638G | p.D4213G |
| 277 | BLOOD | MUC4 | 3 | 195505814 | 195505814 | C | T | nonsynonymous | NM_018406 | c.G12637A | p.D4213N |
| 277 | BLOOD | GOLM1 | 9 | 88648217 | 88648217 | C | T | nonsynonymous | NM_016548 | c.G1109A | p.G370E |
| 277 | BLOOD | FRG2B | 10 | 135440222 | 135440222 | C | T | nonsynonymous | NM_001080998 | c.G25A | p.D9N |
| 277 | BLOOD | NCOR1 | 17 | 16097870 | 16097870 | C | A | nonsynonymous | NM_006311 | c.G14T | p.G5V |
| 277 | BLOOD | KEAP1 | 19 | 10599937 | 10599937 | C | G | nonsynonymous | NM_203500 | c.G1639C | p.V547L |
| 278 | BLOOD | NPEPPS | 17 | 45669428 | 45669428 | - | A | splicing |  |  |  |
| 278 | BLOOD | SIT1 | 9 | 35649916 | 35649916 | C | A | nonsynonymous | NM_014450 | c.G520T | p.A174S |
| 278 | BLOOD | UMODL1 | 21 | 43531264 | 43531264 | C | G | nonsynonymous | NM_173568 | c.C1932G | p.D644E |
| 279 | BLOOD | SH2D5 | 1 | 21054041 | 21054041 | A | C | nonsynonymous | NM_001103161 | c.T143G | p.V48G |
| 27 | BLOOD | TPSD1 | 16 | 1306802 | 1306802 | A | G | nonsynonymous | NM_012217 | c.A259G | p.I87V |
| 27 | BLOOD | SALL3 | 18 | 76752335 | 76752335 | C | G | nonsynonymous | NM_171999 | c.C344G | p.A115G |
| 280 | BLOOD | KIAA0754 | 1 | 39879419 | 39879419 | T | C | nonsynonymous | NM_015038 | c.T3482C | p.V1161A |
| 280 | BLOOD | XRCC4 | 5 | 82406931 | 82406931 | T | C | nonsynonymous | NM_022406 | c.T224C | p.L75S |
| 280 | BLOOD | NCOR1 | 17 | 16097870 | 16097870 | C | A | nonsynonymous | NM_006311 | c.G14T | p.G5V |
| 280 | BLOOD | COIL | 17 | 55038196 | 55038196 | T | C | nonsynonymous | NM_004645 | c.A185G | p.E62G |
| 280 | BLOOD | TCF3 | 19 | 1627422 | 1627422 | T | C | nonsynonymous | NM_003200 | c.A302G | p.K101R |
| 280 | BLOOD | PLIN4 | 19 | 4511746 | 4511746 | A | T | nonsynonymous | NM_001080400 | c.T2184A | p.N728K |
| 280 | BLOOD | SUSD2 | 22 | 24579219 | 24579219 | G | A | nonsynonymous | NM_019601 | c.G271A | p.A91T |
| 281 | BLOOD | NCOR1 | 17 | 16097870 | 16097870 | C | A | nonsynonymous | NM_006311 | c.G14T | p.G5V |
| 282 | BLOOD | CDC27 | 17 | 45249328 | 45249328 | G | A | nonsynonymous | NM_001256 | c.C206T | p.P69L |
| 283 | BLOOD | OR7E24 | 19 | 9361741 | 9361741 | T | - | frameshift | NM_001079935 | c.22delT | p.F8fs |
| 284 | BLOOD | LOC554223 | 6 | 29760353 | 29760373 | GCGGGCGCCGTGGATGGAGCA | - | inframe | NM_001207043 | c.438_458del | p.146_153del |
| 284 | BLOOD | FOXE1 | 9 | 100616701 | 100616706 | GCCGCC | - | inframe | NM_004473 | c.505_510del | p.169_170del |
| 284 | BLOOD | MAU2 | 19 | 19431690 | 19431704 | GCGGCCCAGGCGGCG | - | inframe | NM_015329 | c.22_36del | p.8_12del |
| 284 | BLOOD | MUC4 | 3 | 195505801 | 195505801 | G | A | nonsynonymous | NM_018406 | c.C12650T | p.A4217V |
| 284 | BLOOD | CCDC144NL | 17 | 20769896 | 20769896 | G | T | nonsynonymous | NM_001004306 | c.C536A | p.T179N |
| 284 | BLOOD | CCDC144NL | 17 | 20769899 | 20769899 | G | T | stopgain | NM_001004306 | c.C533A | p.S178X |
| 284 | BLOOD | UPK1A | 19 | 36168894 | 36168894 | A | G | nonsynonymous | NM_007000 | c.A750G | p.I250M |
| 285 | BLOOD | NOTCH2 | 1 | 120612003 | 120612004 | GG | - | frameshift | NM_024408 | c.17_18del | p.P6fs |
| 285 | BLOOD | WDR73 | 15 | 85186877 | 85186894 | CTTGGCTCCGTGTTCCAT | - | inframe | NM_032856 | c.944_961del | p.315_321del |
| 285 | BLOOD | FNDC1 | 6 | 159653330 | 159653330 | G | A | nonsynonymous | NM_032532 | c.G1786A | p.G596S |
| 285 | BLOOD | PRDM10 | 11 | 129801109 | 129801109 | T | G | nonsynonymous | NM_199437 | c.A1332C | p.E444D |
| 285 | BLOOD | SYCP2 | 20 | 58461802 | 58461802 | T | C | nonsynonymous | NM_014258 | c.A2452G | p.I818V |
| 285 | BLOOD | ARSD | X | 2833605 | 2833605 | C | T | stopgain | NM_001669 | c.G992A | p.W331X |
| 285 | BLOOD | ARSD | X | 2836181 | 2836181 | A | T | nonsynonymous | NM_001669 | c.T527A | p.M176K |
| 285 | BLOOD | ARSD | X | 2836184 | 2836184 | C | T | nonsynonymous | NM_001669 | c.G524A | p.G175D |
| 286 | BLOOD | KCNN2 | 5 | 113698631 | 113698631 | - | GCC | inframe | NM_021614 | c.159_160insGCC | p.A53delinsAA |
| 286 | BLOOD | LTN1 | 21 | 30339206 | 30339206 | T | - | frameshift | NM_015565 | c.1745delA | p.N582fs |
| 286 | BLOOD | RHPN2 | 19 | 33490566 | 33490566 | T | C | nonsynonymous | NM_033103 | c.A1151G | p.Q384R |
| 287 | BLOOD | CCNA1 | 13 | 37012866 | 37012866 | T | G | nonsynonymous | NM_003914 | c.T755G | p.V252G |
| 287 | BLOOD | RIMBP3 | 22 | 20457841 | 20457841 | T | C | nonsynonymous | NM_015672 | c.A3461G | p.Q1154R |
| 288 | BLOOD | CHRNB2 | 1 | 154544375 | 154544375 | - | GCGCCT | inframe | NM_000748 | c.1076_1077insGCGCCT | p.Q359delinsQRL |
| 288 | BLOOD | KRTAP4-7 | 17 | 39240782 | 39240796 | CTGCTGCCGCCCCAG | - | inframe | NM_033061 | c.324_338del | p.108_113del |
| 288 | BLOOD | MADCAM1 | 19 | 501767 | 501767 | C | T | nonsynonymous | NM_130760 | c.C766T | p.P256S |
| 289 | BLOOD | FOXE1 | 9 | 100616701 | 100616706 | GCCGCC | - | inframe | NM_004473 | c.505_510del | p.169_170del |
| 289 | BLOOD | OR6C76 | 12 | 55820959 | 55820959 | A | - | frameshift | NM_001005183 | c.922delA | p.K308fs |
| 289 | BLOOD | TRMT11 | 6 | 126307736 | 126307736 | A | G | nonsynonymous | NM_001031712 | c.A40G | p.M14V |
| 289 | BLOOD | USP17L7 | 8 | 11990617 | 11990617 | C | A | nonsynonymous | NM_001256869 | c.G902T | p.R301L |
| 289 | BLOOD | TMPRSS9 | 19 | 2396536 | 2396536 | G | A | splicing |  |  |  |
| 28 | BLOOD | MAD1L1 | 7 | 2020091 | 2020091 | A | G | nonsynonymous | NM_001013836 | c.T1502C | p.L501P |
| 290 | BLOOD | TRAK1 | 3 | 42251577 | 42251577 | - | GGAGGA | inframe | NM_014965 | c.1889_1890insGGAGGA | p.T630delinsTEE |
| 290 | BLOOD | SELPLG | 12 | 109017672 | 109017672 | G | C | nonsynonymous | NM_003006 | c.C412G | p.P138A |
| 291 | BLOOD | KCNN3 | 1 | 154842244 | 154842244 | A | T | nonsynonymous | NM_002249 | c.T197A | p.L66H |
| 291 | BLOOD | HLA-A | 6 | 29910716 | 29910716 | C | G | nonsynonymous | NM_002116 | c.C256G | p.Q86E |
| 291 | BLOOD | NCOR1 | 17 | 16097870 | 16097870 | C | A | nonsynonymous | NM_006311 | c.G14T | p.G5V |
| 291 | BLOOD | OGFR | 20 | 61443725 | 61443725 | C | T | nonsynonymous | NM_007346 | c.C758T | p.A253V |
| 292 | BLOOD | PABPC1 | 8 | 101719004 | 101719004 | G | A | nonsynonymous | NM_002568 | c.C1477T | p.R493C |
| 292 | BLOOD | C17orf97 | 17 | 260158 | 260158 | G | C | nonsynonymous | NM_001013672 | c.G25C | p.A9P |
| 293 | BLOOD | KRT1 | 12 | 53069223 | 53069243 | ACCTCCGGAGCCGTAGCTGCT | - | inframe | NM_006121 | c.1669_1689del | p.557_563del |
| 293 | BLOOD | CHD3 | 17 | 7796794 | 7796794 | A | C | nonsynonymous | NM_001005273 | c.A700C | p.I234L |
| 293 | BLOOD | CHD3 | 17 | 7796803 | 7796803 | T | C | nonsynonymous | NM_001005273 | c.T709C | p.S237P |
| 293 | BLOOD | ZNF814 | 19 | 58385546 | 58385546 | G | T | nonsynonymous | NM_001144989 | c.C1212A | p.D404E |
| 294 | BLOOD | MUC20 | 3 | 195447886 | 195447886 | G | C | nonsynonymous | NM_001282506 | c.G8C | p.C3S |
| 294 | BLOOD | DND1 | 5 | 140052430 | 140052430 | G | T | nonsynonymous | NM_194249 | c.C204A | p.D68E |
| 294 | BLOOD | MUC16 | 19 | 8982303 | 8982303 | C | T | nonsynonymous | NM_024690 | c.G41972A | p.R13991Q |
| 295 | BLOOD | NCOR2 | 12 | 124887058 | 124887058 | - | GCT | inframe | NM_006312 | c.1531_1532insAGC | p.P511delinsQP |
| 295 | BLOOD | CLEC17A | 19 | 14694175 | 14694175 | - | GGA | inframe | NM_001204118 | c.50_51insGGA | p.M17delinsME |
| 295 | BLOOD | RBMX | X | 135960119 | 135960119 | C | T | nonsynonymous | NM_002139 | c.G343A | p.G115R |
| 296 | BLOOD | ZRANB1 | 10 | 126673560 | 126673560 | - | A | frameshift | NM_017580 | c.2127dupA | p.X709delinsX |
| 296 | BLOOD | NUDT11 | X | 51239296 | 51239309 | TCCTCGAGGCAGCC | - | frameshift | NM_018159 |  |  |
| 296 | BLOOD | GAS8 | 16 | 90106769 | 90106769 | A | G | nonsynonymous | NM_001481 | c.A1073G | p.Q358R |
| 297 | BLOOD | NPEPPS | 17 | 45669428 | 45669428 | - | A | splicing |  |  |  |
| 297 | BLOOD | AP1S3 | 2 | 224642579 | 224642579 | A | C | nonsynonymous | NM_001039569 | c.T11G | p.F4C |
| 297 | BLOOD | OR6B2 | 2 | 240969456 | 240969456 | G | A | nonsynonymous | NM_001005853 | c.C391T | p.R131C |
| 297 | BLOOD | PRR21 | 2 | 240982219 | 240982219 | A | G | nonsynonymous | NM_001080835 | c.T181C | p.S61P |
| 297 | BLOOD | CRYBG3 | 3 | 97595757 | 97595757 | A | G | nonsynonymous | NM_153605 | c.A5719G | p.T1907A |
| 297 | BLOOD | TBC1D23 | 3 | 99998531 | 99998531 | G | C | nonsynonymous | NM_001199198 | c.G92C | p.C31S |
| 297 | BLOOD | CXXC4 | 4 | 105411974 | 105411974 | C | T | nonsynonymous | NM_025212 | c.G986A | p.R329H |
| 297 | BLOOD | VWA7 | 6 | 31740806 | 31740806 | G | A | nonsynonymous | NM_025258 | c.C1012T | p.R338C |
| 297 | BLOOD | KRT1 | 12 | 53069236 | 53069256 | TAGCTGCTACCTCCGGAGCCA | - | inframe | NM_006121 | c.1656_1676del | p.552_559del |
| 297 | BLOOD | RBMX | X | 135960230 | 135960230 | C | T | nonsynonymous | NM_002139 | c.G232A | p.A78T |
| 298 | BLOOD | ASPN | 9 | 95237024 | 95237024 | C | A | nonsynonymous | NM_017680 | c.G156T | p.E52D |
| 298 | BLOOD | ASPN | 9 | 95237025 | 95237027 | TCA | - | inframe | NM_017680 | c.153_155del | p.51_52del |
| 298 | BLOOD | CNDP1 | 18 | 72223591 | 72223591 | - | TGC | inframe | NM_032649 | c.43_44insTGC | p.V15delinsVL |
| 298 | BLOOD | MLH1 | 3 | 37061893 | 37061893 | T | C | nonsynonymous | NM_000249 | c.T977C | p.V326A |
| 298 | BLOOD | DNAH1 | 3 | 52425267 | 52425267 | C | T | nonsynonymous | NM_015512 | c.C9814T | p.R3272C |
| 298 | BLOOD | SLMAP | 3 | 57817252 | 57817252 | G | A | nonsynonymous | NM_001304420 | c.G341A | p.R114Q |
| 298 | BLOOD | ASB10 | 7 | 150878220 | 150878220 | G | A | nonsynonymous | NM_001142459 | c.C910T | p.R304C |
| 298 | BLOOD | RBM33 | 7 | 155499605 | 155499605 | G | A | nonsynonymous | NM_053043 | c.G791A | p.R264Q |
| 298 | BLOOD | PCSK5 | 9 | 78547381 | 78547381 | C | G | nonsynonymous | NM_001190482 | c.C279G | p.F93L |
| 298 | BLOOD | GRIN3A | 9 | 104433048 | 104433048 | T | C | nonsynonymous | NM_133445 | c.A1646G | p.N549S |
| 298 | BLOOD | BRD3 | 9 | 136899924 | 136899924 | T | C | nonsynonymous | NM_007371 | c.A1964G | p.K655R |
| 298 | BLOOD | C14orf93 | 14 | 23456518 | 23456518 | T | G | nonsynonymous | NM_021944 | c.A1523C | p.N508T |
| 298 | BLOOD | TOMM20L | 14 | 58863051 | 58863051 | G | A | nonsynonymous | NM_207377 | c.G172A | p.G58S |
| 298 | BLOOD | KIAA0586 | 14 | 58917415 | 58917415 | G | C | nonsynonymous | NM_001244190 | c.G1035C | p.R345S |
| 298 | BLOOD | ABCC11 | 16 | 48234381 | 48234381 | G | A | nonsynonymous | NM_033151 | c.C1888T | p.R630W |
| 298 | BLOOD | LPCAT2 | 16 | 55562359 | 55562359 | G | A | nonsynonymous | NM_017839 | c.G382A | p.V128I |
| 298 | BLOOD | CES1 | 16 | 55862824 | 55862824 | C | T | nonsynonymous | NM_001025194 | c.G112A | p.V38I |
| 298 | BLOOD | MYO1C | 17 | 1372839 | 1372839 | C | T | nonsynonymous | NM_001080779 | c.G2596A | p.E866K |
| 298 | BLOOD | ZNRF4 | 19 | 5455543 | 5455543 | G | C | nonsynonymous | NM_181710 | c.G41C | p.S14T |
| 298 | BLOOD | RFX2 | 19 | 5997100 | 5997100 | C | T | nonsynonymous | NM_000635 | c.G1984A | p.G662R |
| 298 | BLOOD | NKG7 | 19 | 51875686 | 51875686 | G | A | nonsynonymous | NM_005601 | c.C104T | p.T35I |
| 298 | BLOOD | ARSH | X | 2945477 | 2945477 | C | T | nonsynonymous | NM_001011719 | c.C1160T | p.T387M |
| 298 | BLOOD | VCX2 | X | 8138080 | 8138080 | G | C | nonsynonymous | NM_016378 | c.C413G | p.T138S |
| 298 | BLOOD | CFAP47 | X | 35969297 | 35969297 | G | A | nonsynonymous | NM_152632 | c.G706A | p.V236M |
| 298 | BLOOD | CXorf36 | X | 45051111 | 45051111 | C | T | nonsynonymous | NM_176819 | c.G383A | p.R128K |
| 298 | BLOOD | MAP7D3 | X | 135310785 | 135310785 | T | C | nonsynonymous | NM_024597 | c.A1883G | p.Q628R |
| 298 | BLOOD | MAGEA3 | X | 151935240 | 151935240 | C | A | nonsynonymous | NM_005362 | c.G927T | p.L309F |
| 298 | BLOOD | MAGEA3 | X | 151935244 | 151935244 | A | G | nonsynonymous | NM_005362 | c.T923C | p.V308A |
| 298 | BLOOD | HCFC1 | X | 153220360 | 153220360 | A | G | nonsynonymous | NM_005334 | c.T3490C | p.S1164P |
| 299 | BLOOD | DST | 6 | 56483052 | 56483052 | T | C | nonsynonymous | NM_001723 | c.A5780G | p.H1927R |
| 299 | BLOOD | ARSD | X | 2833643 | 2833643 | C | A | nonsynonymous | NM_001669 | c.G954T | p.Q318H |
| 299 | BLOOD | ARSD | X | 2836181 | 2836181 | A | T | nonsynonymous | NM_001669 | c.T527A | p.M176K |
| 299 | BLOOD | ARSD | X | 2836184 | 2836184 | C | T | nonsynonymous | NM_001669 | c.G524A | p.G175D |
| 29 | LUNG | WDR66 | 12 | 122359397 | 122359397 | - | GAGGAGGAGGAGAAA | inframe | NM_144668 | c.186_187insGAGGAGGAGGAGAAA | p.G62delinsGEEEEK |
| 29 | LUNG | SIRPA | 20 | 1895963 | 1895963 | A | G | nonsynonymous | NM_001040023 | c.A298G | p.N100D |
| 29 | LUNG | SIRPA | 20 | 1895965 | 1895965 | C | A | nonsynonymous | NM_001040023 | c.C300A | p.N100K |
| 2 | BLOOD | PRR21 | 2 | 240982219 | 240982219 | A | G | nonsynonymous | NM_001080835 | c.T181C | p.S61P |
| 300 | BLOOD | PHLPP1 | 18 | 60497393 | 60497393 | T | C | nonsynonymous | NM_194449 | c.T1702C | p.C568R |
| 300 | BLOOD | CTAG2 | X | 153881773 | 153881773 | T | C | nonsynonymous | NM_020994 | c.A17G | p.Q6R |
| 301 | BLOOD | MYO18A | 17 | 27424239 | 27424239 | A | C | splicing |  |  |  |
| 302 | BLOOD | MUC4 | 3 | 195511918 | 195511918 | G | T | nonsynonymous | NM_018406 | c.C6533A | p.P2178H |
| 302 | BLOOD | FAM21C | 10 | 46254776 | 46254776 | A | C | nonsynonymous | NM_015262 | c.A1562C | p.Y521S |
| 302 | BLOOD | NPIPA5 | 16 | 15457598 | 15457598 | G | A | nonsynonymous | NM_001277325 | c.C971T | p.A324V |
| 302 | BLOOD | SIRPA | 20 | 1895963 | 1895963 | A | G | nonsynonymous | NM_001040023 | c.A298G | p.N100D |
| 302 | BLOOD | SIRPA | 20 | 1895965 | 1895965 | C | A | nonsynonymous | NM_001040023 | c.C300A | p.N100K |
| 302 | BLOOD | CEP250 | 20 | 34090351 | 34090351 | G | C | nonsynonymous | NM_007186 | c.G4154C | p.R1385P |
| 303 | BLOOD | CELSR2 | 1 | 109792735 | 109792735 | - | CGC | inframe | NM_001408 | c.34_35insCGC | p.T12delinsTP |
| 303 | BLOOD | ATAT1 | 6 | 30610614 | 30610614 | A | C | nonsynonymous | NM_024909 | c.A794C | p.H265P |
| 303 | BLOOD | TCEB3C,TCEB3CL | 18 | 44555312 | 44555312 | G | C | nonsynonymous | NM_145653 | c.C902G | p.S301C |
| 303 | BLOOD | GAGE12J | X | 49179711 | 49179711 | A | T | nonsynonymous | NM_001098406 | c.A39T | p.R13S |
| 304 | BLOOD | SLC16A14 | 2 | 230914604 | 230914604 | C | A | nonsynonymous | NM_152527 | c.G276T | p.L92F |
| 304 | BLOOD | H1FOO | 3 | 129268109 | 129268109 | G | A | nonsynonymous | NM_153833 | c.G644A | p.R215K |
| 305 | BLOOD | TBC1D9B | 5 | 179298526 | 179298526 | C | T | nonsynonymous | NM_198868 | c.G2420A | p.R807Q |
| 305 | BLOOD | TPTE | 21 | 10941967 | 10941967 | T | C | nonsynonymous | NM_199259 | c.A682G | p.I228V |
| 306 | BLOOD | SP8 | 7 | 20824941 | 20824943 | GCC | - | inframe | NM_198956 | c.439_441del | p.147_147del |
| 306 | BLOOD | MST1L | 1 | 17083872 | 17083872 | G | A | nonsynonymous | NM_001271733 | c.C1925T | p.P642L |
| 306 | BLOOD | SNX8 | 7 | 2294728 | 2294728 | G | A | nonsynonymous | NM_013321 | c.C1361T | p.P454L |
| 306 | BLOOD | CNTNAP3B | 9 | 43685357 | 43685357 | C | A | nonsynonymous | NM_001201380 | c.C63A | p.S21R |
| 306 | BLOOD | RIN2 | 20 | 19941419 | 19941419 | C | T | nonsynonymous | NM_001242581 | c.C574T | p.P192S |
| 307 | BLOOD | LRRIQ3 | 1 | 74575212 | 74575212 | - | T | frameshift | NM_001105659 | c.732dupA | p.Q245fs |
| 307 | BLOOD | CYP21A2 | 6 | 32006886 | 32006886 | G | A | nonsynonymous | NM_000500 | c.G308A | p.R103K |
| 307 | BLOOD | PDPR | 16 | 70182390 | 70182390 | T | G | nonsynonymous | NM_017990 | c.T1986G | p.N662K |
| 309 | BLOOD | ARHGAP22 | 10 | 49667877 | 49667877 | C | G | nonsynonymous | NM_021226 | c.G509C | p.R170P |
| 30 | BLOOD | ABCA1 | 9 | 107556793 | 107556793 | - | AA | splicing |  |  |  |
| 30 | BLOOD | WDR66 | 12 | 122359397 | 122359397 | - | GAGGAGGAGGAGAAA | inframe | NM_144668 | c.186_187insGAGGAGGAGGAGAAA | p.G62delinsGEEEEK |
| 30 | BLOOD | VCX2 | X | 8138165 | 8138168 | CCTC | - | frameshift | NM_016378 | c.325_328del | p.E109fs |
| 30 | BLOOD | MUC4 | 3 | 195505836 | 195505836 | G | C | nonsynonymous | NM_018406 | c.C12615G | p.H4205Q |
| 30 | BLOOD | HLA-DQB2 | 6 | 32726788 | 32726788 | T | C | nonsynonymous | NM_001300790 | c.A485G | p.D162G |
| 30 | BLOOD | GRHL2 | 8 | 102589675 | 102589675 | G | T | nonsynonymous | NM_024915 | c.G931T | p.D311Y |
| 310 | BLOOD | KTI12 | 1 | 52499071 | 52499097 | GCCCGCCACCTGAGGTCCCGCGATCGG | - | inframe | NM_138417 | c.337_363del | p.113_121del |
| 310 | BLOOD | LTF | 3 | 46501284 | 46501284 | - | CTT | inframe | NM_002343 | c.68_69insAAG | p.R23delinsRR |
| 310 | BLOOD | IRF5 | 7 | 128587352 | 128587381 | ACTCTGCAGCCGCCCACTCTGCGGCCGCCT | - | inframe | NM_001098630 | c.502_531del | p.168_177del |
| 310 | BLOOD | CTBP2 | 10 | 126715804 | 126715805 | CT | - | frameshift | NM_022802 | c.524_525del | p.Q175fs |
| 310 | BLOOD | PHLDA1 | 12 | 76424938 | 76424940 | TGC | - | inframe | NM_007350 | c.582_584del | p.194_195del |
| 310 | BLOOD | WDR66 | 12 | 122359397 | 122359397 | - | GAGGAGGAGGAGAAA | inframe | NM_144668 | c.186_187insGAGGAGGAGGAGAAA | p.G62delinsGEEEEK |
| 310 | BLOOD | GSE1 | 16 | 85682289 | 85682289 | - | C | frameshift | NM_014615 | c.359dupC | p.T120fs |
| 310 | BLOOD | BCL6B | 17 | 6928019 | 6928019 | - | CAG | inframe | NM_181844 | c.701_702insCAG | p.S234delinsSS |
| 310 | BLOOD | KRTAP4-5 | 17 | 39305775 | 39305775 | - | GGCAGCAGCTGGGGC | inframe | NM_033188 | c.244_245insGCCCCAGCTGCTGCC | p.Q82delinsRPSCCQ |
| 310 | BLOOD | KRTAP4-1 | 17 | 39340796 | 39340852 | CGGCAGCAGCTGGACATACCACAGCTGGGGTGGCAGGTGGTCTGACAGCAGAGTGGG | - | inframe | NM_033060 | c.246_254del | p.82_85del |
| 310 | BLOOD | GPR50 | X | 150349558 | 150349569 | CACCACTGGCCA | - | inframe | NM_004224 | c.1503_1514del | p.501_505del |
| 310 | BLOOD | CCDC13 | 3 | 42775098 | 42775098 | G | A | nonsynonymous | NM_144719 | c.C1375T | p.L459F |
| 310 | BLOOD | C3orf67 | 3 | 58849343 | 58849343 | C | T | nonsynonymous | NM_198463 | c.G1159A | p.V387M |
| 310 | BLOOD | IQCE | 7 | 2645564 | 2645564 | G | C | nonsynonymous | NM_152558 | c.G1798C | p.V600L |
| 310 | BLOOD | MUC3A | 7 | 100550389 | 100550389 | A | G | nonsynonymous | NM_005960 | c.A970G | p.T324A |
| 310 | BLOOD | DNA2 | 10 | 70182530 | 70182530 | G | A | nonsynonymous | NM_001080449 | c.C2326T | p.L776F |
| 310 | BLOOD | ASPSCR1 | 17 | 79968729 | 79968729 | C | T | nonsynonymous | NM_024083 | c.C1222T | p.R408C |
| 310 | BLOOD | TCEB3C,TCEB3CL | 18 | 44555312 | 44555312 | G | C | nonsynonymous | NM_145653 | c.C902G | p.S301C |
| 310 | BLOOD | ADAMTSL5 | 19 | 1507585 | 1507585 | C | A | nonsynonymous | NM_213604 | c.G659T | p.R220L |
| 310 | BLOOD | SUPT20HL1 | X | 24383057 | 24383057 | G | T | nonsynonymous | NM_001136234 | c.G2180T | p.G727V |
| 311 | BLOOD | ATXN1 | 6 | 16327913 | 16327915 | TGA | - | inframe | NM_000332 | c.627_629del | p.209_210del |
| 311 | BLOOD | HDGFRP2 | 19 | 4499633 | 4499647 | AGCTGGCCGGGGAGG | - | frameshift | NM_001001520 | c.1721_1722del | p.K574fs |
| 311 | BLOOD | UBXN11 | 1 | 26608843 | 26608843 | C | A | nonsynonymous | NM_183008 | c.G1510T | p.G504C |
| 311 | BLOOD | PTPN13 | 4 | 87666225 | 87666225 | A | G | nonsynonymous | NM_080683 | c.A2594G | p.H865R |
| 311 | BLOOD | MUC3A | 7 | 100550437 | 100550437 | C | T | nonsynonymous | NM_005960 | c.C1018T | p.P340S |
| 311 | BLOOD | DTD2 | 14 | 31926584 | 31926584 | G | A | nonsynonymous | NM_080664 | c.C16T | p.R6W |
| 312 | BLOOD | FMN2 | 1 | 240370914 | 240370946 | GCCCCCTCTACCCGGAGCGGGAATACCTCCTCC | - | inframe | NM_001305424 | c.2814_2846del | p.938_949del |
| 312 | BLOOD | CTAGE5 | 14 | 39784005 | 39784006 | TA | - | splicing |  |  |  |
| 312 | BLOOD | KRTAP4-5 | 17 | 39305775 | 39305775 | - | GGCAGCAGCTGGGGC | inframe | NM_033188 | c.244_245insGCCCCAGCTGCTGCC | p.Q82delinsRPSCCQ |
| 312 | BLOOD | KRTAP4-1 | 17 | 39340796 | 39340852 | CGGCAGCAGCTGGACATACCACAGCTGGGGTGGCAGGTGGTCTGACAGCAGAGTGGG | - | inframe | NM_033060 | c.246_254del | p.82_85del |
| 312 | BLOOD | SEMA4C | 2 | 97526671 | 97526671 | C | T | nonsynonymous | NM_017789 | c.G2194A | p.D732N |
| 312 | BLOOD | WDR27 | 6 | 170052078 | 170052078 | G | A | nonsynonymous | NM_182552 | c.C1429T | p.R477C |
| 312 | BLOOD | ABCA2 | 9 | 139906990 | 139906990 | C | T | nonsynonymous | NM_001606 | c.G5134A | p.G1712S |
| 312 | BLOOD | AHNAK2 | 14 | 105416174 | 105416174 | A | T | nonsynonymous | NM_138420 | c.T5614A | p.C1872S |
| 312 | BLOOD | CPAMD8 | 19 | 17108127 | 17108127 | C | T | nonsynonymous | NM_015692 | c.G1030A | p.D344N |
| 312 | BLOOD | P2RY8 | X | 1585079 | 1585079 | C | T | nonsynonymous | NM_178129 | c.G373A | p.V125I |
| 312 | BLOOD | ARSD | X | 2836181 | 2836181 | A | T | nonsynonymous | NM_001669 | c.T527A | p.M176K |
| 312 | BLOOD | ARSD | X | 2836184 | 2836184 | C | T | nonsynonymous | NM_001669 | c.G524A | p.G175D |
| 312 | BLOOD | ARSD | X | 2836211 | 2836211 | A | T | nonsynonymous | NM_001669 | c.T497A | p.L166Q |
| 312 | BLOOD | MXRA5 | X | 3228144 | 3228144 | C | G | nonsynonymous | NM_015419 | c.G8100C | p.E2700D |
| 312 | BLOOD | VCX | X | 7811747 | 7811747 | T | C | nonsynonymous | NM_013452 | c.T311C | p.L104P |
| 312 | BLOOD | VCX | X | 7812052 | 7812052 | G | A | nonsynonymous | NM_013452 | c.G616A | p.V206M |
| 312 | BLOOD | FLNA | X | 153578560 | 153578560 | C | T | nonsynonymous | NM_001110556 | c.G7172A | p.R2391H |
| 313 | BLOOD | HDGFRP2 | 19 | 4499633 | 4499647 | AGCTGGCCGGGGAGG | - | frameshift | NM_001001520 | c.1721_1722del | p.K574fs |
| 313 | BLOOD | IL17RC | 3 | 9959261 | 9959261 | A | G | nonsynonymous | NM_153461 | c.A262G | p.K88E |
| 313 | BLOOD | SYNPO2 | 4 | 119948566 | 119948566 | C | T | nonsynonymous | NM_001128933 | c.C1042T | p.P348S |
| 313 | BLOOD | ABCA13 | 7 | 48556388 | 48556388 | T | A | nonsynonymous | NM_152701 | c.T13708A | p.S4570T |
| 313 | BLOOD | PKP3 | 11 | 396836 | 396836 | C | A | nonsynonymous | NM_001303029 | c.C380A | p.P127Q |
| 313 | BLOOD | ZNF814 | 19 | 58385546 | 58385546 | G | T | nonsynonymous | NM_001144989 | c.C1212A | p.D404E |
| 314 | BLOOD | TP53AIP1 | 11 | 128807650 | 128807650 | - | C | frameshift | NM_022112 | c.63dupG | p.Q22fs |
| 314 | BLOOD | FAM155A | 13 | 108518686 | 108518686 | - | CTG | inframe | NM_001080396 | c.258_259insCAG | p.R87delinsQR |
| 314 | BLOOD | KRTAP4-5 | 17 | 39305775 | 39305775 | - | GGCAGCAGCTGGGGC | inframe | NM_033188 | c.244_245insGCCCCAGCTGCTGCC | p.Q82delinsRPSCCQ |
| 314 | BLOOD | SSPO | 7 | 149473580 | 149473580 | A | G | nonsynonymous | NM_198455 | c.A196G | p.T66A |
| 314 | BLOOD | OR52L1 | 11 | 6007949 | 6007949 | G | A | nonsynonymous | NM_001005173 | c.C212T | p.P71L |
| 314 | BLOOD | PCDH17 | 13 | 58207236 | 58207236 | C | G | nonsynonymous | NM_001040429 | c.C556G | p.R186G |
| 314 | BLOOD | ADAMTS7 | 15 | 79058967 | 79058967 | G | T | nonsynonymous | NM_014272 | c.C3286A | p.P1096T |
| 314 | BLOOD | GATAD2A | 19 | 19576174 | 19576174 | G | A | nonsynonymous | NM_017660 | c.G20A | p.R7Q |
| 315 | BLOOD | CTBS | 1 | 85040025 | 85040033 | AGCAGCGCT | - | inframe | NM_004388 | c.66_74del | p.22_25del |
| 315 | BLOOD | ARID1B | 6 | 157100396 | 157100396 | - | CGC | inframe | NM_017519 | c.1333_1334insCGC | p.A445delinsAP |
| 315 | BLOOD | VCX2 | X | 8138165 | 8138168 | CCTC | - | frameshift | NM_016378 | c.325_328del | p.E109fs |
| 315 | BLOOD | MUC5B | 11 | 1264717 | 1264717 | C | T | nonsynonymous | NM_002458 | c.C6607T | p.P2203S |
| 315 | BLOOD | OR11H2 | 14 | 20181502 | 20181502 | C | T | nonsynonymous | NM_001197287 | c.G574A | p.V192I |
| 315 | BLOOD | NFATC4 | 14 | 24845185 | 24845185 | C | T | nonsynonymous | NM_004554 | c.C1934T | p.T645M |
| 315 | BLOOD | AHNAK2 | 14 | 105415229 | 105415229 | T | C | nonsynonymous | NM_138420 | c.A6559G | p.M2187V |
| 315 | BLOOD | NMT1 | 17 | 43138744 | 43138744 | T | G | nonsynonymous | NM_021079 | c.T47G | p.L16R |
| 315 | BLOOD | KANSL1 | 17 | 44249199 | 44249199 | T | G | nonsynonymous | NM_001193466 | c.A311C | p.K104T |
| 315 | BLOOD | HMHA1 | 19 | 1085965 | 1085965 | C | T | nonsynonymous | NM_012292 | c.C3371T | p.T1124I |
| 315 | BLOOD | TJP3 | 19 | 3746846 | 3746846 | C | T | nonsynonymous | NM_001267560 | c.C2294T | p.T765M |
| 315 | BLOOD | CYP4F11 | 19 | 16045166 | 16045166 | G | C | nonsynonymous | NM_021187 | c.C53G | p.P18R |
| 315 | BLOOD | ARMC6 | 19 | 19168228 | 19168228 | C | A | nonsynonymous | NM_001199196 | c.C1297A | p.Q433K |
| 315 | BLOOD | ZNF404 | 19 | 44377205 | 44377205 | T | G | nonsynonymous | NM_001033719 | c.A1152C | p.E384D |
| 315 | BLOOD | CGB7 | 19 | 49558216 | 49558216 | C | T | nonsynonymous | NM_033142 | c.G65A | p.R22K |
| 316 | BLOOD | WDR66 | 12 | 122359408 | 122359408 | G | A | nonsynonymous | NM_144668 | c.G197A | p.G66E |
| 316 | BLOOD | PLAC4 | 21 | 42551425 | 42551425 | C | G | nonsynonymous | NM_182832 | c.G131C | p.R44P |
| 317 | BLOOD | TBP | 6 | 170871013 | 170871013 | - | CAG | inframe | NM_003194 | c.189_190insCAG | p.Q63delinsQQ |
| 317 | BLOOD | GOLGA2 | 9 | 131020796 | 131020798 | CCT | - | inframe | NM_004486 | c.2144_2146del | p.715_716del |
| 317 | BLOOD | WDR66 | 12 | 122359397 | 122359397 | - | GAGGAGGAGGAGAAA | inframe | NM_144668 | c.186_187insGAGGAGGAGGAGAAA | p.G62delinsGEEEEK |
| 317 | BLOOD | APOBR | 16 | 28507398 | 28507424 | GGGACAGCCTCAGGAGGGGAGGAGGCC | - | inframe | NM_018690 | c.1036_1062del | p.346_354del |
| 317 | BLOOD | TRAPPC12 | 2 | 3391458 | 3391458 | G | A | nonsynonymous | NM_016030 | c.G64A | p.A22T |
| 317 | BLOOD | ARSD | X | 2836238 | 2836238 | G | A | nonsynonymous | NM_001669 | c.C470T | p.S157F |
| 318 | BLOOD | FGFRL1 | 4 | 1019055 | 1019056 | CA | - | frameshift | NM_001004358 | c.1435_1436del | p.H479fs |
| 318 | BLOOD | DNAJC4 | 11 | 63999356 | 63999367 | ACTTATTATGAA | - | inframe | NM_005528 | c.100_111del | p.34_37del |
| 318 | BLOOD | NCOR2 | 12 | 124824721 | 124824721 | - | GCCGCTGCT | inframe | NM_006312 | c.5517_5518insAGCAGCGGC | p.G1840delinsSSGG |
| 318 | BLOOD | DNAJB12 | 10 | 74114714 | 74114714 | A | C | nonsynonymous | NM_001002762 | c.T44G | p.V15G |
| 318 | BLOOD | KRTAP4-8 | 17 | 39253953 | 39253953 | G | T | nonsynonymous | NM_031960 | c.C384A | p.S128R |
| 318 | BLOOD | EPN1 | 19 | 56203234 | 56203234 | T | A | nonsynonymous | NM_001130072 | c.T877A | p.S293T |
| 319 | BLOOD | SFTPC | 8 | 22021060 | 22021061 | GT | - | splicing |  |  |  |
| 319 | BLOOD | ABCA1 | 9 | 107556793 | 107556793 | - | A | splicing |  |  |  |
| 319 | BLOOD | KRTAP5-5 | 11 | 1651199 | 1651228 | AGGCTGTGGGGGCTGTGGCTCCGGCTGTGC | - | inframe | NM_001001480 | c.129_158del | p.43_53del |
| 319 | BLOOD | WDR66 | 12 | 122359408 | 122359408 | G | A | nonsynonymous | NM_144668 | c.G197A | p.G66E |
| 319 | BLOOD | IL9R | X | 155239824 | 155239824 | A | G | nonsynonymous | NM_002186 | c.A1316G | p.N439S |
| 319 | BLOOD | IL9R | X | 155239827 | 155239827 | A | G | nonsynonymous | NM_002186 | c.A1319G | p.N440S |
| 31 | BLOOD | ITPKB | 1 | 226924876 | 226924884 | CTGCCGCTG | - | inframe | NM_002221 | c.276_284del | p.92_95del |
| 31 | BLOOD | FAM86B2 | 8 | 12287957 | 12287957 | C | T | nonsynonymous | NM_001137610 | c.G244A | p.E82K |
| 31 | BLOOD | GOLGA8R | 15 | 30696515 | 30696515 | C | A | nonsynonymous | NM_001282484 | c.G1504T | p.A502S |
| 31 | BLOOD | GOLGA8R | 15 | 30696517 | 30696517 | C | T | nonsynonymous | NM_001282484 | c.G1502A | p.R501H |
| 320 | BLOOD | ANKRD36 | 2 | 97877440 | 97877440 | T | C | nonsynonymous | NM_001164315 | c.T3431C | p.M1144T |
| 320 | BLOOD | CPNE3 | 8 | 87563330 | 87563330 | T | G | splicing |  |  |  |
| 321 | BLOOD | UBXN11 | 1 | 26608878 | 26608883 | CCGGGA | - | inframe | NM_183008 | c.1470_1475del | p.490_492del |
| 321 | BLOOD | GFM1 | 3 | 158383162 | 158383162 | T | G | nonsynonymous | NM_024996 | c.T1417G | p.F473V |
| 321 | BLOOD | MUC20 | 3 | 195447886 | 195447886 | G | C | nonsynonymous | NM_001282506 | c.G8C | p.C3S |
| 321 | BLOOD | OR1D5 | 17 | 2966273 | 2966273 | G | A | nonsynonymous | NM_014566 | c.C629T | p.P210L |
| 322 | BLOOD | MUC3A | 7 | 100550389 | 100550389 | A | G | nonsynonymous | NM_005960 | c.A970G | p.T324A |
| 322 | BLOOD | TPSD1 | 16 | 1308333 | 1308333 | G | A | nonsynonymous | NM_012217 | c.G685A | p.G229S |
| 322 | BLOOD | FDXR | 17 | 72859001 | 72859001 | T | C | nonsynonymous | NM_024417 | c.A1414G | p.T472A |
| 322 | BLOOD | RENBP | X | 153205560 | 153205560 | G | A | nonsynonymous | NM_002910 | c.C1072T | p.R358C |
| 323 | BLOOD | CTAGE5 | 14 | 39784005 | 39784006 | TA | - | splicing |  |  |  |
| 324 | BLOOD | JMY | 5 | 78610479 | 78610479 | C | A | nonsynonymous | NM_152405 | c.C2464A | p.P822T |
| 324 | BLOOD | C15orf39 | 15 | 75498580 | 75498580 | C | T | nonsynonymous | NM_015492 | c.C191T | p.A64V |
| 324 | BLOOD | GNAS | 20 | 57484421 | 57484421 | G | A | nonsynonymous | NM_080425 | c.G2531A | p.R844H |
| 325 | BLOOD | IL27 | 16 | 28511005 | 28511005 | G | - | frameshift | NM_145659 | c.699delC | p.P233fs |
| 325 | BLOOD | HLA-A | 6 | 29911260 | 29911260 | A | C | nonsynonymous | NM_002116 | c.A559C | p.T187P |
| 325 | BLOOD | CCDC15 | 11 | 124873801 | 124873801 | C | A | nonsynonymous | NM_025004 | c.C2253A | p.F751L |
| 325 | BLOOD | KRTAP9-6 | 17 | 39421886 | 39421886 | A | G | nonsynonymous | NM_001277331 | c.A257G | p.Y86C |
| 326 | BLOOD | MUC4 | 3 | 195507925 | 195507925 | C | T | nonsynonymous | NM_018406 | c.G10526A | p.G3509D |
| 327 | BLOOD | PCDH11X | X | 91723313 | 91723313 | T | C | stoploss | NM_001168361 | c.T3196C | p.X1066Q |
| 328 | BLOOD | HSPBP1 | 19 | 55790886 | 55790886 | - | GCCGCCGCC | inframe | NM_001130106 | c.90_91insGGCGGCGGC | p.S31delinsGGGS |
| 328 | BLOOD | ESPN | 1 | 6500762 | 6500762 | G | A | nonsynonymous | NM_031475 | c.G752A | p.G251D |
| 328 | BLOOD | MUC4 | 3 | 195513439 | 195513439 | G | A | nonsynonymous | NM_018406 | c.C5012T | p.S1671F |
| 328 | BLOOD | PNPLA8 | 7 | 108155377 | 108155377 | G | A | nonsynonymous | NM_015723 | c.C559T | p.R187C |
| 328 | BLOOD | NUB1 | 7 | 151074223 | 151074223 | T | G | nonsynonymous | NM_001243351 | c.T1832G | p.L611R |
| 328 | BLOOD | HCN4 | 15 | 73615786 | 73615786 | G | C | nonsynonymous | NM_005477 | c.C2648G | p.P883R |
| 328 | BLOOD | GLTSCR1 | 19 | 48183865 | 48183865 | G | A | nonsynonymous | NM_015711 | c.G1438A | p.V480I |
| 329 | BLOOD | ANKRD36B | 2 | 98165912 | 98165912 | C | T | nonsynonymous | NM_025190 | c.G1447A | p.E483K |
| 329 | BLOOD | NDUFS1 | 2 | 206991308 | 206991308 | G | T | nonsynonymous | NM_005006 | c.C2047A | p.P683T |
| 329 | BLOOD | LRRFIP1 | 2 | 238536328 | 238536328 | C | A | nonsynonymous | NM_001137550 | c.C41A | p.P14H |
| 329 | BLOOD | IL17RC | 3 | 9975029 | 9975029 | C | A | nonsynonymous | NM_153461 | c.C2128A | p.H710N |
| 329 | BLOOD | XPC | 3 | 14189448 | 14189448 | C | A | nonsynonymous | NM_004628 | c.G2474T | p.W825L |
| 329 | BLOOD | EPHB1 | 3 | 134644706 | 134644706 | C | A | nonsynonymous | NM_004441 | c.C107A | p.A36D |
| 329 | BLOOD | GTF2IRD1 | 7 | 73929829 | 73929829 | G | T | nonsynonymous | NM_016328 | c.G324T | p.E108D |
| 329 | BLOOD | POR | 7 | 75583322 | 75583322 | G | T | nonsynonymous | NM_000941 | c.G12T | p.M4I |
| 329 | BLOOD | GOT1L1 | 8 | 37792612 | 37792612 | G | T | nonsynonymous | NM_152413 | c.C1051A | p.H351N |
| 329 | BLOOD | PPAPDC3 | 9 | 134165416 | 134165416 | G | T | nonsynonymous | NM_032728 | c.G32T | p.R11L |
| 329 | BLOOD | STK32C | 10 | 134021525 | 134021525 | C | A | nonsynonymous | NM_173575 | c.G1450T | p.G484W |
| 329 | BLOOD | ACSM4 | 12 | 7463314 | 7463314 | G | T | nonsynonymous | NM_001080454 | c.G592T | p.G198W |
| 329 | BLOOD | ACOT2 | 14 | 74036558 | 74036558 | G | T | nonsynonymous | NM_006821 | c.G614T | p.R205L |
| 329 | BLOOD | IRX5 | 16 | 54967390 | 54967390 | G | T | nonsynonymous | NM_005853 | c.G1057T | p.G353W |
| 329 | BLOOD | ELMO3 | 16 | 67237298 | 67237298 | G | T | nonsynonymous | NM_024712 | c.G2008T | p.G670W |
| 329 | BLOOD | ASPSCR1 | 17 | 79974929 | 79974929 | G | T | nonsynonymous | NM_024083 | c.G1588T | p.G530W |
| 329 | BLOOD | R3HDM4 | 19 | 899632 | 899632 | G | T | nonsynonymous | NM_138774 | c.C616A | p.Q206K |
| 329 | BLOOD | PLXNB3 | X | 153040431 | 153040431 | C | A | nonsynonymous | NM_005393 | c.C4028A | p.P1343H |
| 32 | BLOOD | UHRF1BP1L | 12 | 100492135 | 100492136 | CT | - | frameshift | NM_015054 | c.522_523del | p.R174fs |
| 32 | BLOOD | ZNF705G | 8 | 7217837 | 7217837 | A | C | nonsynonymous | NM_001164457 | c.T157G | p.S53A |
| 32 | BLOOD | PPAP2C | 19 | 281468 | 281468 | G | A | nonsynonymous | NM_177526 | c.C619T | p.R207W |
| 32 | BLOOD | CGB1 | 19 | 49539560 | 49539560 | T | C | nonsynonymous | NM_033377 | c.A10G | p.R4G |
| 330 | BLOOD | HS6ST1 | 2 | 129075877 | 129075877 | G | T | nonsynonymous | NM_004807 | c.C261A | p.D87E |
| 330 | BLOOD | RAB39A | 11 | 107799510 | 107799510 | G | T | nonsynonymous | NM_017516 | c.G216T | p.Q72H |
| 330 | BLOOD | HERC2 | 15 | 28483903 | 28483903 | C | A | nonsynonymous | NM_004667 | c.G3593T | p.G1198V |
| 330 | BLOOD | SYN1 | X | 47435577 | 47435577 | C | A | nonsynonymous | NM_006950 | c.G1111T | p.A371S |
| 331 | BLOOD | EMCN | 4 | 101401162 | 101401164 | AAC | - | inframe | NM_016242 | c.97_99del | p.33_33del |
| 331 | BLOOD | TET2 | 4 | 106157588 | 106157588 | - | A | frameshift | NM_001127208 | c.2490dupA | p.I830fs |
| 331 | BLOOD | ARID1B | 6 | 157100396 | 157100396 | - | CGC | inframe | NM_017519 | c.1333_1334insCGC | p.A445delinsAP |
| 331 | BLOOD | SMPD1 | 11 | 6411931 | 6411942 | CTGGTGCTGGCG | - | inframe | NM_000543 | c.103_114del | p.35_38del |
| 331 | BLOOD | RBM25 | 14 | 73572607 | 73572608 | AG | - | frameshift | NM_021239 | c.1195_1196del | p.R399fs |
| 331 | BLOOD | PLAC4 | 21 | 42551272 | 42551272 | C | - | frameshift | NM_182832 | c.284delG | p.G95fs |
| 331 | BLOOD | VIPR1 | 3 | 42576582 | 42576582 | G | A | nonsynonymous | NM_001251885 | c.G1045A | p.V349M |
| 331 | BLOOD | SLC37A2 | 11 | 124951767 | 124951767 | G | A | nonsynonymous | NM_001145290 | c.G850A | p.A284T |
| 331 | BLOOD | KRT38 | 17 | 39593722 | 39593722 | C | T | nonsynonymous | NM_006771 | c.G1313A | p.G438D |
| 331 | BLOOD | SIGLEC5 | 19 | 52133270 | 52133270 | G | T | nonsynonymous | NM_003830 | c.C237A | p.D79E |
| 331 | BLOOD | PLAC4 | 21 | 42551270 | 42551270 | A | G | nonsynonymous | NM_182832 | c.T286C | p.Y96H |
| 331 | BLOOD | RFPL3 | 22 | 32756703 | 32756703 | C | T | nonsynonymous | NM_001098535 | c.C838T | p.R280C |
| 331 | BLOOD | CELSR1 | 22 | 46793604 | 46793604 | C | T | nonsynonymous | NM_014246 | c.G5668A | p.A1890T |
| 331 | BLOOD | RBMX | X | 135960230 | 135960230 | C | T | nonsynonymous | NM_002139 | c.G232A | p.A78T |
| 332 | BLOOD | ABCA1 | 9 | 107556793 | 107556793 | - | AA | splicing |  |  |  |
| 332 | BLOOD | CCDC129 | 7 | 31609381 | 31609381 | A | G | nonsynonymous | NM_194300 | c.A266G | p.Y89C |
| 332 | BLOOD | SLC24A1 | 15 | 65943118 | 65943118 | G | T | nonsynonymous | NM_004727 | c.G2631T | p.E877D |
| 333 | BLOOD | MUC4 | 3 | 195507925 | 195507925 | C | T | nonsynonymous | NM_018406 | c.G10526A | p.G3509D |
| 333 | BLOOD | ATXN3 | 14 | 92537379 | 92537379 | T | C | nonsynonymous | NM_001164782 | c.A43G | p.T15A,ATXN3 |
| 333 | BLOOD | ADAMTS7 | 15 | 79058882 | 79058882 | A | G | nonsynonymous | NM_014272 | c.T3371C | p.V1124A |
| 334 | BLOOD | FMN2 | 1 | 240370914 | 240370946 | GCCCCCTCTACCCGGAGCGGGAATACCTCCTCC | - | inframe | NM_001305424 | c.2814_2846del | p.938_949del |
| 334 | BLOOD | APOBR | 16 | 28507398 | 28507424 | GGGACAGCCTCAGGAGGGGAGGAGGCC | - | inframe | NM_018690 | c.1036_1062del | p.346_354del |
| 334 | BLOOD | LIMK2 | 22 | 31672776 | 31672776 | - | C | frameshift | NM_001031801 | c.1712dupC | p.A571fs |
| 334 | BLOOD | CHST7 | X | 46434472 | 46434472 | G | A | nonsynonymous | NM_019886 | c.G1106A | p.R369H |
| 335 | BLOOD | MED15 | 22 | 20920813 | 20920813 | - | CAG | inframe | NM_001003891 | c.750_751insCAG | p.Q250delinsQQ |
| 335 | BLOOD | MUC4 | 3 | 195507193 | 195507193 | G | A | nonsynonymous | NM_018406 | c.C11258T | p.A3753V |
| 335 | BLOOD | MUC4 | 3 | 195508163 | 195508163 | T | G | nonsynonymous | NM_018406 | c.A10288C | p.T3430P |
| 335 | BLOOD | EP400 | 12 | 132445256 | 132445256 | A | C | nonsynonymous | NM_015409 | c.A92C | p.H31P |
| 335 | BLOOD | CASKIN1 | 16 | 2229617 | 2229617 | G | A | nonsynonymous | NM_020764 | c.C3752T | p.P1251L |
| 335 | BLOOD | MADCAM1 | 19 | 501762 | 501762 | A | C | nonsynonymous | NM_130760 | c.A761C | p.Q254P |
| 335 | BLOOD | IL9R | X | 155239824 | 155239824 | A | G | nonsynonymous | NM_002186 | c.A1316G | p.N439S |
| 335 | BLOOD | IL9R | X | 155239827 | 155239827 | A | G | nonsynonymous | NM_002186 | c.A1319G | p.N440S |
| 336 | BLOOD | ZC3H3 | 8 | 144522387 | 144522389 | GAG | - | inframe | NM_015117 | c.2637_2639del | p.879_880del |
| 336 | BLOOD | RP1L1 | 8 | 10467605 | 10467605 | C | T | nonsynonymous | NM_178857 | c.G4003A | p.G1335R |
| 336 | BLOOD | ATXN3 | 14 | 92537379 | 92537379 | T | C | nonsynonymous | NM_001164782 | c.A43G | p.T15A,ATXN3 |
| 336 | BLOOD | NCOR1 | 17 | 16097870 | 16097870 | C | A | nonsynonymous | NM_006311 | c.G14T | p.G5V |
| 336 | BLOOD | CCDC43 | 17 | 42767010 | 42767010 | C | T | nonsynonymous | NM_144609 | c.G112A | p.A38T |
| 336 | BLOOD | FSCN2 | 17 | 79495897 | 79495897 | A | G | nonsynonymous | NM_012418 | c.A340G | p.T114A |
| 336 | BLOOD | MADCAM1 | 19 | 501762 | 501762 | A | C | nonsynonymous | NM_130760 | c.A761C | p.Q254P |
| 336 | BLOOD | KLK14 | 19 | 51585978 | 51585978 | C | T | splicing |  |  |  |
| 336 | BLOOD | PPP1R12C | 19 | 55606033 | 55606033 | G | A | nonsynonymous | NM_001271618 | c.C1585T | p.R529W |
| 336 | BLOOD | TMEM150B | 19 | 55831502 | 55831502 | G | A | nonsynonymous | NM_001085488 | c.C229T | p.L77F |
| 336 | BLOOD | ZNF543 | 19 | 57838058 | 57838058 | G | A | stopgain | NM_213598 | c.G203A | p.W68X |
| 336 | BLOOD | ZNF417 | 19 | 58423509 | 58423509 | C | A | stopgain | NM_152475 | c.G82T | p.E28X |
| 336 | BLOOD | PRR5 | 22 | 45132853 | 45132853 | C | T | nonsynonymous | NM_181333 | c.C893T | p.P298L |
| 336 | BLOOD | VCX | X | 7811645 | 7811645 | C | G | nonsynonymous | NM_013452 | c.C209G | p.A70G |
| 337 | BLOOD | BOD1L1 | 4 | 13602229 | 13602229 | G | C | nonsynonymous | NM_148894 | c.C6295G | p.L2099V |
| 337 | BLOOD | APOBEC1 | 12 | 7807217 | 7807217 | C | T | nonsynonymous | NM_001304566 | c.G28A | p.G10S |
| 337 | BLOOD | TAS2R43 | 12 | 11244653 | 11244653 | A | C | nonsynonymous | NM_176884 | c.T176G | p.L59R |
| 337 | BLOOD | POC1B-GALNT4 | 12 | 89919703 | 89919703 | G | A | nonsynonymous | NM_001199782 | c.C230T | p.P77L |
| 337 | BLOOD | OR1D5 | 17 | 2966273 | 2966273 | G | A | nonsynonymous | NM_014566 | c.C629T | p.P210L |
| 337 | BLOOD | ENTHD2 | 17 | 79203057 | 79203057 | T | G | nonsynonymous | NM_144679 | c.A1249C | p.T417P |
| 338 | BLOOD | SH2D2A | 1 | 156777073 | 156777073 | T | G | nonsynonymous | NM_001161444 | c.A1067C | p.H356P |
| 338 | BLOOD | EXO1 | 1 | 242020734 | 242020734 | C | A | nonsynonymous | NM_130398 | c.C493A | p.Q165K |
| 338 | BLOOD | EIF2A | 3 | 150290165 | 150290165 | T | G | nonsynonymous | NM_032025 | c.T1232G | p.V411G |
| 338 | BLOOD | CMYA5 | 5 | 79029466 | 79029466 | C | A | nonsynonymous | NM_153610 | c.C4878A | p.D1626E |
| 338 | BLOOD | FOXC1 | 6 | 1612234 | 1612234 | G | T | nonsynonymous | NM_001453 | c.G1554T | p.L518F |
| 338 | BLOOD | ZNF706 | 8 | 102213962 | 102213962 | C | G | nonsynonymous | NM_016096 | c.G8C | p.R3P |
| 338 | BLOOD | CLIP1 | 12 | 122812697 | 122812697 | C | T | nonsynonymous | NM_002956 | c.G3013A | p.E1005K |
| 338 | BLOOD | AKAP13 | 15 | 86287035 | 86287035 | A | C | nonsynonymous | NM_007200 | c.A8371C | p.T2791P |
| 338 | BLOOD | NPHS1 | 19 | 36336437 | 36336437 | T | C | nonsynonymous | NM_004646 | c.A1763G | p.E588G |
| 338 | BLOOD | PNKP | 19 | 50370425 | 50370425 | C | G | nonsynonymous | NM_007254 | c.G37C | p.E13Q |
| 339 | BLOOD | TCP11L1 | 11 | 33087551 | 33087551 | A | C | nonsynonymous | NM_018393 | c.A1148C | p.H383P |
| 33 | BLOOD | NEB | 2 | 152424933 | 152424933 | - | A | splicing |  |  |  |
| 33 | BLOOD | HDGFRP2 | 19 | 4499633 | 4499647 | AGCTGGCCGGGGAGG | - | frameshift | NM_001001520 | c.1721_1722del | p.K574fs |
| 33 | BLOOD | CROCC | 1 | 17257061 | 17257061 | G | T | nonsynonymous | NM_014675 | c.G821T | p.R274L |
| 33 | BLOOD | ANKRD36 | 2 | 97877440 | 97877440 | T | C | nonsynonymous | NM_001164315 | c.T3431C | p.M1144T |
| 33 | BLOOD | HS6ST1 | 2 | 129075939 | 129075939 | T | A | stopgain | NM_004807 | c.A199T | p.K67X |
| 33 | BLOOD | GOLGA6L10 | 15 | 82635194 | 82635194 | T | C | nonsynonymous | NM_001164465 | c.A1505G | p.E502G |
| 33 | BLOOD | ZNF814 | 19 | 58385748 | 58385748 | G | A | nonsynonymous | NM_001144989 | c.C1010T | p.A337V |
| 340 | BLOOD | FAM90A1 | 12 | 8374781 | 8374781 | - | ACG | inframe | NM_018088 | c.1031_1032insCGT | p.T344delinsTV |
| 340 | BLOOD | KCNN3 | 1 | 154842244 | 154842244 | A | T | nonsynonymous | NM_002249 | c.T197A | p.L66H |
| 340 | BLOOD | BCLAF1 | 6 | 136593183 | 136593183 | G | C | nonsynonymous | NM_014739 | c.C1993G | p.H665D |
| 340 | BLOOD | C9orf171 | 9 | 135285840 | 135285840 | C | T | nonsynonymous | NM_207417 | c.C182T | p.P61L |
| 340 | BLOOD | ABCA2 | 9 | 139915879 | 139915879 | G | A | nonsynonymous | NM_001606 | c.C862T | p.R288W |
| 340 | BLOOD | RBMX | X | 135957716 | 135957716 | A | G | nonsynonymous | NM_001164803 | c.T245C | p.M82T |
| 341 | BLOOD | MUC4 | 3 | 195511814 | 195511814 | T | C | nonsynonymous | NM_018406 | c.A6637G | p.S2213G |
| 341 | BLOOD | ZNF318 | 6 | 43320205 | 43320205 | C | A | stopgain | NM_014345 | c.G2680T | p.E894X |
| 341 | BLOOD | ALX4 | 11 | 44331173 | 44331173 | C | T | nonsynonymous | NM_021926 | c.G440A | p.S147N |
| 341 | BLOOD | NRK | X | 105168689 | 105168689 | C | A | nonsynonymous | NM_198465 | c.C2978A | p.A993E |
| 342 | BLOOD | HLA-A | 6 | 29911260 | 29911260 | A | C | nonsynonymous | NM_002116 | c.A559C | p.T187P |
| 342 | BLOOD | SOGA3 | 6 | 127837125 | 127837125 | T | C | nonsynonymous | NM_001012279 | c.A635G | p.E212G |
| 342 | BLOOD | TRUB1 | 10 | 116698112 | 116698112 | A | C | nonsynonymous | NM_139169 | c.A100C | p.T34P |
| 342 | BLOOD | NLRP5 | 19 | 56538513 | 56538513 | A | G | nonsynonymous | NM_153447 | c.A914G | p.Q305R |
| 343 | BLOOD | KDM6B | 17 | 7752532 | 7752543 | CAGAAGGAGCAT | - | inframe | NM_001080424 | c.2926_2937del | p.976_979del |
| 343 | BLOOD | KANK1 | 9 | 711236 | 711236 | C | T | nonsynonymous | NM_001256876 | c.C470T | p.T157I |
| 343 | BLOOD | DDX11 | 12 | 31237978 | 31237978 | C | T | nonsynonymous | NM_001257144 | c.C556T | p.R186W |
| 343 | BLOOD | SLC35G4 | 18 | 11610382 | 11610382 | C | A | nonsynonymous | NM_001282300 | c.C788A | p.T263N |
| 343 | BLOOD | P2RY4 | X | 69478775 | 69478775 | A | C | nonsynonymous | NM_002565 | c.T700G | p.S234A |
| 344 | LUNG | FMN2 | 1 | 240370914 | 240370946 | GCCCCCTCTACCCGGAGCGGGAATACCTCCTCC | - | inframe | NM_001305424 | c.2814_2846del | p.938_949del |
| 344 | LUNG | RP1L1 | 8 | 10466004 | 10466024 | CTGGGCCTCCCCTTCAGCCTC | - | inframe | NM_178857 | c.5584_5604del | p.1862_1868del |
| 344 | LUNG | CTAGE5 | 14 | 39784005 | 39784006 | TA | - | splicing |  |  |  |
| 344 | LUNG | AR | X | 66765149 | 66765151 | TGC | - | inframe | NM_000044 | c.161_163del | p.54_55del |
| 344 | LUNG | WDR66 | 12 | 122359408 | 122359408 | G | A | nonsynonymous | NM_144668 | c.G197A | p.G66E |
| 344 | LUNG | DTD2 | 14 | 31926584 | 31926584 | G | A | nonsynonymous | NM_080664 | c.C16T | p.R6W |
| 345 | LUNG | NUTM2F | 9 | 97080945 | 97080947 | AGA | - | inframe | NM_017561 | c.2071_2073del | p.691_691del |
| 345 | LUNG | KRTAP4-5 | 17 | 39305775 | 39305775 | - | GGCAGCAGCTGGGGC | inframe | NM_033188 | c.244_245insGCCCCAGCTGCTGCC | p.Q82delinsRPSCCQ |
| 345 | LUNG | KRTAP17-1 | 17 | 39471753 | 39471767 | GCCCCCGCAGCCAGA | - | inframe | NM_031964 | c.136_150del | p.46_50del |
| 345 | LUNG | MUC4 | 3 | 195513461 | 195513461 | G | A | nonsynonymous | NM_018406 | c.C4990T | p.P1664S |
| 345 | LUNG | TSPAN32 | 11 | 2325427 | 2325427 | T | C | nonsynonymous | NM_139022 | c.T272C | p.M91T |
| 346 | LUNG | MSH3 | 5 | 79950724 | 79950724 | - | CCGCAGCGC | inframe | NM_002439 | c.178_179insCCGCAGCGC | p.A60delinsAAAP |
| 346 | LUNG | KRTAP5-5 | 11 | 1651199 | 1651228 | AGGCTGTGGGGGCTGTGGCTCCGGCTGTGC | - | inframe | NM_001001480 | c.129_158del | p.43_53del |
| 346 | LUNG | RTL1 | 14 | 101350670 | 101350670 | - | TCT | inframe | NM_001134888 | c.455_456insAGA | p.E152delinsEE |
| 346 | LUNG | CSMD3 | 8 | 113326817 | 113326817 | C | T | nonsynonymous | NM_198123 | c.G7390A | p.D2464N |
| 346 | LUNG | PSMA6 | 14 | 35777256 | 35777256 | A | C | nonsynonymous | NM_002791 | c.A133C | p.K45Q |
| 346 | LUNG | GOLGA6L10 | 15 | 82635194 | 82635194 | T | C | nonsynonymous | NM_001164465 | c.A1505G | p.E502G |
| 347 | LUNG | TCHH | 1 | 152084694 | 152084696 | CTC | - | inframe | NM_007113 | c.997_999del | p.333_333del |
| 347 | LUNG | RNF123 | 3 | 49742583 | 49742583 | C | T | nonsynonymous | NM_022064 | c.C2126T | p.T709M |
| 347 | LUNG | POLRMT | 19 | 632849 | 632849 | C | T | nonsynonymous | NM_005035 | c.G178A | p.V60M |
| 347 | LUNG | ABCA7 | 19 | 1041843 | 1041843 | C | A | nonsynonymous | NM_019112 | c.C174A | p.N58K |
| 348 | LUNG | CCDC180 | 9 | 100092968 | 100092968 | - | GAGGAG | inframe | NM_020893 | c.2325_2326insGAGGAG | p.E775delinsEEE |
| 348 | LUNG | NBEAL2 | 3 | 47030604 | 47030604 | G | A | nonsynonymous | NM_015175 | c.G299A | p.G100D |
| 348 | LUNG | TBC1D28 | 17 | 18544392 | 18544392 | T | C | nonsynonymous | NM_001039397 | c.A101G | p.D34G |
| 348 | LUNG | CYP4F2 | 19 | 15989730 | 15989730 | T | C | nonsynonymous | NM_001082 | c.A1414G | p.T472A |
| 349 | LUNG | MUC4 | 3 | 195505753 | 195505753 | A | G | nonsynonymous | NM_018406 | c.T12698C | p.V4233A |
| 349 | LUNG | PPP1R12A | 12 | 80169711 | 80169711 | A | T | stoploss | NM_001244992 | c.T2923A | p.X975K,PPP1R12A |
| 349 | LUNG | SLC5A4 | 22 | 32643460 | 32643460 | C | A | stopgain | NM_014227 | c.G415T | p.E139X |
| 349 | LUNG | TCEANC | X | 13681679 | 13681679 | G | T | nonsynonymous | NM_001297563 | c.G1052T | p.W351L |
| 34 | BLOOD | NEB | 2 | 152424933 | 152424933 | - | A | splicing |  |  |  |
| 34 | BLOOD | MAP3K1 | 5 | 56177849 | 56177851 | CAA | - | inframe | NM_005921 | c.2822_2824del | p.941_942del |
| 34 | BLOOD | LYST | 1 | 235970023 | 235970023 | C | T | nonsynonymous | NM_000081 | c.G2413A | p.E805K |
| 34 | BLOOD | CDSN | 6 | 31084964 | 31084964 | T | C | nonsynonymous | NM_001264 | c.A428G | p.N143S |
| 34 | BLOOD | KIAA1147 | 7 | 141366203 | 141366203 | A | G | nonsynonymous | NM_001080392 | c.T704C | p.M235T |
| 34 | BLOOD | TMEM26 | 10 | 63195969 | 63195969 | C | T | nonsynonymous | NM_178505 | c.G229A | p.V77I |
| 34 | BLOOD | WDR66 | 12 | 122359408 | 122359408 | G | A | nonsynonymous | NM_144668 | c.G197A | p.G66E |
| 34 | BLOOD | CDC27 | 17 | 45234707 | 45234707 | T | A | nonsynonymous | NM_001256 | c.A519T | p.L173F |
| 34 | BLOOD | ANKRD30B | 18 | 14779986 | 14779986 | G | A | nonsynonymous | NM_001145029 | c.G1448A | p.R483Q |
| 34 | BLOOD | RBMX | X | 135956506 | 135956506 | C | G | nonsynonymous | NM_002139 | c.G971C | p.R324P |
| 34 | BLOOD | RBMX | X | 135960119 | 135960119 | C | T | nonsynonymous | NM_002139 | c.G343A | p.G115R |
| 350 | LUNG | CAMK2B | 7 | 44268438 | 44268438 | - | G | frameshift | NM_001220 | c.1424dupC | p.P475fs |
| 350 | LUNG | HRAS | 11 | 533357 | 533357 | C | G | nonsynonymous | NM_176795 | c.G452C | p.G151A |
| 350 | LUNG | GALNT18 | 11 | 11470353 | 11470353 | G | T | stopgain | NM_198516 | c.C366A | p.Y122X |
| 350 | LUNG | TUBB6 | 18 | 12308320 | 12308320 | G | T | nonsynonymous | NM_001303525 | c.G29T | p.G10V |
| 351 | LUNG | ITIH3 | 3 | 52834608 | 52834608 | G | A | nonsynonymous | NM_002217 | c.G1130A | p.R377Q |
| 351 | LUNG | TFAP2A | 6 | 10410466 | 10410466 | T | G | nonsynonymous | NM_003220 | c.A148C | p.N50H |
| 351 | LUNG | RPAP1 | 15 | 41819466 | 41819466 | G | A | nonsynonymous | NM_015540 | c.C1645T | p.R549C |
| 351 | LUNG | GOLGA6L10 | 15 | 82635194 | 82635194 | T | C | nonsynonymous | NM_001164465 | c.A1505G | p.E502G |
| 351 | LUNG | ARSD | X | 2836041 | 2836041 | A | T | nonsynonymous | NM_001669 | c.T667A | p.F223I |
| 351 | LUNG | ARSD | X | 2836047 | 2836047 | C | T | nonsynonymous | NM_001669 | c.G661A | p.G221S |
| 352 | LUNG | GALNS | 16 | 88923199 | 88923199 | C | A | nonsynonymous | NM_000512 | c.G87T | p.Q29H |
| 352 | LUNG | CCDC144NL | 17 | 20769896 | 20769896 | G | T | nonsynonymous | NM_001004306 | c.C536A | p.T179N |
| 352 | LUNG | CCDC144NL | 17 | 20769899 | 20769899 | G | T | stopgain | NM_001004306 | c.C533A | p.S178X |
| 353 | BLOOD | KCNN2 | 5 | 113698631 | 113698631 | - | GCC | inframe | NM_021614 | c.159_160insGCC | p.A53delinsAA |
| 353 | BLOOD | CD3EAP | 19 | 45912490 | 45912492 | AAG | - | inframe | NM_012099 | c.1264_1266del | p.422_422del |
| 353 | BLOOD | FCGBP | 19 | 40392585 | 40392585 | T | G | nonsynonymous | NM_003890 | c.A7919C | p.E2640A |
| 354 | BLOOD | FAM50B | 6 | 3850479 | 3850479 | A | T | nonsynonymous | NM_012135 | c.A434T | p.N145I |
| 354 | BLOOD | ZNF516 | 18 | 74154244 | 74154244 | G | T | nonsynonymous | NM_014643 | c.C767A | p.A256D |
| 354 | BLOOD | SAMD10 | 20 | 62610741 | 62610741 | C | G | nonsynonymous | NM_080621 | c.G80C | p.R27P |
| 355 | BLOOD | PWWP2B | 10 | 134218293 | 134218293 | A | C | nonsynonymous | NM_138499 | c.A289C | p.T97P |
| 356 | BLOOD | DNAH1 | 3 | 52423487 | 52423487 | G | T | nonsynonymous | NM_015512 | c.G9506T | p.R3169L |
| 356 | BLOOD | SLC27A2 | 15 | 50489846 | 50489846 | T | C | nonsynonymous | NM_003645 | c.T628C | p.W210R |
| 356 | BLOOD | LRFN3 | 19 | 36435631 | 36435631 | C | T | nonsynonymous | NM_024509 | c.C1597T | p.P533S |
| 356 | BLOOD | ZNF524 | 19 | 56114005 | 56114005 | C | A | nonsynonymous | NM_153219 | c.C527A | p.P176H |
| 356 | BLOOD | ARSD | X | 2836041 | 2836041 | A | T | nonsynonymous | NM_001669 | c.T667A | p.F223I |
| 356 | BLOOD | ARSD | X | 2836047 | 2836047 | C | T | nonsynonymous | NM_001669 | c.G661A | p.G221S |
| 356 | BLOOD | IL9R | X | 155239827 | 155239827 | A | G | nonsynonymous | NM_002186 | c.A1319G | p.N440S |
| 357 | BLOOD | SNAPC4 | 9 | 139277995 | 139277997 | GCT | - | inframe | NM_003086 | c.1624_1626del | p.542_542del |
| 357 | BLOOD | MUC21 | 6 | 30955089 | 30955089 | G | C | nonsynonymous | NM_001010909 | c.G1137C | p.E379D |
| 358 | BLOOD | IRF5 | 7 | 128587352 | 128587381 | ACTCTGCAGCCGCCCACTCTGCGGCCGCCT | - | inframe | NM_001098630 | c.502_531del | p.168_177del |
| 358 | BLOOD | KRTAP5-5 | 11 | 1651191 | 1651199 | GGCTGTGGA | - | inframe | NM_001001480 | c.121_129del | p.41_43del |
| 358 | BLOOD | GOLGA8R | 15 | 30700168 | 30700168 | - | TTG | inframe | NM_001282484 | c.813_814insCAA | p.D272delinsQD |
| 358 | BLOOD | PDK4 | 7 | 95216415 | 95216415 | C | A | nonsynonymous | NM_002612 | c.G1002T | p.L334F |
| 359 | BLOOD | WDR66 | 12 | 122359397 | 122359397 | - | GAGGAGGAGGAGAAA | inframe | NM_144668 | c.186_187insGAGGAGGAGGAGAAA | p.G62delinsGEEEEK |
| 360 | BLOOD | NCOR1 | 17 | 16097870 | 16097870 | C | A | nonsynonymous | NM_006311 | c.G14T | p.G5V |
| 361 | BLOOD | AIM1L | 1 | 26671690 | 26671690 | A | G | nonsynonymous | NM_001039775 | c.T1459C | p.S487P |
| 361 | BLOOD | PIK3C2B | 1 | 204419026 | 204419026 | A | C | nonsynonymous | NM_002646 | c.T2186G | p.V729G |
| 361 | BLOOD | PDPR | 16 | 70182390 | 70182390 | T | G | nonsynonymous | NM_017990 | c.T1986G | p.N662K |
| 362 | BLOOD | NPIPA5 | 16 | 15457598 | 15457598 | G | A | nonsynonymous | NM_001277325 | c.C971T | p.A324V |
| 363 | BLOOD | PRDM2 | 1 | 14105121 | 14105121 | - | GAA | inframe | NM_012231 | c.831_832insGAA | p.D277delinsDE |
| 363 | BLOOD | AHCTF1 | 1 | 247004295 | 247004295 | G | T | nonsynonymous | NM_015446 | c.C6641A | p.T2214K |
| 363 | BLOOD | GFPT2 | 5 | 179740856 | 179740856 | C | A | nonsynonymous | NM_005110 | c.G1382T | p.C461F |
| 363 | BLOOD | CHD3 | 17 | 7796794 | 7796794 | A | C | nonsynonymous | NM_001005273 | c.A700C | p.I234L |
| 363 | BLOOD | CDH19 | 18 | 64239432 | 64239432 | A | T | nonsynonymous | NM_001271028 | c.T10A | p.Y4N |
| 363 | BLOOD | RHPN2 | 19 | 33490566 | 33490566 | T | C | nonsynonymous | NM_033103 | c.A1151G | p.Q384R |
| 363 | BLOOD | NPHS1 | 19 | 36336398 | 36336398 | C | G | nonsynonymous | NM_004646 | c.G1802C | p.G601A |
| 364 | BLOOD | HRCT1 | 9 | 35906583 | 35906583 | - | CCA | inframe | NM_001039792 | c.299_300insCCA | p.L100delinsLH |
| 364 | BLOOD | DCHS1 | 11 | 6661600 | 6661600 | G | T | nonsynonymous | NM_003737 | c.C1245A | p.S415R |
| 365 | BLOOD | EXOC6B | 2 | 72411232 | 72411232 | G | T | nonsynonymous | NM_015189 | c.C2281A | p.P761T |
| 365 | BLOOD | BTBD1 | 15 | 83735746 | 83735746 | G | A | nonsynonymous | NM_025238 | c.C158T | p.A53V |
| 365 | BLOOD | AKAP17A | X | 1719897 | 1719897 | C | G | nonsynonymous | NM_005088 | c.C1498G | p.P500A |
| 366 | BLOOD | CCDC180 | 9 | 100092968 | 100092968 | - | GAGGAG | inframe | NM_020893 | c.2325_2326insGAGGAG | p.E775delinsEEE |
| 366 | BLOOD | TXLNA | 1 | 32653720 | 32653720 | C | A | nonsynonymous | NM_175852 | c.C763A | p.L255I |
| 366 | BLOOD | NTRK1 | 1 | 156830892 | 156830892 | C | A | nonsynonymous | NM_002529 | c.C166A | p.L56M |
| 366 | BLOOD | SSPO | 7 | 149488591 | 149488591 | G | T | nonsynonymous | NM_198455 | c.G5042T | p.R1681M |
| 366 | BLOOD | MAP4K5 | 14 | 50911878 | 50911878 | G | A | nonsynonymous | NM_006575 | c.C1220T | p.P407L |
| 366 | BLOOD | TBX21 | 17 | 45820007 | 45820007 | G | A | nonsynonymous | NM_013351 | c.G523A | p.A175T |
| 366 | BLOOD | ANKRD24 | 19 | 4186423 | 4186423 | A | C | nonsynonymous | NM_133475 | c.A1C | p.M1L |
| 368 | BLOOD | GOLGA2 | 9 | 131020796 | 131020798 | CCT | - | inframe | NM_004486 | c.2144_2146del | p.715_716del |
| 368 | BLOOD | FAM90A1 | 12 | 8374781 | 8374781 | - | ACG | inframe | NM_018088 | c.1031_1032insCGT | p.T344delinsTV |
| 368 | BLOOD | SLC25A29 | 14 | 100758891 | 100758892 | TT | - | frameshift | NM_001039355 | c.640_641del | p.K214fs |
| 368 | BLOOD | PRAMEF4 | 1 | 12943200 | 12943200 | A | G | nonsynonymous | NM_001009611 | c.T16C | p.W6R |
| 368 | BLOOD | UBXN11 | 1 | 26608879 | 26608879 | C | T | nonsynonymous | NM_183008 | c.G1474A | p.G492S |
| 368 | BLOOD | ECEL1 | 2 | 233345860 | 233345860 | C | A | nonsynonymous | NM_004826 | c.G1996T | p.G666W |
| 368 | BLOOD | UROC1 | 3 | 126214943 | 126214943 | A | G | nonsynonymous | NM_144639 | c.T1447C | p.S483P |
| 368 | BLOOD | DND1 | 5 | 140052430 | 140052430 | G | T | nonsynonymous | NM_194249 | c.C204A | p.D68E |
| 368 | BLOOD | PDZD8 | 10 | 119134621 | 119134621 | G | T | nonsynonymous | NM_173791 | c.C118A | p.R40S |
| 368 | BLOOD | TMEM8A | 16 | 427710 | 427710 | C | A | nonsynonymous | NM_021259 | c.G260T | p.R87L |
| 368 | BLOOD | APC2 | 19 | 1468950 | 1468950 | C | A | nonsynonymous | NM_005883 | c.C5650A | p.Q1884K |
| 368 | BLOOD | TNFRSF6B | 20 | 62328263 | 62328263 | T | G | nonsynonymous | NM_003823 | c.T143G | p.V48G |
| 369 | BLOOD | PIK3R4 | 3 | 130447530 | 130447530 | - | A | splicing |  |  |  |
| 369 | BLOOD | KRT4 | 12 | 53207583 | 53207583 | - | CACCAAAGCCACCAGTGCCGAAACC | frameshift | NM_002272 | c.259_260insGGTTTCGGCACTGGTGGCTTTGGTG | p.G87fs |
| 369 | BLOOD | CTAGE5 | 14 | 39784005 | 39784006 | TA | - | splicing |  |  |  |
| 369 | BLOOD | KIR2DS4 | 19 | 55350963 | 55350963 | - | CCC | inframe | NM_001281971 | c.451_452insCCC | p.S151delinsSP,KIR2DS4 |
| 369 | BLOOD | IDUA | 4 | 996869 | 996869 | C | A | nonsynonymous | NM_000203 | c.C1448A | p.P483H |
| 369 | BLOOD | TRPM5 | 11 | 2439423 | 2439423 | C | A | stopgain | NM_014555 | c.G880T | p.E294X |
| 370 | BLOOD | KRT4 | 12 | 53207583 | 53207583 | - | CACCAAAGCCACCAGTGCCGAAACC | frameshift | NM_002272 | c.259_260insGGTTTCGGCACTGGTGGCTTTGGTG | p.G87fs |
| 370 | BLOOD | CTAGE5 | 14 | 39784005 | 39784006 | TA | - | splicing |  |  |  |
| 370 | BLOOD | KCNMB2 | 3 | 178525219 | 178525219 | C | T | nonsynonymous | NM_001278911 | c.C22T | p.R8W |
| 370 | BLOOD | PABPC1 | 8 | 101721812 | 101721812 | G | A | nonsynonymous | NM_002568 | c.C1120T | p.R374C |
| 370 | BLOOD | PABPC1 | 8 | 101721817 | 101721817 | T | C | nonsynonymous | NM_002568 | c.A1115G | p.E372G |
| 370 | BLOOD | PABPC1 | 8 | 101721839 | 101721839 | C | A | nonsynonymous | NM_002568 | c.G1093T | p.V365L |
| 370 | BLOOD | SPATA31D1 | 9 | 84610091 | 84610091 | G | T | nonsynonymous | NM_001001670 | c.G4706T | p.G1569V |
| 370 | BLOOD | THAP8 | 19 | 36530430 | 36530430 | G | A | nonsynonymous | NM_152658 | c.C467T | p.T156I |
| 370 | BLOOD | SLC8A2 | 19 | 47941155 | 47941155 | C | A | nonsynonymous | NM_015063 | c.G1961T | p.G654V |
| 371 | BLOOD | FAM90A1 | 12 | 8374781 | 8374781 | - | ACG | inframe | NM_018088 | c.1031_1032insCGT | p.T344delinsTV |
| 371 | BLOOD | NETO1 | 18 | 70423384 | 70423384 | - | A | splicing |  |  |  |
| 371 | BLOOD | HDGFRP2 | 19 | 4499633 | 4499647 | AGCTGGCCGGGGAGG | - | frameshift | NM_001001520 | c.1721_1722del | p.K574fs |
| 371 | BLOOD | NSUN5 | 7 | 72721702 | 72721702 | C | T | nonsynonymous | NM_018044 | c.G269A | p.R90Q |
| 371 | BLOOD | TMEM245 | 9 | 111853375 | 111853375 | G | T | nonsynonymous | NM_032012 | c.C977A | p.P326H |
| 371 | BLOOD | COL27A1 | 9 | 117044783 | 117044783 | G | T | nonsynonymous | NM_032888 | c.G3818T | p.G1273V |
| 371 | BLOOD | TAS2R31 | 12 | 11183722 | 11183722 | A | C | nonsynonymous | NM_176885 | c.T213G | p.F71L |
| 371 | BLOOD | PLIN4 | 19 | 4511746 | 4511746 | A | T | nonsynonymous | NM_001080400 | c.T2184A | p.N728K |
| 372 | BLOOD | SPEN | 1 | 16262465 | 16262465 | A | C | nonsynonymous | NM_015001 | c.A9730C | p.T3244P |
| 372 | BLOOD | OBSCN | 1 | 228503812 | 228503812 | C | A | nonsynonymous | NM_001098623 | c.C13277A | p.T4426K |
| 372 | BLOOD | TCP10 | 6 | 167786686 | 167786686 | C | T | nonsynonymous | NM_004610 | c.G952A | p.A318T |
| 372 | BLOOD | MRPS28 | 8 | 80942299 | 80942299 | A | G | nonsynonymous | NM_014018 | c.T185C | p.L62P |
| 372 | BLOOD | IREB2 | 15 | 78762889 | 78762889 | A | T | nonsynonymous | NM_004136 | c.A645T | p.L215F |
| 372 | BLOOD | POTEC | 18 | 14542949 | 14542949 | T | C | nonsynonymous | NM_001137671 | c.A197G | p.H66R |
| 372 | BLOOD | PLIN4 | 19 | 4511746 | 4511746 | A | T | nonsynonymous | NM_001080400 | c.T2184A | p.N728K |
| 372 | BLOOD | MUC16 | 19 | 8966706 | 8966706 | C | T | nonsynonymous | NM_024690 | c.G43247A | p.R14416Q |
| 373 | BLOOD | HDGFRP2 | 19 | 4499633 | 4499647 | AGCTGGCCGGGGAGG | - | frameshift | NM_001001520 | c.1721_1722del | p.K574fs |
| 373 | BLOOD | BCAT1 | 12 | 24985659 | 24985659 | C | T | nonsynonymous | NM_005504 | c.G1042A | p.E348K |
| 373 | BLOOD | ANKRD30B | 18 | 14779986 | 14779986 | G | A | nonsynonymous | NM_001145029 | c.G1448A | p.R483Q |
| 373 | BLOOD | ANKRD30B | 18 | 14780019 | 14780019 | G | T | nonsynonymous | NM_001145029 | c.G1481T | p.G494V |
| 374 | BLOOD | SEC16A | 9 | 139348749 | 139348749 | G | A | nonsynonymous | NM_014866 | c.C5888T | p.A1963V |
| 374 | BLOOD | RTBDN | 19 | 12936693 | 12936693 | C | T | nonsynonymous | NM_001080997 | c.G517A | p.V173M |
| 375 | BLOOD | PCDH12 | 5 | 141324955 | 141324955 | - | CTGCTGCTG | inframe | NM_016580 | c.3545_3546insCAGCAGCAG | p.R1182delinsSSSR |
| 375 | BLOOD | FOXE1 | 9 | 100616701 | 100616706 | GCCGCC | - | inframe | NM_004473 | c.505_510del | p.169_170del |
| 375 | BLOOD | RBBP6 | 16 | 24564880 | 24564881 | TA | - | splicing |  |  |  |
| 375 | BLOOD | KRTAP4-3 | 17 | 39324139 | 39324139 | - | GCAGCAGGTGGTCAG | inframe | NM_033187 | c.285_286insCTGACCACCTGCTGC | p.R96delinsLTTCCR |
| 375 | BLOOD | GADD45GIP1 | 19 | 13067838 | 13067844 | ACGCGCG | - | frameshift | NM_052850 | c.183_189del | p.F61fs |
| 375 | BLOOD | MSH3 | 5 | 79950736 | 79950736 | C | G | nonsynonymous | NM_002439 | c.C190G | p.P64A |
| 375 | BLOOD | TMEM161B | 5 | 87502292 | 87502292 | G | A | nonsynonymous | NM_153354 | c.C623T | p.A208V |
| 376 | BLOOD | NCOR2 | 12 | 124887058 | 124887058 | - | GCT | inframe | NM_006312 | c.1531_1532insAGC | p.P511delinsQP |
| 376 | BLOOD | RIN2 | 20 | 19941419 | 19941419 | C | T | nonsynonymous | NM_001242581 | c.C574T | p.P192S |
| 376 | BLOOD | ARSD | X | 2833638 | 2833638 | C | T | nonsynonymous | NM_001669 | c.G959A | p.G320D |
| 377 | BLOOD | SYNE2 | 14 | 64464069 | 64464069 | C | T | nonsynonymous | NM_015180 | c.C3203T | p.A1068V |
| 377 | BLOOD | ZBTB33 | X | 119388777 | 119388777 | T | C | nonsynonymous | NM_006777 | c.T1507C | p.Y503H |
| 378 | BLOOD | MARK1 | 1 | 220826682 | 220826682 | A | G | nonsynonymous | NM_018650 | c.A1976G | p.K659R |
| 378 | BLOOD | TTC13 | 1 | 231059641 | 231059641 | T | C | nonsynonymous | NM_024525 | c.A1760G | p.Q587R |
| 378 | BLOOD | PRIM1 | 12 | 57132236 | 57132236 | C | T | nonsynonymous | NM_000946 | c.G1126A | p.V376I |
| 379 | BLOOD | TRAK1 | 3 | 42251577 | 42251577 | - | GGA | inframe | NM_014965 | c.1889_1890insGGA | p.T630delinsTE |
| 379 | BLOOD | UBTFL1 | 11 | 89819888 | 89819888 | - | T | frameshift | NM_001143975 | c.772dupT | p.R257fs |
| 379 | BLOOD | FAM90A1 | 12 | 8374781 | 8374781 | - | ACG | inframe | NM_018088 | c.1031_1032insCGT | p.T344delinsTV |
| 379 | BLOOD | GSE1 | 16 | 85690001 | 85690006 | GAGCGT | - | inframe | NM_014615 | c.1042_1047del | p.348_349del |
| 379 | BLOOD | MUC4 | 3 | 195505836 | 195505836 | G | C | nonsynonymous | NM_018406 | c.C12615G | p.H4205Q |
| 379 | BLOOD | NAAA | 4 | 76846923 | 76846923 | G | A | nonsynonymous | NM_014435 | c.C631T | p.R211W |
| 379 | BLOOD | CCNA2 | 4 | 122744621 | 122744621 | A | G | nonsynonymous | NM_001237 | c.T163C | p.S55P |
| 379 | BLOOD | SMARCA2 | 9 | 2039784 | 2039784 | A | C | nonsynonymous | NM_001289396 | c.A674C | p.Q225P |
| 379 | BLOOD | GTF3C5 | 9 | 135929816 | 135929816 | C | T | nonsynonymous | NM_012087 | c.C1010T | p.P337L |
| 379 | BLOOD | KIAA1549L | 11 | 33682557 | 33682557 | T | A | nonsynonymous | NM_012194 | c.T5265A | p.D1755E |
| 379 | BLOOD | UBTFL1 | 11 | 89819885 | 89819885 | A | T | nonsynonymous | NM_001143975 | c.A768T | p.R256S |
| 379 | BLOOD | CDC27 | 17 | 45234417 | 45234417 | A | G | nonsynonymous | NM_001256 | c.T704C | p.I235T |
| 379 | BLOOD | PLIN4 | 19 | 4511746 | 4511746 | A | T | nonsynonymous | NM_001080400 | c.T2184A | p.N728K |
| 379 | BLOOD | CYP4F2 | 19 | 15989730 | 15989730 | T | C | nonsynonymous | NM_001082 | c.A1414G | p.T472A |
| 379 | BLOOD | PRR12 | 19 | 50098268 | 50098268 | C | A | nonsynonymous | NM_020719 | c.C676A | p.P226T |
| 380 | BLOOD | NCOR2 | 12 | 124887058 | 124887058 | - | GCT | inframe | NM_006312 | c.1531_1532insAGC | p.P511delinsQP |
| 380 | BLOOD | SH3BGR | 21 | 40883671 | 40883671 | - | AGA | inframe | NM_007341 | c.689_690insAGA | p.G230delinsGE |
| 380 | BLOOD | PLAC4 | 21 | 42551272 | 42551272 | C | - | frameshift | NM_182832 | c.284delG | p.G95fs |
| 380 | BLOOD | CENPF | 1 | 214815304 | 214815304 | C | T | nonsynonymous | NM_016343 | c.C3623T | p.A1208V |
| 380 | BLOOD | FMN2 | 1 | 240371360 | 240371360 | C | T | nonsynonymous | NM_001305424 | c.C3260T | p.A1087V |
| 380 | BLOOD | TBC1D5 | 3 | 17470012 | 17470012 | C | T | splicing |  |  |  |
| 380 | BLOOD | MARVELD2 | 5 | 68715388 | 68715388 | C | T | nonsynonymous | NM_001038603 | c.C176T | p.P59L |
| 380 | BLOOD | PRRC1 | 5 | 126887585 | 126887585 | C | T | nonsynonymous | NM_130809 | c.C1315T | p.R439C |
| 380 | BLOOD | ADAMTS2 | 5 | 178563002 | 178563002 | C | T | nonsynonymous | NM_014244 | c.G1993A | p.G665R |
| 380 | BLOOD | MUC3A | 7 | 100549697 | 100549697 | A | G | nonsynonymous | NM_005960 | c.A278G | p.N93S |
| 380 | BLOOD | MUC3A | 7 | 100549703 | 100549703 | C | T | nonsynonymous | NM_005960 | c.C284T | p.P95L |
| 380 | BLOOD | MUC3A | 7 | 100552018 | 100552018 | T | G | nonsynonymous | NM_005960 | c.T2599G | p.S867A |
| 380 | BLOOD | BNC2 | 9 | 16419498 | 16419498 | T | C | nonsynonymous | NM_017637 | c.A2789G | p.D930G |
| 380 | BLOOD | TRPM6 | 9 | 77411729 | 77411729 | C | G | nonsynonymous | NM_017662 | c.G2319C | p.Q773H |
| 380 | BLOOD | KIAA1958 | 9 | 115421637 | 115421637 | C | G | nonsynonymous | NM_133465 | c.C1439G | p.A480G |
| 380 | BLOOD | CNTRL | 9 | 123850642 | 123850642 | C | A | nonsynonymous | NM_007018 | c.C38A | p.A13E |
| 380 | BLOOD | MUC5B | 11 | 1267972 | 1267972 | A | G | nonsynonymous | NM_002458 | c.A9862G | p.R3288G |
| 380 | BLOOD | ABCC8 | 11 | 17464808 | 17464808 | T | C | nonsynonymous | NM_000352 | c.A1384G | p.I462V |
| 380 | BLOOD | MON2 | 12 | 62979252 | 62979252 | A | C | nonsynonymous | NM_001278472 | c.A4662C | p.E1554D |
| 380 | BLOOD | NAA25 | 12 | 112481511 | 112481511 | C | T | nonsynonymous | NM_024953 | c.G2168A | p.R723Q |
| 380 | BLOOD | HECTD4 | 12 | 112605167 | 112605167 | T | C | nonsynonymous | NM_001109662 | c.A12086G | p.Q4029R |
| 380 | BLOOD | KCNG4 | 16 | 84256245 | 84256245 | C | T | nonsynonymous | NM_172347 | c.G1138A | p.V380M |
| 380 | BLOOD | SARM1 | 17 | 26708733 | 26708733 | C | A | nonsynonymous | NM_015077 | c.C979A | p.Q327K |
| 380 | BLOOD | MPP2 | 17 | 41981843 | 41981843 | G | A | nonsynonymous | NM_001278370 | c.C121T | p.L41F |
| 380 | BLOOD | KCNH6 | 17 | 61615606 | 61615606 | A | C | nonsynonymous | NM_030779 | c.A1682C | p.N561T |
| 380 | BLOOD | CEP112 | 17 | 64049981 | 64049981 | G | C | nonsynonymous | NM_001199165 | c.C1091G | p.A364G |
| 380 | BLOOD | DNAH17 | 17 | 76472719 | 76472719 | C | T | nonsynonymous | NM_173628 | c.G8089A | p.V2697I |
| 380 | BLOOD | SLC35G4 | 18 | 11609994 | 11609994 | C | T | nonsynonymous | NM_001282300 | c.C400T | p.R134C |
| 380 | BLOOD | SLC35G4 | 18 | 11610001 | 11610001 | A | G | nonsynonymous | NM_001282300 | c.A407G | p.H136R |
| 380 | BLOOD | ELP2 | 18 | 33722921 | 33722921 | A | G | nonsynonymous | NM_018255 | c.A788G | p.E263G |
| 380 | BLOOD | MADCAM1 | 19 | 501762 | 501762 | A | C | nonsynonymous | NM_130760 | c.A761C | p.Q254P |
| 380 | BLOOD | PLVAP | 19 | 17476933 | 17476933 | G | T | nonsynonymous | NM_031310 | c.C441A | p.F147L |
| 380 | BLOOD | MYH7B | 20 | 33574773 | 33574773 | A | G | nonsynonymous | NM_020884 | c.A1115G | p.E372G |
| 380 | BLOOD | SULF2 | 20 | 46318901 | 46318901 | A | C | nonsynonymous | NM_018837 | c.T706G | p.S236A |
| 380 | BLOOD | PLAC4 | 21 | 42551245 | 42551245 | A | G | nonsynonymous | NM_182832 | c.T311C | p.L104P |
| 380 | BLOOD | PLAC4 | 21 | 42551270 | 42551270 | A | G | nonsynonymous | NM_182832 | c.T286C | p.Y96H |
| 380 | BLOOD | PKNOX1 | 21 | 44438248 | 44438248 | A | C | nonsynonymous | NM_004571 | c.A628C | p.T210P |
| 380 | BLOOD | KRTAP10-10 | 21 | 46057795 | 46057795 | C | T | nonsynonymous | NM_181688 | c.C461T | p.S154F |
| 380 | BLOOD | CCT8L2 | 22 | 17072747 | 17072747 | T | C | nonsynonymous | NM_014406 | c.A694G | p.M232V |
| 380 | BLOOD | PEX26 | 22 | 18566288 | 18566288 | C | G | nonsynonymous | NM_017929 | c.C457G | p.L153V |
| 380 | BLOOD | HIRA | 22 | 19373208 | 19373208 | C | T | nonsynonymous | NM_003325 | c.G1165A | p.A389T |
| 380 | BLOOD | POLDIP3 | 22 | 43010821 | 43010821 | C | T | nonsynonymous | NM_032311 | c.G43A | p.A15T |
| 380 | BLOOD | IL3RA | X | 1497644 | 1497644 | G | C | nonsynonymous | NM_002183 | c.G967C | p.V323L |
| 380 | BLOOD | ARSD | X | 2833605 | 2833605 | C | T | stopgain | NM_001669 | c.G992A | p.W331X |
| 380 | BLOOD | ARSD | X | 2833638 | 2833638 | C | T | nonsynonymous | NM_001669 | c.G959A | p.G320D |
| 380 | BLOOD | ARSD | X | 2833643 | 2833643 | C | A | nonsynonymous | NM_001669 | c.G954T | p.Q318H |
| 381 | BLOOD | CNTNAP3B | 9 | 43861012 | 43861012 | C | T | nonsynonymous | NM_001201380 | c.C1886T | p.A629V |
| 381 | BLOOD | DOCK6 | 19 | 11364347 | 11364347 | G | C | nonsynonymous | NM_020812 | c.C100G | p.H34D |
| 382 | BLOOD | FAM90A1 | 12 | 8374781 | 8374781 | - | ACG | inframe | NM_018088 | c.1031_1032insCGT | p.T344delinsTV |
| 382 | BLOOD | NALCN | 13 | 101733910 | 101733910 | C | T | nonsynonymous | NM_052867 | c.G3853A | p.V1285I |
| 382 | BLOOD | GOLGA6L10 | 15 | 82635194 | 82635194 | T | C | nonsynonymous | NM_001164465 | c.A1505G | p.E502G |
| 382 | BLOOD | CDC27 | 17 | 45249328 | 45249328 | G | A | nonsynonymous | NM_001256 | c.C206T | p.P69L |
| 382 | BLOOD | ANKRD30B | 18 | 14779986 | 14779986 | G | A | nonsynonymous | NM_001145029 | c.G1448A | p.R483Q |
| 383 | BLOOD | FAM90A1 | 12 | 8374781 | 8374781 | - | ACG | inframe | NM_018088 | c.1031_1032insCGT | p.T344delinsTV |
| 384 | BLOOD | SASH1 | 6 | 148664242 | 148664242 | - | GAGCCC | inframe | NM_015278 | c.39_40insGAGCCC | p.P13delinsPEP |
| 384 | BLOOD | CNTNAP3B | 9 | 43849811 | 43849811 | C | A | nonsynonymous | NM_001201380 | c.C1716A | p.D572E |
| 384 | BLOOD | SLC15A3 | 11 | 60718887 | 60718887 | C | A | nonsynonymous | NM_016582 | c.G137T | p.R46L |
| 384 | BLOOD | CLUH | 17 | 2599817 | 2599817 | C | A | nonsynonymous | NM_015229 | c.G2084T | p.R695L |
| 384 | BLOOD | CDC27 | 17 | 45249328 | 45249328 | G | A | nonsynonymous | NM_001256 | c.C206T | p.P69L |
| 385 | BLOOD | FAM90A1 | 12 | 8374781 | 8374781 | - | ACG | inframe | NM_018088 | c.1031_1032insCGT | p.T344delinsTV |
| 385 | BLOOD | SV2C | 5 | 75581068 | 75581068 | C | T | nonsynonymous | NM_001297716 | c.C995T | p.S332F |
| 385 | BLOOD | DPP6 | 7 | 154587585 | 154587585 | C | A | nonsynonymous | NM_130797 | c.C1291A | p.H431N |
| 385 | BLOOD | RP1L1 | 8 | 10467637 | 10467637 | T | C | nonsynonymous | NM_178857 | c.A3971G | p.E1324G |
| 385 | BLOOD | LZTS1 | 8 | 20112355 | 20112355 | A | G | nonsynonymous | NM_021020 | c.T338C | p.L113P |
| 385 | BLOOD | MRVI1 | 11 | 10624764 | 10624764 | C | A | nonsynonymous | NM_130385 | c.G1778T | p.R593L |
| 385 | BLOOD | MS4A15 | 11 | 60540907 | 60540907 | G | T | nonsynonymous | NM_001098835 | c.G448T | p.A150S |
| 385 | BLOOD | KRTAP5-11 | 11 | 71293567 | 71293567 | C | T | nonsynonymous | NM_001005405 | c.G317A | p.C106Y |
| 385 | BLOOD | CACNA1H | 16 | 1263819 | 1263819 | C | T | nonsynonymous | NM_021098 | c.C4817T | p.T1606M |
| 385 | BLOOD | MYO18A | 17 | 27424239 | 27424239 | A | C | splicing |  |  |  |
| 385 | BLOOD | ZNF470 | 19 | 57088420 | 57088420 | G | A | nonsynonymous | NM_001001668 | c.G623A | p.S208N |
| 386 | LUNG | POU4F2 | 4 | 147560458 | 147560466 | GGCGGCGGC | - | inframe | NM_004575 | c.166_174del | p.56_58del |
| 386 | LUNG | SLCO2A1 | 3 | 133748570 | 133748570 | G | A | nonsynonymous | NM_005630 | c.C77T | p.S26L |
| 386 | LUNG | PKD1 | 16 | 2158673 | 2158673 | C | A | nonsynonymous | NM_001009944 | c.G6495T | p.Q2165H |
| 386 | LUNG | GAGE12J | X | 49179711 | 49179711 | A | T | nonsynonymous | NM_001098406 | c.A39T | p.R13S |
| 386 | LUNG | GAGE12J | X | 49179719 | 49179719 | C | G | nonsynonymous | NM_001098406 | c.C47G | p.P16R |
| 387 | LUNG | MADCAM1 | 19 | 501786 | 501786 | C | A | nonsynonymous | NM_130760 | c.C785A | p.P262Q |
| 389 | LUNG | DDX20 | 1 | 112308587 | 112308587 | A | T | nonsynonymous | NM_007204 | c.A1541T | p.Q514L |
| 389 | LUNG | ARG1 | 6 | 131904616 | 131904616 | G | A | nonsynonymous | NM_000045 | c.G787A | p.E263K |
| 389 | LUNG | XPO7 | 8 | 21851875 | 21851875 | G | A | nonsynonymous | NM_015024 | c.G2150A | p.R717Q |
| 389 | LUNG | CEBPD | 8 | 48650498 | 48650498 | C | T | nonsynonymous | NM_005195 | c.G185A | p.S62N |
| 389 | LUNG | GRIN3B | 19 | 1008645 | 1008645 | C | A | nonsynonymous | NM_138690 | c.C2495A | p.A832E |
| 389 | LUNG | PLIN4 | 19 | 4511746 | 4511746 | A | T | nonsynonymous | NM_001080400 | c.T2184A | p.N728K |
| 389 | LUNG | CDC37 | 19 | 10506863 | 10506863 | A | G | nonsynonymous | NM_007065 | c.T119C | p.M40T |
| 390 | BLOOD | POU4F2 | 4 | 147560457 | 147560457 | - | GGC | inframe | NM_004575 | c.165_166insGGC | p.G55delinsGG |
| 390 | BLOOD | TMEM102 | 17 | 7339495 | 7339515 | TCCGTTCCCTTTTTTTAGGTC | - | inframe | NM_178518 | c.215_217del | p.72_73del |
| 390 | BLOOD | HSPBP1 | 19 | 55790886 | 55790886 | - | GCCGCCGCC | inframe | NM_001130106 | c.90_91insGGCGGCGGC | p.S31delinsGGGS |
| 390 | BLOOD | SRRM1 | 1 | 24979487 | 24979487 | C | T | nonsynonymous | NM_005839 | c.C1004T | p.P335L |
| 390 | BLOOD | DENND1A | 9 | 126144390 | 126144390 | G | T | nonsynonymous | NM_020946 | c.C2351A | p.A784D |
| 390 | BLOOD | PAN3 | 13 | 28713030 | 28713030 | T | C | nonsynonymous | NM_175854 | c.T236C | p.L79P |
| 390 | BLOOD | LDHD | 16 | 75149457 | 75149457 | C | G | nonsynonymous | NM_194436 | c.G174C | p.E58D |
| 390 | BLOOD | SLC16A2 | X | 73641569 | 73641569 | T | C | nonsynonymous | NM_006517 | c.T97C | p.S33P |
| 390 | BLOOD | MAGEC3 | X | 140967165 | 140967165 | T | C | nonsynonymous | NM_138702 | c.T463C | p.S155P |
| 390 | BLOOD | PLXNB3 | X | 153035798 | 153035798 | G | A | nonsynonymous | NM_005393 | c.G1792A | p.V598I |
| 390 | BLOOD | CTAG2 | X | 153881525 | 153881525 | G | C | nonsynonymous | NM_020994 | c.C265G | p.Q89E |
| 391 | BLOOD | DCP1B | 12 | 2062323 | 2062323 | - | TGC | inframe | NM_152640 | c.782_783insGCA | p.Q261delinsQQ |
| 391 | BLOOD | GDF15 | 19 | 18499305 | 18499305 | - | CGT | inframe | NM_004864 | c.487_488insCGT | p.P163delinsPS |
| 391 | BLOOD | ZNF283 | 19 | 44351167 | 44351172 | GGAGAT | - | frameshift | NM_001297752 |  |  |
| 391 | BLOOD | CIDEC | 3 | 9912149 | 9912149 | G | T | nonsynonymous | NM_001199551 | c.C217A | p.P73T |
| 391 | BLOOD | WWC2 | 4 | 184186899 | 184186899 | G | A | nonsynonymous | NM_024949 | c.G2098A | p.A700T |
| 391 | BLOOD | SORCS1 | 10 | 108439437 | 108439437 | C | T | nonsynonymous | NM_001013031 | c.G1616A | p.G539E |
| 391 | BLOOD | TJP3 | 19 | 3740624 | 3740624 | A | G | nonsynonymous | NM_001267560 | c.A1706G | p.N569S |
| 391 | BLOOD | RHPN2 | 19 | 33490566 | 33490566 | T | C | nonsynonymous | NM_033103 | c.A1151G | p.Q384R |
| 391 | BLOOD | OGFR | 20 | 61444633 | 61444633 | G | A | nonsynonymous | NM_007346 | c.G1666A | p.E556K |
| 391 | BLOOD | PPP2R3B | X | 299360 | 299360 | G | A | nonsynonymous | NM_013239 | c.C1556T | p.A519V |
| 392 | BLOOD | CCDC66 | 3 | 56650054 | 56650054 | - | CTC | inframe | NM_001141947 | c.1816_1817insCTC | p.S606delinsSP |
| 392 | BLOOD | KIAA2018 | 3 | 113376111 | 113376113 | TGC | - | inframe | NM_001009899 | c.4416_4418del | p.1472_1473del |
| 392 | BLOOD | PRSS48 | 4 | 152201018 | 152201018 | - | CAGGT | frameshift | NM_183375 | c.123_124insCAGGT | p.W41fs |
| 392 | BLOOD | HAVCR1 | 5 | 156479558 | 156479572 | TTGGAACAGTCGTCA | - | inframe | NM_001099414 | c.473_487del | p.158_163del |
| 392 | BLOOD | HLA-A | 6 | 29912029 | 29912029 | G | - | frameshift | NM_002116 | c.750delG | p.Q250fs |
| 392 | BLOOD | ARID1B | 6 | 157100396 | 157100396 | - | CGC | inframe | NM_017519 | c.1333_1334insCGC | p.A445delinsAP |
| 392 | BLOOD | OR2A14 | 7 | 143826730 | 143826732 | CTT | - | inframe | NM_001001659 | c.525_527del | p.175_176del |
| 392 | BLOOD | CCDC180 | 9 | 100092968 | 100092968 | - | GAGGAG | inframe | NM_020893 | c.2325_2326insGAGGAG | p.E775delinsEEE |
| 392 | BLOOD | YY1 | 14 | 100705788 | 100705790 | CCA | - | inframe | NM_003403 | c.207_209del | p.69_70del |
| 392 | BLOOD | PRKCSH | 19 | 11558341 | 11558346 | GAGGAG | - | inframe | NM_002743 | c.937_942del | p.313_314del |
| 392 | BLOOD | TRAF3IP1 | 2 | 239307457 | 239307457 | A | G | nonsynonymous | NM_015650 | c.A1973G | p.K658R |
| 392 | BLOOD | LTF | 3 | 46487937 | 46487937 | C | T | nonsynonymous | NM_002343 | c.G1351A | p.V451M |
| 392 | BLOOD | IL17RB | 3 | 53892824 | 53892824 | G | A | nonsynonymous | NM_018725 | c.G826A | p.V276I |
| 392 | BLOOD | C3orf56 | 3 | 126916036 | 126916036 | G | A | nonsynonymous | NM_001007534 | c.G508A | p.V170I |
| 392 | BLOOD | TMCC1 | 3 | 129546776 | 129546776 | A | G | nonsynonymous | NM_001017395 | c.T446C | p.M149T |
| 392 | BLOOD | MUC4 | 3 | 195517169 | 195517169 | T | C | nonsynonymous | NM_018406 | c.A1282G | p.I428V |
| 392 | BLOOD | FNDC9 | 5 | 156770117 | 156770117 | G | A | nonsynonymous | NM_001001343 | c.C428T | p.P143L |
| 392 | BLOOD | FOXI1 | 5 | 169535155 | 169535155 | C | T | nonsynonymous | NM_012188 | c.C677T | p.T226I |
| 392 | BLOOD | UIMC1 | 5 | 176332431 | 176332431 | C | T | nonsynonymous | NM_016290 | c.G2012A | p.R671H |
| 392 | BLOOD | SYCP2L | 6 | 10935412 | 10935412 | A | G | nonsynonymous | NM_001040274 | c.A1805G | p.Q602R |
| 392 | BLOOD | KIF13A | 6 | 17764896 | 17764896 | G | C | nonsynonymous | NM_022113 | c.C4863G | p.D1621E |
| 392 | BLOOD | HLA-A | 6 | 29910716 | 29910716 | C | G | nonsynonymous | NM_002116 | c.C256G | p.Q86E |
| 392 | BLOOD | HLA-A | 6 | 29912108 | 29912108 | G | C | nonsynonymous | NM_002116 | c.G829C | p.E277Q |
| 392 | BLOOD | CYP39A1 | 6 | 46593179 | 46593179 | T | G | nonsynonymous | NM_001278738 | c.A847C | p.I283L |
| 392 | BLOOD | CDHR3 | 7 | 105660972 | 105660972 | C | T | nonsynonymous | NM_152750 | c.C1807T | p.R603C |
| 392 | BLOOD | CTTNBP2 | 7 | 117513445 | 117513445 | C | T | nonsynonymous | NM_033427 | c.G25A | p.E9K |
| 392 | BLOOD | FLNC | 7 | 128494043 | 128494043 | T | C | nonsynonymous | NM_001458 | c.T6500C | p.M2167T |
| 392 | BLOOD | KLF9 | 9 | 73028201 | 73028201 | G | A | nonsynonymous | NM_001206 | c.C79T | p.H27Y |
| 392 | BLOOD | OR1D5 | 17 | 2966273 | 2966273 | G | A | nonsynonymous | NM_014566 | c.C629T | p.P210L |
| 392 | BLOOD | PALM | 19 | 746695 | 746695 | G | A | nonsynonymous | NM_002579 | c.G1045A | p.E349K |
| 392 | BLOOD | DPP9 | 19 | 4704192 | 4704192 | G | C | nonsynonymous | NM_139159 | c.C551G | p.A184G |
| 392 | BLOOD | ADARB1 | 21 | 46595812 | 46595812 | C | T | nonsynonymous | NM_001112 | c.C196T | p.R66C |
| 392 | BLOOD | PLCXD1 | X | 200939 | 200939 | C | T | nonsynonymous | NM_018390 | c.C85T | p.P29S |
| 392 | BLOOD | PPP2R3B | X | 322193 | 322193 | C | T | nonsynonymous | NM_013239 | c.G457A | p.A153T |
| 392 | BLOOD | ARSD | X | 2833638 | 2833638 | C | T | nonsynonymous | NM_001669 | c.G959A | p.G320D |
| 392 | BLOOD | ARSD | X | 2833643 | 2833643 | C | A | nonsynonymous | NM_001669 | c.G954T | p.Q318H |
| 392 | BLOOD | ARSD | X | 2836181 | 2836181 | A | T | nonsynonymous | NM_001669 | c.T527A | p.M176K |
| 393 | BLOOD | SPOCK1 | 5 | 136834138 | 136834138 | T | C | nonsynonymous | NM_004598 | c.A110G | p.N37S |
| 393 | BLOOD | PPP2R3B | X | 347385 | 347385 | C | A | nonsynonymous | NM_013239 | c.G42T | p.K14N |
| 394 | BLOOD | COL4A2 | 13 | 111143601 | 111143601 | A | G | nonsynonymous | NM_001846 | c.A3368G | p.E1123G |
| 394 | BLOOD | ANKRD30B | 18 | 14779986 | 14779986 | G | A | nonsynonymous | NM_001145029 | c.G1448A | p.R483Q |
| 394 | BLOOD | RTN2 | 19 | 45992677 | 45992677 | C | T | nonsynonymous | NM_005619 | c.G1168A | p.G390S |
| 394 | BLOOD | PDK3 | X | 24521595 | 24521595 | C | T | nonsynonymous | NM_005391 | c.C472T | p.R158C |
| 395 | LUNG | GXYLT1 | 12 | 42512844 | 42512844 | A | C | nonsynonymous | NM_173601 | c.T444G | p.H148Q |
| 396 | LUNG | FAM90A1 | 12 | 8374781 | 8374781 | - | ACG | inframe | NM_018088 | c.1031_1032insCGT | p.T344delinsTV |
| 397 | BLOOD | TUBGCP3 | 13 | 113201853 | 113201867 | TGGGAAAGTCGCGCG | - | inframe | NM_001286279 | c.1235_1249del | p.412_417del |
| 398 | LUNG | HDGFRP2 | 19 | 4499633 | 4499647 | AGCTGGCCGGGGAGG | - | frameshift | NM_001001520 | c.1721_1722del | p.K574fs |
| 398 | LUNG | PELP1 | 17 | 4586240 | 4586240 | T | G | nonsynonymous | NM_014389 | c.A578C | p.D193A |
| 398 | LUNG | ZNF814 | 19 | 58385748 | 58385748 | G | A | nonsynonymous | NM_001144989 | c.C1010T | p.A337V |
| 398 | LUNG | GNAS | 20 | 57429568 | 57429568 | A | C | nonsynonymous | NM_001077490 | c.A1061C | p.H354P |
| 399 | BLOOD | AUTS2 | 7 | 70255576 | 70255576 | - | CCACCA | inframe | NM_015570 | c.3374_3375insCCACCA | p.S1125delinsSHH |
| 399 | BLOOD | RSU1 | 10 | 16737156 | 16737156 | - | G | splicing | NM_015570 | c.3374_3375insCCA | p.S1125delinsSH |
| 399 | BLOOD | EP400 | 12 | 132547093 | 132547093 | - | CAG | inframe | NM_015409 | c.8181_8182insCAG | p.Q2727delinsQQ |
| 399 | BLOOD | DMKN | 19 | 36002362 | 36002412 | CTGCTGCCACCACTGCTGCCGCCACTGCTGCCGCCACTGCTGCTGCCACTG | - | inframe | NM_033317 | c.819_869del | p.273_290del |
| 399 | BLOOD | KRT1 | 12 | 53069236 | 53069256 | TAGCTGCTACCTCCGGAGCCA | - | inframe | NM_006121 | c.1656_1676del | p.552_559del |
| 399 | BLOOD | SEZ6 | 17 | 27283241 | 27283241 | C | G | nonsynonymous | NM_178860 | c.G2888C | p.R963P |
| 3 | BLOOD | DTNB | 2 | 25754440 | 25754440 | A | - | frameshift | NM_021907 | c.903delT | p.H301fs |
| 3 | BLOOD | FAM90A1 | 12 | 8374781 | 8374781 | - | ACG | inframe | NM_018088 | c.1031_1032insCGT | p.T344delinsTV |
| 3 | BLOOD | NFAT5 | 16 | 69726652 | 69726654 | CTC | - | inframe | NM_006599 | c.2870_2872del | p.957_958del |
| 3 | BLOOD | CAMSAP2 | 1 | 200817354 | 200817354 | T | C | nonsynonymous | NM_001297707 | c.T1490C | p.I497T |
| 3 | BLOOD | ANAPC1 | 2 | 112614429 | 112614429 | G | A | stopgain | NM_022662 | c.C1393T | p.Q465X |
| 3 | BLOOD | PARD3B | 2 | 205410778 | 205410778 | G | T | nonsynonymous | NM_001302769 | c.G56T | p.G19V |
| 3 | BLOOD | NEU4 | 2 | 242757910 | 242757910 | G | A | nonsynonymous | NM_001167602 | c.G991A | p.G331S |
| 3 | BLOOD | RAPGEF2 | 4 | 160260410 | 160260410 | T | A | nonsynonymous | NM_014247 | c.T1955A | p.L652Q |
| 3 | BLOOD | NCAM1 | 11 | 113078586 | 113078586 | G | A | nonsynonymous | NM_001242607 | c.G778A | p.D260N |
| 3 | BLOOD | IFT88 | 13 | 21264844 | 21264844 | A | G | nonsynonymous | NM_175605 | c.A2270G | p.D757G |
| 3 | BLOOD | ING1 | 13 | 111371947 | 111371947 | G | A | nonsynonymous | NM_005537 | c.G937A | p.D313N |
| 3 | BLOOD | ICE2 | 15 | 60741583 | 60741583 | T | A | nonsynonymous | NM_001018089 | c.A1172T | p.N391I |
| 3 | BLOOD | VCX | X | 7811645 | 7811645 | C | G | nonsynonymous | NM_013452 | c.C209G | p.A70G |
| 3 | BLOOD | HMGB3 | X | 150156313 | 150156313 | C | T | nonsynonymous | NM_001301229 | c.C529T | p.R177W |
| 400 | BLOOD | DCP1B | 12 | 2062323 | 2062323 | - | TGC | inframe | NM_152640 | c.782_783insGCA | p.Q261delinsQQ |
| 400 | BLOOD | PRMT6 | 1 | 107600460 | 107600460 | G | T | nonsynonymous | NM_018137 | c.G1123T | p.D375Y |
| 400 | BLOOD | ZNF717 | 3 | 75790880 | 75790880 | A | G | nonsynonymous | NM_001290210 | c.T65C | p.V22A |
| 400 | BLOOD | TAS2R31 | 12 | 11183697 | 11183697 | C | A | nonsynonymous | NM_176885 | c.G238T | p.V80L |
| 400 | BLOOD | TAS2R46 | 12 | 11214437 | 11214437 | T | C | nonsynonymous | NM_176887 | c.A457G | p.I153V |
| 400 | BLOOD | TAS2R46 | 12 | 11214455 | 11214455 | T | C | nonsynonymous | NM_176887 | c.A439G | p.I147V |
| 400 | BLOOD | CLIP1 | 12 | 122812697 | 122812697 | C | T | nonsynonymous | NM_002956 | c.G3013A | p.E1005K |
| 400 | BLOOD | AKAP13 | 15 | 86287035 | 86287035 | A | C | nonsynonymous | NM_007200 | c.A8371C | p.T2791P |
| 400 | BLOOD | MYLK3 | 16 | 46744689 | 46744689 | C | A | nonsynonymous | NM_182493 | c.G2127T | p.L709F |
| 400 | BLOOD | KRTAP4-11 | 17 | 39274020 | 39274020 | C | G | nonsynonymous | NM_033059 | c.G548C | p.S183T |
| 400 | BLOOD | PNKP | 19 | 50370425 | 50370425 | C | G | nonsynonymous | NM_007254 | c.G37C | p.E13Q |
| 401 | BLOOD | CCDC144NL | 17 | 20769896 | 20769896 | G | T | nonsynonymous | NM_001004306 | c.C536A | p.T179N |
| 401 | BLOOD | CCDC144NL | 17 | 20769899 | 20769899 | G | T | stopgain | NM_001004306 | c.C533A | p.S178X |
| 402 | BLOOD | CELSR2 | 1 | 109792735 | 109792735 | - | CGC | inframe | NM_001408 | c.34_35insCGC | p.T12delinsTP |
| 402 | BLOOD | BMP2K | 4 | 79792137 | 79792148 | CAGCAGCAGCAG | - | inframe | NM_198892 | c.1432_1443del | p.478_481del |
| 402 | BLOOD | MICA;MICA | 6 | 31380161 | 31380161 | - | CTGCTGCTGCT | frameshift | NM_001177519 | c.952_953insCTGCTGCTGCT | p.G318fs |
| 402 | BLOOD | TSPYL1 | 6 | 116600465 | 116600465 | - | CAC | inframe | NM_003309 | c.528_529insGTG | p.K177delinsVK |
| 402 | BLOOD | IST1 | 16 | 71956530 | 71956535 | ATGCCT | - | inframe | NM_001270975 | c.706_711del | p.236_237del |
| 402 | BLOOD | OR7G3 | 19 | 9236698 | 9236698 | - | ATGGT | frameshift | NM_001001958 | c.928_929insACCAT | p.S310fs |
| 402 | BLOOD | ZNF579 | 19 | 56089908 | 56089908 | - | CCG | inframe | NM_152600 | c.1097_1098insCGG | p.G366delinsGG |
| 402 | BLOOD | EXO1 | 1 | 242020734 | 242020734 | C | A | nonsynonymous | NM_130398 | c.C493A | p.Q165K |
| 402 | BLOOD | NIF3L1 | 2 | 201756998 | 201756998 | G | A | nonsynonymous | NM_001136039 | c.G332A | p.R111H |
| 402 | BLOOD | SUSD5 | 3 | 33194757 | 33194757 | T | C | nonsynonymous | NM_015551 | c.A1367G | p.E456G |
| 402 | BLOOD | PPM1M | 3 | 52282458 | 52282458 | C | G | nonsynonymous | NM_001122870 | c.C267G | p.H89Q |
| 402 | BLOOD | MTHFD2L | 4 | 75067034 | 75067034 | G | A | nonsynonymous | NM_001144978 | c.G659A | p.G220E |
| 402 | BLOOD | PDHA2 | 4 | 96761363 | 96761363 | T | A | nonsynonymous | NM_005390 | c.T62A | p.V21E |
| 402 | BLOOD | UBE2D3 | 4 | 103730849 | 103730849 | G | T | nonsynonymous | NM_181891 | c.C100A | p.Q34K |
| 402 | BLOOD | SCLT1 | 4 | 129867300 | 129867300 | C | A | nonsynonymous | NM_144643 | c.G1301T | p.R434L |
| 402 | BLOOD | IGF2R | 6 | 160469510 | 160469510 | C | G | nonsynonymous | NM_000876 | c.C2449G | p.L817V |
| 402 | BLOOD | SRRM3 | 7 | 75896593 | 75896593 | G | A | nonsynonymous | NM_001291831 | c.G848A | p.R283Q |
| 402 | BLOOD | GLDC | 9 | 6554781 | 6554781 | C | A | nonsynonymous | NM_000170 | c.G2203T | p.V735L |
| 402 | BLOOD | TEX10 | 9 | 103109328 | 103109328 | T | C | nonsynonymous | NM_017746 | c.A541G | p.I181V |
| 402 | BLOOD | EXPH5 | 11 | 108380281 | 108380281 | T | C | nonsynonymous | NM_015065 | c.A5953G | p.K1985E |
| 402 | BLOOD | TMPRSS4 | 11 | 117979606 | 117979606 | G | C | nonsynonymous | NM_001290094 | c.G503C | p.C168S |
| 402 | BLOOD | NECTIN1 | 11 | 119548402 | 119548402 | C | T | nonsynonymous | NM_002855 | c.G596A | p.R199Q |
| 402 | BLOOD | CLIP1 | 12 | 122812697 | 122812697 | C | T | nonsynonymous | NM_002956 | c.G3013A | p.E1005K |
| 402 | BLOOD | UPF3A | 13 | 115047496 | 115047496 | G | C | nonsynonymous | NM_023011 | c.G208C | p.V70L,UPF3A |
| 402 | BLOOD | SAMD4A | 14 | 55168914 | 55168914 | C | G | nonsynonymous | NM_015589 | c.C331G | p.H111D |
| 402 | BLOOD | TMED3 | 15 | 79603592 | 79603592 | A | G | nonsynonymous | NM_007364 | c.A1G | p.M1V |
| 402 | BLOOD | GOLGA6L10 | 15 | 82635194 | 82635194 | T | C | nonsynonymous | NM_001164465 | c.A1505G | p.E502G |
| 402 | BLOOD | CCNF | 16 | 2498978 | 2498978 | G | A | nonsynonymous | NM_001761 | c.G1217A | p.R406Q |
| 402 | BLOOD | MYLK3 | 16 | 46744689 | 46744689 | C | A | nonsynonymous | NM_182493 | c.G2127T | p.L709F |
| 402 | BLOOD | ACSF3 | 16 | 89211714 | 89211714 | G | A | nonsynonymous | NM_001127214 | c.G1406A | p.R469Q |
| 402 | BLOOD | DNAH2 | 17 | 7671326 | 7671326 | A | G | nonsynonymous | NM_020877 | c.A3784G | p.T1262A |
| 402 | BLOOD | PLIN4 | 19 | 4511283 | 4511283 | C | T | nonsynonymous | NM_001080400 | c.G2647A | p.A883T |
| 402 | BLOOD | KIR2DL4 | 19 | 55317669 | 55317669 | C | G | nonsynonymous | NM_001080772 | c.C625G | p.P209A |
| 402 | BLOOD | OGFR | 20 | 61444633 | 61444633 | G | A | nonsynonymous | NM_007346 | c.G1666A | p.E556K |
| 402 | BLOOD | OGFR | 20 | 61444637 | 61444637 | G | C | nonsynonymous | NM_007346 | c.G1670C | p.S557T |
| 402 | BLOOD | NCF4 | 22 | 37260160 | 37260160 | A | C | nonsynonymous | NM_000631 | c.A106C | p.T36P |
| 402 | BLOOD | VCX | X | 7812052 | 7812052 | G | A | nonsynonymous | NM_013452 | c.G616A | p.V206M |
| 402 | BLOOD | SYTL4 | X | 99941714 | 99941714 | G | A | nonsynonymous | NM_080737 | c.C1249T | p.R417W |
| 403 | BLOOD | FAM90A1 | 12 | 8374781 | 8374781 | - | ACG | inframe | NM_018088 | c.1031_1032insCGT | p.T344delinsTV |
| 403 | BLOOD | KRT4 | 12 | 53207583 | 53207583 | - | CACCAAAGCCACCAGTGCCGAAACC | frameshift | NM_002272 | c.259_260insGGTTTCGGCACTGGTGGCTTTGGTG | p.G87fs |
| 403 | BLOOD | CTAGE5 | 14 | 39784005 | 39784006 | TA | - | splicing |  |  |  |
| 403 | BLOOD | HSPG2 | 1 | 22186113 | 22186113 | T | G | nonsynonymous | NM_001291860 | c.A5242C | p.T1748P |
| 403 | BLOOD | COL9A2 | 1 | 40768853 | 40768853 | C | A | nonsynonymous | NM_001852 | c.G1553T | p.R518L |
| 403 | BLOOD | PLXNB1 | 3 | 48463811 | 48463811 | C | A | nonsynonymous | NM_002673 | c.G1348T | p.G450W |
| 403 | BLOOD | CABLES1 | 18 | 20716331 | 20716331 | C | A | nonsynonymous | NM_001100619 | c.C605A | p.A202D |
| 403 | BLOOD | NWD1 | 19 | 16884122 | 16884122 | G | A | nonsynonymous | NM_001290355 | c.G2191A | p.G731S |
| 404 | BLOOD | SGK223 | 8 | 8176387 | 8176387 | - | GGGGCG | inframe | NM_001080826 | c.3497_3498insCGCCCC | p.P1166delinsPAP |
| 404 | BLOOD | KRT4 | 12 | 53207583 | 53207583 | - | CACCAAAGCCACCAGTGCCGAAACCAG | inframe | NM_002272 | c.259_260insCTGGTTTCGGCACTGGTGGCTTTGGTG | p.G87delinsAGFGTGGFGG |
| 404 | BLOOD | CTAGE5 | 14 | 39784005 | 39784006 | TA | - | splicing |  |  |  |
| 404 | BLOOD | OBSCN | 1 | 228464655 | 228464655 | C | A | nonsynonymous | NM_001098623 | c.C6512A | p.P2171Q |
| 404 | BLOOD | RNF39 | 6 | 30039069 | 30039069 | G | T | nonsynonymous | NM_025236 | c.C1082A | p.P361Q |
| 404 | BLOOD | TARSL2 | 15 | 102264553 | 102264553 | G | T | stopgain | NM_152334 | c.C38A | p.S13X |
| 405 | BLOOD | KRTAP5-5 | 11 | 1651191 | 1651199 | GGCTGTGGA | - | inframe | NM_001001480 | c.121_129del | p.41_43del |
| 405 | BLOOD | PRRC2A | 6 | 31599370 | 31599370 | G | C | nonsynonymous | NM_004638 | c.G2920C | p.E974Q |
| 405 | BLOOD | ELN | 7 | 73471717 | 73471717 | G | A | nonsynonymous | NM_001278939 | c.G1363A | p.A455T |
| 405 | BLOOD | CTBP2 | 10 | 126678128 | 126678128 | T | A | nonsynonymous | NM_001329 | c.A1297T | p.T433S |
| 405 | BLOOD | MUC5B | 11 | 1253976 | 1253976 | A | G | nonsynonymous | NM_002458 | c.A2041G | p.S681G |
| 405 | BLOOD | MUC5B | 11 | 1253980 | 1253980 | A | G | nonsynonymous | NM_002458 | c.A2045G | p.D682G |
| 405 | BLOOD | ADAMTS7 | 15 | 79058879 | 79058879 | A | G | nonsynonymous | NM_014272 | c.T3374C | p.L1125P |
| 405 | BLOOD | ADAMTS7 | 15 | 79058880 | 79058880 | G | T | nonsynonymous | NM_014272 | c.C3373A | p.L1125M |
| 405 | BLOOD | ADAMTS7 | 15 | 79058882 | 79058882 | A | G | nonsynonymous | NM_014272 | c.T3371C | p.V1124A |
| 405 | BLOOD | GAGE13,GAGE2E,GAGE8 | X | 49189246 | 49189246 | C | T | nonsynonymous | NM_001098412 | c.C31T | p.R11W |
| 406 | BLOOD | ZNF706 | 8 | 102213962 | 102213962 | C | G | nonsynonymous | NM_016096 | c.G8C | p.R3P |
| 406 | BLOOD | IL18BP | 11 | 71712875 | 71712875 | T | C | nonsynonymous | NM_001039660 | c.T553C | p.S185P |
| 406 | BLOOD | CLIP1 | 12 | 122812697 | 122812697 | C | T | nonsynonymous | NM_002956 | c.G3013A | p.E1005K |
| 406 | BLOOD | AKAP13 | 15 | 86287035 | 86287035 | A | C | nonsynonymous | NM_007200 | c.A8371C | p.T2791P |
| 406 | BLOOD | CDC27 | 17 | 45232055 | 45232055 | C | T | nonsynonymous | NM_001256 | c.G940A | p.G314R |
| 406 | BLOOD | CDC27 | 17 | 45232079 | 45232079 | G | A | nonsynonymous | NM_001256 | c.C916T | p.P306S |
| 406 | BLOOD | PNKP | 19 | 50370425 | 50370425 | C | G | nonsynonymous | NM_007254 | c.G37C | p.E13Q |
| 406 | BLOOD | RRP1 | 21 | 45211298 | 45211298 | C | A | nonsynonymous | NM_003683 | c.C201A | p.D67E |
| 407 | BLOOD | MUC5B | 11 | 1253976 | 1253976 | A | G | nonsynonymous | NM_002458 | c.A2041G | p.S681G |
| 407 | BLOOD | MUC5B | 11 | 1253980 | 1253980 | A | G | nonsynonymous | NM_002458 | c.A2045G | p.D682G |
| 407 | BLOOD | MED17 | 11 | 93535027 | 93535027 | C | A | nonsynonymous | NM_004268 | c.C1355A | p.A452E |
| 407 | BLOOD | CYP4F2 | 19 | 15989730 | 15989730 | T | C | nonsynonymous | NM_001082 | c.A1414G | p.T472A |
| 408 | BLOOD | MADCAM1 | 19 | 501767 | 501767 | C | T | nonsynonymous | NM_130760 | c.C766T | p.P256S |
| 408 | BLOOD | PPP1R13L | 19 | 45889404 | 45889404 | G | T | nonsynonymous | NM_001142502 | c.C1850A | p.P617Q |
| 409 | BLOOD | MUC4 | 3 | 195507673 | 195507673 | A | G | nonsynonymous | NM_018406 | c.T10778C | p.V3593A |
| 409 | BLOOD | DCBLD1 | 6 | 117859971 | 117859971 | G | A | nonsynonymous | NM_173674 | c.G949A | p.D317N |
| 409 | BLOOD | PKHD1L1 | 8 | 110492352 | 110492352 | C | T | nonsynonymous | NM_177531 | c.C9311T | p.T3104I |
| 409 | BLOOD | CENPB | 20 | 3765947 | 3765947 | G | A | nonsynonymous | NM_001810 | c.C1184T | p.A395V |
| 409 | BLOOD | SYTL4 | X | 99956964 | 99956964 | G | A | stopgain | NM_080737 | c.C70T | p.R24X |
| 410 | LUNG | KRTAP4-1 | 17 | 39340796 | 39340852 | CGGCAGCAGCTGGACATACCACAGCTGGGGTGGCAGGTGGTCTGACAGCAGAGTGGG | - | inframe | NM_033060 | c.246_254del | p.82_85del |
| 410 | LUNG | PLAC4 | 21 | 42551272 | 42551272 | C | - | frameshift | NM_182832 | c.284delG | p.G95fs |
| 410 | LUNG | KMT2D | 12 | 49435162 | 49435162 | T | G | nonsynonymous | NM_003482 | c.A6391C | p.T2131P |
| 410 | LUNG | PLAC4 | 21 | 42551222 | 42551222 | T | G | nonsynonymous | NM_182832 | c.A334C | p.I112L |
| 410 | LUNG | PLAC4 | 21 | 42551245 | 42551245 | A | G | nonsynonymous | NM_182832 | c.T311C | p.L104P |
| 410 | LUNG | PLAC4 | 21 | 42551270 | 42551270 | A | G | nonsynonymous | NM_182832 | c.T286C | p.Y96H |
| 411 | LUNG | ODF1 | 8 | 103573011 | 103573037 | TGCAACCCCTGCAGCCCCTGCAACCCG | - | inframe | NM_024410 | c.652_678del | p.218_226del |
| 411 | LUNG | OBSCN | 1 | 228559967 | 228559967 | C | T | nonsynonymous | NM_001098623 | c.C21488T | p.P7163L |
| 411 | LUNG | TOR4A | 9 | 140174165 | 140174165 | G | A | nonsynonymous | NM_017723 | c.G1024A | p.A342T |
| 411 | LUNG | PDPR | 16 | 70172845 | 70172845 | G | A | nonsynonymous | NM_017990 | c.G1234A | p.V412I |
| 411 | LUNG | R3HDM4 | 19 | 897519 | 897519 | C | T | nonsynonymous | NM_138774 | c.G725A | p.R242Q |
| 411 | LUNG | LRRC8E | 19 | 7964768 | 7964768 | C | G | nonsynonymous | NM_025061 | c.C1361G | p.P454R |
| 411 | LUNG | CACNA1A | 19 | 13428124 | 13428124 | C | T | nonsynonymous | NM_001127222 | c.G1357A | p.A453T |
| 412 | BLOOD | CTBS | 1 | 85039999 | 85040007 | GCAGCGCCA | - | inframe | NM_004388 | c.92_100del | p.31_34del |
| 412 | BLOOD | NUTM2F | 9 | 97080945 | 97080947 | AGA | - | inframe | NM_017561 | c.2071_2073del | p.691_691del |
| 412 | BLOOD | DSC3 | 18 | 28588315 | 28588315 | A | C | nonsynonymous | NM_001941 | c.T1440G | p.I480M |
| 412 | BLOOD | DSC2 | 18 | 28654748 | 28654748 | C | A | nonsynonymous | NM_024422 | c.G1789T | p.V597F |
| 413 | BLOOD | RP1L1 | 8 | 10467579 | 10467581 | TTC | - | inframe | NM_178857 | c.4027_4029del | p.1343_1343del |
| 413 | BLOOD | HLA-DQB1 | 6 | 32629935 | 32629935 | C | G | nonsynonymous | NM_001243961 | c.G470C | p.G157A |
| 413 | BLOOD | CEL | 9 | 135947018 | 135947018 | A | G | nonsynonymous | NM_001807 | c.A2138G | p.E713G |
| 413 | BLOOD | CEL | 9 | 135947020 | 135947020 | A | G | nonsynonymous | NM_001807 | c.A2140G | p.T714A |
| 413 | BLOOD | CEL | 9 | 135947023 | 135947023 | G | C | nonsynonymous | NM_001807 | c.G2143C | p.A715P |
| 413 | BLOOD | PIDD1 | 11 | 800630 | 800630 | G | A | nonsynonymous | NM_145886 | c.C1954T | p.R652W |
| 414 | BLOOD | NPIPB5 | 16 | 22545744 | 22545755 | TCCACCCTCAGC | - | inframe | NM_001135865 | c.1440_1451del | p.480_484del |
| 414 | BLOOD | KRTAP4-1 | 17 | 39340796 | 39340852 | CGGCAGCAGCTGGACATACCACAGCTGGGGTGGCAGGTGGTCTGACAGCAGAGTGGG | - | inframe | NM_033060 | c.246_254del | p.82_85del |
| 414 | BLOOD | MUC4 | 3 | 195511358 | 195511358 | C | G | nonsynonymous | NM_018406 | c.G7093C | p.D2365H |
| 414 | BLOOD | ASIC3 | 7 | 150748286 | 150748286 | C | T | nonsynonymous | NM_004769 | c.C1094T | p.A365V |
| 414 | BLOOD | TAS2R19 | 12 | 11174372 | 11174372 | C | G | nonsynonymous | NM_176888 | c.G799C | p.V267L |
| 414 | BLOOD | DDX11 | 12 | 31237978 | 31237978 | C | T | nonsynonymous | NM_001257144 | c.C556T | p.R186W |
| 414 | BLOOD | TICRR | 15 | 90126163 | 90126163 | C | T | stopgain | NM_152259 | c.C901T | p.R301X |
| 414 | BLOOD | NCOR1 | 17 | 16097870 | 16097870 | C | A | nonsynonymous | NM_006311 | c.G14T | p.G5V |
| 414 | BLOOD | ZNF814 | 19 | 58385748 | 58385748 | G | A | nonsynonymous | NM_001144989 | c.C1010T | p.A337V |
| 415 | BLOOD | YEATS2 | 3 | 183493744 | 183493746 | GGA | - | inframe | NM_018023 | c.2410_2412del | p.804_804del |
| 415 | BLOOD | PRSS48 | 4 | 152201018 | 152201018 | - | CAGGT | frameshift | NM_183375 | c.123_124insCAGGT | p.W41fs |
| 415 | BLOOD | SPAG1 | 8 | 101206459 | 101206459 | - | GAC | inframe | NM_003114 | c.1059_1060insGAC | p.K353delinsKD |
| 415 | BLOOD | OR13C2 | 9 | 107367393 | 107367396 | GTTA | - | frameshift | NM_001004481 | c.513_516del | p.N171fs |
| 415 | BLOOD | PLAC9 | 10 | 81904097 | 81904097 | - | AGA | inframe | NM_001012973 | c.281_282insAGA | p.G94delinsGE |
| 415 | BLOOD | CHD4 | 12 | 6711207 | 6711209 | CTT | - | inframe | NM_001273 | c.355_357del | p.119_119del |
| 415 | BLOOD | FAM90A1 | 12 | 8374781 | 8374781 | - | ACG | inframe | NM_018088 | c.1031_1032insCGT | p.T344delinsTV |
| 415 | BLOOD | OR7G3 | 19 | 9236698 | 9236698 | - | ATGGT | frameshift | NM_001001958 | c.928_929insACCAT | p.S310fs |
| 415 | BLOOD | ADM2 | 22 | 50921149 | 50921166 | ACACTCGGGCCCCCGAAG | - | inframe | NM_001253845 | c.264_281del | p.88_94del |
| 415 | BLOOD | RFWD2 | 1 | 176175819 | 176175819 | A | C | nonsynonymous | NM_022457 | c.T296G | p.V99G |
| 415 | BLOOD | CEP350 | 1 | 180044304 | 180044304 | A | G | nonsynonymous | NM_014810 | c.A5715G | p.I1905M |
| 415 | BLOOD | KIAA1614 | 1 | 180905724 | 180905724 | C | G | stopgain | NM_020950 | c.C2679G | p.Y893X |
| 415 | BLOOD | ZNF148 | 3 | 124951307 | 124951307 | C | T | nonsynonymous | NM_021964 | c.G2263A | p.V755M |
| 415 | BLOOD | INPP5E | 9 | 139329253 | 139329253 | C | T | nonsynonymous | NM_019892 | c.G875A | p.R292H |
| 415 | BLOOD | BAZ2A | 12 | 57005679 | 57005679 | A | T | nonsynonymous | NM_013449 | c.T1493A | p.V498E |
| 415 | BLOOD | PARP4 | 13 | 25021323 | 25021323 | A | G | nonsynonymous | NM_006437 | c.T3116C | p.I1039T |
| 415 | BLOOD | HMHA1 | 19 | 1080681 | 1080681 | G | A | nonsynonymous | NM_012292 | c.G1913A | p.G638E |
| 415 | BLOOD | MYO9B | 19 | 17305607 | 17305607 | A | G | nonsynonymous | NM_004145 | c.A3371G | p.E1124G |
| 415 | BLOOD | IL12RB1 | 19 | 18180367 | 18180367 | G | A | nonsynonymous | NM_001290024 | c.C1298T | p.P433L |
| 415 | BLOOD | LRRC4B | 19 | 51052073 | 51052073 | G | A | nonsynonymous | NM_001080457 | c.C23T | p.P8L |
| 415 | BLOOD | APOL2 | 22 | 36624231 | 36624231 | C | T | nonsynonymous | NM_145637 | c.G233A | p.R78K |
| 415 | BLOOD | LGALS2 | 22 | 37966689 | 37966689 | G | A | nonsynonymous | NM_006498 | c.C143T | p.P48L |
| 416 | BLOOD | MAGEF1 | 3 | 184429133 | 184429133 | - | TCC | inframe | NM_022149 | c.476_477insGGA | p.D159delinsED |
| 416 | BLOOD | PIBF1 | 13 | 73409508 | 73409508 | - | A | splicing |  |  |  |
| 416 | BLOOD | IRF2BPL | 14 | 77493648 | 77493650 | GCG | - | inframe | NM_024496 | c.486_488del | p.162_163del |
| 416 | BLOOD | NME4 | 16 | 450140 | 450140 | - | AG | frameshift | NM_001286435 | c.472_473insAG | p.R158fs |
| 416 | BLOOD | IL32 | 16 | 3119297 | 3119297 | - | G | frameshift | NM_001012718 | c.509dupG | p.R170fs |
| 416 | BLOOD | APOBEC3H | 22 | 39496323 | 39496325 | AAC | - | inframe | NM_001166003 | c.40_42del | p.14_14del |
| 416 | BLOOD | FOXP1 | 3 | 71027096 | 71027096 | T | C | nonsynonymous | NM_032682 | c.A1231G | p.T411A |
| 416 | BLOOD | ARHGEF26 | 3 | 153912438 | 153912438 | C | G | nonsynonymous | NM_001251962 | c.C1776G | p.I592M |
| 416 | BLOOD | IGF2R | 6 | 160523631 | 160523631 | C | T | nonsynonymous | NM_000876 | c.C6923T | p.A2308V |
| 416 | BLOOD | TACC1 | 8 | 38677696 | 38677696 | G | A | nonsynonymous | NM_006283 | c.G934A | p.E312K |
| 416 | BLOOD | AHNAK2 | 14 | 105415229 | 105415229 | T | C | nonsynonymous | NM_138420 | c.A6559G | p.M2187V |
| 416 | BLOOD | MED24 | 17 | 38189700 | 38189700 | G | A | nonsynonymous | NM_014815 | c.C569T | p.T190I |
| 416 | BLOOD | SNAPC2 | 19 | 7987522 | 7987522 | C | T | nonsynonymous | NM_003083 | c.C878T | p.P293L |
| 417 | BLOOD | HSPBP1 | 19 | 55790886 | 55790886 | - | GCCGCCGCC | inframe | NM_001130106 | c.90_91insGGCGGCGGC | p.S31delinsGGGS |
| 417 | BLOOD | OBSCN | 1 | 228528225 | 228528225 | C | A | nonsynonymous | NM_001098623 | c.C17434A | p.L5812M |
| 417 | BLOOD | NEB | 2 | 152473911 | 152473911 | C | G | nonsynonymous | NM_004543 | c.G10383C | p.M3461I |
| 417 | BLOOD | MUC4 | 3 | 195505788 | 195505788 | G | C | nonsynonymous | NM_018406 | c.C12663G | p.H4221Q |
| 417 | BLOOD | ESRRA | 11 | 64083328 | 64083328 | C | T | nonsynonymous | NM_001282450 | c.C1162T | p.L388F |
| 417 | BLOOD | ESRRA | 11 | 64083331 | 64083331 | C | T | nonsynonymous | NM_001282450 | c.C1165T | p.R389C |
| 417 | BLOOD | DENND5B | 12 | 31562217 | 31562217 | G | A | nonsynonymous | NM_144973 | c.C2783T | p.T928I |
| 418 | BLOOD | RBM25 | 14 | 73572607 | 73572608 | AG | - | frameshift | NM_021239 | c.1195_1196del | p.R399fs |
| 418 | BLOOD | KCNH2 | 7 | 150655573 | 150655573 | G | A | nonsynonymous | NM_000238 | c.C490T | p.R164C |
| 418 | BLOOD | CEL | 9 | 135947032 | 135947032 | C | A | nonsynonymous | NM_001807 | c.C2152A | p.P718T |
| 418 | BLOOD | CYP2A13 | 19 | 41595958 | 41595958 | C | T | nonsynonymous | NM_000766 | c.C350T | p.A117V |
| 419 | BLOOD | TNRC6A | 16 | 24788423 | 24788434 | GCAGCCACAGCC | - | inframe | NM_014494 | c.333_344del | p.111_115del |
| 419 | BLOOD | CHD3 | 17 | 7796794 | 7796794 | A | C | nonsynonymous | NM_001005273 | c.A700C | p.I234L |
| 419 | BLOOD | CDC27 | 17 | 45249328 | 45249328 | G | A | nonsynonymous | NM_001256 | c.C206T | p.P69L |
| 419 | BLOOD | CDC27 | 17 | 45249360 | 45249360 | A | T | stopgain | NM_001256 | c.T174A | p.Y58X |
| 420 | BLOOD | PCDH12 | 5 | 141324955 | 141324955 | - | CTGCTGCTG | inframe | NM_016580 | c.3545_3546insCAGCAGCAG | p.R1182delinsSSSR |
| 420 | BLOOD | RBMXL1 | 1 | 89449390 | 89449390 | T | C | nonsynonymous | NM_019610 | c.A120G | p.I40M |
| 420 | BLOOD | PRR21 | 2 | 240982248 | 240982248 | T | C | nonsynonymous | NM_001080835 | c.A152G | p.H51R |
| 420 | BLOOD | DENND6A | 3 | 57632086 | 57632086 | G | C | nonsynonymous | NM_152678 | c.C898G | p.P300A |
| 420 | BLOOD | TTC14 | 3 | 180327530 | 180327530 | C | T | nonsynonymous | NM_133462 | c.C1513T | p.H505Y |
| 420 | BLOOD | DROSHA | 5 | 31495479 | 31495479 | C | A | nonsynonymous | NM_013235 | c.G1669T | p.A557S |
| 420 | BLOOD | FKBPL | 6 | 32097421 | 32097421 | G | C | nonsynonymous | NM_022110 | c.C137G | p.T46R |
| 420 | BLOOD | MGAM | 7 | 141705413 | 141705413 | T | C | nonsynonymous | NM_004668 | c.T83C | p.I28T |
| 420 | BLOOD | ERMP1 | 9 | 5830877 | 5830877 | G | A | nonsynonymous | NM_024896 | c.C490T | p.P164S |
| 420 | BLOOD | DDX11 | 12 | 31237978 | 31237978 | C | T | nonsynonymous | NM_001257144 | c.C556T | p.R186W |
| 420 | BLOOD | MED4 | 13 | 48669181 | 48669181 | C | G | nonsynonymous | NM_014166 | c.G34C | p.E12Q |
| 420 | BLOOD | MADCAM1 | 19 | 501762 | 501762 | A | C | nonsynonymous | NM_130760 | c.A761C | p.Q254P |
| 420 | BLOOD | RBMX | X | 135956506 | 135956506 | C | G | nonsynonymous | NM_002139 | c.G971C | p.R324P |
| 420 | BLOOD | RBMX | X | 135958730 | 135958730 | C | A | nonsynonymous | NM_002139 | c.G473T | p.G158V |
| 421 | BLOOD | MUC20 | 3 | 195452799 | 195452799 | C | T | nonsynonymous | NM_001282506 | c.C1325T | p.T442I |
| 421 | BLOOD | CIZ1 | 9 | 130952718 | 130952718 | A | G | nonsynonymous | NM_001131016 | c.T176C | p.L59P |
| 421 | BLOOD | ARG2 | 14 | 68086741 | 68086741 | A | G | nonsynonymous | NM_001172 | c.A47G | p.H16R |
| 421 | BLOOD | MADCAM1 | 19 | 501786 | 501786 | C | A | nonsynonymous | NM_130760 | c.C785A | p.P262Q |
| 421 | BLOOD | LMF2 | 22 | 50944492 | 50944492 | C | T | nonsynonymous | NM_033200 | c.G746A | p.R249H |
| 422 | BLOOD | DND1 | 5 | 140052407 | 140052407 | G | A | nonsynonymous | NM_194249 | c.C227T | p.P76L |
| 422 | BLOOD | MUC2 | 11 | 1092872 | 1092872 | C | G | nonsynonymous | NM_002457 | c.C4691G | p.T1564S |
| 423 | BLOOD | KRT2 | 12 | 53045603 | 53045603 | - | AAGCCGCTGCCACCTCCA | inframe | NM_000423 | c.323_324insTGGAGGTGGCAGCGGCTT | p.F108delinsFGGGSGF |
| 423 | BLOOD | KNDC1 | 10 | 134999646 | 134999646 | C | T | nonsynonymous | NM_152643 | c.C794T | p.T265I |
| 423 | BLOOD | OR1D5 | 17 | 2966273 | 2966273 | G | A | nonsynonymous | NM_014566 | c.C629T | p.P210L |
| 423 | BLOOD | ANKRD30B | 18 | 14779986 | 14779986 | G | A | nonsynonymous | NM_001145029 | c.G1448A | p.R483Q |
| 424 | BLOOD | NOTCH2 | 1 | 120612003 | 120612004 | GG | - | frameshift | NM_024408 | c.17_18del | p.P6fs |
| 424 | BLOOD | MCC | 5 | 112824033 | 112824033 | - | GCTGCC | inframe | NM_001085377 | c.78_79insGGCAGC | p.S27delinsGSS |
| 424 | BLOOD | EP400 | 12 | 132547093 | 132547093 | - | CAG | inframe | NM_015409 | c.8181_8182insCAG | p.Q2727delinsQQ |
| 424 | BLOOD | KDM6B | 17 | 7750177 | 7750177 | - | ACCACC | inframe | NM_001080424 | c.752_753insACCACC | p.L251delinsLPP |
| 424 | BLOOD | KRT6B | 12 | 52845435 | 52845435 | C | T | nonsynonymous | NM_005555 | c.G428A | p.S143N |
| 424 | BLOOD | ACOT2 | 14 | 74041748 | 74041748 | A | G | nonsynonymous | NM_006821 | c.A983G | p.H328R |
| 424 | BLOOD | HSBP1 | 16 | 83842988 | 83842988 | T | A | nonsynonymous | NM_001537 | c.T191A | p.L64Q |
| 424 | BLOOD | ZNRF4 | 19 | 5455543 | 5455543 | G | C | nonsynonymous | NM_181710 | c.G41C | p.S14T |
| 424 | BLOOD | TTLL1 | 22 | 43471583 | 43471583 | T | C | nonsynonymous | NM_012263 | c.A10G | p.K4E |
| 424 | BLOOD | ARSD | X | 2836184 | 2836184 | C | T | nonsynonymous | NM_001669 | c.G524A | p.G175D |
| 425 | BLOOD | KDM6B | 17 | 7750177 | 7750177 | - | ACCACC | inframe | NM_001080424 | c.752_753insACCACC | p.L251delinsLPP |
| 426 | BLOOD | TMEM2 | 9 | 74300311 | 74300311 | - | A | splicing |  |  |  |
| 426 | BLOOD | TTN | 2 | 179400106 | 179400106 | G | A | nonsynonymous | NM_001267550 | c.C101236T | p.R33746C |
| 426 | BLOOD | HOXC5 | 12 | 54427115 | 54427115 | A | C | nonsynonymous | NM_018953 | c.A209C | p.H70P |
| 426 | BLOOD | CHD3 | 17 | 7796794 | 7796794 | A | C | nonsynonymous | NM_001005273 | c.A700C | p.I234L |
| 426 | BLOOD | PIPOX | 17 | 27381646 | 27381646 | G | A | stopgain | NM_016518 | c.G744A | p.W248X |
| 426 | BLOOD | ASXL1 | 20 | 31022238 | 31022238 | C | T | stopgain | NM_015338 | c.C1723T | p.Q575X |
| 427 | BLOOD | UFSP1 | 7 | 100486737 | 100486737 | C | - | frameshift | NM_001015072 | c.156delG | p.S52fs |
| 427 | BLOOD | ZNF283 | 19 | 44351167 | 44351172 | GGAGAT | - | frameshift | NM_001297752 |  |  |
| 427 | BLOOD | HRC | 19 | 49657889 | 49657889 | - | TCC | inframe | NM_002152 | c.605_606insGGA | p.E202delinsEE |
| 427 | BLOOD | RALY | 20 | 32664864 | 32664864 | - | CAG | inframe | NM_016732 | c.689_690insCAG | p.A230delinsAS |
| 427 | BLOOD | HSPG2 | 1 | 22200454 | 22200454 | G | T | nonsynonymous | NM_001291860 | c.C3710A | p.A1237E |
| 427 | BLOOD | STAM2 | 2 | 152982745 | 152982745 | T | C | nonsynonymous | NM_005843 | c.A1174G | p.M392V |
| 427 | BLOOD | KIF4B | 5 | 154395970 | 154395970 | A | G | nonsynonymous | NM_001099293 | c.A2551G | p.K851E |
| 427 | BLOOD | HK3 | 5 | 176314060 | 176314060 | C | G | nonsynonymous | NM_002115 | c.G1800C | p.Q600H |
| 427 | BLOOD | OR2B3 | 6 | 29054914 | 29054914 | T | A | nonsynonymous | NM_001005226 | c.A112T | p.T38S |
| 427 | BLOOD | PRRC2A | 6 | 31600106 | 31600106 | C | A | nonsynonymous | NM_004638 | c.C3656A | p.S1219Y |
| 427 | BLOOD | TNXB | 6 | 32052444 | 32052444 | C | T | nonsynonymous | NM_019105 | c.G3191A | p.R1064H |
| 427 | BLOOD | NOTCH4 | 6 | 32170037 | 32170037 | A | G | nonsynonymous | NM_004557 | c.T3571C | p.C1191R |
| 427 | BLOOD | KIFC1 | 6 | 33374614 | 33374614 | G | A | nonsynonymous | NM_002263 | c.G1939A | p.V647I |
| 427 | BLOOD | SCUBE3 | 6 | 35205741 | 35205741 | C | T | nonsynonymous | NM_001303136 | c.C772T | p.H258Y |
| 427 | BLOOD | MAPK13 | 6 | 36106767 | 36106767 | C | T | nonsynonymous | NM_002754 | c.C953T | p.T318M |
| 427 | BLOOD | CCDC146 | 7 | 76903799 | 76903799 | A | C | nonsynonymous | NM_020879 | c.A1270C | p.K424Q |
| 427 | BLOOD | TJP1 | 15 | 30053378 | 30053378 | T | A | nonsynonymous | NM_003257 | c.A974T | p.H325L |
| 427 | BLOOD | ISLR2 | 15 | 74426858 | 74426858 | C | G | nonsynonymous | NM_001130136 | c.C1763G | p.P588R |
| 427 | BLOOD | ADAMTS7 | 15 | 79051801 | 79051801 | C | A | nonsynonymous | NM_014272 | c.G5023T | p.A1675S |
| 427 | BLOOD | ZNF423 | 16 | 49670535 | 49670535 | T | C | nonsynonymous | NM_015069 | c.A2528G | p.N843S |
| 427 | BLOOD | HYDIN | 16 | 71004596 | 71004596 | G | A | nonsynonymous | NM_001270974 | c.C5446T | p.R1816C |
| 427 | BLOOD | TAF1C | 16 | 84213176 | 84213176 | G | A | nonsynonymous | NM_001243159 | c.C754T | p.R252C |
| 427 | BLOOD | CDH15 | 16 | 89260188 | 89260188 | G | A | nonsynonymous | NM_004933 | c.G2018A | p.R673H |
| 427 | BLOOD | CHD3 | 17 | 7796794 | 7796794 | A | C | nonsynonymous | NM_001005273 | c.A700C | p.I234L |
| 427 | BLOOD | SEZ6 | 17 | 27283241 | 27283241 | C | G | nonsynonymous | NM_178860 | c.G2888C | p.R963P |
| 427 | BLOOD | ATP9B | 18 | 77134012 | 77134012 | T | C | nonsynonymous | NM_198531 | c.T3185C | p.V1062A |
| 427 | BLOOD | ZNF729 | 19 | 22497201 | 22497201 | A | G | nonsynonymous | NM_001242680 | c.A982G | p.T328A |
| 427 | BLOOD | ARSD | X | 2835863 | 2835863 | G | T | nonsynonymous | NM_001669 | c.C845A | p.A282D |
| 428 | BLOOD | POU4F2 | 4 | 147560457 | 147560457 | - | GGC | inframe | NM_004575 | c.165_166insGGC | p.G55delinsGG |
| 428 | BLOOD | AUTS2 | 7 | 70255576 | 70255576 | - | CCA | inframe | NM_015570 | c.3374_3375insCCA | p.S1125delinsSH |
| 428 | BLOOD | UPF3A | 13 | 115047496 | 115047496 | G | C | nonsynonymous | NM_015570 | c.3374_3375insCCA | p.S1125delinsSH |
| 430 | BLOOD | IRF5 | 7 | 128587352 | 128587381 | ACTCTGCAGCCGCCCACTCTGCGGCCGCCT | - | inframe | NM_001098630 | c.502_531del | p.168_177del |
| 430 | BLOOD | ANKRD36 | 2 | 97877478 | 97877478 | G | A | nonsynonymous | NM_001164315 | c.G3469A | p.V1157M |
| 430 | BLOOD | MUC4 | 3 | 195505859 | 195505859 | T | C | nonsynonymous | NM_018406 | c.A12592G | p.T4198A |
| 431 | BLOOD | PDCD6 | 5 | 271858 | 271869 | CCGGCCCTGGGG | - | inframe | NM_013232 | c.23_34del | p.8_12del |
| 431 | BLOOD | MUC4 | 3 | 195505836 | 195505836 | G | C | nonsynonymous | NM_018406 | c.C12615G | p.H4205Q |
| 431 | BLOOD | RPUSD4 | 11 | 126075677 | 126075677 | C | T | splicing |  |  |  |
| 431 | BLOOD | MYO5C | 15 | 52510748 | 52510748 | G | T | nonsynonymous | NM_018728 | c.C3922A | p.Q1308K |
| 432 | BLOOD | SKA3 | 13 | 21746601 | 21746601 | G | - | frameshift | NM_145061 | c.208delC | p.Q70fs |
| 432 | BLOOD | PHF21A | 11 | 45986887 | 45986887 | G | T | nonsynonymous | NM_001101802 | c.C972A | p.S324R |
| 432 | BLOOD | CLIP1 | 12 | 122812697 | 122812697 | C | T | nonsynonymous | NM_002956 | c.G3013A | p.E1005K |
| 432 | BLOOD | MYLK3 | 16 | 46744689 | 46744689 | C | A | nonsynonymous | NM_182493 | c.G2127T | p.L709F |
| 433 | BLOOD | RBM5 | 3 | 50155888 | 50155889 | GA | - | frameshift | NM_005778 | c.2447_2448del | p.*816fs |
| 433 | BLOOD | MAU2 | 19 | 19431690 | 19431704 | GCGGCCCAGGCGGCG | - | inframe | NM_015329 | c.22_36del | p.8_12del |
| 433 | BLOOD | FOXJ3 | 1 | 42776742 | 42776742 | C | A | nonsynonymous | NM_001198851 | c.G23T | p.C8F |
| 433 | BLOOD | EXO1 | 1 | 242020734 | 242020734 | C | A | nonsynonymous | NM_130398 | c.C493A | p.Q165K |
| 433 | BLOOD | MUC4 | 3 | 195513491 | 195513491 | G | T | nonsynonymous | NM_018406 | c.C4960A | p.P1654T |
| 433 | BLOOD | PHF21A | 11 | 45986887 | 45986887 | G | T | nonsynonymous | NM_001101802 | c.C972A | p.S324R |
| 433 | BLOOD | CLIP1 | 12 | 122812697 | 122812697 | C | T | nonsynonymous | NM_002956 | c.G3013A | p.E1005K |
| 433 | BLOOD | CCNA1 | 13 | 37012866 | 37012866 | T | G | nonsynonymous | NM_003914 | c.T755G | p.V252G |
| 433 | BLOOD | AKAP13 | 15 | 86287035 | 86287035 | A | C | nonsynonymous | NM_007200 | c.A8371C | p.T2791P |
| 433 | BLOOD | MYLK3 | 16 | 46744689 | 46744689 | C | A | nonsynonymous | NM_182493 | c.G2127T | p.L709F |
| 434 | BLOOD | ZNF706 | 8 | 102213962 | 102213962 | C | G | nonsynonymous | NM_016096 | c.G8C | p.R3P |
| 434 | BLOOD | MYLK3 | 16 | 46744689 | 46744689 | C | A | nonsynonymous | NM_182493 | c.G2127T | p.L709F |
| 434 | BLOOD | PNKP | 19 | 50370425 | 50370425 | C | G | nonsynonymous | NM_007254 | c.G37C | p.E13Q |
| 435 | BLOOD | LOC554223 | 6 | 29760353 | 29760373 | GCGGGCGCCGTGGATGGAGCA | - | inframe | NM_001207043 | c.438_458del | p.146_153del |
| 435 | BLOOD | BAIAP2L2 | 22 | 38482353 | 38482394 | TGCGGGAGCGGGACTGGCCATCCCAGTACTCCGAGGGTGCTA | - | inframe | NM_025045 | c.1322_1363del | p.441_455del |
| 435 | BLOOD | CUL9 | 6 | 43188650 | 43188650 | G | A | splicing |  |  |  |
| 435 | BLOOD | GAB2 | 11 | 77937657 | 77937657 | C | G | nonsynonymous | NM_080491 | c.G1061C | p.R354P |
| 435 | BLOOD | CHD3 | 17 | 7796794 | 7796794 | A | C | nonsynonymous | NM_001005273 | c.A700C | p.I234L |
| 436 | BLOOD | POU4F2 | 4 | 147560457 | 147560457 | - | GGC | inframe | NM_004575 | c.165_166insGGC | p.G55delinsGG |
| 436 | BLOOD | NPHP4 | 1 | 6029316 | 6029316 | A | T | nonsynonymous | NM_015102 | c.T283A | p.L95M |
| 436 | BLOOD | ANKRD36 | 2 | 97877478 | 97877478 | G | A | nonsynonymous | NM_001164315 | c.G3469A | p.V1157M |
| 437 | BLOOD | ODF1 | 8 | 103573011 | 103573037 | TGCAACCCCTGCAGCCCCTGCAACCCG | - | inframe | NM_024410 | c.652_678del | p.218_226del |
| 437 | BLOOD | FAM90A1 | 12 | 8374781 | 8374781 | - | ACG | inframe | NM_018088 | c.1031_1032insCGT | p.T344delinsTV |
| 437 | BLOOD | ZNF653 | 19 | 11598224 | 11598224 | C | T | nonsynonymous | NM_138783 | c.G1054A | p.E352K |
| 437 | BLOOD | APLP1 | 19 | 36367509 | 36367509 | C | G | nonsynonymous | NM_001024807 | c.C1435G | p.P479A |
| 437 | BLOOD | SIRPA | 20 | 1895963 | 1895963 | A | G | nonsynonymous | NM_001040023 | c.A298G | p.N100D |
| 437 | BLOOD | SIRPA | 20 | 1895965 | 1895965 | C | A | nonsynonymous | NM_001040023 | c.C300A | p.N100K |
| 438 | BLOOD | ZIC5 | 13 | 100622668 | 100622688 | GGCGGCGGCGGCGGCGGCGGC | - | inframe | NM_033132 | c.1242_1262del | p.414_421del |
| 438 | BLOOD | HDGFRP2 | 19 | 4499633 | 4499647 | AGCTGGCCGGGGAGG | - | frameshift | NM_001001520 | c.1721_1722del | p.K574fs |
| 439 | BLOOD | TCOF1 | 5 | 149776385 | 149776387 | AGA | - | inframe | NM_001135243 | c.4322_4324del | p.1441_1442del |
| 439 | BLOOD | SOGA3 | 6 | 127837125 | 127837125 | T | C | nonsynonymous | NM_001012279 | c.A635G | p.E212G |
| 439 | BLOOD | CHD3 | 17 | 7796803 | 7796803 | T | C | nonsynonymous | NM_001005273 | c.T709C | p.S237P |
| 440 | BLOOD | CYP2A13 | 19 | 41595958 | 41595958 | C | T | nonsynonymous | NM_000766 | c.C350T | p.A117V |
| 440 | BLOOD | ZNF814 | 19 | 58385546 | 58385546 | G | T | nonsynonymous | NM_001144989 | c.C1212A | p.D404E |
| 441 | LUNG | CASC5 | 15 | 40914465 | 40914465 | C | A | nonsynonymous | NM_170589 | c.C2081A | p.T694K |
| 442 | LUNG | KDM6B | 17 | 7751859 | 7751864 | CACCAC | - | inframe | NM_001080424 | c.2253_2258del | p.751_753del |
| 442 | LUNG | MED15 | 22 | 20920813 | 20920813 | - | CAG | inframe | NM_001003891 | c.750_751insCAG | p.Q250delinsQQ |
| 442 | LUNG | MUC20 | 3 | 195447886 | 195447886 | G | C | nonsynonymous | NM_001282506 | c.G8C | p.C3S |
| 442 | LUNG | RAD50 | 5 | 131944338 | 131944338 | C | T | nonsynonymous | NM_005732 | c.C2750T | p.T917I |
| 442 | LUNG | ARSD | X | 2836181 | 2836181 | A | T | nonsynonymous | NM_001669 | c.T527A | p.M176K |
| 442 | LUNG | ARSD | X | 2836184 | 2836184 | C | T | nonsynonymous | NM_001669 | c.G524A | p.G175D |
| 444 | LUNG | DDHD2 | 8 | 38103783 | 38103783 | C | G | nonsynonymous | NM_015214 | c.C1090G | p.Q364E |
| 445 | LUNG | KLHL5 | 4 | 39064161 | 39064161 | - | C | frameshift | NM_015990 | c.27_28insC | p.H9fs |
| 445 | LUNG | ZNF814 | 19 | 58385546 | 58385546 | G | T | nonsynonymous | NM_001144989 | c.C1212A | p.D404E |
| 446 | LUNG | PDCD6 | 5 | 271858 | 271869 | CCGGCCCTGGGG | - | inframe | NM_013232 | c.23_34del | p.8_12del |
| 446 | LUNG | HLA-DQB1 | 6 | 32629935 | 32629935 | C | G | nonsynonymous | NM_001243961 | c.G470C | p.G157A |
| 446 | LUNG | ADAM2 | 8 | 39613300 | 39613300 | C | A | nonsynonymous | NM_001464 | c.G1744T | p.A582S |
| 446 | LUNG | JAK2 | 9 | 5126343 | 5126343 | G | A | nonsynonymous | NM_004972 | c.G3188A | p.R1063H |
| 446 | LUNG | KIF24 | 9 | 34255757 | 34255757 | C | T | nonsynonymous | NM_194313 | c.G3848A | p.R1283H |
| 446 | LUNG | PKD2L1 | 10 | 102057362 | 102057362 | A | C | nonsynonymous | NM_001253837 | c.T592G | p.W198G,PKD2L1 |
| 446 | LUNG | HDAC7 | 12 | 48187265 | 48187265 | C | A | nonsynonymous | NM_015401 | c.G1565T | p.R522L |
| 446 | LUNG | ATG14 | 14 | 55857656 | 55857656 | A | G | nonsynonymous | NM_014924 | c.T382C | p.C128R |
| 446 | LUNG | SHPK | 17 | 3524560 | 3524560 | T | C | nonsynonymous | NM_013276 | c.A794G | p.Y265C |
| 446 | LUNG | FBL | 19 | 40330874 | 40330874 | G | A | nonsynonymous | NM_001436 | c.C377T | p.S126L |
| 446 | LUNG | KLK10 | 19 | 51518060 | 51518060 | T | C | nonsynonymous | NM_002776 | c.A827G | p.N276S |
| 446 | LUNG | SBK2 | 19 | 56042661 | 56042661 | C | T | nonsynonymous | NM_001101401 | c.G305A | p.G102D |
| 446 | LUNG | SPAG4 | 20 | 34205139 | 34205139 | C | A | nonsynonymous | NM_003116 | c.C386A | p.P129Q |
| 446 | LUNG | AIFM3 | 22 | 21331032 | 21331032 | C | T | nonsynonymous | NM_144704 | c.C1123T | p.R375C |
| 447 | BLOOD | CCDC180 | 9 | 100092968 | 100092968 | - | GAGGAG | inframe | NM_020893 | c.2325_2326insGAGGAG | p.E775delinsEEE |
| 447 | BLOOD | DHRS7 | 14 | 60611700 | 60611708 | TTAAAGATT | - | inframe | NM_016029 | c.996_1004del | p.332_335del |
| 447 | BLOOD | SOX30 | 5 | 157078813 | 157078813 | C | A | nonsynonymous | NM_178424 | c.G274T | p.A92S |
| 447 | BLOOD | ADCYAP1R1 | 7 | 31104520 | 31104520 | A | G | nonsynonymous | NM_001118 | c.A125G | p.N42S |
| 447 | BLOOD | TTC16 | 9 | 130488631 | 130488631 | G | C | nonsynonymous | NM_144965 | c.G1373C | p.G458A |
| 447 | BLOOD | SKA3 | 13 | 21746799 | 21746799 | C | A | nonsynonymous | NM_145061 | c.G125T | p.R42I |
| 447 | BLOOD | MTUS2 | 13 | 29933450 | 29933450 | C | T | nonsynonymous | NM_001033602 | c.C2987T | p.P996L |
| 447 | BLOOD | RXFP2 | 13 | 32367033 | 32367033 | C | G | nonsynonymous | NM_130806 | c.C1594G | p.R532G |
| 447 | BLOOD | HERC2 | 15 | 28518046 | 28518046 | G | A | nonsynonymous | NM_004667 | c.C905T | p.T302M |
| 447 | BLOOD | XAB2 | 19 | 7694391 | 7694391 | G | C | nonsynonymous | NM_020196 | c.C23G | p.S8W |
| 447 | BLOOD | PNKP | 19 | 50370425 | 50370425 | C | G | nonsynonymous | NM_007254 | c.G37C | p.E13Q |
| 447 | BLOOD | ZNF814 | 19 | 58385546 | 58385546 | G | T | nonsynonymous | NM_001144989 | c.C1212A | p.D404E |
| 447 | BLOOD | PLXNB3 | X | 153041544 | 153041544 | T | C | nonsynonymous | NM_005393 | c.T4604C | p.M1535T |
| 448 | LUNG | ANP32E | 1 | 150199040 | 150199045 | TCCTCT | - | inframe | NM_001280559 | c.576_581del | p.192_194del |
| 448 | LUNG | FMN2 | 1 | 240370914 | 240370946 | GCCCCCTCTACCCGGAGCGGGAATACCTCCTCC | - | inframe | NM_001305424 | c.2814_2846del | p.938_949del |
| 448 | LUNG | ODF1 | 8 | 103573011 | 103573037 | TGCAACCCCTGCAGCCCCTGCAACCCG | - | inframe | NM_024410 | c.652_678del | p.218_226del |
| 448 | LUNG | TRPM3 | 9 | 73458045 | 73458045 | - | A | splicing |  |  |  |
| 448 | LUNG | GOLGA2 | 9 | 131020796 | 131020798 | CCT | - | inframe | NM_004486 | c.2144_2146del | p.715_716del |
| 448 | LUNG | FAM90A1 | 12 | 8374781 | 8374781 | - | ACG | inframe | NM_018088 | c.1031_1032insCGT | p.T344delinsTV |
| 448 | LUNG | HDGFRP2 | 19 | 4499633 | 4499647 | AGCTGGCCGGGGAGG | - | frameshift | NM_001001520 | c.1721_1722del | p.K574fs |
| 448 | LUNG | PIK3CD | 1 | 9776134 | 9776134 | G | A | nonsynonymous | NM_005026 | c.G598A | p.E200K |
| 448 | LUNG | RBMXL1 | 1 | 89449390 | 89449390 | T | C | nonsynonymous | NM_019610 | c.A120G | p.I40M |
| 448 | LUNG | CSRP1 | 1 | 201458071 | 201458071 | T | A | nonsynonymous | NM_001193572 | c.A323T | p.K108I |
| 448 | LUNG | FN1 | 2 | 216251538 | 216251538 | G | A | nonsynonymous | NM_212482 | c.C4486T | p.R1496W |
| 448 | LUNG | ZNF142 | 2 | 219521110 | 219521110 | C | T | nonsynonymous | NM_001105537 | c.G43A | p.G15R |
| 448 | LUNG | HACL1 | 3 | 15614707 | 15614707 | C | T | nonsynonymous | NM_012260 | c.G952A | p.A318T |
| 448 | LUNG | ZNF860 | 3 | 32030834 | 32030834 | C | T | nonsynonymous | NM_001137674 | c.C263T | p.T88I |
| 448 | LUNG | CMTM6 | 3 | 32544147 | 32544147 | T | C | nonsynonymous | NM_017801 | c.A91G | p.M31V |
| 448 | LUNG | NICN1 | 3 | 49462410 | 49462410 | T | C | nonsynonymous | NM_032316 | c.A572G | p.H191R |
| 448 | LUNG | FAM208A | 3 | 56695029 | 56695029 | C | A | nonsynonymous | NM_001112736 | c.G1177T | p.V393F |
| 448 | LUNG | ABI3BP | 3 | 100494075 | 100494075 | C | G | nonsynonymous | NM_015429 | c.G2305C | p.V769L |
| 448 | LUNG | TRPA1 | 8 | 72981337 | 72981337 | G | A | nonsynonymous | NM_007332 | c.C365T | p.A122V |
| 448 | LUNG | SPATA31C1 | 9 | 90537739 | 90537739 | C | T | nonsynonymous | NM_001145124 | c.C2917T | p.H973Y |
| 448 | LUNG | AKNA | 9 | 117105993 | 117105993 | T | A | nonsynonymous | NM_030767 | c.A3752T | p.D1251V |
| 448 | LUNG | LRRC8A | 9 | 131671142 | 131671142 | G | A | nonsynonymous | NM_001127244 | c.G1699A | p.V567M |
| 448 | LUNG | REXO4 | 9 | 136273917 | 136273917 | T | C | nonsynonymous | NM_001279350 | c.A638G | p.Y213C |
| 448 | LUNG | TMEM141 | 9 | 139686439 | 139686439 | G | C | nonsynonymous | NM_032928 | c.G162C | p.R54S |
| 448 | LUNG | MUC5B | 11 | 1276432 | 1276432 | G | T | nonsynonymous | NM_002458 | c.G15826T | p.V5276L |
| 448 | LUNG | CD163L1 | 12 | 7527284 | 7527284 | C | T | nonsynonymous | NM_174941 | c.G3163A | p.G1055S |
| 448 | LUNG | SRRM4 | 12 | 119540049 | 119540049 | C | T | nonsynonymous | NM_194286 | c.C140T | p.P47L |
| 448 | LUNG | MTUS2 | 13 | 29933450 | 29933450 | C | T | nonsynonymous | NM_001033602 | c.C2987T | p.P996L |
| 448 | LUNG | TRPM1 | 15 | 31294404 | 31294404 | G | A | nonsynonymous | NM_001252024 | c.C4499T | p.T1500M |
| 448 | LUNG | THSD4 | 15 | 72023502 | 72023502 | G | A | nonsynonymous | NM_024817 | c.G1576A | p.V526M |
| 448 | LUNG | SH2D7 | 15 | 78390886 | 78390886 | A | G | nonsynonymous | NM_001101404 | c.A593G | p.Q198R |
| 448 | LUNG | POLG | 15 | 89869871 | 89869871 | G | A | nonsynonymous | NM_001126131 | c.C1684T | p.R562W |
| 448 | LUNG | MAN2A2 | 15 | 91456607 | 91456607 | A | G | nonsynonymous | NM_006122 | c.A2689G | p.I897V |
| 448 | LUNG | OR1D5 | 17 | 2966273 | 2966273 | G | A | nonsynonymous | NM_014566 | c.C629T | p.P210L |
| 448 | LUNG | PPP2R3B | X | 322193 | 322193 | C | T | nonsynonymous | NM_013239 | c.G457A | p.A153T |
| 448 | LUNG | MXRA5 | X | 3235724 | 3235724 | C | T | nonsynonymous | NM_015419 | c.G5998A | p.G2000S |
| 448 | LUNG | MXRA5 | X | 3239545 | 3239545 | C | T | nonsynonymous | NM_015419 | c.G4181A | p.G1394D |
| 448 | LUNG | MXRA5 | X | 3241256 | 3241256 | T | C | nonsynonymous | NM_015419 | c.A2470G | p.I824V |
| 448 | LUNG | KAL1 | X | 8504833 | 8504833 | C | T | nonsynonymous | NM_000216 | c.G1600A | p.V534I |
| 448 | LUNG | TLR8 | X | 12924826 | 12924826 | A | G | nonsynonymous | NM_138636 | c.A1G | p.M1V |
| 448 | LUNG | MAGEB10 | X | 27839572 | 27839572 | T | C | nonsynonymous | NM_182506 | c.T149C | p.F50S |
| 448 | LUNG | DMD | X | 32503194 | 32503194 | T | C | nonsynonymous | NM_000109 | c.A2621G | p.D874G |
| 448 | LUNG | KDM6A | X | 44929077 | 44929077 | C | A | nonsynonymous | NM_021140 | c.C2177A | p.T726K,KDM6A |
| 448 | LUNG | CXorf36 | X | 45051111 | 45051111 | C | T | nonsynonymous | NM_176819 | c.G383A | p.R128K |
| 448 | LUNG | ZXDB | X | 57618870 | 57618870 | G | A | nonsynonymous | NM_007157 | c.G389A | p.G130D |
| 448 | LUNG | HDX | X | 83723541 | 83723541 | A | G | nonsynonymous | NM_001177479 | c.T1190C | p.F397S |
| 448 | LUNG | SATL1 | X | 84363140 | 84363140 | A | G | nonsynonymous | NM_001012980 | c.T835C | p.W279R |
| 448 | LUNG | CPXCR1 | X | 88008807 | 88008807 | G | A | nonsynonymous | NM_033048 | c.G392A | p.R131H |
| 448 | LUNG | TCEAL2 | X | 101382005 | 101382005 | G | C | nonsynonymous | NM_080390 | c.G203C | p.G68A |
| 448 | LUNG | RBMX | X | 135956408 | 135956408 | A | G | nonsynonymous | NM_002139 | c.T1069C | p.Y357H |
| 448 | LUNG | RBMX | X | 135956462 | 135956462 | G | C | nonsynonymous | NM_002139 | c.C1015G | p.R339G |
| 448 | LUNG | RBMX | X | 135956467 | 135956467 | C | T | nonsynonymous | NM_002139 | c.G1010A | p.S337N |
| 448 | LUNG | RBMX | X | 135956506 | 135956506 | C | G | nonsynonymous | NM_002139 | c.G971C | p.R324P |
| 448 | LUNG | GPR101 | X | 136113464 | 136113464 | C | A | nonsynonymous | NM_054021 | c.G370T | p.V124L |
| 448 | LUNG | MAGEC1 | X | 140994381 | 140994381 | C | G | nonsynonymous | NM_005462 | c.C1191G | p.H397Q |
| 449 | LUNG | RB1 | 13 | 48939088 | 48939088 | C | T | nonsynonymous | NM_000321 | c.C920T | p.T307I |
| 449 | LUNG | KRTAP4-8 | 17 | 39253953 | 39253953 | G | T | nonsynonymous | NM_031960 | c.C384A | p.S128R |
| 449 | LUNG | TCEB3B | 18 | 44561481 | 44561481 | G | A | nonsynonymous | NM_016427 | c.C155T | p.T52M |
| 449 | LUNG | ZNF814 | 19 | 58385748 | 58385748 | G | A | nonsynonymous | NM_001144989 | c.C1010T | p.A337V |
| 449 | LUNG | ZXDB | X | 57618849 | 57618849 | A | C | nonsynonymous | NM_007157 | c.A368C | p.E123A |
| 450 | LUNG | DMKN | 19 | 36002386 | 36002386 | C | T | nonsynonymous | NM_033317 | c.G845A | p.S282N |
| 451 | BLOOD | RUNX2 | 6 | 45390487 | 45390504 | GGCGGCGGCGGCGGCTGC | - | inframe | NM_001024630 | c.216_233del | p.72_78del |
| 451 | BLOOD | SLC5A10 | 17 | 18918510 | 18918510 | - | CGGTACGGGGGTGGGGGC | inframe | NM_001042450 | c.1239_1240insCGGTACGGGGGTGGGGGC | p.G413delinsGRYGGGG |
| 451 | BLOOD | IRF6 | 1 | 209961970 | 209961970 | C | G | nonsynonymous | NM_006147 | c.G1199C | p.R400P |
| 451 | BLOOD | SELPLG | 12 | 109017672 | 109017672 | G | C | nonsynonymous | NM_003006 | c.C412G | p.P138A |
| 451 | BLOOD | MYO9B | 19 | 17286535 | 17286535 | C | T | nonsynonymous | NM_004145 | c.C2204T | p.S735L |
| 453 | BLOOD | ARID4B | 1 | 235383263 | 235383263 | - | A | frameshift | NM_016374 | c.1427dupT | p.L476fs |
| 453 | BLOOD | NUF2 | 1 | 163317691 | 163317691 | C | T | nonsynonymous | NM_145697 | c.C1087T | p.H363Y |
| 453 | BLOOD | FBXO28 | 1 | 224321776 | 224321776 | G | T | nonsynonymous | NM_015176 | c.G378T | p.R126S |
| 453 | BLOOD | FBXO28 | 1 | 224321780 | 224321780 | G | A | nonsynonymous | NM_015176 | c.G382A | p.E128K |
| 453 | BLOOD | FBXO28 | 1 | 224321782 | 224321782 | G | C | nonsynonymous | NM_015176 | c.G384C | p.E128D |
| 453 | BLOOD | EXO1 | 1 | 242020734 | 242020734 | C | A | nonsynonymous | NM_130398 | c.C493A | p.Q165K |
| 453 | BLOOD | TET1 | 10 | 70404633 | 70404633 | C | T | nonsynonymous | NM_030625 | c.C2147T | p.S716L |
| 453 | BLOOD | CLIP1 | 12 | 122812697 | 122812697 | C | T | nonsynonymous | NM_002956 | c.G3013A | p.E1005K |
| 453 | BLOOD | SLAIN1 | 13 | 78318644 | 78318644 | C | A | nonsynonymous | NM_001242871 | c.C51A | p.F17L |
| 453 | BLOOD | ETFA | 15 | 76578041 | 76578041 | C | A | nonsynonymous | NM_000126 | c.G601T | p.D201Y |
| 453 | BLOOD | MYLK3 | 16 | 46744689 | 46744689 | C | A | nonsynonymous | NM_182493 | c.G2127T | p.L709F |
| 453 | BLOOD | EIF2S3 | X | 24073777 | 24073777 | C | A | nonsynonymous | NM_001415 | c.C115A | p.Q39K |
| 454 | BLOOD | PCDH12 | 5 | 141324955 | 141324955 | - | CTGCTGCTG | inframe | NM_016580 | c.3545_3546insCAGCAGCAG | p.R1182delinsSSSR |
| 454 | BLOOD | CLEC17A | 19 | 14694175 | 14694175 | - | GGA | inframe | NM_001204118 | c.50_51insGGA | p.M17delinsME |
| 454 | BLOOD | TMEM82 | 1 | 16070895 | 16070895 | C | A | nonsynonymous | NM_001013641 | c.C577A | p.L193M |
| 454 | BLOOD | PCDHA9 | 5 | 140228604 | 140228604 | A | G | nonsynonymous | NM_031857 | c.A524G | p.N175S |
| 454 | BLOOD | HLA-DQB2 | 6 | 32725559 | 32725559 | C | T | nonsynonymous | NM_001300790 | c.G748A | p.G250S |
| 454 | BLOOD | ACO1 | 9 | 32429452 | 32429452 | T | G | nonsynonymous | NM_002197 | c.T1520G | p.I507S |
| 454 | BLOOD | MMS19 | 10 | 99236625 | 99236625 | C | T | nonsynonymous | NM_001289404 | c.G115A | p.V39I |
| 454 | BLOOD | DTX4 | 11 | 58956761 | 58956761 | C | A | nonsynonymous | NM_015177 | c.C1124A | p.T375K |
| 454 | BLOOD | BSX | 11 | 122852349 | 122852349 | G | C | nonsynonymous | NM_001098169 | c.C31G | p.P11A |
| 454 | BLOOD | THSD1 | 13 | 52971687 | 52971687 | A | G | nonsynonymous | NM_018676 | c.T701C | p.I234T |
| 454 | BLOOD | PER1 | 17 | 8050683 | 8050683 | C | G | nonsynonymous | NM_002616 | c.G1514C | p.S505T |
| 454 | BLOOD | TCF3 | 19 | 1620975 | 1620975 | C | T | nonsynonymous | NM_003200 | c.G1085A | p.G362D |
| 454 | BLOOD | ZNF823 | 19 | 11833390 | 11833390 | C | T | nonsynonymous | NM_001080493 | c.G959A | p.R320Q |
| 454 | BLOOD | CYP4F11 | 19 | 16038038 | 16038038 | C | T | nonsynonymous | NM_021187 | c.G509A | p.S170N |
| 454 | BLOOD | GATAD2A | 19 | 19611937 | 19611937 | G | A | nonsynonymous | NM_017660 | c.G1212A | p.M404I |
| 454 | BLOOD | FCGBP | 19 | 40392802 | 40392802 | C | T | nonsynonymous | NM_003890 | c.G7702A | p.A2568T |
| 454 | BLOOD | IL4I1 | 19 | 50393238 | 50393238 | C | T | nonsynonymous | NM_152899 | c.G1393A | p.E465K |
| 454 | BLOOD | KLK14 | 19 | 51582808 | 51582808 | G | A | nonsynonymous | NM_022046 | c.C412T | p.R138W |
| 454 | BLOOD | PPP2R3B | X | 299360 | 299360 | G | A | nonsynonymous | NM_013239 | c.C1556T | p.A519V |
| 454 | BLOOD | IL3RA | X | 1497644 | 1497644 | G | C | nonsynonymous | NM_002183 | c.G967C | p.V323L |
| 454 | BLOOD | WDR44 | X | 117528056 | 117528056 | G | A | nonsynonymous | NM_019045 | c.G865A | p.A289T |
| 455 | BLOOD | KRT3 | 12 | 53189414 | 53189431 | CCAAAGCCACCAGCCCCT | - | inframe | NM_057088 | c.396_413del | p.132_138del |
| 455 | BLOOD | GOLGA6L10 | 15 | 82635194 | 82635194 | T | C | nonsynonymous | NM_001164465 | c.A1505G | p.E502G |
| 456 | BLOOD | FAM90A1 | 12 | 8374781 | 8374781 | - | ACG | inframe | NM_018088 | c.1031_1032insCGT | p.T344delinsTV |
| 456 | BLOOD | ANKRD36 | 2 | 97877440 | 97877440 | T | C | nonsynonymous | NM_001164315 | c.T3431C | p.M1144T |
| 456 | BLOOD | BRCC3 | X | 154305532 | 154305532 | G | T | stopgain | NM_024332 | c.G283T | p.E95X |
| 457 | BLOOD | FAM90A1 | 12 | 8374781 | 8374781 | - | ACG | inframe | NM_018088 | c.1031_1032insCGT | p.T344delinsTV |
| 457 | BLOOD | FSCB | 14 | 44974154 | 44974154 | - | GGGGCCTCCTCAGCTGGTGGAGGCTGAACTTCAGAG | inframe | NM_032135 | c.2036_2037insCTCTGAAGTTCAGCCTCCACCAGCTGAGGAGGCCCC | p.P679delinsPSEVQPPPAEEAP |
| 457 | BLOOD | HDGFRP2 | 19 | 4499633 | 4499647 | AGCTGGCCGGGGAGG | - | frameshift | NM_001001520 | c.1721_1722del | p.K574fs |
| 457 | BLOOD | MUC4 | 3 | 195515093 | 195515093 | G | T | nonsynonymous | NM_018406 | c.C3358A | p.P1120T |
| 458 | BLOOD | FOXL2 | 3 | 138664804 | 138664804 | G | T | stopgain | NM_023067 | c.C761A | p.S254X |
| 458 | BLOOD | CEL | 9 | 135947032 | 135947032 | C | A | nonsynonymous | NM_001807 | c.C2152A | p.P718T |
| 458 | BLOOD | PTF1A | 10 | 23482235 | 23482235 | G | T | nonsynonymous | NM_178161 | c.G776T | p.R259L |
| 458 | BLOOD | MYO7A | 11 | 76913345 | 76913345 | G | T | nonsynonymous | NM_000260 | c.G5044T | p.A1682S,MYO7A |
| 458 | BLOOD | FSCN2 | 17 | 79496099 | 79496099 | G | T | nonsynonymous | NM_012418 | c.G542T | p.S181I |
| 459 | BLOOD | SKA3 | 13 | 21746601 | 21746601 | G | - | frameshift | NM_145061 | c.208delC | p.Q70fs |
| 459 | BLOOD | SOGA3 | 6 | 127837125 | 127837125 | T | C | nonsynonymous | NM_001012279 | c.A635G | p.E212G |
| 459 | BLOOD | GOLGA6L10 | 15 | 82635194 | 82635194 | T | C | nonsynonymous | NM_001164465 | c.A1505G | p.E502G |
| 461 | BLOOD | ZSWIM6 | 5 | 60628422 | 60628422 | G | A | nonsynonymous | NM_020928 | c.G323A | p.R108H |
| 461 | BLOOD | TCEB3C,TCEB3CL | 18 | 44555312 | 44555312 | G | C | nonsynonymous | NM_145653 | c.C902G | p.S301C |
| 462 | BLOOD | ANKRD36 | 2 | 97877478 | 97877478 | G | A | nonsynonymous | NM_001164315 | c.G3469A | p.V1157M |
| 462 | BLOOD | ZNF717 | 3 | 75790880 | 75790880 | A | G | nonsynonymous | NM_001290210 | c.T65C | p.V22A |
| 463 | BLOOD | ATG2A | 11 | 64678098 | 64678098 | C | T | nonsynonymous | NM_015104 | c.G1697A | p.R566H |
| 463 | BLOOD | NCOR1 | 17 | 16097870 | 16097870 | C | A | nonsynonymous | NM_006311 | c.G14T | p.G5V |
| 464 | BLOOD | ANKRD36 | 2 | 97868086 | 97868086 | T | C | nonsynonymous | NM_001164315 | c.T2915C | p.L972S |
| 464 | BLOOD | BDP1 | 5 | 70812052 | 70812052 | G | T | nonsynonymous | NM_018429 | c.G4814T | p.R1605I |
| 464 | BLOOD | TH | 11 | 2191097 | 2191097 | G | T | nonsynonymous | NM_199292 | c.C188A | p.P63Q |
| 464 | BLOOD | FAT3 | 11 | 92616488 | 92616488 | T | C | nonsynonymous | NM_001008781 | c.T12866C | p.L4289P |
| 464 | BLOOD | GAS6 | 13 | 114537583 | 114537583 | T | G | nonsynonymous | NM_000820 | c.A775C | p.T259P |
| 464 | BLOOD | SLC35G4 | 18 | 11610024 | 11610024 | A | G | nonsynonymous | NM_001282300 | c.A430G | p.I144V |
| 464 | BLOOD | ARHGAP6 | X | 11157535 | 11157535 | G | C | nonsynonymous | NM_013427 | c.C2373G | p.D791E |
| 465 | BLOOD | IL17RC | 3 | 9959221 | 9959221 | - | TCTGGTCTT | inframe | NM_013427 | c.C2373G | p.D791E |
| 465 | BLOOD | GOLIM4 | 3 | 167742354 | 167742356 | TCC | - | inframe | NM_014498 | c.1818_1820del | p.606_607del |
| 465 | BLOOD | IRF5 | 7 | 128587352 | 128587381 | ACTCTGCAGCCGCCCACTCTGCGGCCGCCT | - | inframe | NM_001098630 | c.502_531del | p.168_177del |
| 465 | BLOOD | TNKS2 | 10 | 93601945 | 93601945 | - | A | frameshift | NM_025235 | c.1857dupA | p.T619fs |
| 465 | BLOOD | CTAGE5 | 14 | 39784005 | 39784006 | TA | - | splicing |  |  |  |
| 465 | BLOOD | RIN3 | 14 | 93154538 | 93154540 | GGC | - | inframe | NM_024832 | c.2899_2901del | p.967_967del |
| 465 | BLOOD | BCL6B | 17 | 6928019 | 6928019 | - | CAG | inframe | NM_181844 | c.701_702insCAG | p.S234delinsSS |
| 465 | BLOOD | HDGFRP2 | 19 | 4499633 | 4499647 | AGCTGGCCGGGGAGG | - | frameshift | NM_001001520 | c.1721_1722del | p.K574fs |
| 465 | BLOOD | ZNF772 | 19 | 57988666 | 57988666 | - | GCC | inframe | NM_001024596 | c.11_12insGGC | p.A4delinsAA |
| 465 | BLOOD | SDC3 | 1 | 31351560 | 31351560 | C | G | nonsynonymous | NM_014654 | c.G166C | p.E56Q |
| 465 | BLOOD | ADGRB2 | 1 | 32222347 | 32222347 | T | C | nonsynonymous | NM_001294335 | c.A91G | p.T31A |
| 465 | BLOOD | GJB3 | 1 | 35250428 | 35250428 | G | A | nonsynonymous | NM_024009 | c.G65A | p.R22H |
| 465 | BLOOD | RHBDL2 | 1 | 39384989 | 39384989 | G | C | nonsynonymous | NM_001304746 | c.C136G | p.R46G |
| 465 | BLOOD | COL11A1 | 1 | 103548505 | 103548505 | C | T | nonsynonymous | NM_001854 | c.G130A | p.A44T |
| 465 | BLOOD | CSF1 | 1 | 110466397 | 110466397 | A | G | nonsynonymous | NM_000757 | c.A1154G | p.Q385R |
| 465 | BLOOD | DISC1 | 1 | 231830492 | 231830492 | C | T | nonsynonymous | NM_001164542 | c.C988T | p.L330F |
| 465 | BLOOD | NTPCR | 1 | 233091465 | 233091465 | G | A | nonsynonymous | NM_032324 | c.G197A | p.G66E |
| 465 | BLOOD | LYST | 1 | 235969077 | 235969077 | C | A | nonsynonymous | NM_000081 | c.G3359T | p.S1120I |
| 465 | BLOOD | NID1 | 1 | 236208869 | 236208869 | G | T | nonsynonymous | NM_002508 | c.C640A | p.Q214K |
| 465 | BLOOD | PXDN | 2 | 1652488 | 1652488 | C | T | nonsynonymous | NM_012293 | c.G3064A | p.V1022M |
| 465 | BLOOD | ANKRD36B | 2 | 98169816 | 98169816 | T | G | nonsynonymous | NM_025190 | c.A1190C | p.Q397P |
| 465 | BLOOD | MYO7B | 2 | 128347689 | 128347689 | C | A | nonsynonymous | NM_001080527 | c.C1877A | p.P626H |
| 465 | BLOOD | RAB6C | 2 | 130738031 | 130738031 | A | G | nonsynonymous | NM_032144 | c.A343G | p.R115G |
| 465 | BLOOD | XIRP2 | 2 | 168106796 | 168106796 | A | G | nonsynonymous | NM_152381 | c.A8894G | p.E2965G |
| 465 | BLOOD | TTN | 2 | 179436796 | 179436796 | C | T | nonsynonymous | NM_001267550 | c.G74063A | p.R24688H |
| 465 | BLOOD | TTN | 2 | 179599653 | 179599653 | G | A | nonsynonymous | NM_001267550 | c.C14998T | p.R5000C |
| 465 | BLOOD | ABCB6 | 2 | 220078564 | 220078564 | G | C | nonsynonymous | NM_005689 | c.C1562G | p.T521S |
| 465 | BLOOD | OBSL1 | 2 | 220419340 | 220419340 | G | A | stopgain | NM_015311 | c.C4732T | p.Q1578X |
| 465 | BLOOD | MOGAT1 | 2 | 223553156 | 223553156 | A | T | nonsynonymous | NM_058165 | c.A188T | p.H63L |
| 465 | BLOOD | HACL1 | 3 | 15606131 | 15606131 | T | C | nonsynonymous | NM_012260 | c.A1444G | p.N482D |
| 465 | BLOOD | ACVR2B | 3 | 38518844 | 38518844 | G | A | nonsynonymous | NM_001106 | c.G119A | p.R40H |
| 465 | BLOOD | CCDC13 | 3 | 42754660 | 42754660 | T | C | nonsynonymous | NM_144719 | c.A1867G | p.K623E |
| 465 | BLOOD | CCDC66 | 3 | 56605311 | 56605311 | A | G | nonsynonymous | NM_001141947 | c.A917G | p.Q306R |
| 465 | BLOOD | OR5AC2 | 3 | 97806251 | 97806251 | A | G | nonsynonymous | NM_054106 | c.A235G | p.I79V |
| 465 | BLOOD | MYH15 | 3 | 108147680 | 108147680 | G | A | stopgain | NM_014981 | c.C3421T | p.R1141X |
| 465 | BLOOD | AGTR1 | 3 | 148458977 | 148458977 | T | G | nonsynonymous | NM_009585 | c.T155G | p.V52G |
| 465 | BLOOD | COMMD2 | 3 | 149459406 | 149459406 | C | G | nonsynonymous | NM_016094 | c.G502C | p.D168H |
| 465 | BLOOD | ERICH6 | 3 | 150398311 | 150398311 | C | A | nonsynonymous | NM_152394 | c.G1055T | p.R352L |
| 465 | BLOOD | ZBBX | 3 | 167045759 | 167045759 | T | C | nonsynonymous | NM_024687 | c.A833G | p.K278R |
| 465 | BLOOD | HTR3C | 3 | 183778117 | 183778117 | G | A | nonsynonymous | NM_130770 | c.G1321A | p.V441I |
| 465 | BLOOD | CRIPAK | 4 | 1388944 | 1388944 | G | A | nonsynonymous | NM_175918 | c.G645A | p.M215I |
| 465 | BLOOD | PTPN13 | 4 | 87662871 | 87662871 | G | A | nonsynonymous | NM_080683 | c.G2389A | p.V797I |
| 465 | BLOOD | FBXW7 | 4 | 153332525 | 153332525 | G | C | nonsynonymous | NM_033632 | c.C431G | p.T144R |
| 465 | BLOOD | C4orf46 | 4 | 159590838 | 159590838 | T | C | nonsynonymous | NM_001008393 | c.A269G | p.E90G |
| 465 | BLOOD | C4orf46 | 4 | 159590839 | 159590839 | C | T | nonsynonymous | NM_001008393 | c.G268A | p.E90K |
| 465 | BLOOD | C4orf46 | 4 | 159590841 | 159590841 | G | T | nonsynonymous | NM_001008393 | c.C266A | p.T89K |
| 465 | BLOOD | PCDHA4 | 5 | 140187869 | 140187869 | G | C | nonsynonymous | NM_018907 | c.G1097C | p.G366A |
| 465 | BLOOD | PCDHA9 | 5 | 140230137 | 140230137 | C | A | nonsynonymous | NM_031857 | c.C2057A | p.A686D |
| 465 | BLOOD | SOX30 | 5 | 157053521 | 157053521 | A | G | nonsynonymous | NM_178424 | c.T2089C | p.Y697H |
| 465 | BLOOD | HIVEP1 | 6 | 12124679 | 12124679 | A | G | nonsynonymous | NM_002114 | c.A4651G | p.K1551E |
| 465 | BLOOD | ZFP57 | 6 | 29641514 | 29641514 | C | T | nonsynonymous | NM_001109809 | c.G374A | p.R125Q |
| 465 | BLOOD | KCNK17 | 6 | 39267246 | 39267246 | A | G | nonsynonymous | NM_031460 | c.T956C | p.L319P |
| 465 | BLOOD | LRRC73 | 6 | 43475272 | 43475272 | C | A | nonsynonymous | NM_001012974 | c.G802T | p.G268C |
| 465 | BLOOD | ORC3 | 6 | 88346168 | 88346168 | A | T | nonsynonymous | NM_012381 | c.A1346T | p.D449V |
| 465 | BLOOD | ASCC3 | 6 | 101163360 | 101163360 | T | C | nonsynonymous | NM_006828 | c.A2129G | p.K710R |
| 465 | BLOOD | QRSL1 | 6 | 107103496 | 107103496 | G | A | nonsynonymous | NM_018292 | c.G1049A | p.R350K |
| 465 | BLOOD | MICAL1 | 6 | 109767059 | 109767059 | A | G | nonsynonymous | NM_022765 | c.T2588C | p.V863A |
| 465 | BLOOD | CCDC136 | 7 | 128445459 | 128445459 | A | G | nonsynonymous | NM_022742 | c.A829G | p.M277V |
| 465 | BLOOD | CLCN1 | 7 | 143048784 | 143048784 | G | A | nonsynonymous | NM_000083 | c.G2693A | p.G898E |
| 465 | BLOOD | ZNF777 | 7 | 149152860 | 149152860 | G | A | nonsynonymous | NM_015694 | c.C254T | p.A85V |
| 465 | BLOOD | SSPO | 7 | 149519022 | 149519022 | C | T | stopgain | NM_198455 | c.C12826T | p.R4276X |
| 465 | BLOOD | NUB1 | 7 | 151064102 | 151064102 | G | A | nonsynonymous | NM_001243351 | c.G950A | p.R317H |
| 465 | BLOOD | PROSC | 8 | 37620247 | 37620247 | G | A | nonsynonymous | NM_007198 | c.G70A | p.V24M |
| 465 | BLOOD | IDO2 | 8 | 39847312 | 39847312 | A | T | stopgain | NM_194294 | c.A661T | p.R221X |
| 465 | BLOOD | JPH1 | 8 | 75157149 | 75157149 | G | A | nonsynonymous | NM_020647 | c.C1520T | p.T507M |
| 465 | BLOOD | PKHD1L1 | 8 | 110451125 | 110451125 | G | A | splicing |  |  |  |
| 465 | BLOOD | PTAR1 | 9 | 72349104 | 72349104 | T | G | nonsynonymous | NM_001099666 | c.A390C | p.L130F |
| 465 | BLOOD | RIC8A | 11 | 212840 | 212840 | C | T | nonsynonymous | NM_001286134 | c.C1214T | p.P405L |
| 465 | BLOOD | WNK1 | 12 | 1005586 | 1005586 | C | T | nonsynonymous | NM_018979 | c.C5933T | p.S1978F |
| 465 | BLOOD | PLEKHG6 | 12 | 6426536 | 6426536 | G | A | nonsynonymous | NM_001144856 | c.G824A | p.R275Q |
| 465 | BLOOD | PHC1 | 12 | 9090424 | 9090424 | G | T | splicing |  |  |  |
| 465 | BLOOD | PZP | 12 | 9309819 | 9309819 | G | A | stopgain | NM_002864 | c.C3502T | p.Q1168X |
| 465 | BLOOD | PZP | 12 | 9309881 | 9309881 | C | T | nonsynonymous | NM_002864 | c.G3440A | p.G1147E |
| 465 | BLOOD | STYK1 | 12 | 10774490 | 10774490 | C | G | nonsynonymous | NM_018423 | c.G1049C | p.S350T |
| 465 | BLOOD | DDX47 | 12 | 12982368 | 12982368 | G | C | nonsynonymous | NM_016355 | c.G1248C | p.E416D |
| 465 | BLOOD | ADCY6 | 12 | 49177117 | 49177117 | C | T | nonsynonymous | NM_015270 | c.G101A | p.R34Q |
| 465 | BLOOD | RACGAP1 | 12 | 50400395 | 50400395 | A | T | nonsynonymous | NM_013277 | c.T110A | p.F37Y |
| 465 | BLOOD | APOF | 12 | 56755393 | 56755393 | C | G | nonsynonymous | NM_001638 | c.G597C | p.L199F |
| 465 | BLOOD | UTP20 | 12 | 101685838 | 101685838 | A | T | nonsynonymous | NM_014503 | c.A1129T | p.N377Y |
| 465 | BLOOD | HEATR5A | 14 | 31771546 | 31771546 | G | A | nonsynonymous | NM_015473 | c.C5419T | p.L1807F |
| 465 | BLOOD | ABCD4 | 14 | 74759065 | 74759065 | C | T | nonsynonymous | NM_005050 | c.G1043A | p.R348Q |
| 465 | BLOOD | UNC79 | 14 | 94008977 | 94008977 | G | A | nonsynonymous | NM_020818 | c.G1159A | p.V387I |
| 465 | BLOOD | MARK3 | 14 | 103871488 | 103871488 | A | G | nonsynonymous | NM_001128918 | c.A127G | p.I43V |
| 465 | BLOOD | BAG5 | 14 | 104028334 | 104028334 | T | C | nonsynonymous | NM_001015049 | c.A11G | p.H4R |
| 465 | BLOOD | CEP170B | 14 | 105349142 | 105349142 | G | A | nonsynonymous | NM_001112726 | c.G550A | p.A184T |
| 465 | BLOOD | OR1D5 | 17 | 2966273 | 2966273 | G | A | nonsynonymous | NM_014566 | c.C629T | p.P210L |
| 465 | BLOOD | P2RX5 | 17 | 3583059 | 3583059 | G | A | stopgain | NM_002561 | c.C1084T | p.Q362X |
| 465 | BLOOD | RABEP1 | 17 | 5276643 | 5276643 | A | G | nonsynonymous | NM_001291581 | c.A1790G | p.E597G |
| 465 | BLOOD | CDRT1 | 17 | 15508676 | 15508676 | G | A | stopgain | NM_006382 | c.C1294T | p.R432X |
| 465 | BLOOD | RAI1 | 17 | 17700053 | 17700053 | A | G | nonsynonymous | NM_030665 | c.A3791G | p.E1264G |
| 465 | BLOOD | LRRC48 | 17 | 17881007 | 17881007 | G | A | nonsynonymous | NM_001130090 | c.G95A | p.G32E |
| 465 | BLOOD | FBXW10 | 17 | 18654358 | 18654358 | A | G | nonsynonymous | NM_001267585 | c.A1114G | p.R372G |
| 465 | BLOOD | MMP28 | 17 | 34106309 | 34106309 | C | T | nonsynonymous | NM_001032278 | c.G130A | p.G44R |
| 465 | BLOOD | APBA3 | 19 | 3759751 | 3759751 | G | T | nonsynonymous | NM_004886 | c.C512A | p.S171Y |
| 465 | BLOOD | MYO1F | 19 | 8615058 | 8615058 | C | T | nonsynonymous | NM_012335 | c.G1087A | p.D363N |
| 465 | BLOOD | COL5A3 | 19 | 10106259 | 10106259 | G | A | nonsynonymous | NM_015719 | c.C1568T | p.P523L |
| 465 | BLOOD | ZNF787 | 19 | 56599432 | 56599432 | G | C | nonsynonymous | NM_001002836 | c.C1109G | p.A370G |
| 465 | BLOOD | NDUFV3 | 21 | 44324329 | 44324329 | G | A | nonsynonymous | NM_021075 | c.G1207A | p.E403K |
| 465 | BLOOD | CSF2RA | X | 1424548 | 1424548 | G | A | nonsynonymous | NM_001161531 | c.G1153A | p.G385S |
| 465 | BLOOD | MXRA5 | X | 3235724 | 3235724 | C | T | nonsynonymous | NM_015419 | c.G5998A | p.G2000S |
| 465 | BLOOD | LANCL3 | X | 37527659 | 37527659 | T | C | nonsynonymous | NM_198511 | c.T1142C | p.L381P |
| 465 | BLOOD | CFP | X | 47486695 | 47486695 | G | A | nonsynonymous | NM_001145252 | c.C611T | p.P204L |
| 466 | BLOOD | ESCO2 | 8 | 27633987 | 27633991 | TTTTG | - | frameshift | NM_001017420 | c.162_166del | p.H54fs |
| 466 | BLOOD | DEFB132 | 20 | 238436 | 238441 | TGGTCT | - | inframe | NM_207469 | c.17_22del | p.6_8del |
| 466 | BLOOD | FAM179A | 2 | 29240131 | 29240131 | T | C | nonsynonymous | NM_199280 | c.T1156C | p.S386P |
| 466 | BLOOD | FAM179A | 2 | 29240132 | 29240132 | C | T | nonsynonymous | NM_199280 | c.C1157T | p.S386F |
| 466 | BLOOD | PCDHB10 | 5 | 140573600 | 140573600 | C | G | nonsynonymous | NM_018930 | c.C1475G | p.P492R |
| 466 | BLOOD | COL11A2 | 6 | 33132720 | 33132720 | T | C | nonsynonymous | NM_080681 | c.A4514G | p.N1505S |
| 466 | BLOOD | DMBT1 | 10 | 124392398 | 124392398 | G | T | nonsynonymous | NM_017579 | c.G5972T | p.R1991L |
| 466 | BLOOD | ITGA7 | 12 | 56101421 | 56101421 | A | C | nonsynonymous | NM_001144996 | c.T46G | p.C16G |
| 466 | BLOOD | PSTPIP1 | 15 | 77320931 | 77320931 | G | C | nonsynonymous | NM_003978 | c.G454C | p.D152H |
| 466 | BLOOD | RSPH6A | 19 | 46307977 | 46307977 | C | G | nonsynonymous | NM_030785 | c.G1186C | p.E396Q |
| 466 | BLOOD | RIN2 | 20 | 19937447 | 19937447 | C | T | nonsynonymous | NM_001242581 | c.C494T | p.P165L |
| 466 | BLOOD | RIPK4 | 21 | 43187195 | 43187195 | C | T | nonsynonymous | NM_020639 | c.G7A | p.G3S |
| 466 | BLOOD | RBMX | X | 135957672 | 135957672 | A | T | nonsynonymous | NM_002139 | c.T614A | p.V205D |
| 467 | BLOOD | IRF5 | 7 | 128587352 | 128587381 | ACTCTGCAGCCGCCCACTCTGCGGCCGCCT | - | inframe | NM_001098630 | c.502_531del | p.168_177del |
| 467 | BLOOD | CREB3L2 | 7 | 137612914 | 137612916 | TGG | - | inframe | NM_194071 | c.299_301del | p.100_101del |
| 467 | BLOOD | HSPBP1 | 19 | 55790886 | 55790886 | - | GCCGCCGCC | inframe | NM_001130106 | c.90_91insGGCGGCGGC | p.S31delinsGGGS |
| 467 | BLOOD | UBXN11 | 1 | 26608849 | 26608849 | T | A | nonsynonymous | NM_183008 | c.A1504T | p.S502C |
| 467 | BLOOD | TRMT1L | 1 | 185119609 | 185119609 | C | A | nonsynonymous | NM_030934 | c.G362T | p.C121F |
| 467 | BLOOD | MCUR1 | 6 | 13814624 | 13814624 | C | T | nonsynonymous | NM_001031713 | c.G38A | p.R13H |
| 467 | BLOOD | TCP11L1 | 11 | 33087551 | 33087551 | A | C | nonsynonymous | NM_018393 | c.A1148C | p.H383P |
| 467 | BLOOD | CCNA1 | 13 | 37012866 | 37012866 | T | G | nonsynonymous | NM_003914 | c.T755G | p.V252G |
| 467 | BLOOD | ETFA | 15 | 76578041 | 76578041 | C | A | nonsynonymous | NM_000126 | c.G601T | p.D201Y |
| 467 | BLOOD | TPSAB1 | 16 | 1291622 | 1291622 | A | G | nonsynonymous | NM_003294 | c.A421G | p.T141A |
| 467 | BLOOD | ZCCHC14 | 16 | 87448079 | 87448079 | C | G | nonsynonymous | NM_015144 | c.G1133C | p.R378P |
| 467 | BLOOD | PNKP | 19 | 50370425 | 50370425 | C | G | nonsynonymous | NM_007254 | c.G37C | p.E13Q |
| 467 | BLOOD | CLTCL1 | 22 | 19213150 | 19213150 | C | A | nonsynonymous | NM_007098 | c.G1954T | p.V652F |
| 467 | BLOOD | NCF4 | 22 | 37260160 | 37260160 | A | C | nonsynonymous | NM_000631 | c.A106C | p.T36P |
| 468 | BLOOD | NPIPB5 | 16 | 22545744 | 22545755 | TCCACCCTCAGC | - | inframe | NM_001135865 | c.1440_1451del | p.480_484del |
| 468 | BLOOD | ATXN3 | 14 | 92537379 | 92537379 | T | C | nonsynonymous | NM_001164782 | c.A43G | p.T15A,ATXN3 |
| 469 | BLOOD | SLC46A2 | 9 | 115651904 | 115651904 | A | G | nonsynonymous | NM_033051 | c.T1058C | p.I353T |
| 469 | BLOOD | SLC2A6 | 9 | 136338603 | 136338603 | G | A | nonsynonymous | NM_017585 | c.C1156T | p.P386S |
| 469 | BLOOD | MYT1 | 20 | 62839371 | 62839371 | T | A | nonsynonymous | NM_004535 | c.T822A | p.D274E |
| 470 | BLOOD | MAGEF1 | 3 | 184429133 | 184429133 | - | TCC | inframe | NM_022149 | c.476_477insGGA | p.D159delinsED |
| 470 | BLOOD | OR13C2 | 9 | 107367393 | 107367396 | GTTA | - | frameshift | NM_001004481 | c.513_516del | p.N171fs |
| 470 | BLOOD | RBM23 | 14 | 23371265 | 23371265 | - | GCA | inframe | NM_001077351 | c.1169_1170insTGC | p.A390delinsAA |
| 470 | BLOOD | CRTAP | 3 | 33155657 | 33155657 | C | A | nonsynonymous | NM_006371 | c.C88A | p.R30S |
| 470 | BLOOD | RP1L1 | 8 | 10467605 | 10467605 | C | T | nonsynonymous | NM_178857 | c.G4003A | p.G1335R |
| 470 | BLOOD | FAM86B2 | 8 | 12287914 | 12287914 | A | G | nonsynonymous | NM_001137610 | c.T287C | p.L96P |
| 470 | BLOOD | HRCT1 | 9 | 35906607 | 35906607 | G | A | nonsynonymous | NM_001039792 | c.G323A | p.R108H |
| 470 | BLOOD | MAMDC4 | 9 | 139749099 | 139749099 | G | A | nonsynonymous | NM_206920 | c.G905A | p.R302H |
| 470 | BLOOD | MADCAM1 | 19 | 501786 | 501786 | C | A | nonsynonymous | NM_130760 | c.C785A | p.P262Q |
| 470 | BLOOD | CT45A10 | X | 134948078 | 134948078 | C | T | nonsynonymous | NM_001291527 | c.G247A | p.G83R,CT45A10 |
| 470 | BLOOD | PLXNB3 | X | 153035798 | 153035798 | G | A | nonsynonymous | NM_005393 | c.G1792A | p.V598I |
| 471 | BLOOD | NAB2 | 12 | 57485446 | 57485446 | T | C | nonsynonymous | NM_005967 | c.T622C | p.F208L |
| 472 | BLOOD | OR12D2 | 6 | 29365197 | 29365197 | G | - | frameshift | NM_013936 | c.721delG | p.A241fs |
| 472 | BLOOD | MYCT1 | 6 | 153019100 | 153019103 | AGAT | - | frameshift | NM_025107 | c.63_66del | p.R21fs |
| 473 | BLOOD | PRR21 | 2 | 240982219 | 240982219 | A | G | nonsynonymous | NM_001080835 | c.T181C | p.S61P |
| 473 | BLOOD | VEGFB | 11 | 64005040 | 64005040 | A | C | nonsynonymous | NM_003377 | c.A559C | p.T187P |
| 473 | BLOOD | TPSB2 | 16 | 1279438 | 1279438 | C | T | nonsynonymous | NM_024164 | c.G253A | p.A85T |
| 473 | BLOOD | ZNF837 | 19 | 58879606 | 58879606 | C | A | nonsynonymous | NM_138466 | c.G1094T | p.C365F |
| 473 | BLOOD | APOL3 | 22 | 36556846 | 36556846 | C | G | nonsynonymous | NM_145640 | c.G94C | p.G32R |
| 473 | BLOOD | STAG2 | X | 123164815 | 123164815 | G | T | nonsynonymous | NM_001282418 | c.G128T | p.C43F |
| 474 | BLOOD | RBM5 | 3 | 50155888 | 50155889 | GA | - | frameshift | NM_005778 | c.2447_2448del | p.*816fs |
| 474 | BLOOD | MADCAM1 | 19 | 501767 | 501767 | C | T | nonsynonymous | NM_130760 | c.C766T | p.P256S |
| 475 | BLOOD | AQP7 | 9 | 33386465 | 33386465 | A | G | nonsynonymous | NM_001170 | c.T343C | p.Y115H |
| 476 | BLOOD | POU4F2 | 4 | 147560457 | 147560457 | - | GGC | inframe | NM_004575 | c.165_166insGGC | p.G55delinsGG |
| 476 | BLOOD | APOBR | 16 | 28507398 | 28507424 | GGGACAGCCTCAGGAGGGGAGGAGGCC | - | inframe | NM_018690 | c.1036_1062del | p.346_354del |
| 476 | BLOOD | DGKZ | 11 | 46397012 | 46397012 | C | T | nonsynonymous | NM_001105540 | c.C2305T | p.R769W |
| 476 | BLOOD | HEATR5A | 14 | 31849724 | 31849724 | C | A | nonsynonymous | NM_015473 | c.G1657T | p.A553S |
| 476 | BLOOD | CECR2 | 22 | 17982127 | 17982127 | G | A | nonsynonymous | NM_001290046 | c.G175A | p.E59K |
| 477 | BLOOD | KCNN3 | 1 | 154842244 | 154842244 | A | T | nonsynonymous | NM_002249 | c.T197A | p.L66H |
| 477 | BLOOD | SIRT6 | 19 | 4175707 | 4175707 | C | A | nonsynonymous | NM_016539 | c.G584T | p.R195L |
| 477 | BLOOD | RANBP3 | 19 | 5951436 | 5951436 | G | T | nonsynonymous | NM_007322 | c.C250A | p.Q84K |
| 478 | BLOOD | HDGFRP2 | 19 | 4499633 | 4499647 | AGCTGGCCGGGGAGG | - | frameshift | NM_001001520 | c.1721_1722del | p.K574fs |
| 479 | BLOOD | FAM90A1 | 12 | 8374781 | 8374781 | - | ACG | inframe | NM_018088 | c.1031_1032insCGT | p.T344delinsTV |
| 479 | BLOOD | HLA-DQB2 | 6 | 32725559 | 32725559 | C | T | nonsynonymous | NM_001300790 | c.G748A | p.G250S |
| 479 | BLOOD | UPK3B | 7 | 76140336 | 76140336 | G | C | nonsynonymous | NM_030570 | c.G367C | p.D123H,UPK3B |
| 479 | BLOOD | POLR3B | 12 | 106820975 | 106820975 | C | T | nonsynonymous | NM_001160708 | c.C928T | p.L310F,POLR3B |
| 480 | BLOOD | KCNN3 | 1 | 154842244 | 154842244 | A | T | nonsynonymous | NM_002249 | c.T197A | p.L66H |
| 480 | BLOOD | SLC22A20 | 11 | 64981648 | 64981648 | C | A | nonsynonymous | NM_001004326 | c.C305A | p.P102Q |
| 481 | BLOOD | TDG | 12 | 104373728 | 104373728 | - | A | frameshift | NM_003211 | c.287dupA | p.E96fs |
| 481 | BLOOD | MUC20 | 3 | 195447886 | 195447886 | G | C | nonsynonymous | NM_001282506 | c.G8C | p.C3S |
| 481 | BLOOD | ADGRV1 | 5 | 90002172 | 90002172 | A | C | nonsynonymous | NM_032119 | c.A8691C | p.E2897D |
| 481 | BLOOD | DOT1L | 19 | 2226655 | 2226655 | C | A | nonsynonymous | NM_032482 | c.C4135A | p.L1379M |
| 482 | BLOOD | GUCY1A2 | 11 | 106617277 | 106617277 | C | A | nonsynonymous | NM_001256424 | c.G1929T | p.K643N |
| 482 | BLOOD | GFAP | 17 | 42992542 | 42992542 | G | A | nonsynonymous | NM_002055 | c.C313T | p.R105W |
| 483 | BLOOD | RFWD2 | 1 | 176175819 | 176175819 | A | C | nonsynonymous | NM_022457 | c.T296G | p.V99G |
| 483 | BLOOD | ANKRD36 | 2 | 97869931 | 97869931 | A | T | nonsynonymous | NM_001164315 | c.A2992T | p.T998S |
| 483 | BLOOD | EP400 | 12 | 132445256 | 132445256 | A | C | nonsynonymous | NM_015409 | c.A92C | p.H31P |
| 483 | BLOOD | ARHGEF18 | 19 | 7533891 | 7533891 | G | A | nonsynonymous | NM_001130955 | c.G3097A | p.A1033T |
| 484 | BLOOD | HRCT1 | 9 | 35906584 | 35906586 | CCA | - | inframe | NM_001039792 | c.300_302del | p.100_101del |
| 484 | BLOOD | KRT1 | 12 | 53069223 | 53069243 | ACCTCCGGAGCCGTAGCTGCT | - | inframe | NM_006121 | c.1669_1689del | p.557_563del |
| 484 | BLOOD | SOGA3 | 6 | 127837125 | 127837125 | T | C | nonsynonymous | NM_001012279 | c.A635G | p.E212G |
| 484 | BLOOD | LAPTM4B | 8 | 98788011 | 98788011 | T | C | nonsynonymous | NM_018407 | c.T47C | p.L16P |
| 484 | BLOOD | GSE1 | 16 | 85704703 | 85704703 | C | T | nonsynonymous | NM_014615 | c.C3518T | p.A1173V |
| 484 | BLOOD | SEZ6 | 17 | 27283241 | 27283241 | C | G | nonsynonymous | NM_178860 | c.G2888C | p.R963P |
| 484 | BLOOD | ATP8B3 | 19 | 1796099 | 1796099 | G | A | nonsynonymous | NM_138813 | c.C1919T | p.T640M |
| 484 | BLOOD | ZNF556 | 19 | 2877690 | 2877690 | C | T | nonsynonymous | NM_024967 | c.C734T | p.S245F |
| 484 | BLOOD | FUT6 | 19 | 5831671 | 5831671 | C | T | nonsynonymous | NM_000150 | c.G908A | p.R303Q |
| 484 | BLOOD | FBN3 | 19 | 8203162 | 8203162 | C | T | nonsynonymous | NM_032447 | c.G1064A | p.G355D |
| 484 | BLOOD | ANGPTL4 | 19 | 8436190 | 8436190 | G | A | nonsynonymous | NM_139314 | c.G823A | p.V275M |
| 484 | BLOOD | KIAA1683 | 19 | 18368549 | 18368549 | T | C | nonsynonymous | NM_025249 | c.A2984G | p.H995R |
| 484 | BLOOD | MYH14 | 19 | 50766675 | 50766675 | A | C | nonsynonymous | NM_001145809 | c.A2692C | p.K898Q |
| 485 | BLOOD | IFT122 | 3 | 129214358 | 129214358 | T | C | nonsynonymous | NM_052989 | c.T2116C | p.F706L |
| 485 | BLOOD | SEZ6 | 17 | 27283241 | 27283241 | C | G | nonsynonymous | NM_178860 | c.G2888C | p.R963P |
| 486 | BLOOD | MADCAM1 | 19 | 501701 | 501701 | - | ACACCACCTCCCCGGAGCCTCCCA | inframe | NM_130760 | c.700_701insACACCACCTCCCCGGAGCCTCCCA | p.D234delinsDTTSPEPPN |
| 486 | BLOOD | MUC5B | 11 | 1269856 | 1269856 | C | G | nonsynonymous | NM_002458 | c.C11746G | p.P3916A |
| 486 | BLOOD | MUC5B | 11 | 1269860 | 1269860 | C | G | nonsynonymous | NM_002458 | c.C11750G | p.T3917R |
| 486 | BLOOD | PRMT8 | 12 | 3649787 | 3649787 | T | C | nonsynonymous | NM_019854 | c.T91C | p.S31P |
| 487 | BLOOD | GAB2 | 11 | 77937657 | 77937657 | C | G | nonsynonymous | NM_080491 | c.G1061C | p.R354P |
| 488 | BLOOD | APOBR | 16 | 28507398 | 28507424 | GGGACAGCCTCAGGAGGGGAGGAGGCC | - | inframe | NM_018690 | c.1036_1062del | p.346_354del |
| 489 | BLOOD | EP400 | 12 | 132547093 | 132547093 | - | CAG | inframe | NM_015409 | c.8181_8182insCAG | p.Q2727delinsQQ |
| 489 | BLOOD | PLAC4 | 21 | 42551222 | 42551222 | T | G | nonsynonymous | NM_182832 | c.A334C | p.I112L |
| 490 | BLOOD | SEZ6 | 17 | 27283241 | 27283241 | C | G | nonsynonymous | NM_178860 | c.G2888C | p.R963P |
| 490 | BLOOD | PSG1 | 19 | 43372938 | 43372938 | G | A | nonsynonymous | NM_001184825 | c.C958T | p.R320C |
| 490 | BLOOD | CEP250 | 20 | 34079138 | 34079138 | G | A | nonsynonymous | NM_007186 | c.G2855A | p.R952K |
| 491 | BLOOD | HDGFRP2 | 19 | 4499633 | 4499647 | AGCTGGCCGGGGAGG | - | frameshift | NM_001001520 | c.1721_1722del | p.K574fs |
| 491 | BLOOD | EPHA2 | 1 | 16474932 | 16474932 | A | C | nonsynonymous | NM_004431 | c.T764G | p.V255G |
| 491 | BLOOD | MANEAL | 1 | 38262417 | 38262417 | T | G | nonsynonymous | NM_001031740 | c.T662G | p.V221G |
| 491 | BLOOD | GNPAT | 1 | 231411911 | 231411911 | A | C | nonsynonymous | NM_014236 | c.A1968C | p.E656D |
| 491 | BLOOD | EPAS1 | 2 | 46609200 | 46609200 | C | A | nonsynonymous | NM_001430 | c.C2259A | p.D753E |
| 491 | BLOOD | SNRK | 3 | 43389767 | 43389767 | G | T | nonsynonymous | NM_017719 | c.G2016T | p.L672F |
| 491 | BLOOD | ZNF706 | 8 | 102213962 | 102213962 | C | G | nonsynonymous | NM_016096 | c.G8C | p.R3P |
| 491 | BLOOD | CLIP1 | 12 | 122812697 | 122812697 | C | T | nonsynonymous | NM_002956 | c.G3013A | p.E1005K |
| 491 | BLOOD | MYLK3 | 16 | 46744689 | 46744689 | C | A | nonsynonymous | NM_182493 | c.G2127T | p.L709F |
| 491 | BLOOD | NPHS1 | 19 | 36336437 | 36336437 | T | C | nonsynonymous | NM_004646 | c.A1763G | p.E588G |
| 491 | BLOOD | NCF4 | 22 | 37260160 | 37260160 | A | C | nonsynonymous | NM_000631 | c.A106C | p.T36P |
| 491 | BLOOD | APOBEC3H | 22 | 39498015 | 39498015 | C | G | nonsynonymous | NM_001166003 | c.C511G | p.R171G |
| 492 | BLOOD | CTBS | 1 | 85039999 | 85040007 | GCAGCGCCA | - | inframe | NM_004388 | c.92_100del | p.31_34del |
| 493 | BLOOD | IGSF3 | 1 | 117122285 | 117122285 | - | TCC | inframe | NM_001007237 | c.3062_3063insGGA | p.D1021delinsED |
| 493 | BLOOD | SLC12A4 | 16 | 67981606 | 67981606 | C | T | nonsynonymous | NM_001145961 | c.G1933A | p.A645T |
| 494 | BLOOD | GOLGA8B | 15 | 34820194 | 34820194 | C | T | nonsynonymous | NM_001023567 | c.G1538A | p.R513Q |
| 494 | BLOOD | NPHS1 | 19 | 36336437 | 36336437 | T | C | nonsynonymous | NM_004646 | c.A1763G | p.E588G |
| 494 | BLOOD | RBMX | X | 135956506 | 135956506 | C | G | nonsynonymous | NM_002139 | c.G971C | p.R324P |
| 495 | BLOOD | HSPBP1 | 19 | 55790886 | 55790886 | - | GCCGCCGCC | inframe | NM_001130106 | c.90_91insGGCGGCGGC | p.S31delinsGGGS |
| 495 | BLOOD | MUC3A | 7 | 100550443 | 100550443 | A | G | nonsynonymous | NM_005960 | c.A1024G | p.S342G |
| 496 | BLOOD | IRF5 | 7 | 128587352 | 128587381 | ACTCTGCAGCCGCCCACTCTGCGGCCGCCT | - | inframe | NM_001098630 | c.502_531del | p.168_177del |
| 496 | BLOOD | CASP5 | 11 | 104879687 | 104879687 | T | - | frameshift | NM_004347 | c.28delA | p.R10fs |
| 496 | BLOOD | ARSD | X | 2836211 | 2836211 | A | T | nonsynonymous | NM_001669 | c.T497A | p.L166Q |
| 497 | BLOOD | TSPAN10 | 17 | 79614933 | 79614936 | TAAC | - | splicing |  |  |  |
| 497 | BLOOD | HCN2 | 19 | 615947 | 615955 | CCGCCGCCG | - | inframe | NM_001194 | c.2143_2151del | p.715_717del |
| 497 | BLOOD | CLTCL1 | 22 | 19263266 | 19263266 | C | A | nonsynonymous | NM_007098 | c.G130T | p.V44F |
| 498 | BLOOD | FAM90A1 | 12 | 8374781 | 8374781 | - | ACG | inframe | NM_018088 | c.1031_1032insCGT | p.T344delinsTV |
| 498 | BLOOD | CDC27 | 17 | 45232055 | 45232055 | C | T | nonsynonymous | NM_001256 | c.G940A | p.G314R |
| 498 | BLOOD | CDC27 | 17 | 45232070 | 45232070 | C | T | nonsynonymous | NM_001256 | c.G925A | p.D309N |
| 498 | BLOOD | CDC27 | 17 | 45232075 | 45232075 | A | T | nonsynonymous | NM_001256 | c.T920A | p.V307E |
| 498 | BLOOD | CDC27 | 17 | 45232079 | 45232079 | G | A | nonsynonymous | NM_001256 | c.C916T | p.P306S |
| 499 | BLOOD | FAM163B | 9 | 136444231 | 136444231 | G | C | nonsynonymous | NM_001080515 | c.C414G | p.F138L |
| 4 | BLOOD | RPTN | 1 | 152129066 | 152129101 | TGGTGGGAATCTCTGTCTTGTTTCTCAGACTGACCA | - | inframe | NM_001122965 | c.474_509del | p.158_170del |
| 4 | BLOOD | KRTAP5-1 | 11 | 1606121 | 1606150 | CCACAGCCACCCTTGGATCCCCCACAAGAG | - | inframe | NM_001005922 | c.330_359del | p.110_120del |
| 4 | BLOOD | FAM90A1 | 12 | 8374781 | 8374781 | - | ACG | inframe | NM_018088 | c.1031_1032insCGT | p.T344delinsTV |
| 4 | BLOOD | MUC4 | 3 | 195508108 | 195508108 | G | A | nonsynonymous | NM_018406 | c.C10343T | p.S3448L |
| 4 | BLOOD | ASNA1 | 19 | 12848333 | 12848333 | T | G | nonsynonymous | NM_004317 | c.T14G | p.V5G |
| 500 | BLOOD | C6orf223 | 6 | 43970504 | 43970509 | GCGGCG | - | inframe | NM_153246 | c.370_375del | p.124_125del |
| 500 | BLOOD | KRT2 | 12 | 53045603 | 53045603 | - | AAGCCGCTGCCACCTCCA | inframe | NM_000423 | c.323_324insTGGAGGTGGCAGCGGCTT | p.F108delinsFGGGSGF |
| 500 | BLOOD | CDK11A | 1 | 1635356 | 1635356 | C | T | nonsynonymous | NM_024011 | c.G1818A | p.M606I |
| 500 | BLOOD | CCDC144NL | 17 | 20769896 | 20769896 | G | T | nonsynonymous | NM_001004306 | c.C536A | p.T179N |
| 500 | BLOOD | CCDC144NL | 17 | 20769899 | 20769899 | G | T | stopgain | NM_001004306 | c.C533A | p.S178X |
| 500 | BLOOD | SDK2 | 17 | 71361418 | 71361418 | C | T | nonsynonymous | NM_001144952 | c.G5284A | p.E1762K |
| 501 | BLOOD | SLC35D3 | 6 | 137245052 | 137245052 | G | T | nonsynonymous | NM_001008783 | c.G469T | p.G157W |
| 501 | BLOOD | PITX3 | 10 | 103990377 | 103990377 | C | A | nonsynonymous | NM_005029 | c.G803T | p.R268L |
| 501 | BLOOD | ADAMTS7 | 15 | 79051771 | 79051771 | G | T | nonsynonymous | NM_014272 | c.C5053A | p.R1685S |
| 501 | BLOOD | APOBR | 16 | 28509547 | 28509547 | C | A | nonsynonymous | NM_018690 | c.C3101A | p.P1034H |
| 502 | BLOOD | FAM90A1 | 12 | 8374781 | 8374781 | - | ACG | inframe | NM_018088 | c.1031_1032insCGT | p.T344delinsTV |
| 502 | BLOOD | NCOA3 | 20 | 46279864 | 46279866 | CAA | - | inframe | NM_001174087 | c.3787_3789del | p.1263_1263del |
| 502 | BLOOD | TRIM58 | 1 | 248023967 | 248023967 | G | A | nonsynonymous | NM_015431 | c.G469A | p.A157T |
| 502 | BLOOD | IDUA | 4 | 995586 | 995586 | C | T | nonsynonymous | NM_000203 | c.C709T | p.L237F |
| 502 | BLOOD | IFNGR1 | 6 | 137519557 | 137519557 | T | A | nonsynonymous | NM_000416 | c.A1081T | p.N361Y |
| 502 | BLOOD | RP1L1 | 8 | 10467628 | 10467628 | G | A | nonsynonymous | NM_178857 | c.C3980T | p.T1327I |
| 502 | BLOOD | RP1L1 | 8 | 10467629 | 10467629 | T | C | nonsynonymous | NM_178857 | c.A3979G | p.T1327A |
| 502 | BLOOD | RP1L1 | 8 | 10467637 | 10467637 | T | C | nonsynonymous | NM_178857 | c.A3971G | p.E1324G |
| 502 | BLOOD | JAK2 | 9 | 5073770 | 5073770 | G | T | nonsynonymous | NM_004972 | c.G1849T | p.V617F |
| 502 | BLOOD | MLLT3 | 9 | 20414398 | 20414398 | C | G | nonsynonymous | NM_004529 | c.G446C | p.S149T |
| 502 | BLOOD | GXYLT1 | 12 | 42538411 | 42538411 | G | A | nonsynonymous | NM_173601 | c.C38T | p.A13V |
| 502 | BLOOD | FAM186B | 12 | 49994404 | 49994404 | C | G | nonsynonymous | NM_032130 | c.G1019C | p.G340A |
| 502 | BLOOD | PARP4 | 13 | 25021323 | 25021323 | A | G | nonsynonymous | NM_006437 | c.T3116C | p.I1039T |
| 502 | BLOOD | SLC52A3 | 20 | 742437 | 742437 | G | A | nonsynonymous | NM_033409 | c.C1105T | p.L369F |
| 503 | BLOOD | ZNF705G | 8 | 7218632 | 7218632 | C | T | nonsynonymous | NM_001164457 | c.G139A | p.G47R |
| 504 | BLOOD | TAF1B | 2 | 9989570 | 9989570 | - | A | frameshift | NM_005680 | c.187dupA | p.L62fs |
| 504 | BLOOD | FAM90A1 | 12 | 8374781 | 8374781 | - | ACG | inframe | NM_018088 | c.1031_1032insCGT | p.T344delinsTV |
| 504 | BLOOD | TPSD1 | 16 | 1306817 | 1306817 | G | A | nonsynonymous | NM_012217 | c.G274A | p.A92T |
| 504 | BLOOD | CHD3 | 17 | 7796794 | 7796794 | A | C | nonsynonymous | NM_001005273 | c.A700C | p.I234L |
| 505 | BLOOD | MAGI1 | 3 | 65425560 | 65425560 | - | CTG | inframe | NM_001033057 | c.1263_1264insCAG | p.T422delinsQT |
| 505 | BLOOD | COL7A1 | 3 | 48612903 | 48612903 | G | A | nonsynonymous | NM_000094 | c.C6049T | p.P2017S |
| 506 | BLOOD | PLCH2 | 1 | 2411245 | 2411245 | G | A | nonsynonymous | NM_001303013 | c.G404A | p.S135N |
| 506 | BLOOD | DND1 | 5 | 140052430 | 140052430 | G | T | nonsynonymous | NM_194249 | c.C204A | p.D68E |
| 506 | BLOOD | CNTNAP3B | 9 | 43915893 | 43915893 | G | C | nonsynonymous | NM_001201380 | c.G3741C | p.M1247I |
| 506 | BLOOD | MUC5B | 11 | 1258240 | 1258240 | C | T | nonsynonymous | NM_002458 | c.C3143T | p.A1048V |
| 506 | BLOOD | DDX54 | 12 | 113623186 | 113623186 | T | G | nonsynonymous | NM_024072 | c.A71C | p.K24T |
| 506 | BLOOD | NKX2-8 | 14 | 37050642 | 37050642 | C | G | nonsynonymous | NM_014360 | c.G185C | p.S62T |
| 507 | BLOOD | GRM7 | 3 | 7621013 | 7621013 | T | - | frameshift | NM_000844 | c.2420delT | p.I807fs |
| 507 | BLOOD | FAM90A1 | 12 | 8374781 | 8374781 | - | ACG | inframe | NM_018088 | c.1031_1032insCGT | p.T344delinsTV |
| 507 | BLOOD | HHIP | 4 | 145635453 | 145635453 | C | A | stopgain | NM_022475 | c.C1500A | p.C500X |
| 507 | BLOOD | OR4A16 | 11 | 55111496 | 55111496 | T | A | nonsynonymous | NM_001005274 | c.T820A | p.S274T |
| 508 | BLOOD | FOXE1 | 9 | 100616701 | 100616706 | GCCGCC | - | inframe | NM_004473 | c.505_510del | p.169_170del |
| 508 | BLOOD | OR1D5 | 17 | 2966273 | 2966273 | G | A | nonsynonymous | NM_014566 | c.C629T | p.P210L |
| 508 | BLOOD | MADCAM1 | 19 | 498669 | 498669 | G | A | nonsynonymous | NM_130760 | c.G511A | p.E171K |
| 508 | BLOOD | H2BFM | X | 103294760 | 103294760 | C | T | stopgain | NM_001164416 | c.C217T | p.Q73X |
| 509 | BLOOD | HLA-A | 6 | 29910699 | 29910699 | G | A | nonsynonymous | NM_002116 | c.G239A | p.G80E |
| 509 | BLOOD | ASB16 | 17 | 42255657 | 42255657 | G | A | nonsynonymous | NM_080863 | c.G1261A | p.A421T |
| 509 | BLOOD | PLAC4 | 21 | 42551245 | 42551245 | A | G | nonsynonymous | NM_182832 | c.T311C | p.L104P |
| 510 | BLOOD | SEZ6 | 17 | 27283241 | 27283241 | C | G | nonsynonymous | NM_178860 | c.G2888C | p.R963P |
| 511 | BLOOD | SEZ6 | 17 | 27283247 | 27283247 | C | G | nonsynonymous | NM_178860 | c.G2882C | p.R961P |
| 511 | BLOOD | MYOM1 | 18 | 3135552 | 3135552 | G | T | nonsynonymous | NM_003803 | c.C2202A | p.D734E |
| 511 | BLOOD | SSBP4 | 19 | 18541720 | 18541720 | A | G | nonsynonymous | NM_032627 | c.A349G | p.M117V |
| 512 | BLOOD | H1FOO | 3 | 129268109 | 129268109 | G | A | nonsynonymous | NM_153833 | c.G644A | p.R215K |
| 512 | BLOOD | UPF3A | 13 | 115047496 | 115047496 | G | C | nonsynonymous | NM_023011 | c.G208C | p.V70L,UPF3A |
| 512 | BLOOD | MED25 | 19 | 50338843 | 50338843 | C | G | nonsynonymous | NM_030973 | c.C1727G | p.A576G |
| 513 | BLOOD | MIR205HG | 1 | 209605637 | 209605648 | AGCAGCAGCAGC | - | inframe | NM_001104548 | c.252_263del | p.84_88del |
| 513 | BLOOD | HCN2 | 19 | 615947 | 615955 | CCGCCGCCG | - | inframe | NM_001194 | c.2143_2151del | p.715_717del |
| 513 | BLOOD | PRKX | X | 3631167 | 3631167 | A | G | nonsynonymous | NM_005044 | c.T128C | p.V43A |
| 513 | BLOOD | ZXDB | X | 57618870 | 57618870 | G | A | nonsynonymous | NM_007157 | c.G389A | p.G130D |
| 514 | BLOOD | HSPG2 | 1 | 22186113 | 22186113 | T | G | nonsynonymous | NM_001291860 | c.A5242C | p.T1748P |
| 515 | BLOOD | FOXE1 | 9 | 100616701 | 100616706 | GCCGCC | - | inframe | NM_004473 | c.505_510del | p.169_170del |
| 515 | BLOOD | PDE1A | 2 | 183053736 | 183053736 | T | C | nonsynonymous | NM_001003683 | c.A1225G | p.K409E |
| 516 | BLOOD | EP400 | 12 | 132445256 | 132445256 | A | C | nonsynonymous | NM_015409 | c.A92C | p.H31P |
| 516 | BLOOD | LENG9 | 19 | 54974460 | 54974460 | C | A | nonsynonymous | NM_001301782 | c.G250T | p.D84Y |
| 517 | BLOOD | FAM90A1 | 12 | 8374781 | 8374781 | - | ACG | inframe | NM_018088 | c.1031_1032insCGT | p.T344delinsTV |
| 517 | BLOOD | MUC4 | 3 | 195505859 | 195505859 | T | C | nonsynonymous | NM_018406 | c.A12592G | p.T4198A |
| 517 | BLOOD | CCDC144NL | 17 | 20769896 | 20769896 | G | T | nonsynonymous | NM_001004306 | c.C536A | p.T179N |
| 517 | BLOOD | CCDC144NL | 17 | 20769899 | 20769899 | G | T | stopgain | NM_001004306 | c.C533A | p.S178X |
| 517 | BLOOD | ARSD | X | 2836181 | 2836181 | A | T | nonsynonymous | NM_001669 | c.T527A | p.M176K |
| 517 | BLOOD | ARSD | X | 2836184 | 2836184 | C | T | nonsynonymous | NM_001669 | c.G524A | p.G175D |
| 517 | BLOOD | ARSD | X | 2836211 | 2836211 | A | T | nonsynonymous | NM_001669 | c.T497A | p.L166Q |
| 517 | BLOOD | ARSD | X | 2836238 | 2836238 | G | A | nonsynonymous | NM_001669 | c.C470T | p.S157F |
| 518 | BLOOD | FAM86B2 | 8 | 12287957 | 12287957 | C | T | nonsynonymous | NM_001137610 | c.G244A | p.E82K |
| 518 | BLOOD | MBD3L5 | 19 | 7032688 | 7032688 | T | G | nonsynonymous | NM_001136507 | c.T410G | p.I137S |
| 518 | BLOOD | ZNF814 | 19 | 58385748 | 58385748 | G | A | nonsynonymous | NM_001144989 | c.C1010T | p.A337V |
| 518 | BLOOD | PLAC4 | 21 | 42551270 | 42551270 | A | G | nonsynonymous | NM_182832 | c.T286C | p.Y96H |
| 519 | BLOOD | RBM5 | 3 | 50155888 | 50155889 | GA | - | frameshift | NM_005778 | c.2447_2448del | p.*816fs |
| 519 | BLOOD | FAM90A1 | 12 | 8374781 | 8374781 | - | ACG | inframe | NM_018088 | c.1031_1032insCGT | p.T344delinsTV |
| 519 | BLOOD | KRTAP4-8 | 17 | 39253949 | 39253949 | T | A | nonsynonymous | NM_031960 | c.A388T | p.S130C |
| 519 | BLOOD | KRT35 | 17 | 39635733 | 39635733 | G | A | nonsynonymous | NM_002280 | c.C577T | p.R193W |
| 520 | BLOOD | TPSAB1 | 16 | 1291989 | 1291989 | C | A | nonsynonymous | NM_003294 | c.C661A | p.Q221K |
| 521 | BLOOD | USH2A | 1 | 215960153 | 215960153 | A | C | nonsynonymous | NM_206933 | c.T10246G | p.C3416G |
| 521 | BLOOD | DND1 | 5 | 140052430 | 140052430 | G | T | nonsynonymous | NM_194249 | c.C204A | p.D68E |
| 521 | BLOOD | SLC8A3 | 14 | 70515561 | 70515561 | G | A | nonsynonymous | NM_183002 | c.C2330T | p.S777L |
| 522 | BLOOD | POU4F2 | 4 | 147560457 | 147560457 | - | GGC | inframe | NM_004575 | c.165_166insGGC | p.G55delinsGG |
| 522 | BLOOD | MUC4 | 3 | 195515422 | 195515422 | G | T | nonsynonymous | NM_018406 | c.C3029A | p.P1010H |
| 523 | BLOOD | TCERG1 | 5 | 145838636 | 145838647 | CAGGCCCAGGCC | - | inframe | NM_006706 | c.628_639del | p.210_213del |
| 523 | BLOOD | VPS13D | 1 | 12416087 | 12416087 | C | T | stopgain | NM_015378 | c.C9811T | p.R3271X |
| 523 | BLOOD | VCX2 | X | 8138284 | 8138284 | G | C | nonsynonymous | NM_016378 | c.C209G | p.A70G |
| 524 | BLOOD | CBWD6 | 9 | 69247582 | 69247582 | G | C | splicing |  |  |  |
| 524 | BLOOD | ANKRD30B | 18 | 14779986 | 14779986 | G | A | nonsynonymous | NM_001145029 | c.G1448A | p.R483Q |
| 525 | BLOOD | SAAL1 | 11 | 18127558 | 18127558 | - | CGG | inframe | NM_138421 | c.30_31insCCG | p.G11delinsPG |
| 525 | BLOOD | PITPNM3 | 17 | 6386883 | 6386883 | C | T | nonsynonymous | NM_031220 | c.G541A | p.V181I |
| 525 | BLOOD | MXRA5 | X | 3239545 | 3239545 | C | T | nonsynonymous | NM_015419 | c.G4181A | p.G1394D |
| 525 | BLOOD | CHM | X | 85302508 | 85302508 | T | C | nonsynonymous | NM_000390 | c.A29G | p.D10G |
| 526 | BLOOD | EFCAB12 | 3 | 129127679 | 129127680 | TA | - | frameshift | NM_207307 | c.1057_1058del | p.Y353fs |
| 526 | BLOOD | ANKRD36 | 2 | 97877478 | 97877478 | G | A | nonsynonymous | NM_001164315 | c.G3469A | p.V1157M |
| 526 | BLOOD | CRIPAK | 4 | 1388944 | 1388944 | G | A | nonsynonymous | NM_175918 | c.G645A | p.M215I |
| 526 | BLOOD | KRT10 | 17 | 38975149 | 38975149 | G | T | nonsynonymous | NM_000421 | c.C1638A | p.S546R |
| 526 | BLOOD | KRT10 | 17 | 38975151 | 38975151 | T | C | nonsynonymous | NM_000421 | c.A1636G | p.S546G |
| 527 | BLOOD | APOBR | 16 | 28507398 | 28507424 | GGGACAGCCTCAGGAGGGGAGGAGGCC | - | inframe | NM_018690 | c.1036_1062del | p.346_354del |
| 527 | BLOOD | PABPC1 | 8 | 101719201 | 101719201 | A | G | nonsynonymous | NM_002568 | c.T1361C | p.I454T |
| 528 | BLOOD | CTBS | 1 | 85039999 | 85040007 | GCAGCGCCA | - | inframe | NM_004388 | c.92_100del | p.31_34del |
| 529 | BLOOD | BCL11B | 14 | 99641455 | 99641455 | A | C | nonsynonymous | NM_001282237 | c.T1715G | p.V572G |
| 529 | BLOOD | SEZ6 | 17 | 27283241 | 27283241 | C | G | nonsynonymous | NM_178860 | c.G2888C | p.R963P |
| 529 | BLOOD | OGFR | 20 | 61444637 | 61444637 | G | C | nonsynonymous | NM_007346 | c.G1670C | p.S557T |
| 529 | BLOOD | ZXDB | X | 57619097 | 57619097 | G | A | nonsynonymous | NM_007157 | c.G616A | p.G206R |
| 530 | BLOOD | CACTIN | 19 | 3613226 | 3613226 | - | CCTCGC | inframe | NM_001080543 | c.1615_1616insGCGAGG | p.A539delinsGEA |
| 530 | BLOOD | OR7G3 | 19 | 9236698 | 9236698 | - | ATGGT | frameshift | NM_001001958 | c.928_929insACCAT | p.S310fs |
| 530 | BLOOD | HSPBP1 | 19 | 55790886 | 55790886 | - | GCCGCCGCC | inframe | NM_001130106 | c.90_91insGGCGGCGGC | p.S31delinsGGGS |
| 530 | BLOOD | IRF6 | 1 | 209961970 | 209961970 | C | G | nonsynonymous | NM_006147 | c.G1199C | p.R400P |
| 530 | BLOOD | H1FOO | 3 | 129268109 | 129268109 | G | A | nonsynonymous | NM_153833 | c.G644A | p.R215K |
| 530 | BLOOD | FNDC1 | 6 | 159653261 | 159653261 | G | A | nonsynonymous | NM_032532 | c.G1717A | p.G573S |
| 530 | BLOOD | CDC27 | 17 | 45249335 | 45249335 | T | G | nonsynonymous | NM_001256 | c.A199C | p.T67P |
| 530 | BLOOD | PGPEP1 | 19 | 18474346 | 18474346 | C | T | nonsynonymous | NM_017712 | c.C583T | p.L195F |
| 531 | BLOOD | EXO1 | 1 | 242020734 | 242020734 | C | A | nonsynonymous | NM_130398 | c.C493A | p.Q165K |
| 531 | BLOOD | ANXA8L1 | 10 | 47756662 | 47756662 | T | G | nonsynonymous | NM_001098845 | c.T576G | p.N192K |
| 531 | BLOOD | BBOX1 | 11 | 27076985 | 27076985 | G | T | nonsynonymous | NM_003986 | c.G8T | p.C3F |
| 531 | BLOOD | NPHS1 | 19 | 36336437 | 36336437 | T | C | nonsynonymous | NM_004646 | c.A1763G | p.E588G |
| 531 | BLOOD | EIF2S3 | X | 24073777 | 24073777 | C | A | nonsynonymous | NM_001415 | c.C115A | p.Q39K |
| 532 | BLOOD | EXO1 | 1 | 242020734 | 242020734 | C | A | nonsynonymous | NM_130398 | c.C493A | p.Q165K |
| 532 | BLOOD | TYMP | 22 | 50964290 | 50964290 | C | T | nonsynonymous | NM_001953 | c.G1358A | p.R453H |
| 533 | BLOOD | MUC4 | 3 | 195505836 | 195505836 | G | C | nonsynonymous | NM_018406 | c.C12615G | p.H4205Q |
| 533 | BLOOD | IER2 | 19 | 13264338 | 13264338 | C | T | nonsynonymous | NM_004907 | c.C338T | p.P113L |
| 534 | BLOOD | PDK4 | 7 | 95216415 | 95216415 | C | A | nonsynonymous | NM_002612 | c.G1002T | p.L334F |
| 534 | BLOOD | ZXDB | X | 57618870 | 57618870 | G | A | nonsynonymous | NM_007157 | c.G389A | p.G130D |
| 536 | BLOOD | PDE4DIP | 1 | 144923729 | 144923729 | T | - | frameshift | NM_014644 | c.729delA | p.E243fs |
| 536 | BLOOD | POU4F2 | 4 | 147560457 | 147560457 | - | GGC | inframe | NM_004575 | c.165_166insGGC | p.G55delinsGG |
| 536 | BLOOD | EP400 | 12 | 132547093 | 132547093 | - | CAG | inframe | NM_015409 | c.8181_8182insCAG | p.Q2727delinsQQ |
| 536 | BLOOD | KRTAP9-1 | 17 | 39346595 | 39346595 | - | GCTGTGGGTCCAGCTGCTGCCAGCCTA | inframe | NM_001190460 | c.457_458insGCTGTGGGTCCAGCTGCTGCCAGCCTA | p.C153delinsCCGSSCCQPS |
| 536 | BLOOD | OR7G3 | 19 | 9236698 | 9236698 | - | ATGGT | frameshift | NM_001001958 | c.928_929insACCAT | p.S310fs |
| 536 | BLOOD | HSPG2 | 1 | 22155455 | 22155455 | C | A | nonsynonymous | NM_001291860 | c.G12113T | p.R4038L |
| 536 | BLOOD | CNTN3 | 3 | 74350932 | 74350932 | A | G | nonsynonymous | NM_020872 | c.T1811C | p.V604A |
| 536 | BLOOD | IK | 5 | 140038340 | 140038340 | A | G | nonsynonymous | NM_006083 | c.A929G | p.K310R |
| 536 | BLOOD | MYH4 | 17 | 10358358 | 10358358 | C | T | nonsynonymous | NM_017533 | c.G2335A | p.E779K |
| 536 | BLOOD | SIGLEC7 | 19 | 51650516 | 51650516 | C | T | nonsynonymous | NM_014385 | c.C1163T | p.A388V |
| 537 | BLOOD | BPI | 20 | 36936014 | 36936014 | - | CAA | inframe | NM_001725 | c.188_189insCAA | p.I63delinsIN |
| 537 | BLOOD | ANKRD36 | 2 | 97877440 | 97877440 | T | C | nonsynonymous | NM_001164315 | c.T3431C | p.M1144T |
| 537 | BLOOD | ANAPC1 | 2 | 112614429 | 112614429 | G | A | stopgain | NM_022662 | c.C1393T | p.Q465X |
| 537 | BLOOD | FAM83H | 8 | 144809550 | 144809550 | T | A | nonsynonymous | NM_198488 | c.A2081T | p.E694V |
| 537 | BLOOD | EPPK1 | 8 | 144943109 | 144943109 | G | C | nonsynonymous | NM_031308 | c.C4313G | p.T1438R |
| 537 | BLOOD | BCL2L10 | 15 | 52404510 | 52404510 | G | C | nonsynonymous | NM_020396 | c.C414G | p.C138W |
| 537 | BLOOD | EME2 | 16 | 1823458 | 1823458 | C | T | nonsynonymous | NM_001257370 | c.C230T | p.A77V |
| 537 | BLOOD | SUPT20HL2 | X | 24329890 | 24329890 | G | C | nonsynonymous | NM_001136233 | c.C1543G | p.P515A |
| 538 | BLOOD | POU4F2 | 4 | 147560458 | 147560466 | GGCGGCGGC | - | inframe | NM_004575 | c.166_174del | p.56_58del |
| 538 | BLOOD | ATAD3B | 1 | 1431060 | 1431060 | T | C | nonsynonymous | NM_031921 | c.T1810C | p.Y604H |
| 538 | BLOOD | PRR35 | 16 | 614069 | 614069 | A | C | nonsynonymous | NM_145270 | c.A775C | p.T259P |
| 538 | BLOOD | CHD3 | 17 | 7796803 | 7796803 | T | C | nonsynonymous | NM_001005273 | c.T709C | p.S237P |
| 538 | BLOOD | MADCAM1 | 19 | 501786 | 501786 | C | A | nonsynonymous | NM_130760 | c.C785A | p.P262Q |
| 538 | BLOOD | TTLL1 | 22 | 43471583 | 43471583 | T | C | nonsynonymous | NM_012263 | c.A10G | p.K4E |
| 538 | BLOOD | BEX5 | X | 101409117 | 101409117 | G | A | nonsynonymous | NM_001159560 | c.C121T | p.P41S |
| 539 | BLOOD | CBX4 | 17 | 77808240 | 77808240 | - | GTG | inframe | NM_003655 | c.1200_1201insCAC | p.A401delinsHA |
| 539 | BLOOD | PDE4DIP | 1 | 144852379 | 144852379 | G | T | nonsynonymous | NM_001198834 | c.C7064A | p.P2355H |
| 539 | BLOOD | MUC4 | 3 | 195505859 | 195505859 | T | C | nonsynonymous | NM_018406 | c.A12592G | p.T4198A |
| 539 | BLOOD | SIRPA | 20 | 1895963 | 1895963 | A | G | nonsynonymous | NM_001040023 | c.A298G | p.N100D |
| 539 | BLOOD | SIRPA | 20 | 1895965 | 1895965 | C | A | nonsynonymous | NM_001040023 | c.C300A | p.N100K |
| 539 | BLOOD | DMD | X | 31986607 | 31986607 | G | A | nonsynonymous | NM_000109 | c.C6439T | p.R2147W |
| 539 | BLOOD | ZXDB | X | 57618870 | 57618870 | G | A | nonsynonymous | NM_007157 | c.G389A | p.G130D |
| 539 | BLOOD | SLC16A2 | X | 73641569 | 73641569 | T | C | nonsynonymous | NM_006517 | c.T97C | p.S33P |
| 542 | BLOOD | IGSF3 | 1 | 117122285 | 117122285 | - | TCC | inframe | NM_001007237 | c.3062_3063insGGA | p.D1021delinsED |
| 542 | BLOOD | AQP7 | 9 | 33386510 | 33386510 | C | T | nonsynonymous | NM_001170 | c.G298A | p.A100T |
| 543 | BLOOD | ZNF706 | 8 | 102213962 | 102213962 | C | G | nonsynonymous | NM_016096 | c.G8C | p.R3P |
| 543 | BLOOD | MADCAM1 | 19 | 501786 | 501786 | C | A | nonsynonymous | NM_130760 | c.C785A | p.P262Q |
| 544 | BLOOD | POU4F2 | 4 | 147560457 | 147560457 | - | GGC | inframe | NM_004575 | c.165_166insGGC | p.G55delinsGG |
| 544 | BLOOD | MUC4 | 3 | 195511102 | 195511102 | G | A | nonsynonymous | NM_018406 | c.C7349T | p.P2450L |
| 544 | BLOOD | GAB2 | 11 | 77937657 | 77937657 | C | G | nonsynonymous | NM_080491 | c.G1061C | p.R354P |
| 544 | BLOOD | HOXC5 | 12 | 54427115 | 54427115 | A | C | nonsynonymous | NM_018953 | c.A209C | p.H70P |
| 544 | BLOOD | TTLL1 | 22 | 43471583 | 43471583 | T | C | nonsynonymous | NM_012263 | c.A10G | p.K4E |
| 545 | BLOOD | PCDHB10 | 5 | 140574170 | 140574175 | AGGCCG | - | inframe | NM_018930 | c.2045_2050del | p.682_684del |
| 545 | BLOOD | ATP6V1A | 3 | 113524266 | 113524266 | G | C | nonsynonymous | NM_001690 | c.G1655C | p.R552P |
| 545 | BLOOD | RP1L1 | 8 | 10467625 | 10467625 | T | A | nonsynonymous | NM_178857 | c.A3983T | p.E1328V |
| 545 | BLOOD | RP1L1 | 8 | 10467626 | 10467626 | C | T | nonsynonymous | NM_178857 | c.G3982A | p.E1328K |
| 545 | BLOOD | RP1L1 | 8 | 10467628 | 10467628 | G | A | nonsynonymous | NM_178857 | c.C3980T | p.T1327I |
| 545 | BLOOD | MADCAM1 | 19 | 501762 | 501762 | A | C | nonsynonymous | NM_130760 | c.A761C | p.Q254P |
| 545 | BLOOD | RASGRP4 | 19 | 38905715 | 38905715 | G | C | nonsynonymous | NM_170604 | c.C1003G | p.R335G |
| 545 | BLOOD | RIN2 | 20 | 19941419 | 19941419 | C | T | nonsynonymous | NM_001242581 | c.C574T | p.P192S |
| 549 | LUNG | SPATA21 | 1 | 16731492 | 16731492 | C | G | nonsynonymous | NM_198546 | c.G781C | p.E261Q |
| 550 | LUNG | FAM71E1 | 19 | 50971007 | 50971007 | G | A | nonsynonymous | NM_138411 | c.C571T | p.R191C |
| 550 | LUNG | ARSD | X | 2836047 | 2836047 | C | T | nonsynonymous | NM_001669 | c.G661A | p.G221S |
| 551 | LUNG | ZNF718 | 4 | 154894 | 154894 | A | G | nonsynonymous | NM_001039127 | c.A419G | p.K140R,ZNF718 |
| 551 | LUNG | ZNF718 | 4 | 154895 | 154895 | A | C | nonsynonymous | NM_001039127 | c.A420C | p.K140N,ZNF718 |
| 551 | LUNG | CYP21A2 | 6 | 32008862 | 32008862 | G | T | nonsynonymous | NM_000500 | c.G1439T | p.R480L |
| 551 | LUNG | AK8 | 9 | 135668127 | 135668127 | G | A | nonsynonymous | NM_152572 | c.C1015T | p.R339C |
| 551 | LUNG | ANKK1 | 11 | 113270475 | 113270475 | C | T | nonsynonymous | NM_178510 | c.C1784T | p.T595I |
| 551 | LUNG | ADAMTS7 | 15 | 79051846 | 79051846 | T | C | nonsynonymous | NM_014272 | c.A4978G | p.I1660V |
| 551 | LUNG | LAMA5 | 20 | 60897450 | 60897450 | G | A | nonsynonymous | NM_005560 | c.C6221T | p.P2074L |
| 551 | LUNG | P2RY8 | X | 1585079 | 1585079 | C | T | nonsynonymous | NM_178129 | c.G373A | p.V125I |
| 552 | LUNG | MCC | 5 | 112824048 | 112824048 | - | GCC | inframe | NM_001085377 | c.63_64insGGC | p.S22delinsGS |
| 552 | LUNG | FGR | 1 | 27942346 | 27942346 | T | A | nonsynonymous | NM_005248 | c.A692T | p.D231V |
| 552 | LUNG | MADCAM1 | 19 | 501786 | 501786 | C | A | nonsynonymous | NM_130760 | c.C785A | p.P262Q |
| 552 | LUNG | ZXDB | X | 57618845 | 57618845 | G | A | nonsynonymous | NM_007157 | c.G364A | p.E122K |
| 552 | LUNG | ZXDB | X | 57618849 | 57618849 | A | C | nonsynonymous | NM_007157 | c.A368C | p.E123A |
| 552 | LUNG | ZXDB | X | 57618870 | 57618870 | G | A | nonsynonymous | NM_007157 | c.G389A | p.G130D |
| 553 | LUNG | IGSF3 | 1 | 117122285 | 117122285 | - | TCC | inframe | NM_001007237 | c.3062_3063insGGA | p.D1021delinsED |
| 553 | LUNG | FANK1 | 10 | 127585212 | 127585212 | A | T | nonsynonymous | NM_145235 | c.A1T | p.M1L |
| 554 | LUNG | DBI | 2 | 120125198 | 120125198 | C | - | frameshift | NM_001178042 | c.38delC | p.A13fs |
| 554 | LUNG | ALG1L2 | 3 | 129814935 | 129814935 | C | - | frameshift | NM_001136152 | c.468delC | p.G156fs |
| 554 | LUNG | ATXN1 | 6 | 16327913 | 16327915 | TGA | - | inframe | NM_000332 | c.627_629del | p.209_210del |
| 554 | LUNG | RSPH9 | 6 | 43638655 | 43638657 | AGA | - | inframe | NM_152732 | c.800_802del | p.267_268del |
| 554 | LUNG | COG3 | 13 | 46039203 | 46039203 | - | GGC | inframe | NM_031431 | c.32_33insGGC | p.E11delinsEA |
| 554 | LUNG | COL18A1 | 21 | 46923943 | 46923945 | TTC | - | inframe | NM_130444 | c.3947_3949del | p.1316_1317del |
| 554 | LUNG | PLCH2 | 1 | 2435759 | 2435759 | G | A | nonsynonymous | NM_014638 | c.G3358A | p.V1120M |
| 554 | LUNG | PADI1 | 1 | 17550167 | 17550167 | G | A | nonsynonymous | NM_013358 | c.G325A | p.V109M |
| 554 | LUNG | LRRIQ3 | 1 | 74648385 | 74648385 | T | A | nonsynonymous | NM_001105659 | c.A410T | p.K137I |
| 554 | LUNG | FAM69A | 1 | 93341897 | 93341897 | T | C | nonsynonymous | NM_001006605 | c.A145G | p.T49A |
| 554 | LUNG | PDE4DIP | 1 | 144852477 | 144852477 | G | A | nonsynonymous | NM_014644 | c.C7022T | p.P2341L |
| 554 | LUNG | KCNN3 | 1 | 154842244 | 154842244 | A | T | nonsynonymous | NM_002249 | c.T197A | p.L66H |
| 554 | LUNG | ANKRD36B | 2 | 98201508 | 98201508 | C | A | nonsynonymous | NM_025190 | c.G349T | p.V117F |
| 554 | LUNG | POTEE | 2 | 132021702 | 132021702 | A | G | nonsynonymous | NM_001083538 | c.A2674G | p.I892V |
| 554 | LUNG | KCNH7 | 2 | 163250969 | 163250969 | C | T | nonsynonymous | NM_033272 | c.G2640A | p.M880I |
| 554 | LUNG | ICA1L | 2 | 203676470 | 203676470 | T | G | nonsynonymous | NM_138468 | c.A909C | p.Q303H |
| 554 | LUNG | CYP20A1 | 2 | 204154575 | 204154575 | T | G | nonsynonymous | NM_177538 | c.T1059G | p.I353M |
| 554 | LUNG | LRRC2 | 3 | 46580676 | 46580676 | A | T | nonsynonymous | NM_024512 | c.T349A | p.L117M |
| 554 | LUNG | HRG | 3 | 186395145 | 186395145 | C | A | nonsynonymous | NM_000412 | c.C1051A | p.P351T |
| 554 | LUNG | CCDC158 | 4 | 77290670 | 77290670 | C | T | nonsynonymous | NM_001042784 | c.G1256A | p.R419Q |
| 554 | LUNG | SYNPO2 | 4 | 119948506 | 119948506 | G | A | nonsynonymous | NM_001128933 | c.G982A | p.V328I |
| 554 | LUNG | MFSD8 | 4 | 128878744 | 128878744 | T | A | nonsynonymous | NM_152778 | c.A66T | p.E22D |
| 554 | LUNG | PCDHGB7 | 5 | 140799072 | 140799072 | G | A | nonsynonymous | NM_018927 | c.G1646A | p.R549H |
| 554 | LUNG | SCIN | 7 | 12666377 | 12666377 | A | T | nonsynonymous | NM_001112706 | c.A1150T | p.M384L |
| 554 | LUNG | PSPH | 7 | 56079483 | 56079483 | A | G | nonsynonymous | NM_004577 | c.T650C | p.V217A |
| 554 | LUNG | ZSCAN21 | 7 | 99662218 | 99662218 | C | A | nonsynonymous | NM_145914 | c.C1400A | p.T467N |
| 554 | LUNG | C9orf89 | 9 | 95875334 | 95875334 | G | C | nonsynonymous | NM_032310 | c.G497C | p.R166T |
| 554 | LUNG | HBG1 | 11 | 5269623 | 5269623 | G | C | nonsynonymous | NM_000559 | c.C410G | p.A137G |
| 554 | LUNG | FLT3 | 13 | 28611382 | 28611382 | T | G | nonsynonymous | NM_004119 | c.A1249C | p.I417L |
| 554 | LUNG | SERPINE3 | 13 | 51935474 | 51935474 | A | G | nonsynonymous | NM_001101320 | c.A1061G | p.E354G |
| 554 | LUNG | CDC27 | 17 | 45214528 | 45214528 | A | T | nonsynonymous | NM_001256 | c.T1903A | p.Y635N |
| 554 | LUNG | BCAM | 19 | 45317469 | 45317469 | G | A | nonsynonymous | NM_005581 | c.G845A | p.R282H |
| 554 | LUNG | ZNF665 | 19 | 53668419 | 53668419 | G | A | stopgain | NM_024733 | c.C1324T | p.R442X |
| 554 | LUNG | PEG3 | 19 | 57327420 | 57327420 | C | A | nonsynonymous | NM_001146184 | c.G2390T | p.S797I |
| 556 | LUNG | WDR66 | 12 | 122359397 | 122359397 | - | GAGGAGGAGGAGAAA | inframe | NM_144668 | c.186_187insGAGGAGGAGGAGAAA | p.G62delinsGEEEEK |
| 557 | LUNG | PCDHGA10 | 5 | 140795208 | 140795208 | - | A | frameshift | NM_032090 | c.2467dupA | p.V822fs |
| 557 | LUNG | C1orf174 | 1 | 3807360 | 3807360 | G | A | nonsynonymous | NM_207356 | c.C391T | p.R131C |
| 557 | LUNG | VGLL4 | 3 | 11606422 | 11606422 | A | G | nonsynonymous | NM_001284391 | c.T149C | p.V50A |
| 557 | LUNG | FTH1P18 | X | 37061389 | 37061389 | C | T | nonsynonymous | NM_001271682 | c.G373A | p.G125R |
| 557 | LUNG | FTH1P18 | X | 37061416 | 37061416 | C | T | nonsynonymous | NM_001271682 | c.G346A | p.G116S |
| 557 | LUNG | CXorf36 | X | 45011096 | 45011096 | C | T | nonsynonymous | NM_176819 | c.G1103A | p.R368Q |
| 557 | LUNG | GATA1 | X | 48649679 | 48649679 | G | A | nonsynonymous | NM_002049 | c.G163A | p.A55T |
| 558 | LUNG | TTBK1 | 6 | 43251750 | 43251750 | G | A | nonsynonymous | NM_032538 | c.G3272A | p.R1091Q |
| 559 | BLOOD | RAD18 | 3 | 9005053 | 9005053 | T | G | nonsynonymous | NM_020165 | c.A17C | p.E6A |
| 559 | BLOOD | LRTM1 | 3 | 54952826 | 54952826 | G | C | nonsynonymous | NM_020678 | c.C698G | p.P233R |
| 559 | BLOOD | HEG1 | 3 | 124731553 | 124731553 | G | A | nonsynonymous | NM_020733 | c.C2870T | p.T957M |
| 559 | BLOOD | PRIM2 | 6 | 57398318 | 57398318 | G | A | splicing |  |  |  |
| 559 | BLOOD | EXD3 | 9 | 140201476 | 140201476 | G | A | stopgain | NM_017820 | c.C2557T | p.R853X |
| 559 | BLOOD | HTATIP2 | 11 | 20388820 | 20388820 | C | G | nonsynonymous | NM_001098522 | c.C296G | p.A99G |
| 559 | BLOOD | PNKP | 19 | 50370425 | 50370425 | C | G | nonsynonymous | NM_007254 | c.G37C | p.E13Q |
| 560 | BLOOD | SOWAHB | 4 | 77818089 | 77818089 | - | GCT | inframe | NM_001029870 | c.913_914insAGC | p.R305delinsQR |
| 560 | BLOOD | MTFR1 | 8 | 66619280 | 66619285 | CCACAC | - | inframe | NM_014637 | c.553_558del | p.185_186del |
| 560 | BLOOD | ANKRD36 | 2 | 97877478 | 97877478 | G | A | nonsynonymous | NM_001164315 | c.G3469A | p.V1157M |
| 560 | BLOOD | EDEM1 | 3 | 5255091 | 5255091 | C | G | nonsynonymous | NM_014674 | c.C1768G | p.L590V |
| 560 | BLOOD | PLOD2 | 3 | 145878767 | 145878767 | A | T | nonsynonymous | NM_000935 | c.T10A | p.C4S |
| 560 | BLOOD | TIMD4 | 5 | 156347505 | 156347505 | A | G | nonsynonymous | NM_138379 | c.T1022C | p.M341T |
| 560 | BLOOD | C10orf55 | 10 | 75671481 | 75671481 | G | A | nonsynonymous | NM_001001791 | c.C418T | p.P140S |
| 560 | BLOOD | CEP290 | 12 | 88502847 | 88502847 | G | C | nonsynonymous | NM_025114 | c.C2479G | p.L827V |
| 560 | BLOOD | ACOT2 | 14 | 74036255 | 74036255 | A | G | nonsynonymous | NM_006821 | c.A311G | p.K104R |
| 560 | BLOOD | CDC27 | 17 | 45249335 | 45249335 | T | G | nonsynonymous | NM_001256 | c.A199C | p.T67P |
| 560 | BLOOD | CGB7 | 19 | 49558216 | 49558216 | C | T | nonsynonymous | NM_033142 | c.G65A | p.R22K |
| 561 | BLOOD | ASPN | 9 | 95237025 | 95237027 | TCA | - | inframe | NM_017680 | c.153_155del | p.51_52del |
| 561 | BLOOD | IDO2 | 8 | 39821152 | 39821152 | C | A | nonsynonymous | NM_194294 | c.C164A | p.P55H |
| 561 | BLOOD | SERPINF2 | 17 | 1650418 | 1650418 | G | A | nonsynonymous | NM_000934 | c.G473A | p.G158D |
| 561 | BLOOD | GNAS | 20 | 57429573 | 57429573 | C | A | nonsynonymous | NM_080425 | c.C1253A | p.A418D |
| 561 | BLOOD | GNAS | 20 | 57429574 | 57429574 | C | A | nonsynonymous | NM_001077490 | c.C1067A | p.P356Q |
| 562 | BLOOD | HOXD9 | 2 | 176988291 | 176988293 | GCA | - | inframe | NM_014213 | c.795_797del | p.265_266del |
| 562 | BLOOD | HACL1 | 3 | 15633166 | 15633168 | AAC | - | inframe | NM_012260 | c.247_249del | p.83_83del |
| 562 | BLOOD | AVPR1A | 12 | 63544554 | 63544562 | CAGAGGCCA | - | inframe | NM_000706 | c.55_63del | p.19_21del |
| 562 | BLOOD | RIN3 | 14 | 93154538 | 93154540 | GGC | - | inframe | NM_024832 | c.2899_2901del | p.967_967del |
| 562 | BLOOD | MUC16 | 19 | 9082466 | 9082483 | GTGTGAAGGTTAACGTCT | - | inframe | NM_024690 | c.9332_9349del | p.3111_3117del |
| 562 | BLOOD | TRIP11 | 14 | 92470251 | 92470251 | G | T | nonsynonymous | NM_004239 | c.C4069A | p.Q1357K |
| 562 | BLOOD | KRTAP4-9 | 17 | 39262166 | 39262166 | G | C | nonsynonymous | NM_001146041 | c.G526C | p.V176L |
| 562 | BLOOD | SLC35G4 | 18 | 11609994 | 11609994 | C | T | nonsynonymous | NM_001282300 | c.C400T | p.R134C |
| 562 | BLOOD | TNPO2 | 19 | 12812982 | 12812982 | G | A | nonsynonymous | NM_001136196 | c.C2315T | p.T772M |
| 562 | BLOOD | CACNA1I | 22 | 40056439 | 40056439 | G | A | nonsynonymous | NM_021096 | c.G2695A | p.G899R |
| 562 | BLOOD | ARSD | X | 2836047 | 2836047 | C | T | nonsynonymous | NM_001669 | c.G661A | p.G221S |
| 562 | BLOOD | ARSD | X | 2836181 | 2836181 | A | T | nonsynonymous | NM_001669 | c.T527A | p.M176K |
| 562 | BLOOD | ARSD | X | 2836184 | 2836184 | C | T | nonsynonymous | NM_001669 | c.G524A | p.G175D |
| 563 | BLOOD | SLC22A16 | 6 | 110759940 | 110759940 | C | T | nonsynonymous | NM_033125 | c.G1294A | p.V432I |
| 563 | BLOOD | SOGA3 | 6 | 127837119 | 127837119 | G | C | nonsynonymous | NM_001012279 | c.C641G | p.A214G |
| 563 | BLOOD | GEMIN4 | 17 | 648156 | 648156 | C | T | nonsynonymous | NM_015721 | c.G3127A | p.G1043S |
| 563 | BLOOD | EIF5A | 17 | 7210402 | 7210402 | C | T | nonsynonymous | NM_001143760 | c.C37T | p.R13C |
| 563 | BLOOD | PIK3R5 | 17 | 8790936 | 8790936 | C | T | nonsynonymous | NM_001142633 | c.G1681A | p.V561M |
| 563 | BLOOD | ABHD17A | 19 | 1880904 | 1880904 | A | C | nonsynonymous | NM_031213 | c.T476G | p.L159R |
| 564 | BLOOD | NADK | 1 | 1684347 | 1684347 | - | CCT | inframe | NM_023018 | c.1336_1337insAGG | p.G446delinsEG |
| 564 | BLOOD | BCL6B | 17 | 6928019 | 6928019 | - | CAG | inframe | NM_181844 | c.701_702insCAG | p.S234delinsSS |
| 564 | BLOOD | MYOM1 | 18 | 3135629 | 3135631 | AGT | - | inframe | NM_003803 | c.2123_2125del | p.708_709del |
| 564 | BLOOD | LDLRAD2 | 1 | 22140910 | 22140910 | C | G | nonsynonymous | NM_001013693 | c.C105G | p.C35W |
| 564 | BLOOD | ZFYVE9 | 1 | 52704264 | 52704264 | A | G | nonsynonymous | NM_004799 | c.A1175G | p.H392R |
| 564 | BLOOD | UQCC3 | 11 | 62439530 | 62439530 | T | G | nonsynonymous | NM_001085372 | c.T226G | p.W76G |
| 564 | BLOOD | MYEOV | 11 | 69063726 | 69063726 | G | T | nonsynonymous | NM_138768 | c.G809T | p.G270V |
| 564 | BLOOD | PIK3C2G | 12 | 18793382 | 18793382 | C | T | nonsynonymous | NM_001288772 | c.C4202T | p.A1401V |
| 564 | BLOOD | MYO1A | 12 | 57442059 | 57442059 | C | G | nonsynonymous | NM_001256041 | c.G49C | p.E17Q |
| 564 | BLOOD | CDC27 | 17 | 45258943 | 45258943 | G | A | nonsynonymous | NM_001256 | c.C88T | p.R30C |
| 564 | BLOOD | LILRA4 | 19 | 54848121 | 54848121 | C | T | nonsynonymous | NM_012276 | c.G1246A | p.V416M |
| 564 | BLOOD | PPP6R2 | 22 | 50857832 | 50857832 | G | T | nonsynonymous | NM_001242898 | c.G786T | p.E262D |
| 565 | BLOOD | MCC | 5 | 112824036 | 112824036 | - | GCC | inframe | NM_001085377 | c.75_76insGGC | p.S26delinsGS |
| 565 | BLOOD | TRIM52 | 5 | 180687429 | 180687431 | TCT | - | inframe | NM_032765 | c.384_386del | p.128_129del |
| 565 | BLOOD | HLA-A | 6 | 29912029 | 29912029 | G | - | frameshift | NM_002116 | c.750delG | p.Q250fs |
| 565 | BLOOD | RP1L1 | 8 | 10466873 | 10466873 | - | CTT | inframe | NM_178857 | c.4734_4735insAAG | p.L1579delinsKL |
| 565 | BLOOD | OR4X1 | 11 | 48285982 | 48285984 | CTT | - | inframe | NM_001004726 | c.570_572del | p.190_191del |
| 565 | BLOOD | DUSP16 | 12 | 12630666 | 12630674 | GCACGCTGG | - | inframe | NM_030640 | c.1091_1099del | p.364_367del |
| 565 | BLOOD | VSIG10 | 12 | 118506327 | 118506327 | - | TCCTCC | inframe | NM_019086 | c.1421_1422insGGAGGA | p.E474delinsEEE |
| 565 | BLOOD | ATXN3 | 14 | 92537354 | 92537354 | - | CTG | inframe | NM_001164782 | c.67_68insCAG | p.G23delinsAG,ATXN3 |
| 565 | BLOOD | UTP15 | 5 | 72875159 | 72875159 | G | A | nonsynonymous | NM_032175 | c.G1298A | p.R433K |
| 565 | BLOOD | PCDH12 | 5 | 141336858 | 141336858 | C | T | nonsynonymous | NM_016580 | c.G559A | p.G187S |
| 565 | BLOOD | TIMD4 | 5 | 156347509 | 156347509 | G | C | nonsynonymous | NM_138379 | c.C1018G | p.L340V |
| 565 | BLOOD | FAM193B | 5 | 176951716 | 176951716 | G | T | nonsynonymous | NM_001190946 | c.C1766A | p.T589N |
| 565 | BLOOD | OR5V1 | 6 | 29323837 | 29323837 | T | C | nonsynonymous | NM_030876 | c.A136G | p.I46V |
| 565 | BLOOD | HLA-A | 6 | 29910716 | 29910716 | C | G | nonsynonymous | NM_002116 | c.C256G | p.Q86E |
| 565 | BLOOD | TRIM31 | 6 | 30078235 | 30078235 | T | C | nonsynonymous | NM_007028 | c.A734G | p.Q245R |
| 565 | BLOOD | CFB | 6 | 31916231 | 31916231 | A | C | nonsynonymous | NM_001710 | c.A978C | p.E326D |
| 565 | BLOOD | HLA-DRB5 | 6 | 32487309 | 32487309 | T | C | nonsynonymous | NM_002125 | c.A490G | p.S164G |
| 565 | BLOOD | HLA-DRB5 | 6 | 32497970 | 32497970 | T | C | nonsynonymous | NM_002125 | c.A32G | p.Y11C |
| 565 | BLOOD | HLA-DRB1 | 6 | 32549452 | 32549452 | C | G | nonsynonymous | NM_002124 | c.G534C | p.Q178H |
| 565 | BLOOD | ITPR3 | 6 | 33651918 | 33651918 | G | A | nonsynonymous | NM_002224 | c.G4910A | p.R1637H |
| 565 | BLOOD | REPIN1 | 7 | 150068475 | 150068475 | G | C | nonsynonymous | NM_014374 | c.G145C | p.G49R |
| 565 | BLOOD | IDO2 | 8 | 39871098 | 39871098 | C | T | nonsynonymous | NM_194294 | c.C773T | p.P258L |
| 565 | BLOOD | OR52B2 | 11 | 6191528 | 6191528 | T | C | nonsynonymous | NM_001004052 | c.A29G | p.H10R |
| 565 | BLOOD | MICAL2 | 11 | 12183823 | 12183823 | C | G | nonsynonymous | NM_001282663 | c.C121G | p.L41V |
| 565 | BLOOD | IGSF22 | 11 | 18730954 | 18730954 | A | G | nonsynonymous | NM_173588 | c.T2978C | p.V993A |
| 565 | BLOOD | IGSF22 | 11 | 18731005 | 18731005 | C | T | nonsynonymous | NM_173588 | c.G2927A | p.R976Q |
| 565 | BLOOD | NAT10 | 11 | 34165053 | 34165053 | G | A | nonsynonymous | NM_024662 | c.G2947A | p.A983T |
| 565 | BLOOD | OR4C3 | 11 | 48347274 | 48347274 | C | T | nonsynonymous | NM_001004702 | c.C782T | p.A261V |
| 565 | BLOOD | HEPHL1 | 11 | 93803598 | 93803598 | G | C | nonsynonymous | NM_001098672 | c.G1122C | p.K374N |
| 565 | BLOOD | ALKBH8 | 11 | 107427622 | 107427622 | G | C | nonsynonymous | NM_138775 | c.C237G | p.N79K |
| 565 | BLOOD | FLI1 | 11 | 128628065 | 128628065 | C | T | nonsynonymous | NM_002017 | c.C74T | p.A25V |
| 565 | BLOOD | BRCA2 | 13 | 32914904 | 32914904 | G | T | nonsynonymous | NM_000059 | c.G6412T | p.V2138F |
| 565 | BLOOD | MTHFSD | 16 | 86575777 | 86575777 | T | C | nonsynonymous | NM_001159377 | c.A485G | p.Y162C |
| 565 | BLOOD | MISP | 19 | 763550 | 763550 | G | A | nonsynonymous | NM_173481 | c.G2000A | p.R667H |
| 565 | BLOOD | DOT1L | 19 | 2226415 | 2226415 | T | A | nonsynonymous | NM_032482 | c.T3895A | p.S1299T |
| 565 | BLOOD | DOCK6 | 19 | 11319598 | 11319598 | C | T | nonsynonymous | NM_020812 | c.G4933A | p.V1645I |
| 565 | BLOOD | CYP4F12 | 19 | 15784436 | 15784436 | C | T | nonsynonymous | NM_023944 | c.C97T | p.R33C |
| 565 | BLOOD | JAK3 | 19 | 17953950 | 17953950 | G | C | nonsynonymous | NM_000215 | c.C452G | p.P151R |
| 565 | BLOOD | ZNF233 | 19 | 44778178 | 44778178 | G | T | nonsynonymous | NM_001207005 | c.G1365T | p.E455D |
| 565 | BLOOD | PRODH | 22 | 18905961 | 18905961 | C | T | nonsynonymous | NM_016335 | c.G1295A | p.R432H |
| 565 | BLOOD | CCDC116 | 22 | 21989106 | 21989106 | G | A | nonsynonymous | NM_152612 | c.G754A | p.V252M |
| 565 | BLOOD | SSTR3 | 22 | 37602608 | 37602608 | G | A | nonsynonymous | NM_001051 | c.C1235T | p.T412M |
| 565 | BLOOD | TRIOBP | 22 | 38121094 | 38121094 | T | G | nonsynonymous | NM_001039141 | c.T2531G | p.L844R |
| 565 | BLOOD | MIOX | 22 | 50928010 | 50928010 | G | A | nonsynonymous | NM_017584 | c.G686A | p.R229H |
| 566 | BLOOD | RETSAT | 2 | 85570849 | 85570849 | C | T | nonsynonymous | NM_017750 | c.G1606A | p.G536R |
| 566 | BLOOD | OPLAH | 8 | 145110772 | 145110772 | C | T | nonsynonymous | NM_017570 | c.G2167A | p.A723T |
| 566 | BLOOD | TMEM132A | 11 | 60703492 | 60703492 | A | G | nonsynonymous | NM_178031 | c.A2185G | p.S729G |
| 567 | BLOOD | KAT6B | 10 | 76788660 | 76788668 | GAAGAGGAA | - | inframe | NM_012330 | c.4078_4086del | p.1360_1362del |
| 567 | BLOOD | FRMD1 | 6 | 168462625 | 168462625 | C | T | nonsynonymous | NM_024919 | c.G907A | p.A303T |
| 567 | BLOOD | VCX2 | X | 8138284 | 8138284 | G | C | nonsynonymous | NM_016378 | c.C209G | p.A70G |
| 568 | BLOOD | CDK11B | 1 | 1571841 | 1571841 | A | C | nonsynonymous | NM_001787 | c.T1953G | p.D651E |
| 6 | BLOOD | KMT2C | 7 | 151875096 | 151875096 | - | TT | splicing |  |  |  |
| 6 | BLOOD | FBN2 | 5 | 127863579 | 127863579 | G | A | nonsynonymous | NM_001999 | c.C518T | p.T173I |
| 6 | BLOOD | CPNE3 | 8 | 87563330 | 87563330 | T | G | splicing |  |  |  |
| 6 | BLOOD | ZNF814 | 19 | 58385546 | 58385546 | G | T | nonsynonymous | NM_001144989 | c.C1212A | p.D404E |
| 6 | BLOOD | ARSD | X | 2836047 | 2836047 | C | T | nonsynonymous | NM_001669 | c.G661A | p.G221S |
| 7 | BLOOD | HLA-A | 6 | 29912108 | 29912108 | G | C | nonsynonymous | NM_002116 | c.G829C | p.E277Q |
| 7 | BLOOD | RYR3 | 15 | 33962623 | 33962623 | T | G | nonsynonymous | NM_001036 | c.T5726G | p.V1909G |
| 7 | BLOOD | NARFL | 16 | 781697 | 781697 | C | G | nonsynonymous | NM_022493 | c.G902C | p.S301T |
| 87 | BLOOD | HLA-A | 6 | 29910716 | 29910716 | C | G | nonsynonymous | NM_002116 | c.C256G | p.Q86E |
| 88 | BLOOD | SNAPC4 | 9 | 139277995 | 139277997 | GCT | - | inframe | NM_003086 | c.1624_1626del | p.542_542del |
| 88 | BLOOD | BIK | 22 | 43525235 | 43525264 | TGCTGCTGGCGCTGCTGCTGCTGCTGGCGC | - | inframe | NM_001197 | c.407_436del | p.136_146del |
| 8 | BLOOD | RPTN | 1 | 152129066 | 152129101 | TGGTGGGAATCTCTGTCTTGTTTCTCAGACTGACCA | - | inframe | NM_001122965 | c.474_509del | p.158_170del |
| 8 | BLOOD | CCDC180 | 9 | 100092968 | 100092968 | - | GAGGAG | inframe | NM_020893 | c.2325_2326insGAGGAG | p.E775delinsEEE |
| 8 | BLOOD | NBPF10 | 1 | 145365372 | 145365372 | C | G | nonsynonymous | NM_001039703 | c.C8527G | p.L2843V |
| 8 | BLOOD | INSRR | 1 | 156816461 | 156816461 | G | A | nonsynonymous | NM_014215 | c.C1660T | p.R554C |
| 8 | BLOOD | INSRR | 1 | 156821885 | 156821885 | A | G | nonsynonymous | NM_014215 | c.T736C | p.C246R |
| 8 | BLOOD | CEP170 | 1 | 243328887 | 243328887 | G | T | stopgain | NM_014812 | c.C2375A | p.S792X |
| 8 | BLOOD | CTTNBP2 | 7 | 117513445 | 117513445 | C | T | nonsynonymous | NM_033427 | c.G25A | p.E9K |
| 8 | BLOOD | GSN | 9 | 124088950 | 124088950 | G | A | nonsynonymous | NM_000177 | c.G1730A | p.R577Q |
| 8 | BLOOD | SLC34A3 | 9 | 140128564 | 140128564 | G | A | nonsynonymous | NM_001177316 | c.G929A | p.R310H |
| 8 | BLOOD | TRUB1 | 10 | 116698112 | 116698112 | A | C | nonsynonymous | NM_139169 | c.A100C | p.T34P |
| 8 | BLOOD | HERC2 | 15 | 28518046 | 28518046 | G | A | nonsynonymous | NM_004667 | c.C905T | p.T302M |
| 8 | BLOOD | ZCCHC14 | 16 | 87448079 | 87448079 | C | G | nonsynonymous | NM_015144 | c.G1133C | p.R378P |
| 8 | BLOOD | MAMSTR | 19 | 49216607 | 49216607 | G | A | nonsynonymous | NM_001130915 | c.C1165T | p.P389S |
| 8 | BLOOD | IGSF5 | 21 | 41173190 | 41173190 | G | A | nonsynonymous | NM_001080444 | c.G1130A | p.R377Q |
| 90 | BLOOD | ANXA6 | 5 | 150496701 | 150496701 | C | T | nonsynonymous | NM_001155 | c.G1559A | p.R520Q |
| 90 | BLOOD | CNTNAP3B | 9 | 43822686 | 43822686 | C | A | nonsynonymous | NM_001201380 | c.C1240A | p.R414S |
| 90 | BLOOD | C16orf59 | 16 | 2512504 | 2512504 | G | A | nonsynonymous | NM_025108 | c.G839A | p.R280H |
| 90 | BLOOD | PIK3R5 | 17 | 8794074 | 8794074 | C | G | nonsynonymous | NM_001142633 | c.G638C | p.G213A |
| 90 | BLOOD | ARSD | X | 2833605 | 2833605 | C | T | stopgain | NM_001669 | c.G992A | p.W331X |
| 90 | BLOOD | ARSD | X | 2833638 | 2833638 | C | T | nonsynonymous | NM_001669 | c.G959A | p.G320D |
| 90 | BLOOD | ARSD | X | 2833643 | 2833643 | C | A | nonsynonymous | NM_001669 | c.G954T | p.Q318H |
| 90 | BLOOD | ARSD | X | 2835863 | 2835863 | G | T | nonsynonymous | NM_001669 | c.C845A | p.A282D |
| 90 | BLOOD | ARSD | X | 2836211 | 2836211 | A | T | nonsynonymous | NM_001669 | c.T497A | p.L166Q |
| 91 | LUNG | PTH1R | 3 | 46940255 | 46940255 | G | T | nonsynonymous | NM_000316 | c.G742T | p.A248S |
| 92 | LUNG | MARCKS | 6 | 114181301 | 114181301 | - | TGAGGC | inframe | NM_002356 | c.545_546insTGAGGC | p.G182delinsGEA |
| 92 | LUNG | C17orf97 | 17 | 263412 | 263441 | GAGGCCCTCAAGGGCTTCCACCCCGACCCT | - | inframe | NM_001013672 | c.778_807del | p.260_269del |
| 92 | LUNG | MUC4 | 3 | 195505788 | 195505788 | G | C | nonsynonymous | NM_018406 | c.C12663G | p.H4221Q |
| 93 | LUNG | RBM5 | 3 | 50155888 | 50155889 | GA | - | frameshift | NM_005778 | c.2447_2448del | p.*816fs |
| 93 | LUNG | VCX2 | X | 8138159 | 8138164 | CTTCCA | - | inframe | NM_016378 | c.329_334del | p.110_112del |
| 93 | LUNG | SH2D5 | 1 | 21050899 | 21050899 | A | C | nonsynonymous | NM_001103161 | c.T620G | p.V207G |
| 93 | LUNG | ANKRD36 | 2 | 97877478 | 97877478 | G | A | nonsynonymous | NM_001164315 | c.G3469A | p.V1157M |
| 93 | LUNG | STOX2 | 4 | 184932245 | 184932245 | C | A | nonsynonymous | NM_020225 | c.C2254A | p.Q752K |
| 94 | LUNG | KRTAP5-1 | 11 | 1606121 | 1606150 | CCACAGCCACCCTTGGATCCCCCACAAGAG | - | inframe | NM_001005922 | c.330_359del | p.110_120del |
| 94 | LUNG | SLMAP | 3 | 57850537 | 57850537 | G | A | nonsynonymous | NM_001304420 | c.G1139A | p.R380Q |
| 94 | LUNG | UPK3B | 7 | 76140210 | 76140210 | G | A | nonsynonymous | NM_030570 | c.G241A | p.V81M |
| 94 | LUNG | PVRIG | 7 | 99817805 | 99817805 | G | T | nonsynonymous | NM_024070 | c.G187T | p.G63W |
| 94 | LUNG | TLL2 | 10 | 98172999 | 98172999 | C | T | nonsynonymous | NM_012465 | c.G998A | p.R333Q |
| 95 | LUNG | MICA;MICA | 6 | 31380161 | 31380161 | G | - | frameshift | NM_001177519 | c.952delG | p.G318fs |
| 95 | LUNG | BAIAP2L2 | 22 | 38483155 | 38483155 | - | TCATGGGTG | inframe | NM_025045 | c.1234_1235insCACCCATGA | p.N412delinsTPMN |
| 96 | LUNG | KRTAP4-5 | 17 | 39305775 | 39305775 | - | GGCAGCAGCTGGGGC | inframe | NM_033188 | c.244_245insGCCCCAGCTGCTGCC | p.Q82delinsRPSCCQ |
| 96 | LUNG | TBC1D9B | 5 | 179298526 | 179298526 | C | T | nonsynonymous | NM_198868 | c.G2420A | p.R807Q |
| 96 | LUNG | PLP2 | X | 49028363 | 49028363 | C | T | nonsynonymous | NM_002668 | c.C16T | p.R6C |
| 97 | LUNG | RNGTT | 6 | 89638988 | 89638988 | - | A | splicing |  |  |  |
| 97 | LUNG | MFSD12 | 19 | 3551140 | 3551140 | G | - | frameshift | NM_174983 | c.351delC | p.G117fs |
| 97 | LUNG | MET | 7 | 116411923 | 116411923 | C | T | nonsynonymous | NM_001127500 | c.C2962T | p.R988C |
| 97 | LUNG | GLIS3 | 9 | 4118388 | 4118388 | G | A | nonsynonymous | NM_152629 | c.C625T | p.P209S |
| 97 | LUNG | SAA2,SAA2-SAA4 | 11 | 18267478 | 18267478 | G | A | nonsynonymous | NM_030754 | c.C209T | p.A70V |
| 97 | LUNG | DTD2 | 14 | 31926584 | 31926584 | G | A | nonsynonymous | NM_080664 | c.C16T | p.R6W |
| 98 | LUNG | NADK | 1 | 1690614 | 1690614 | G | C | stopgain | NM_001198994 | c.C501G | p.Y167X |
| 98 | LUNG | ETV7 | 6 | 36341244 | 36341244 | G | T | nonsynonymous | NM_016135 | c.C419A | p.P140Q |
| 98 | LUNG | IGDCC4 | 15 | 65678318 | 65678318 | T | G | nonsynonymous | NM_020962 | c.A3031C | p.S1011R |
| 98 | LUNG | CDC27 | 17 | 45214528 | 45214528 | A | T | nonsynonymous | NM_001256 | c.T1903A | p.Y635N |
| 98 | LUNG | SLC35G4 | 18 | 11610382 | 11610382 | C | A | nonsynonymous | NM_001282300 | c.C788A | p.T263N |
| 99 | BLOOD | CCDC180 | 9 | 100092968 | 100092968 | - | GAGGAG | inframe | NM_020893 | c.2325_2326insGAGGAG | p.E775delinsEEE |
| 99 | BLOOD | FAM90A1 | 12 | 8374781 | 8374781 | - | ACG | inframe | NM_018088 | c.1031_1032insCGT | p.T344delinsTV |
| 99 | BLOOD | CBX4 | 17 | 77807917 | 77807917 | - | GCCGCC | inframe | NM_003655 | c.1523_1524insGGCGGC | p.A508delinsAAA |
| 9 | BLOOD | NAV1 | 1 | 201617945 | 201617945 | - | CGG | inframe | NM_020443 | c.149_150insCGG | p.P50delinsPG |
| 9 | BLOOD | ACIN1 | 14 | 23548783 | 23548783 | - | GAACGT | inframe | NM_014977 | c.1934_1935insACGTTC | p.S645delinsSRS |
| 9 | BLOOD | MUC5B | 11 | 1253976 | 1253976 | A | G | nonsynonymous | NM_002458 | c.A2041G | p.S681G |
| 9 | BLOOD | CACNA1H | 16 | 1254361 | 1254361 | A | T | nonsynonymous | NM_021098 | c.A2354T | p.K785M |
| 9 | BLOOD | NAA38 | 17 | 7760574 | 7760574 | C | T | stopgain | NM_032356 | c.G168A | p.W56X |
| 9 | BLOOD | ZNRF3 | 22 | 29446903 | 29446903 | C | A | nonsynonymous | NM_001206998 | c.C2734A | p.Q912K |
| 9 | BLOOD | P2RY4 | X | 69479204 | 69479204 | G | T | nonsynonymous | NM_002565 | c.C271A | p.L91I |
